# Statistical Mechanics of Linear *k*-mer Lattice Gases: From Theory to Applications

**DOI:** 10.3390/e27070750

**Published:** 2025-07-14

**Authors:** Julian Jose Riccardo, Pedro Marcelo Pasinetti, Jose Luis Riccardo, Antonio Jose Ramirez-Pastor

**Affiliations:** Departamento de Física, Instituto de Física Aplicada, Universidad Nacional de San Luis-CONICET, Ejército de Los Andes 950, San Luis D5700BWS, Argentina; julianriccardo@gmail.com (J.J.R.); mapasi@gmail.com (P.M.P.)

**Keywords:** multisite occupancy adsorption, lattice–gas models, statistical thermodynamics, exclusion statistics

## Abstract

The statistical mechanics of structured particles with arbitrary size and shape adsorbed onto discrete lattices presents a longstanding theoretical challenge, mainly due to complex spatial correlations and entropic effects that emerge at finite densities. Even for simplified systems such as hard-core linear *k*-mers, exact solutions remain limited to low-dimensional or highly constrained cases. In this review, we summarize the main theoretical approaches developed by our research group over the past three decades to describe adsorption phenomena involving linear *k*-mers—also known as multisite occupancy adsorption—on regular lattices. We examine modern approximations such as an extension to two dimensions of the exact thermodynamic functions obtained in one dimension, the Fractional Statistical Theory of Adsorption based on Haldane’s fractional statistics, and the so-called Occupation Balance based on expansion of the reciprocal of the fugacity, and hybrid approaches such as the semi-empirical model obtained by combining exact one-dimensional calculations and the Guggenheim–DiMarzio approach. For interacting systems, statistical thermodynamics is explored within generalized Bragg–Williams and quasi-chemical frameworks. Particular focus is given to the recently proposed Multiple Exclusion statistics, which capture the correlated exclusion effects inherent to non-monomeric particles. Applications to monolayer and multilayer adsorption are analyzed, with relevance to hydrocarbon separation technologies. Finally, computational strategies, including advanced Monte Carlo techniques, are reviewed in the context of high-density regimes. This work provides a unified framework for understanding entropic and cooperative effects in lattice-adsorbed polyatomic systems and highlights promising directions for future theoretical and computational research.

## 1. Introduction

Understanding the statistical mechanics of structured particles with arbitrary size and shape in external fields remains a major theoretical challenge, largely due to the complex entropic contributions arising from particle configurations at finite densities. Even for simplified models, such as linear particles with hard-core interactions on regular lattices, the problem is generally analytically intractable. This difficulty stems from spatial correlations among allowed particle configurations, which complicate the calculation of thermodynamic potentials. These correlations underlie various emergent collective behaviors, including nematic ordering in systems of linear *k*-mers [1] and entropy-driven competition in multicomponent mixtures. Exact solutions have been found only in a few special cases, such as dimers on square lattices [2] and hexagons on regular lattices [3].

In continuum systems, this problem has been extensively studied. In three-dimensional colloidal suspensions, Onsager famously demonstrated that elongated molecules undergo a phase transition from an isotropic to a nematic phase [1]. In two dimensions, although continuous rotational symmetry cannot be spontaneously broken, a Kosterlitz–Thouless transition occurs, which is characterized by a power-law decay in orientational correlations [4,5].

In contrast, the case of hard-core particles on lattices is less well understood. Early work by Flory [6] and Huggins [7] initiated the study of rigid rods, or *k*-mers, modeled as linear arrangements of *k* identical units occupying contiguous lattice sites. These rods interact solely through hard-core exclusion, meaning that no site may be occupied by more than one unit.

The Flory–Huggins (FH) theory, developed independently by Flory [6] and Huggins [7], generalizes the theory of binary liquid mixtures or dilute polymer solutions on lattices. In the lattice–gas framework, the adsorption of *k*-mers on homogeneous surfaces is formally analogous to polymer–solvent binary solutions.

Considerable work has been devoted to evaluating the Flory–Huggins (FH) theory in comparison with experimental data. While it may not always yield quantitatively precise predictions, the theory has proven to be qualitatively—and often semi-quantitatively—reliable. It is widely acknowledged that this foundational model captures the key characteristics that differentiate polymer solutions from those involving small molecules. Over time, several refined versions of the FH model have been introduced. A detailed analysis of these developments can be found in the book by Des Cloizeaux and Jannink [8].

The FH framework, originally formulated for molecules of arbitrary shape assuming an isotropic spatial distribution, offers a natural basis for incorporating the orientational behavior of adsorbed molecules. In this context, DiMarzio [9] proposed an approximate method for calculating the total number of configurations, Ω, for packing linear polymer chains of various shapes and orientations. His approach evaluates Ω by accounting for the distribution of molecules along allowed directions. These directions may either form a continuous set—where the configuration count depends on a density function f(r) describing the number of rods within a differential solid angle Δr—or be discrete, as in lattice-based models. Leveraging detailed information about molecular orientation, DiMarzio’s approach provides insight into different liquid crystalline phases, including nematic, smectic, and cholesteric structures, and offers theoretical justification for their formation. When the model is restricted to orientations that precisely conform to lattice directions under an isotropic assumption, it converges to a result originally derived by Guggenheim [10], which is now referred to as the Guggenheim–DiMarzio (GD) approximation.

In the 2000s, two novel approaches were proposed for the description of multisite adsorption. The first, developed by Ramirez-Pastor et al. [11], introduced the Extension Ansatz (EA) model for linear adsorbates on homogeneous surfaces, based on exact one-dimensional thermodynamic expressions and their generalization to higher dimensions. The second, the Fractional Statistical Theory of Adsorption (FSTA) [12,13], incorporates the internal configuration of the adsorbed molecule as a model parameter. The FSTA generalizes Haldane’s fractional exclusion statistics [14,15], originally developed for quantum systems, to describe classical polyatomic adsorption at gas–solid interfaces.

Comparisons with simulation data [11] have shown that the GD approximation agrees well at low surface coverage, while the EA model performs better at high coverage. These insights led to the development of the Semi-empirical (SE) Model for Polyatomic Adsorption [11,16], a hybrid model combining exact 1D results with appropriately weighted GD approximations.

More recently, the Multiple Exclusion (ME) statistics framework was introduced to describe classical systems in which particles access spatially correlated states [17,18]. ME statistics account for situations in which multiple particles simultaneously exclude access to a common state—an intrinsic feature of non-monomeric particles on a lattice. The uncorrelated limit of ME statistics recovers both the Haldane–Wu and FSTA formalisms. This approach was further extended in Ref. [19] to mixtures of particles with arbitrary shapes and sizes, allowing for analytical expressions of thermodynamic quantities in terms of coverage and species densities.

Although numerous studies have explored the adsorption of polyatomic species on discrete lattice structures, several important issues remain unresolved. Even though the theoretical framework for such problems is well-defined, obtaining exact analytical solutions for systems of correlated adsorbates, such as *k*-mers, has historically proven to be a major challenge. Exact results are largely confined to one-dimensional systems [20], with only a few known configurations in higher dimensions yielding tractable solutions. A well-known case is the dimer lattice–gas model (k=2), which has been extensively analyzed in the literature [2,21,22,23,24,25,26,27,28]. For a comprehensive review on the entropy associated with close-packed dimers on two-dimensional lattices, see Ref. [29].

The difficulty of solving the *k*-mer adsorption problem grows significantly when one incorporates additional features such as multisite occupancy combined with lateral interactions or surface heterogeneity. In such scenarios, even approximate treatments of the thermodynamic properties become complex. Therefore, simple solvable models on uniform surfaces are particularly useful as reference systems for constructing approximate or semi-empirical methods that can later be extended to more intricate systems, including those with interacting adsorbed molecules [30,31] or structurally heterogeneous surfaces [32,33,34,35,36,37].

In this work, we present an overview of recent theoretical developments in the modeling of structured particle adsorption on regular lattices (commonly referred to as multisite occupancy adsorption). We focus on how the geometry and size of particles affect the configurational entropy of the adsorbed layer—an aspect that has rarely been systematically treated in thermodynamic models. Understanding entropic effects in polyatomic systems is particularly relevant for applications such as alkane and hydrocarbon adsorption, which are key to petrochemical separation technologies.

While the majority of this review focuses on hard-core models, in which particle overlap is strictly forbidden, it is important to acknowledge both the substantial body of previous work and the growing interest in developing models for soft particle adsorption [38,39,40,41,42,43,44,45]. In soft particle models, particles can overlap or interact more flexibly, offering a more realistic description of many physical systems, such as polymers or biological macromolecules on surfaces. Recent studies have investigated the effective surface coverage of coarse-grained soft matter [46], as well as the non-equilibrium structures emerging from sequential adsorption processes [47]. These models serve as valuable complements to hard-core approaches, providing deeper insight into systems where entropic and energetic contributions are more subtle and interdependent.

This review aims to synthesize our group’s contributions and present an overview of the computational methodologies we have developed over the past thirty years, with a particular emphasis on linear *k*-mers adsorbed onto regular lattices. Our work has provided insights into the entropic and cooperative effects inherent to these systems, contributing to a better understanding of entropy density dependence and phenomena such as phase transitions. While our primary focus is on linear *k*-mers, we recognize that the broader field of lattice gas statistical mechanics encompasses a wealth of substantial research on other particle shapes, such as squares, rectangles, triangles, branched structures, and flexible chains, on both regular and Bethe-like lattices [48,49,50,51,52,53,54,55,56,57,58,59,60,61,62,63,64,65,66,67,68,69]. These studies, although falling outside the scope of our current discussion, offer complementary valuable perspectives on the complex behaviors of polyatomic systems on lattices.

The remainder of this paper is structured as follows: Section 2 examines the thermodynamics of one-dimensional lattice gases in the multilayer regime. Section 3 presents theoretical approximations for non-interacting polyatomic species in two dimensions, including the EA extension of one-dimensional results, the FSTA framework based on fractional statistics, the Occupation Balance (OB) approximation, and the SE model. Adsorption of single and multicomponent species is discussed in both monolayer and multilayer contexts.

Section 4 explores two-dimensional lattice gases of interacting structured species via mean-field and quasi-chemical approaches. Intermolecular interactions give rise to possible phase transitions. Section 5 introduces the ME statistics framework for classical lattice gases of arbitrarily shaped particles, generalizing the formalism of multiple exclusion statistics presented in Ref. [18]. Section 6 extends ME statistics to multicomponent systems, analytically describing the exclusion spectra in terms of lattice coverage and species densities. This can be considered as a suitable framework to address complex lattice–gas mixtures where spatial state correlations are significant for understanding their phase behavior. Section 7 discusses applications of the main theoretical models developed in this review, comparing model predictions with Monte Carlo simulations and experimental data. Section 8 focuses on computational methods. From a computational standpoint, analyzing the statistical behavior of polyatomic adsorbates presents significant challenges. While monomer systems (k=1) typically reach thermal equilibrium efficiently using conventional MC adsorption–desorption algorithms, the relaxation times for larger adsorbates increase sharply with growing surface coverage. As a result, simulations at high densities become computationally intensive and may yield inaccurate results due to incomplete equilibration. To address these limitations, advanced MC methods incorporating cluster-based moves have been introduced in the literature. These improved algorithms have enabled researchers to explore the system’s properties more effectively in dense regimes. In Section 8, the main computational algorithms of interest for the study of adsorption problems involving multiple-site occupancy are presented. Most of these algorithms have been used throughout the present work. Section 9 presents our conclusions and future perspectives. Finally, Appendix A provides a summary of the main models and methods discussed throughout this review

## 2. Thermodynamic Functions of Lattice Gases of Polyatomics in One Dimension: Multilayer Adsorption

Multilayer adsorption has long been a subject of significant scientific interest [70,71,72,73], and advancements in this area have been especially important for the characterization of solid surfaces. Numerous theoretical models have been formulated to describe equilibrium multilayer adsorption processes [74,75,76,77,78], with the Brunauer–Emmett–Teller (BET) model [78] being among the most frequently employed due to its practicality and simplicity.

The BET theory is based on the assumption that adsorbate molecules are monomers, each occupying a single adsorption site. Once a monolayer forms, additional molecules can adsorb atop previously adsorbed ones, forming subsequent layers. This model disregards lateral interactions and treats the adsorption energy for layers beyond the first as uniform, typically set equal to the enthalpy of condensation for the adsorbate. Its success lies in its straightforward adsorption isotherm, few adjustable parameters, and the physical interpretability of those parameters, making it a powerful tool for determining surface area and adsorption energies of solids.

To preserve the simplicity of the BET model while extending it to consider multisite occupancy in multilayer adsorption, we adopt a minimal extension inspired by its original formulation. The substrate is modeled as a uniform one-dimensional lattice and the adsorbing species are linear chains made up of *k* identical units (*k*-mers), each occupying one site. The following assumptions are introduced: (*i*) adsorption of a *k*-mer can occur directly atop another already adsorbed molecule; (ii) lateral interactions are neglected; (iii) the adsorption energy in all layers beyond the first is constant and equivalent to the molar enthalpy of condensation of the bulk fluid. Consequently, the parameter $c=q_{1}/q_{i}=q_{1}/q$ with $q_{i}=q\;\left(i=2,\ldots ,\infty \right)$ expresses the ratio between the single-molecule partition functions in the first and subsequent layers.

**Figure 1:**
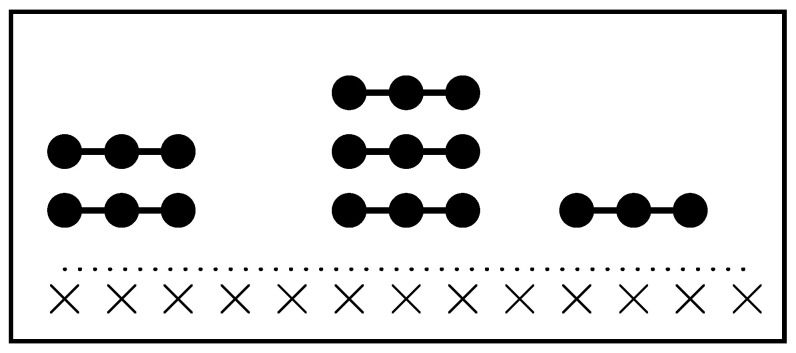
Schematic illustration of the lattice gas model for *k*-mer adsorption under multilayer conditions (trimer adsorption is shown as an example). Adsorption sites are marked with crosses on the substrate, and the adsorbed molecules are represented as sequences of black spheres linked by solid lines.

An additional layer of complexity arises from the spatial constraints in the first layer: *k*-mers leave behind sequences of unoccupied sites of length l<k, where no new *k*-mer can adsorb. This spatial exclusion introduces considerable complexity when calculating the configurational entropy compared to the monomer case, as illustrated in Figure 1.

In a lattice containing *M* available adsorption sites, the maximum number of vertical columns that can be formed is $n_{M}=M/k$. Let us define $\Omega_{k}\left(n,M\right)$ as the number of distinct ways to arrange *n* such columns across the *M* sites. When the surface permits unlimited stacking of layers, the grand partition function for the adsorbed phase in equilibrium with a gas reservoir at chemical potential μ and temperature *T* is expressed as:

(1) $$\Xi \left(\lambda ,M\right)=\sum_{n=0}^{n_{M}=M/k}\Omega_{k}\left(n,M\right)\;\;\xi^{n}\left(\lambda \right),$$

where $\lambda =\exp(\mu /k_{B}T)$ is the fugacity and μ, *T*, and $k_{B}$ denote the chemical potential, temperature, and Boltzmann constant, respectively. Furthermore,

(2) $$\Omega_{k}\left(n,M\right)=\left[\begin{array}{c} M-(k-1)n\\ n \end{array}\right]=\frac{\left[M-(k-1)n\right]!}{n!(M-kn)!},$$

and

(3) $$\xi \left(\lambda \right)=\sum_{i=1}^{\infty }q_{1}q^{i-1}\lambda^{i}=c\sum_{i=1}^{\infty }q^{i}\lambda^{i}=\frac{c\lambda q}{1-\lambda q}$$

represents the grand partition function corresponding to a single vertical column containing at least one *k*-mer in the first adsorption layer.

As direct evaluation of the summation in Equation (1) is not feasible for k>1, we adopt the conventional approach of identifying and evaluating the dominant contribution to the sum.

Thus, using the Stirling’s approximation lnn!=nlnn−n,

(4) $$\ln\left[\Omega_{k}\left(n,M\right)\xi^{n}\right]=\left[M-(k-1)n\right]\ln\left[M-(k-1)n\right]-n\ln n-\left(M-kn\right)\ln\left(M-kn\right)+n\ln\xi ,$$

and

(5) $$\frac{\partial \left\{\ln\left[\Omega_{k}\left(n,M\right)\xi^{n}\right]\right\}}{\partial n}=-\left(k-1\right)\ln\left[M-(k-1)n\right]+k\ln\left(M-kn\right)+\ln\left(\frac{\xi }{n}\right)=0,$$

(6) $$\ln\left\{{\left[\frac{M-kn}{M-(k-1)n}\right]}^{k}\frac{\xi }{n}\left[M-(k-1)n\right]\right\}=0,$$

which leads to the following nonlinear equation

(7) $${\left(M-kn\right)}^{k}=\frac{n}{\xi }{\left[M-(k-1)n\right]}^{k-1}.$$

Resolving $\left(M-kn\right)={\left(n/\xi \right)}^{1/k}{\left[M-(k-1)n\right]}^{(k-1)/k}$ from Equation (7) and replacing it in Equation (1), one obtains

(8) $$\ln\Xi \left(\lambda ,M\right)=\frac{M}{k}\ln\left\{\frac{\xi }{n}\left[M-(k-1)n\right]\right\}.$$

From Equation (8), it can be implicitly understood that the value of *n* on the right-hand side corresponds to the most probable configuration; i.e., the term that maximizes the grand partition function in Equation (1). Consequently, Equations (7) and (8) serve as the foundational expressions for determining the thermodynamic behavior of *k*-mers in the multilayer adsorption regime.

The corresponding thermodynamic quantities can be readily obtained using the grand canonical ensemble framework. In this context,

(9) $$\bar{n}=\lambda {\left(\frac{\partial \ln\Xi }{\partial \lambda }\right)}_{T,M},$$

(10) $$s=k_{B}T{\left(\frac{\partial \ln\Xi }{\partial T}\right)}_{M,\mu }+k\ln\Xi ,$$

(11) $$e=-{\left(\frac{\partial \ln\Xi }{\partial \beta }\right)}_{M,\lambda },$$

where $\bar{n}$ represents the average number of adsorbed columns, while *s* and *e* denote the entropy and internal energy per site, respectively. Based on the standard definition, the surface coverage θ, the fraction of the lattice occupied by adsorbed units, follows directly as:

(12) $$\theta =\frac{k\bar{n}}{M}=\frac{k\lambda }{M}{\left(\frac{\partial \ln\Xi }{\partial \lambda }\right)}_{T,M}.$$

In the case of adsorbed monomers (k=1), (Mξ/n)=(1+ξ) from Equation (7) and

(13) lnΞ(λ,M)=Mln(1+ξ).

From Equation (9), we have

(14) $$\bar{n}=\lambda {\left(\frac{\partial \ln\Xi }{\partial \lambda }\right)}_{T,M}=\frac{\lambda \xi^{\prime }}{1+\xi }M,$$

where $\xi^{\prime }=d\xi /d\lambda =cq/{\left(1-\lambda q\right)}^{2}$. Finally, the surface coverage satisfies

(15) $$\theta =\frac{\bar{n}}{M}=\frac{cq\lambda }{\left(1-\lambda q)[1+(c-1)\lambda q\right]},$$

which corresponds to the well-known BET isotherm equation.

### Multilayer Adsorption of Dimers

In this section, we examine multilayer adsorption of a homonuclear dimer as the simplest example of a polyatomic adsorbate. This model not only enhances the theoretical framework by incorporating the influence of adsorbate size on the thermodynamics, but also offers practical improvements in describing experimental adsorption processes—especially for nitrogen, a commonly used probe in surface area analysis of non-porous materials. A more accurate model could improve the determination of key surface parameters such as adsorption energy and surface area.

For dimers, the Equation (7) reads

(16) $${\left(M-2n\right)}^{2}=\frac{n}{\xi }\left(M-n\right)=\frac{n}{\xi }\left[\left(M-2n\right)+n\right].$$

Denoting z=(M−2n), Equation (16) can be rewritten as

(17) $$z^{2}-\frac{n}{\xi }z-\frac{n^{2}}{\xi }=0.$$

Only one of the solutions of Equation (17) remains due to physical reasons (z≥0),

(18) $$z=\frac{n}{2\xi }+\frac{1}{2}\sqrt{\frac{n^{2}}{\xi^{2}}+\frac{4n^{2}}{\xi }}=\frac{n}{\xi }\left(\frac{1+\sqrt{1+4\xi }}{2}\right).$$

From Equation (8),

(19) $$\begin{array}{ccc} \ln\Xi (\lambda ,M) & = & \frac{M}{2}\ln\left[\frac{(M-n)\xi }{n}\right]\\ & = & \frac{M}{2}\ln\left[\left(M-2n\right)\frac{\xi }{n}+\xi \right] \end{array}$$

which, from the definition of *z* and Equation (18), results

(20) $$\begin{array}{ccc} \ln\Xi (\lambda ,M) & = & \frac{M}{2}\ln\left(\frac{1}{2}+\xi +\frac{\sqrt{1+4\xi }}{2}\right). \end{array}$$

The mean number of adsorbed particles $\bar{n}$ is [Equation (9)]

(21) $$\begin{array}{ccc} \bar{n} & = & \frac{M}{2}\lambda \frac{\left[\xi^{\prime }+\frac{\xi^{\prime }}{\sqrt{1+4\xi }}\right]}{\left(\frac{1}{2}+\xi +\frac{\sqrt{1+4\xi }}{2}\right)}. \end{array}$$

Finally, after some algebra, the adsorption isotherm becomes

(22) $$\theta =\frac{1}{\left(1-\lambda q\right)}\left\{1-{\left[\frac{\left(1-\lambda q\right)}{(4c-1)\lambda q+1}\right]}^{1/2}\right\}.$$

Assuming $\lambda q=P/cP_{H}$ and substituting into Equation (22), the parameter $P_{H}$ naturally emerges as Henry’s law constant, inferred from the low-pressure limit of the coverage θ.

To facilitate comparison with the classical BET theory, we consider an ideal gas phase and assume that the adsorbed molecules in layers beyond the first behave similarly to those in the bulk liquid; that is, $q=q_{l}$, where $q_{l}$ is the molecular partition function for the liquid phase. Thus,

(23) $$\lambda =\Lambda^{3}P/k_{B}T,$$

where $\Lambda^{3}={\left(h^{2}/2\pi mk_{B}T\right)}^{1/2}$ and

(24) $$cP_{H}=\frac{P}{\lambda q}=\frac{k_{B}T}{\Lambda^{3}q}=P_{0},$$

m,P, and $P_{0}$ being the molecular mass of the adsorbate, the gas pressure, and the saturation pressure of the bulk liquid, respectively.

Then,

(25) $$\theta =\frac{1}{\left(1-P/P_{0}\right)}\left\{1-{\left[\frac{\left(1-P/P_{0}\right)}{\left(4c-1\right)P/P_{0}+1}\right]}^{1/2}\right\}.$$

**Figure 2:**
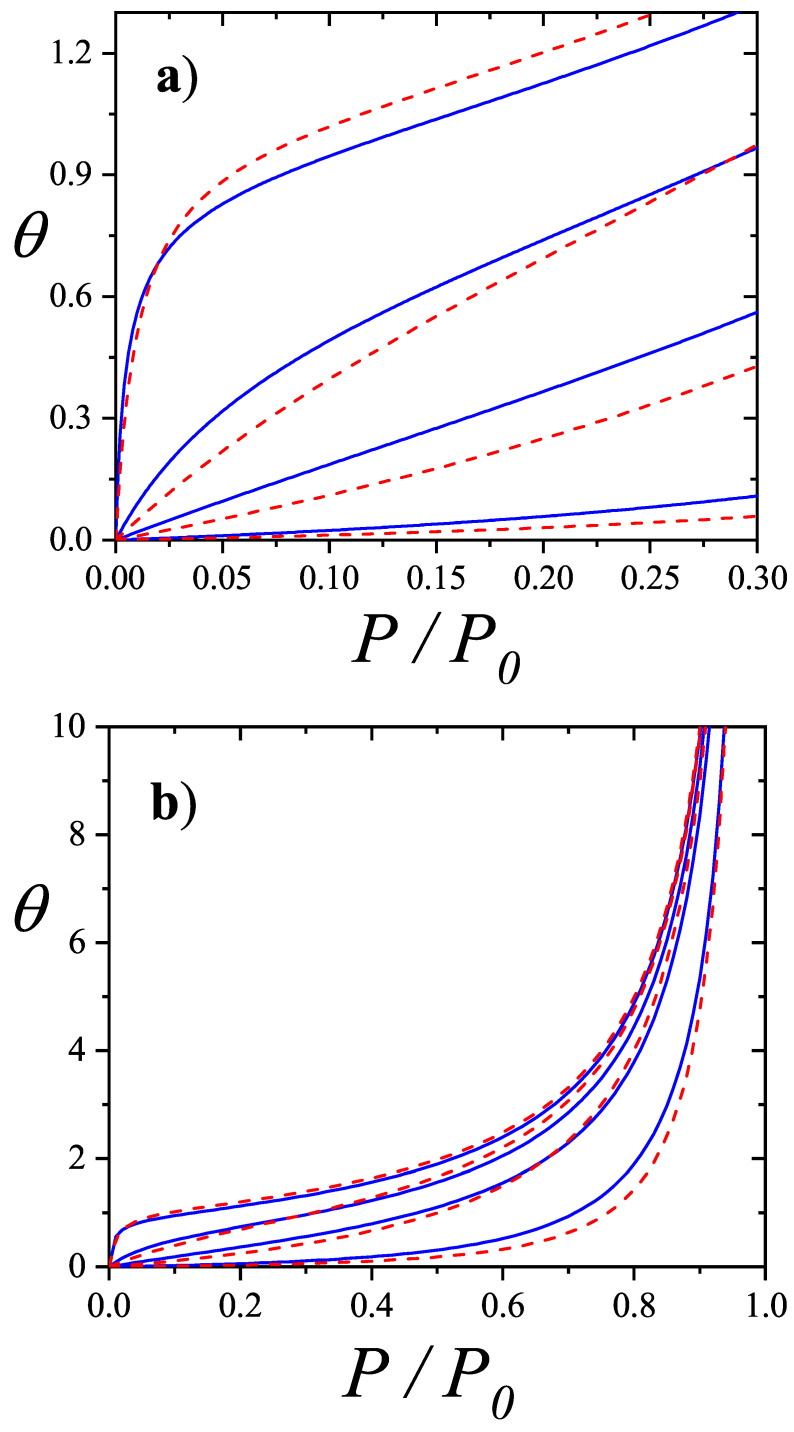
Adsorption isotherms for various values of the parameter *c*. The solid curves represent the isotherms for dimers obtained in this study [Equation (25)], while the dashed curves correspond to the BET isotherm (monomers). The pairs of curves, from bottom to top, correspond to c=0.1,1,5, and 100, in the ranges 0−0.3 of $P/P_{0}$ in (**a**) and 0−1 of $P/P_{0}$ in (**b**).

The adsorption isotherm Equation (25) is shown in Figure 2a,b for small, medium, and large values of the parameter *c*, in comparison with the BET isotherm

(26) $$\theta =\frac{c_{BET}\;P/P_{0}}{\left(1-P/P_{0}\right)\;\left(1+\left(c_{BET}-1\right)\;P/P_{0}\right)}.$$

The derived isotherm reflects two types of behavior: for c≫1, it shows type-II characteristics while, for c≪1, the isotherm resembles type-III. Regardless of *c*, the curves generated by the dimer model differ both qualitatively and quantitatively from the BET prediction, and they intersect across the full range of $P/P_{0}$.

Similar to the BET model, the dimer isotherm can also be written in a quasi-linear form. Using $\theta =v/v_{m}$, where *v* is the volume of gas adsorbed and $v_{m}$ represents the monolayer capacity, we obtain an expression from Equations (25) and (26) that corresponds to the dimer-based multilayer adsorption,

(27) $$\frac{P/P_{0}}{v(1-P/P_{0})}=\frac{P/P_{0}}{v_{m}}{\left\{1-{\left[\frac{\left(1-P/P_{0}\right)}{\left(4c-1\right)P/P_{0}+1}\right]}^{1/2}\right\}}^{-1},$$

and

(28) $$\frac{P/P_{0}}{v\left(1-P/P_{0}\right)}=\frac{1}{c_{BET}\;\;v_{m,BET}}+\frac{\left(c_{BET}-1\right)}{c_{BET}\;\;v_{m,BET}}P/P_{0}.$$

**Figure 3:**
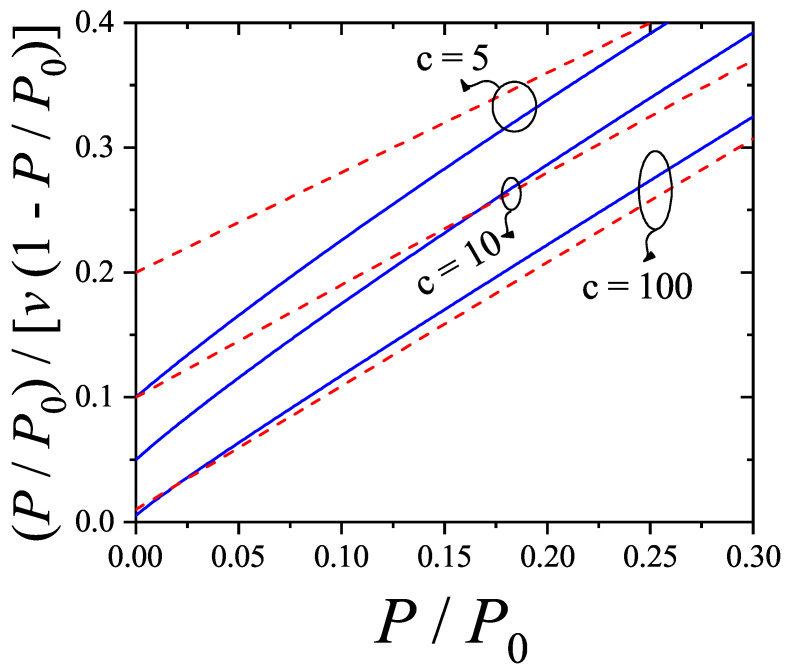
Plot of $\left[P/P_{0}\right]/\left[v\;(1-P/P_{0})\right]$ as a function of $P/P_{0}$ for $v_{m}=1$ (arbitrary units) and c=100, 10, and 5 (from bottom to top). The solid and dashed lines refer to the models depicted in Figure 2.

Unlike the BET equation [Equation (28)], the resulting expression [Equation (27)] is inherently non-linear in $P/P_{0}$, as shown in Figure 3. This non-linearity becomes especially pronounced in the low-pressure region ($P/P_{0}<0.3$), leading to significant deviations between the two models. Consequently, fitting experimental data of nitrogen or other polyatomic gases using the dimer-based isotherm could yield noticeably different values of *c* and $v_{m}$ compared to BET-based estimates.

Expansion of Equation (28) about $P/P_{0}=0$ reveals that, to first order,

(29) $$\frac{P/P_{0}}{v\left(1-P/P_{0}\right)}\approx \frac{1}{2cv_{m}}+\frac{\left(3c-1\right)}{2cv_{m}}P/P_{0}.$$

Matching terms with Equation (27) results in a mapping: $c=c_{BET}/3$ and $v_{m}=\left(3/2\right)v_{m,BET}$. This correction aligns with previous findings, such as the observation that the adsorption of nitrogen onto graphite leads to surface areas about 1.22 times greater than those calculated using the BET method (see Ref. [79]). These insights emphasize that considering the polyatomic nature of adsorbates results in more accurate estimates of surface areas, especially for molecules that occupy multiple adsorption sites. It is important to note that the presence of non-linear terms in Equation (27) implies that, in practice, the relationships between *c*, $v_{m}$, and their BET counterparts will deviate from the simple ratios mentioned above when fitting actual experimental isotherms.

Moreover, the curvature observed at low pressures in the dimer model aligns well with trends seen in real adsorption data. Although such curvature is often attributed to lateral interactions or surface heterogeneity, the presented results demonstrate that configurational entropy—arising solely from the internal structure of the adsorbate—can significantly influence the adsorption behavior, even in the absence of these additional effects.

## 3. Thermodynamic Functions of Lattice Gases of Polyatomics in Two Dimensions: Analytical Approaches for Single Species and Mixtures

The phenomenon of gas adsorption onto solid surfaces has been the subject of extensive research since the early 20th century. Despite this long-standing interest, developing a comprehensive theoretical framework to describe both the equilibrium and dynamic behaviors of polyatomic adsorbates on two-dimensional surfaces remains a significant challenge in surface science [37,70,73,80,81]. One of the central difficulties in these systems—particularly when dealing with *k*-mers (i.e., molecules that span multiple lattice sites)—is accurately accounting for the configurational entropy. This involves quantifying the number of microscopic arrangements that correspond to a fixed number of adsorbed particles and available lattice sites, which directly influences the thermodynamic properties.

From a theoretical standpoint, solving the statistical mechanics of a two-dimensional lattice populated by both vacancies and *k*-mers remains an open problem. A complete analytical solution is not yet available, and researchers typically rely on approximation techniques to investigate such systems.

One of the foundational approaches to understanding the adsorption of *k*-mers on uniform surfaces was the pioneering Flory–Huggins (FH) approximation, developed independently by Flory [6] and Huggins [7]. This model extends the Bragg–Williams approximation, originally applied to binary mixtures on a two-dimensional lattice [80] to account for systems involving chain-like molecules. Over time, several modifications of the FH approximation have been proposed to address its limitations and improve its predictive capabilities. An in-depth examination of these developments can be found in the book by Des Cloizeaux and Jannink [8] and in Ref. [80].

Within the lattice–gas framework, the adsorption of pure linear chains bears a close analogy to the behavior of polymer mixtures, specifically involving linear polymers dissolved in a monomeric solvent. Guggenheim introduced an alternative method to evaluate the combinatorial contribution to the canonical partition function in such systems [10]. Building on this, DiMarzio later derived a correction factor which is applicable to rigid rod-like molecules [9], a result now commonly referred to as the Guggenheim–DiMarzio (GD) approximation.

The FH theory has been subject to significant criticism. One of the primary concerns is its reliance on the mean-field approximation, which assumes that each molecule experiences an average interaction field from its surroundings, thereby neglecting local correlations and concentration fluctuations. This simplification leads to several important shortcomings. For instance, the theory fails to accurately predict the lower critical solution temperature observed in certain polymer blends where phase separation occurs upon heating, a phenomenon well-documented in detailed polymer phase diagrams [82]. Similarly, in binary blends with polymers of equal chain length, FH theory predicts a symmetric critical concentration of 0.5, which often contradicts experimental results that indicate marked asymmetry, as discussed in scaling theories of polymer physics [83]. This discrepancy arises from the theory’s oversimplified treatment of the interaction parameter which, in reality, depends on temperature, composition, and chain length. In practice, this parameter can vary considerably with these factors, leading to noticeable deviations from experimental data, as demonstrated in statistical mechanical analyses of polymer solutions.

Another significant shortcoming is that the FH model neglects intramolecular correlations, which are particularly important in dilute solutions where interactions between distant segments of the same polymer chain become dominant and can lead to local demixing and high-concentration regions. While this effect diminishes at higher polymer concentrations, the lattice-based nature of the FH theory prevents it from accurately capturing such phenomena, as highlighted in studies of self-interacting lattice trees [84,85,86,87]. The assumption of a uniform distribution of segments and the restriction to nearest-neighbor interactions also lead to inaccuracies, especially in systems where long-range correlations or non-uniform segment distributions play a significant role, as shown in investigations of critical behavior near surfaces [88].

Furthermore, the lattice-based character of the theory limits its applicability in dilute regimes or in systems that do not conform to a lattice structure, such as aqueous solutions of hydrophobic polymers. The theory does not account for volume changes upon mixing or for important features such as chain flexibility, branching, or stiffness—factors that become critical in the thermodynamics of branched polymers and gels. For example, studies on the swelling behavior of hydrophobic polymers have revealed that FH theory tends to overestimate the critical temperatures for phase separation due to its neglect of fluctuations and correlations.

Of particular significance are the works of Gujrati [89,90,91], who made fundamental contributions to the critical understanding of the inherent limitations of classical models widely used in the statistical physics of polymer systems.

Through rigorous analysis, Gujrati demonstrated that the Flory and Flory–Huggins models systematically underestimate the combinatorial entropy of semi-flexible polymer chains [89,90]. By deriving exact lower bounds for the configurational entropy on square and cubic lattices, he showed that—contrary to the predictions of Flory–Huggins theory—the entropy remains strictly positive for any non-zero fraction of gauche bonds [91]. This result mathematically invalidates the notion of a first-order transition to a fully ordered phase at finite temperatures [90], and further challenges conclusions based on the Gibbs–DiMarzio model regarding the existence of a thermodynamic glass transition.

Despite these limitations, the FH theory continues to serve as a foundational tool for understanding the thermodynamics of polymer solutions. It offers a simple yet effective framework for modeling phase behavior and remains a useful starting point for the development of more sophisticated models. However, in systems where local correlations, complex chain architectures, or specific interactions are critical, more advanced approaches are necessary for accurate theoretical descriptions. As noted at the beginning of this section, a more detailed discussion of FH theory can be found in the book by des Cloizeaux and Jannink [8].

### 3.1. Two-Dimensional Model of Non-Interacting Structured Particles: More Recent Approximations from Our Group

In recent years, several new theoretical frameworks have been proposed by our group in order to model adsorption processes involving multisite occupancy. The first approach, referred to here as the Exact Approximation (EA), is grounded in exact expressions for the thermodynamic functions of linear molecules adsorbed onto one-dimensional lattices and their subsequent extension to higher-dimensional systems [20,92,93]. The second, known as the Fractional Statistical Theory of Adsorption (FSTA) [12,13], draws upon a generalization of Haldane’s quantum fractional statistics formalism [14,15]. A third model, termed the Occupation Balance (OB) method, involves a series expansion of the inverse fugacity [93,94,95]. Lastly, the Semi-Empirical (SE) model provides a practical approximation by blending exact one-dimensional results with insights from the Guggenheim–DiMarzio approach [11].

The EA, FSTA, OB, and SE frameworks are presented below: EA in Section 3.1.1, FSTA in Section 3.1.2, OB in Section 3.1.3, and SE in Section 3.1.4. Section 3.1.5 includes a brief reference to Multiple Exclusion Statistics, which are further developed in Section 5 and Section 6. Section 3.2 and Section 3.3 are dedicated to the analysis of the mixture and multilayer problems, respectively.

#### 3.1.1. Extension to Higher Dimensions of the Exact Thermodynamic Functions in One Dimension (EA)

Consider a *d*-dimensional lattice of connectivity γ composed of *M* equally spaced sites with lattice spacing *a* and periodic boundary conditions. This setup ensures translational symmetry, eliminating edge effects from the analysis.

We examine the case where *N* linear *k*-mers are adsorbed onto the lattice such that each segment (or monomer) of a *k*-mer occupies a single site. In addition, we consider the constraint that no lattice site can host more than one segment, thus enforcing a monolayer regime. The only interaction present is excluded volume: *k*-mers cannot overlap or share sites. As there are no further interactions between the *k*-mers themselves, all spatial arrangements of *N* such molecules over *M* sites are equally likely. Consequently, the canonical partition function Q(M,N,T) reduces to the total number of allowed configurations, Ω(M,N,γ), multiplied by the Boltzmann factor associated with the total adsorption energy, $E_{k}\left(N\right)$,

(30) $$Q\left(M,N,T\right)=\Omega \left(M,N,\gamma \right)\exp\left[-\frac{E_{k}\left(N\right)}{k_{B}T}\right].$$

Assuming the substrate is uniform, the total interaction energy can be expressed as $E_{k}\left(N\right)=k\epsilon_{0}N$, where $\epsilon_{0}$ denotes the binding energy between each monomer unit and the lattice.

In this 2D setting, we examine two distinct molecular conformations: (i) “linear *k*-mers”, which consist of *k* connected monomers aligned in a straight line, and (ii) “flexible *k*-mers”, composed of *k* contiguous monomers forming a chain. In the flexible case, the placement starts with the first monomer, while the second occupies one of the γ nearest-neighbor sites. Each subsequent monomer in the chain is placed on one of the γ−1 neighboring sites adjacent to the previous monomer, ensuring that no overlaps occur. This sequential placement continues until all *k* units are placed on the lattice.

**Figure 4:**
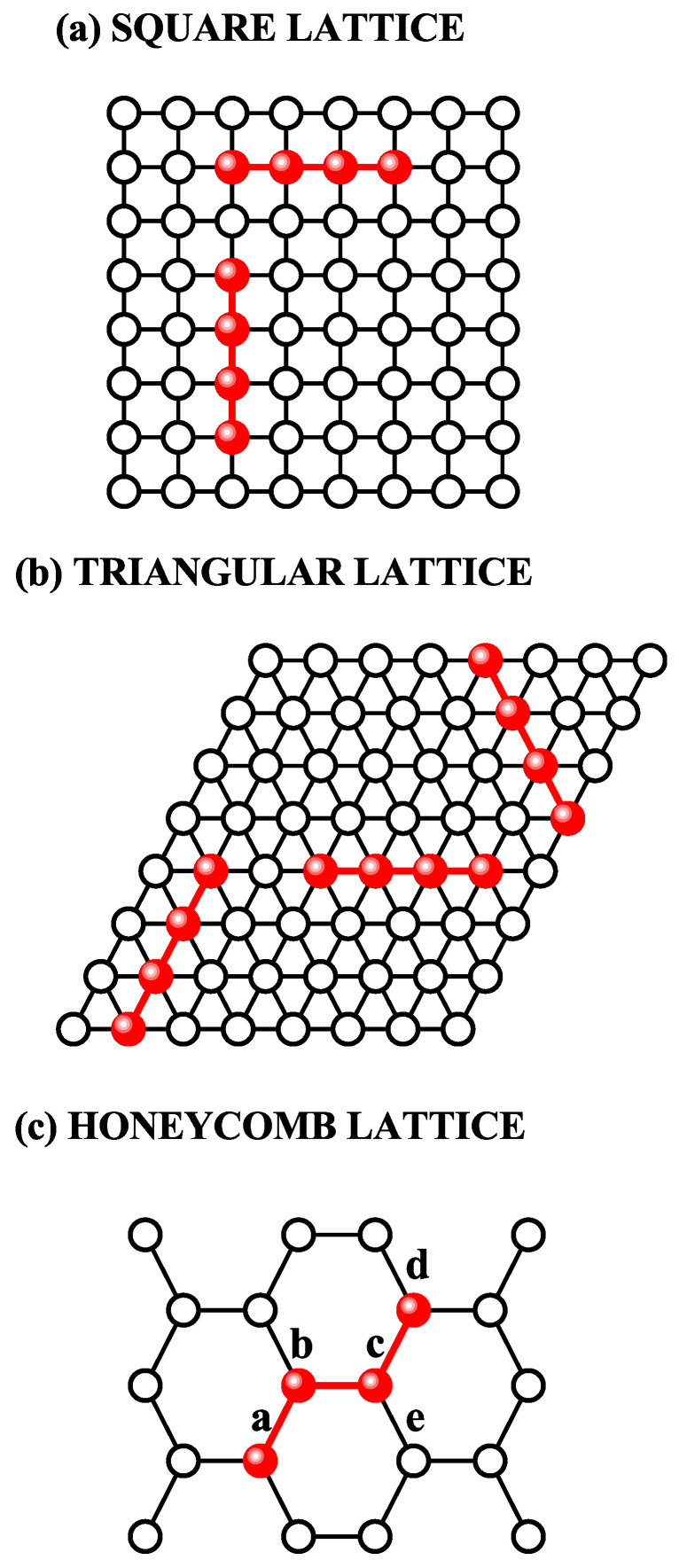
Linear tetramers adsorbed onto (**a**) square, (**b**) triangular, and (**c**) honeycomb lattices. Full and empty circles represent tetramer units and empty sites, respectively.

For square and triangular lattices (see Figure 4a,b), defining a linear *k*-mer is straightforward: it refers to a sequence of *k* monomeric units aligned along a straight path on the lattice. However, in the case of the honeycomb lattice, its intrinsic geometry prevents the formation of such strictly linear chains. To address this issue, we redefine a “linear *k*-mer” for the honeycomb structure as a connected sequence of *k* monomers placed according to the following rule: after placing the first monomer, the second occupies one of its three nearest-neighbor sites. The third monomer is positioned on one of the two adjacent sites to the second monomer, and each subsequent monomer (i≥4) is added to a neighboring site of the previous monomer in such a way that the distance from the first monomer is maximized. This approach ensures that all *k* monomers are placed without overlap. An example of this is shown in Figure 4c, where a linear tetramer is adsorbed onto a honeycomb lattice. After the first three monomers are placed at positions labeled a, b, and c, the fourth monomer can occupy either of the two available sites labeled d or e.

In general, the total number of accessible states for placing *N k*-mers on a lattice with *M* sites, denoted as Ω(M,N,γ), is dependent on the lattice’s connectivity. A practical approximation for Ω(M,N,γ) assumes a random spatial distribution of the molecules and utilizes theoretical arguments from several studies [6,8,33,34,80] to relate the configurational term for arbitrary γ to its exact expression in one dimension (i.e., for γ=2). Accordingly,

(31) $$\Omega \left(M,N,\gamma \right)=K{\left(\gamma ,k\right)}^{N}\Omega \left(M,N,2\right),$$

where Ω(M,N,2) can be exactly evaluated by counting the number of ways to arrange *N* indistinguishable *k*-mers among $n_{e}$ available elements, where $n_{e}$ is given by

(32) $$\begin{array}{ccc} n_{e} & = & \mathrm{number}\;\mathrm{of}\;k-\mathrm{mers}\;+\;\mathrm{number}\;\mathrm{of}\;\mathrm{empty}\;\mathrm{sites}\\ & = & N+M-kN=M-\left(k-1\right)N. \end{array}$$

Accordingly,

(33) $$\Omega \left(M,N,2\right)=\left(\begin{array}{c} n_{e}\\ N \end{array}\right)=\frac{\left[M-\left(k-1\right)N\right]!}{N!\left[M-kN\right]!}$$

(a particular solution for dimers has been presented in [96]).

On the other hand, the function K(γ,k) denotes the number of possible configurations per site for placing a *k*-mer at zero coverage. This function depends on both the connectivity of the lattice and the geometric characteristics of the adsorbing molecule. It can be demonstrated that

(34) $$K\left(\gamma ,k\right)=\left\{\begin{array}{cc} \gamma /2 & \;\;\;\mathrm{for}\;\;\mathrm{linear}\;\;k\text{-mers}\\ \left[\gamma {\left(\gamma -1\right)}^{\left(k-2\right)}\right]/2-m^{\prime } & \;\;\;\mathrm{for}\;\;\mathrm{flexible}\;\;k\text{-mers} \end{array}\right.,$$

where $m^{\prime }$ denotes the subset of those configurations that result in self-overlapping, which must be excluded. This correction ensures that K(γ,k) accurately reflects the number of valid placements. Notably, for monomer adsorption (k=1), we recover K(γ,1)=1, and Equation (34) is only applicable for k≥2.

In the canonical ensemble, the Helmholtz free energy F(M,N,T) relates to Ω(M,N,γ) as follows:

(35) $$\beta F\left(M,N,T\right)=-\ln Q\left(M,N,T\right)=-\ln\Omega \left(M,N,\gamma \right)+\beta k\epsilon_{0}N,$$

where $\beta =1/k_{B}T$.

The remaining thermodynamic functions can be obtained from the general differential form [80]

(36) dF=−SdT−ΠdM+μdN,

where *S*, Π, and μ designate the entropy, spreading pressure, and chemical potential, respectively which, by definition, are given as follows:

(37) $$S=-{\left(\frac{\partial F}{\partial T}\right)}_{M,N}\;\;\mu ={\left(\frac{\partial F}{\partial N}\right)}_{T,M}.$$

Thus, from Equations (33) and (35),

(38) $$\beta F\left(M,N,T\right)=-\left\{N\ln K\left(\gamma ,k\right)+\ln\left[M-\left(k-1\right)N\right]!-\ln N!-\ln\left[M-kN\right]!\right\}+\beta k\epsilon_{0}N,$$

which can be accurately written in terms of the Stirling’s approximation

(39) $$\begin{array}{ccc} \beta F(M,N,T) & = & -\left[M-\left(k-1\right)N\right]\ln\left[M-\left(k-1\right)N\right]+\left[M-\left(k-1\right)N\right]\\ & & +\left[N\ln N-N\right]+\left[\left(M-kN\right)\ln\left(M-kN\right)-\left(M-kN\right)\right]\\ & & +\beta k\epsilon_{0}N-N\ln K\left(\gamma ,k\right)\\ & = & -\left[M-\left(k-1\right)N\right]\ln\left[M-\left(k-1\right)N\right]+N\ln N\\ & & +\left(M-kN\right)\ln\left(M-kN\right)+\beta k\epsilon_{0}N-N\ln K\left(\gamma ,k\right). \end{array}$$

Henceforth, from Equations (37) and (39)

(40) $$\frac{S(M,N)}{k_{B}}=\left[M-\left(k-1\right)N\right]\ln\left[M-\left(k-1\right)N\right]-N\ln N-\left(M-kN\right)\ln\left(M-kN\right)+N\ln K\left(\gamma ,k\right),$$

and

(41) $$\beta \left(\mu -k\epsilon_{0}\right)=\ln\frac{kN}{M}+\left(k-1\right)\ln\left[1-\left(k-1\right)\frac{N}{M}\right]-k\ln\left[1-\frac{kN}{M}\right]-\ln K\left(\gamma ,k\right).$$

Introducing intensive variables such as the lattice coverage θ=kN/M, the molar free energy f=F/M, and the molar entropy s=S/M, Equations (40) and (41) can be reformulated in terms of these normalized quantities as follows: θ and *T*,

(42) $$\frac{s(\theta ,\gamma )}{k_{B}}=\left[1-\frac{\left(k-1\right)}{k}\theta \right]\ln\left[1-\frac{\left(k-1\right)}{k}\theta \right]-\frac{\theta }{k}\ln\frac{\theta }{k}-\left(1-\theta \right)\ln\left(1-\theta \right)+\frac{\theta }{k}\ln K(\gamma ,k),$$

and

(43) $$kK(\gamma ,k)\exp\left[\beta \left(\mu -k\epsilon_{0}\right)\right]=\frac{\theta {\left[1-\frac{\left(k-1\right)}{k}\theta \right]}^{k-1}}{{\left(1-\theta \right)}^{k}}.$$

Equations (34)–(43) thus offer the foundational thermodynamic relationships for systems of non-interacting polyatomic species adsorbed onto lattices with general coordination number γ.

#### 3.1.2. Fractional Statistics Thermodynamic Theory of Adsorption of Polyatomics (FSTA)

We introduce the foundation of a phenomenological thermodynamic theory for the adsorption of polyatomic species, drawing upon a novel conceptual approach inspired by the formalism of Haldane’s fractional statistics [14,15], which generalizes the Pauli Exclusion Principle. For clarity and simplicity, our formulation focuses on adsorption processes within a uniform adsorption field. This framework—which we refer to as Fractional Statistics Thermodynamic Adsorption (FSTA)—begins with the idea that the interaction between a single molecule and a solid surface can be modeled through an adsorption potential field (this field is typically represented as a lattice of adsorption sites; however, for molecules or particles composed of multiple subunits, the correspondence between a physical equilibrium state and a single lattice site becomes more complex, as will be explained later), which contains a total of *G* local minima across the coordinate space relevant to the adsorption configuration. One can interpret *G* as the number of distinct equilibrium states accessible to an individual adsorbed molecule.

Due to the finite size and geometric configuration of the adsorbed particle, the presence of a molecule on the surface can render a subset of these *G* states unavailable for further adsorption. We quantify the average number of excluded states per adsorbed particle by introducing the parameter *g*, which reflects the nature of the so-called “statistical interactions” among adsorbing species, as will be discussed further on. This parameter *g* (or, more generally, a function *g*) is the central phenomenological element of our theory, possessing a clear physical interpretation for both lattice and continuum models. It can be extracted from experimental thermodynamic data and is directly linked to the spatial configuration of the adsorbed entity. Essentially, the configurational entropy of the system is encoded in *g*, allowing for a more intuitive and simplified description of the statistical and thermodynamic behavior of complex adsorbed polyatomic molecules. Furthermore, as the exclusion of states may result from the overlap effects caused by multiple adsorbed particles, the parameter *g* is typically a function of particle density; hence, we write g≡g(N).

Consider now a scenario where (N−1) indistinguishable particles are present within a system of fixed volume *V* that includes *G* available equilibrium states. The number of states still accessible to the *N*th particle upon its introduction into the system is given by [14]:

(44) $$d_{N}=G-\sum_{N^{\prime }=1}^{N-1}g\left(N^{\prime }\right)=G-G_{0}\left(N\right).$$

This expression essentially corresponds to the definition introduced by Haldane [14] as a generalization of the Pauli Exclusion Principle, which forms the foundation of Haldane’s fractional statistics—often referred to as quantum fractional statistics. Building on this idea, we propose that the class of physical systems considered here can be described by a generalized statistical framework characterized by a parameter g(N)≥1. Within this framework, the number of accessible configurations for a system composed of *N* molecules distributed over *G* available states is given by

(45) $$W\left(N\right)=\frac{\left(d_{N}+N-1\right)!}{\left[N!\left(d_{N}-1\right)!\right]}=\frac{\left[G-G_{0}\left(N\right)+N-1\right]!}{\left\{N!\left[G-G_{0}\left(N\right)-1\right]!\right\}}.$$

In the special case where each particle excludes exactly one state from further occupancy, g(N)=1 for all *N*, leading to $G_{0}\left(N\right)=N-1$, and the expression for W(N) simplifies to the standard combinatorial result for fermion-like behavior: W(N)=G!/[N!(G−N)!]. Conversely, if no exclusion occurs—meaning that g(N)=0 for all *N*—then we recover the bosonic case, where the number of configurations is given by $W\left(N\right)=\left(G+N-1\right)!/\left[N!\left(G-N\right)!\right]$.

More generally, the configurational entropy per site and the adsorption isotherm for a system of non-interacting adsorbed polyatomic species can be derived from Equation (45):

(46) $$\begin{array}{ccc} \frac{s(n,\gamma )}{k_{B}} & = & -\frac{n}{ak}\ln n+\frac{1}{ak}\left[1-{\tilde{G}}_{0}\left(n\right)+n\right]\ln\left[1-{\tilde{G}}_{0}\left(n\right)+n\right]\\ & & -\frac{1}{ak}\left[1-{\tilde{G}}_{0}\left(n\right)\right]\ln\left[1-{\tilde{G}}_{0}\left(n\right)\right], \end{array}$$

and

(47) $$\exp\left[\beta \left(\mu -k\epsilon_{0}\right)\right]=\frac{n{\left[1-{\tilde{G}}_{0}\left(n\right)+n\right]}^{\left({\tilde{G}}_{0}^{\prime }-1\right)}}{{\left[1-{\tilde{G}}_{0}\left(n\right)\right]}^{{\tilde{G}}_{0}^{\prime }}},$$

where the density n=N/G remains finite as N,G→∞ and is proportional to the surface coverage θ, such that n=aθ. The coverage θ represents either the fraction $N/N_{m}$ or $v/v_{m}$, where *N* (or *v*) is the number of adsorbed molecules (or amount) at a given chemical potential μ and temperature *T*, and $N_{m}$ (or $v_{m}$) corresponds to monolayer coverage. The terms ${\tilde{G}}_{0}\left(n\right)\equiv \lim_{N,G\to \infty }G_{0}\left(N\right)/G$ and ${\tilde{G}}_{0}^{\prime }\equiv d{\tilde{G}}_{0}/dn$ describe the normalized and derivative exclusion functions, respectively.

In the following, we consider a simplified—but often accurate—scenario in the FSTA formalism, where the exclusion parameter *g* is taken as a constant. It should be noted that, as the surface coverage increases, adsorbed molecules may change configuration, making *g* density-dependent in general; therefore, experimental values of *g* can vary with the pressure range studied, as seen in Equation (47). The parameter *a* is linked to the low-density limit through the relation θ→0, βμ≈lnaθ. Under the assumptions ${\tilde{G}}_{0}=gn$ and ${\tilde{G}}_{0}^{\prime }=g$, Equation (47) leads to a specific adsorption isotherm:

(48) $$\exp\left[\beta \left(\mu -k\epsilon_{0}\right)\right]=\frac{a\theta {\left[1-a\theta \left(g-1\right)\right]}^{g-1}}{{\left[1-a\theta g\right]}^{g}}.$$

Equation (48) includes well-established isotherms as limiting cases. For instance, when particles occupy a single adsorption site (such as spherical molecules), they effectively exclude only one state, corresponding to g=1. In this limit, Equation (48) simplifies to the classical Langmuir isotherm [80]. On the other hand, if the adsorbing molecules are linear chains lying flat along a one-dimensional substrate and composed of *k* identical units, then g=k, and Equation (48) recovers the exact isotherm for this configuration. This illustrates the fundamental link between the exclusion parameter *g* and the geometric characteristics of the adsorbing molecules.

Finally, we briefly outline several representative adsorption configurations addressed within this formalism. Consider polyatomic adsorbates composed of *k* monomeric units, of which $k^{\prime }$ are in direct contact with the surface, while the remaining $\left(k-k^{\prime }\right)$ units extend outward or are oriented away. On a lattice including *M* sites, the surface coverage is given by $\theta =k^{\prime }N/M$. For example: (1) If m=1, g=1, and a=1, the scenario corresponds to end-on adsorption, where only one segment of each *k*-mer is in contact with the surface. (2) If m=1, $g=k^{\prime }$, and $a=1/k^{\prime }$, the model describes chains with $k^{\prime }$ units attached to the substrate and the rest detached. (3) For fully adsorbed flat configurations where all *k* units occupy distinct sites, $k^{\prime }=k$ and the total number of accessible adsorption states is G=Mm, with *m* denoting the number of distinguishable configurations per lattice site. Then, 1/a=km.

As a specific case, rigid linear *k*-mers adsorbed flat on a two-dimensional lattice with connectivity γ have m=γ/2, which gives g=kγ/2 and a=2/(γk). Inserting these values into Equation (48) yields the adsorption isotherm for this configuration:

(49) $$\exp\left[\beta \left(\mu -k\epsilon_{0}\right)\right]=\frac{\frac{2\theta }{k\gamma }{\left[1-\theta \frac{\left(k\gamma -2\right)}{k\gamma }\right]}^{\left(\frac{k\gamma }{2}-1\right)}}{{\left[1-\theta \right]}^{\frac{k\gamma }{2}}}.$$

#### 3.1.3. Occupation Balance Approximation (OB)

It is well-known that the mean number of particles in the adlayer $\bar{N}$ and the chemical potential μ are related through the following general relationship in the grand canonical ensemble

(50) $$\bar{N}=\lambda {\left[\frac{\partial \ln\Xi (M,\lambda )}{\partial \lambda }\right]}_{M},$$

where λ=exp(βμ) and Ξ is the grand partition function. Solving $\lambda^{-1}$ from Equation (50), we have

(51) $$\lambda^{-1}=\frac{1}{\bar{N}}{\left[\frac{\partial \ln\Xi (M,\lambda )}{\partial \lambda }\right]}_{M}=\frac{\bar{R}\left(M,\lambda \right)}{\bar{N}},$$

where the quantity $\bar{R}\left(M,\lambda \right)$ can be proven to be the mean number of states available to a particle on *M* sites at λ. If $Y_{t}\left(M,N\right)$ and $R_{i}\left(M,N\right)$ denote the total number of configurations of *N* distinguishable particles on *M* sites and the number of states available to the (N+1)th particle in the *i*th configuration [out of $Y_{t}\left(M,N\right)$], respectively, then

(52) $$Y_{t}\left(M,N+1\right)=\sum_{i=1}^{Y_{t}\left(M,N\right)}R_{i}\left(M,N\right).$$

The total number of configurations of (N+1) indistinguishable particles on *M* sites, $G_{t}\left(M,N+1\right)$, can be obtained from Equation (52) as

(53) $$\begin{array}{ccc} G_{t}\left(M,N+1\right) & = & \frac{Y_{t}\left(M,N+1\right)}{(N+1)!}=\frac{\sum_{i=1}^{Y_{t}\left(M,N\right)}R_{i}\left(M,N\right)}{(N+1)!}\\ & = & \frac{N!}{(N+1)!}\sum_{i=1}^{G_{t}\left(M,N\right)}R_{i}\left(M,N\right)=\frac{1}{N+1}\sum_{i=1}^{G_{t}\left(M,N\right)}R_{i}\left(M,N\right). \end{array}$$

In the previous arguments we consider that, for each configuration of *N* indistinguishable particles, there exist N! configurations of *N* distinguishable particles.

The average of $R_{i}\left(M,N\right)$ over a grand canonical ensemble is

(54) $$\begin{array}{ccc} \bar{R}\left(M,\lambda \right)=\langle R_{i}\left(M,N\right)\rangle & = & \frac{1}{\Xi }\sum_{N=0}^{N_{m}}\left\{\lambda^{N}\sum_{i=1}^{G_{t}}R_{i}\left(M,N\right)\right\}\\ & = & \frac{1}{\Xi }\sum_{N=0}^{N_{m}-1}\left(N+1\right)\lambda^{N}G_{t}\left(M,N+1\right)\\ & = & \frac{\lambda^{-1}}{\Xi }\sum_{N^{\prime }=1}^{N_{m}}\lambda^{N^{\prime }}N^{\prime }G_{t}\left(M,N^{\prime }\right)=\frac{\bar{N}}{\lambda }, \end{array}$$

as already proposed in Equation (51), where $N^{\prime }=N+1$, $N_{m}$ is the maximum number of particles that fit in the lattice, and $R_{i}\left(M,N_{m}\right)=0$.

The advantage of using Equation (51) to calculate the coverage dependence of the fugacity λ can be seen when dealing with the adsorption of dimers in the monolayer regime. $\bar{R}\left[M,\lambda \left(\bar{N}\right)\right]=\bar{R}\left(M,\bar{N}\right)$ for dimers (occupying two nearest-neighbor lattice sites) is, at first order, $\bar{R}\left(M,\bar{N}\right)\approx \gamma M/2-\left(2\gamma -1\right)\bar{N}$ (note that, if it is assumed that each dimer is independent from the neighboring ones, each dimer excludes (2γ−1) states out of a total of γM/2), where the second terms account for the mean number of states excluded by the adsorbed dimers on a lattice with connectivity γ. Thus,

(55) $$\lim_{M\to \infty }\lambda^{-1}\approx \lim_{M\to \infty }\frac{\gamma M/2-\left(2\gamma -1\right)\bar{N}}{\bar{N}}=\frac{\gamma }{\theta }-\left(2\gamma -1\right),$$

where $\lim_{M\to \infty }2\bar{N}/M=\theta$.

The term (2γ−1) overestimates the number of excluded states due to the simultaneous exclusion of neighboring particles. Then, the approximation can be further refined by considering the mean number of states that are simultaneously excluded by $\bar{N}$ dimers, $\bar{L}\left(M,\bar{N}\right)$. It is possible to demonstrate that, in general, $\bar{R}\left(M,\bar{N}\right)=\gamma M/2-\left(2\gamma -1\right)\bar{N}+\bar{L}\left(M,\bar{N}\right)$ for linear *k*-mers.

For dimers, $\bar{L}\left(M,\bar{N}\right)$ is the average number of occupied nearest-neighbor sites. As it is not possible to obtain exact solutions for $\bar{L}\left(M,\bar{N}\right)$, we use the approximation

(56) $$\bar{L}\left(M,\bar{N}\right)\approx \frac{\bar{N}\left(\bar{N}-1\right)}{2}\bar{L}\left(M,2\right),$$

where $\bar{N}\left(\bar{N}-1\right)/2$ is the number of possible pairs for $\bar{N}$ indistinguishable particles.

Considering a system of two adsorbed dimers on a square lattice (γ=4), we can write

(57) $$\bar{L}\left(M,2\right)=\frac{g_{1}\left(M,2\right)+2g_{2}\left(M,2\right)}{G_{t}\left(M,2\right)}=\frac{18}{\left(2M-7\right)},$$

where $G_{t}\left(M,2\right)=M\left(2M-7\right)$. In addition, $g_{1}\left(M,2\right)=14M$ and $g_{2}\left(M,2\right)=2M$ are the number of states with one and two occupied nearest-neighbors, respectively. Finally, we can write

(58) $$\begin{array}{ccc} \lim_{M\to \infty }\lambda^{-1} & = & \lim_{M\to \infty }\frac{2M-7\bar{N}+\bar{L}\left(M,\bar{N}\right)}{\bar{N}}\\ & \approx & \lim_{M\to \infty }\frac{1}{\bar{N}}\left[2M-7\bar{N}+\frac{9\bar{N}\left(\bar{N}-1\right)}{\left(2M+7\right)}\right]\\ & \approx & \frac{4}{\theta }-7+\frac{9}{4}\theta +O\left(\theta^{2}\right). \end{array}$$

Finally, considering that the terms neglected in Equation (58) are $O(\theta^{2})$, it becomes

(59) $$\lambda^{-1}=\frac{4}{\theta }-7+\frac{9}{4}\theta +a\theta^{2}\;\;\;\;\;\;\;\;\;\;\;\left(\mathrm{square}\;\mathrm{lattice}\right),$$

and the constant a=3/4 can be determined from the limiting condition λ→∞ for θ→1. Similarly,

(60) $$\lambda^{-1}=\frac{3}{\theta }-5+\frac{4}{3}\theta +\frac{2}{3}\theta^{2}\;\;\;\;\;\;\;\;\;\;\;\left(\mathrm{honeycomb}\;\mathrm{lattice}\right),$$

and

(61) $$\lambda^{-1}=\frac{6}{\theta }-11+\frac{23}{6}\theta +\frac{7}{6}\theta^{2}\;\;\;\;\;\;\;\;\;\;\;\left(\mathrm{triangular}\;\mathrm{lattice}\right).$$

The entropy per lattice site can be evaluated in the limit T→∞ as follows

(62) $$\frac{\mu }{k_{B}T}=\ln\lambda =-\frac{1}{k_{B}}\lim_{M,T\to \infty }{\left[\frac{\partial S(M,N,T)}{\partial N}\right]}_{M,T}=-\frac{2}{k_{B}}\left[\frac{ds(\theta )}{d\theta }\right].$$

Then,

(63) $$\frac{s(\theta )}{k_{B}}=-\frac{1}{2}\int_{0}^{\theta }\ln\lambda \left(\theta^{\prime }\right)\;\;d\theta^{\prime }.$$

From Equations (59)–(61) and (63), we obtain

(64) $$\begin{array}{ccc} \frac{s(\theta )}{k_{B}} & = & \frac{\theta }{2}\left[\ln C-\ln\theta -2\right]-\frac{\left(1-\theta \right)}{2}\ln\left(1-\theta \right)-\frac{\left(A-\theta \right)}{2}\ln\left(A-\theta \right)\\ & + & \frac{\left(B+\theta \right)}{2}\ln\left(B+\theta \right)+\frac{A}{2}\ln A-\frac{B}{2}\ln B, \end{array}$$

where $A=2\left(\sqrt{7/3}-1\right),\left(3/2\right)\left(\sqrt{3}-1\right)$, and $\left(15/7\right)\left(\sqrt{53}/5-1\right)$; C=3/4,2/3, and 7/6; and $B=2\left(\sqrt{7/3}+1\right),\left(3/2\right)\left(\sqrt{3}+1\right)$, and $\left(15/7\right)\left(\sqrt{53}/5+1\right)$ for square, honeycomb, and triangular lattices, respectively.

#### 3.1.4. Semi-Empirical Adsorption Model for Polyatomics (SE)

In this section, we present a semi-empirical approximation for the adsorption isotherm of non-interacting *k*-mers on a regular lattice, which yields highly accurate predictions.

Our starting point is Equation (51), representing the Occupation Balance formalism. To approximate the term *R*, we adopt a modified version of the method originally developed by Flory for the derivation of $\Omega (N_{1},N_{2})$ in terms of the placement probabilities $w_{i}$. Accordingly, R(M,λ) can be expressed as:

(65) $$R=\left(\frac{\gamma }{2}M\right)\prod_{i=1}^{k}P_{i}.$$

The interpretation of Equation (65) is straightforward: the term in parentheses accounts for the total number of possible linear arrangements of *k* consecutive sites on the lattice. These configurations can be classified into three categories: fully occupied *k*-tuples (associated with adsorbed *k*-mers), completely unoccupied *k*-tuples (available for adsorption), and frustrated *k*-tuples (those partially filled or interrupted by segments from different *k*-mers). To evaluate the contribution of the empty *k*-tuples, we introduce a multiplicative factor representing the probability that a given *k*-tuple is vacant. This factor is modeled as a product of *k* terms, denoted $P_{i}$, where each $P_{i}$ is the conditional probability that the *i*-th site in the tuple is empty, assuming the previous i−1 sites are also unoccupied. For the initial case i=1, we obtain:

(66) $$P_{1}=1-\theta ,$$

which corresponds to an exact expression.

To simplify further, we consider the most basic approximation, assuming $P_{i}=P_{1}$ for all *i*. Under this assumption, and using Equations (51)–(66), one recovers the well-known Flory–Huggins (FH) isotherm for non-interacting, linear *k*-mers adsorbing flat onto a uniform surface:

(67) $$\lambda^{-1}=\frac{R}{\bar{N}}=\frac{\gamma k}{2}\frac{M}{k\bar{N}}{P_{1}}^{k}=\frac{\gamma k{\left(1-\theta \right)}^{k}}{2\theta }.$$

This example illustrates the flexibility of the proposed formalism, which can accommodate a variety of multisite adsorption scenarios.

More generally, each $P_{i}$ can be expressed as:

(68) $$P_{i}=\left(1-\theta \right)C_{i},$$

where $C_{i}$ is a correction term, with $C_{1}=1$ by definition and $C_{i}\to 1$ in the low-coverage limit (θ→0). Inserting these into Equations (65)–(68), we derive:

(69) $$R=\frac{\gamma }{2}M{\left(1-\theta \right)}^{k}\prod_{i=2}^{k}C_{i}=\frac{\gamma }{2}M{\left(1-\theta \right)}^{k}{\tilde{C}}^{k-1},$$

and

(70) $$\tilde{C}={\left(\prod_{i=2}^{k}C_{i}\right)}^{\frac{1}{k-1}},$$

where $\tilde{C}$ is the geometric mean of the individual correction terms $C_{i}$. Combining this with Equations (51) and (69), we arrive at a generalized form of the adsorption isotherm for linear *k*-mers:

(71) $$\lambda^{-1}=\frac{\gamma k{\left(1-\theta \right)}^{k}{\tilde{C}}^{k-1}}{2\theta },$$

or

(72) $$\beta \left(\mu -k\epsilon_{0}\right)=\ln\left(\frac{\theta }{k}\right)-k\ln\left(1-\theta \right)-\ln\left(\frac{\gamma }{2}\right)-\left(k-1\right)\ln\tilde{C}.$$

It is insightful to compare this expression emerging from the Occupation Balance (OB) framework with those derived from other major theories addressing linear polyatomic adsorption. For this purpose, Equations (43) and (49) are recast in terms of a unified structure,

(73) $$\beta \left(\mu -k\epsilon_{0}\right)=\ln\left(\frac{\theta }{k}\right)-k\ln\left(1-\theta \right)-\ln\left(\frac{\gamma }{2}\right)+\left(k-1\right)\ln\left[1-\frac{(k-1)\theta }{k}\right]\;\;\;\;\;\;\;\;\;\;\;EA,$$

and

(74) $$\beta \left(\mu -k\epsilon_{0}\right)=\ln\left(\frac{\theta }{k}\right)-\frac{k\gamma }{2}\ln\left(1-\theta \right)-\ln\left(\frac{\gamma }{2}\right)+\left(\frac{k\gamma }{2}-1\right)\ln\left[1-\theta \frac{\left(k\gamma -2\right)}{k\gamma }\right]\;\;\;FSTA.$$

The corresponding expressions within the Flory–Huggins (FH) and Guggenheim–DiMarzio (GD) approximations are as follows [6,7,9,10,11,16]:

(75) $$\beta \left(\mu -k\epsilon_{0}\right)=\ln\left(\frac{\theta }{k}\right)-k\ln\left(1-\theta \right)-\ln\left(\frac{\gamma }{2}\right)\;\;\;\;\;\;\;\;\;\;\;\;\;\;\;\;\;\;\;\;\;\;\;\;\;\;FH\;\;\;\left(k\geq 2\right),$$

and

(76) $$\beta \left(\mu -k\epsilon_{0}\right)=\ln\left(\frac{\theta }{k}\right)-k\ln\left(1-\theta \right)-\ln\left(\frac{\gamma }{2}\right)+\left(k-1\right)\ln\left[1-\frac{\left(k-1\right)}{k}\frac{2\theta }{\gamma }\right]\;\;\;\;\;\;\;\;\;GD.$$

Notably, the EA, FH, and GD models inherently conform to the structure of Equation (72). The Fractional Statistical Theory of Adsorption (FSTA)—even in its simplest form for linear *k*-mers—can also be transformed into this structure with straightforward algebra.

From this reformulation, it becomes evident that the key differences among these models lie in how each theory estimates the average correction factor $\tilde{C}$. As a concrete example, both EA and GD produce the exact result for one-dimensional systems. Thus, comparing Equation (72) with the EA isotherm (or GD with γ=2) yields:

(77) $${\tilde{C}}^{-1}=1-\frac{k-1}{k}\theta \;\;\;\;\;\;\;\;\;\;\;\;\;\;\;\;\;\;\left(\gamma =2\right).$$

This relationship has been rigorously demonstrated in the literature [9].

Moreover, earlier comparative studies [11] involving simulations on two-dimensional lattices showed that the GD model aligns well with numerical data at low surface coverage, while EA achieves better agreement at high coverage levels. When cast into the forms given by Equations (73) and (76), it becomes apparent that the only distinction between EA and GD lies in the behavior of the correction function $\tilde{C}$. Based on these insights and the structure provided by Equation (72), we propose a new semi-empirical isotherm for polyatomic adsorption (denoted SE):

(78) $$\begin{array}{ccc} \beta \left(\mu -k\epsilon_{0}\right) & = & \ln\left(\frac{\theta }{k}\right)-k\ln\left(1-\theta \right)-\ln\left(\frac{\gamma }{2}\right)\\ & & +\left(1-\theta \right)\left(k-1\right)\ln\left[1-\frac{\left(k-1\right)}{k}\frac{2\theta }{\gamma }\right]\\ & & +\theta \left(k-1\right)\ln\left[1-\frac{(k-1)\theta }{k}\right]. \end{array}$$

The first line retains the common contributions shared by both the EA and GD models. The following lines introduce a weighted interpolation between the GD and EA correction functions, modulated by the surface coverage θ. This blending provides a smooth transition between the two regimes, ensuring accurate predictions across the full range of coverage.

#### 3.1.5. Brief Introduction to Multiple Exclusion Statistics

In Section 5 and Section 6, we review a comprehensive statistical framework to describe the thermodynamics of classical lattice gases formed by rigid particles with arbitrary size and shape, focusing particularly on the behavior of linear *k*-mers on a square lattice. This framework extends the recently proposed multiple exclusion (ME) statistics [17] to multicomponent systems, capturing the complex spatial correlations inherent to structured particle configurations. A generalized density of states formalism is introduced, parameterized by state exclusion correlation parameters that account for both self-exclusion and cross-exclusion effects between different species. First, the ME statistical mechanics for a single particle species [18] are reviewed, with a rigorous derivation of the generalized entropy, Helmholtz free energy, and chemical potential functions. The theory is then applied to isolated species of *k*-mers on the square lattice, rationalizing the emergence of an entropy-driven isotropic–nematic transition for large enough *k*, with no transition observed for k≤6. Analytical expressions for thermodynamic potentials are obtained as functions of the mean lattice occupation, revealing the critical role played by state exclusion multiplicity and the density dependence of the available states. The thermodynamic quantities derived from this formalism show remarkable agreement with Monte Carlo (MC) simulation results across all density regimes, validating the ME statistics approach.

Building on this foundation, a more refined formulation of the ME statistics to mixtures of different species presented in Ref. [19] is also presented. This provides a more robust approach to lattice gases with various axes of symmetry, such that the effect of state cross-exclusion between particles differently oriented can be finely quantified.

Particularly, for the *k*-mer problem in a square lattice, two particle orientations apply: modeling horizontal and vertical *k*-mers as two distinct but inter-related species. This formalism embodies cross-exclusion effects explicitly and introduces a generalized density of states which is capable of accounting for spatial correlations between different species. Analytical solutions for the Helmholtz free energy surface $\beta f(n_{1},n_{2})$ are derived, providing access to equilibrium occupation paths, phase coexistence regions, and order parameter behaviors. Importantly, the theory predicts two distinct phase transitions for large *k* (k≥7): (i) a continuous isotropic-to-nematic transition at intermediate coverage, driven by the entropy gain associated with increasing orientational order increasing the multiple state exclusion as compared to isotropic configurations with excessively large state exclusion but less efficient multiple exclusion; and (ii) a first-order nematic-to-isotropic transition at high coverage, associated with the breakdown of nematic order due to geometric constraints near lattice saturation due to an isotropic configuration having higher entropy at saturation than the vanishing one for a full aligned nematic phase. This system is addressed in detail, along with other applications of the analytical models treated in this review.

The model also accurately predicts critical densities and chemical potentials for both transitions, being in close agreement with existing MC simulation data and shedding light on the order of transitions; this is particularly important considering the still controversial nature of the high-coverage nematic–isotropic transition, which has been identified as weakly first-order—consistent with recent MC observations.

A novel aspect of this work is the introduction of the state exclusion frequency functions $e_{ij}\left(\theta \right)$ and the cumulative exclusion spectrum functions $G_{ij}\left(\theta \right)$, which offer a thermodynamic characterization of phase transitions in terms of the coverage dependence of state exclusion.

The ME statistics in general, and the mixture formulations in particular, provide a unified, self-consistent, and predictive formalism for lattice gases of structured particles. This framework not only explains the nematic ordering transitions observed in *k*-mer systems, but also lays the groundwork for studying more complex systems such as rods on triangular or cubic lattices, particles with additional axes of symmetry, or mixtures of particles with different shapes and sizes. Future work should explore these generalizations, aiming to connect exclusion statistics formulations with broader classes of phase transitions in the context of soft condensed matter and statistical physics.

### 3.2. Two-Dimensional Model of Non-Interacting (k-mer–l-mer) Binary Mixtures

In this section, we examine the adsorption behavior of a binary mixture consisting of linear rigid molecules—specifically, *k*-mers and *l*-mers—on two-dimensional lattice surfaces. Each *k*-mer (or *l*-mer) is modeled as a chain of *k* (or *l*) identical units aligned linearly, with a fixed inter-unit spacing equal to the lattice constant *a*. Without loss of generality, we consider that l<k throughout this analysis.

The surface is idealized as a two-dimensional grid composed of *M* adsorption sites (with M→∞), characterized by a coordination number γ and subjected to periodic boundary conditions. This setup ensures the equivalence of all lattice sites, thereby eliminating edge effects from the thermodynamic analysis.

Adsorption occurs in such a way that each *k*-mer or *l*-mer lies flat on the surface and occupies *k* or *l* adjacent sites, respectively. Overlapping between molecules is prohibited, which ensures that the model reflects the monolayer adsorption limit.

As no lateral interactions are assumed between adsorbed species, all possible spatial arrangements of $N_{k}$ *k*-mers and $N_{l}$ *l*-mers across the *M* lattice sites are considered to have equal statistical weight. Therefore, the canonical partition function, $Q(M,N_{k},N_{l},T)$, is given by the product of the total number of allowed configurations, $\Omega (M,N_{k},N_{l})$, and a Boltzmann factor that accounts for the combined interaction energy of all adsorbed particles with the surface, $E(N_{k},N_{l})$:

(79) $$Q\left(M,N_{k},N_{l},T\right)=\Omega \left(M,N_{k},N_{l}\right)\exp\left[-\frac{E(N_{k},N_{l})}{k_{B}T}\right].$$

On the other hand, $E(N_{k},N_{l})$ can be written as

(80) $$E\left(N_{k},N_{l}\right)=\epsilon_{k}N_{k}+\epsilon_{l}N_{l},$$

where $\epsilon_{i}$ represents the adsorption energy of an *i*-mer (i=k,l).

In order to calculate $\Omega (M,N_{k},N_{l})$, different theories can be used [97,98]. Some examples are presented in the following sections.

#### 3.2.1. EA Approximation

As previously discussed for single species [6,8,33,34,80], the number of configurations of $N_{k}$ *k*-mers and $N_{l}$ *l*-mers on *M* sites, $\Omega (M,N_{k},N_{l},\gamma )$, depends on the lattice connectivity γ, and can be written in terms of the same quantity in one dimension (γ=2). Thus,

(81) $$\Omega \left(M,N_{k},N_{l},\gamma \right)=K_{k}{\left(\gamma ,k\right)}^{N_{k}}K_{l}{\left(\gamma ,l\right)}^{N_{l}}\Omega \left(M,N_{k},N_{l},2\right),$$

where $K_{i}\left(\gamma ,i\right)$ (i=k,l) can be obtained from Equation (34), and $\Omega (M,N_{k},N_{l},2)$ can be readily calculated as the total number of permutations of the $N_{k}$ indistinguishable *k*-mers and $N_{l}$ indistinguishable *l*-mers out of $n_{e}$ entities, with $n_{e}$ defined as

(82) $$\begin{array}{ccc} n_{e} & = & \mathrm{number}\;\mathrm{of}\;k-\mathrm{mers}\;+\;\mathrm{number}\;\mathrm{of}\;l-\mathrm{mers}\;+\;\mathrm{number}\;\mathrm{of}\;\mathrm{empty}\;\mathrm{sites}\\ & = & N_{k}+N_{l}+M-kN_{k}-lN_{l}=M-\left(k-1\right)N_{k}-\left(l-1\right)N_{l}. \end{array}$$

Accordingly,

(83) $$\Omega \left(M,N_{k},N_{k},2\right)=\frac{\left[M-\left(k-1\right)N_{k}-\left(l-1\right)N_{l}\right]!}{N_{k}!N_{l}!\left[M-kN_{k}-lN_{l}\right]!}.$$

Then, introducing Equation (83) into Equation (31),

(84) $$\Omega \left(M,N_{k},N_{l},\gamma \right)=K_{k}{\left(\gamma ,k\right)}^{N_{k}}K_{l}{\left(\gamma ,l\right)}^{N_{l}}\frac{\left[M-\left(k-1\right)N_{k}-\left(l-1\right)N_{l}\right]!}{N_{k}!N_{l}!\left[M-kN_{k}-lN_{l}\right]!}.$$

From Equation (35), we have that

(85) $$\begin{array}{ccc} \beta F(M,N_{k},N_{l},\gamma ,T) & = & -\ln Q(M,N_{k},N_{l},\gamma ,T)\\ & = & -\ln\Omega \left(M,N_{k},N_{l},\gamma \right)+\beta \epsilon_{k}N_{k}+\beta \epsilon_{l}N_{l}, \end{array}$$

with $\beta =1/k_{B}T$.

The chemical potential of the adsorbed species *i*, $\mu_{i,ads}$, can be calculated as [80]

(86) $$\mu_{i,ads}={\left(\frac{\partial F}{\partial N_{i}}\right)}_{{N_{j}}^{\prime }s}\;\;\;\;\;\;\;\;\;\left\{i,j=k,l\right\}.$$

From Equations (84)–(86), it follows that

(87) $$\begin{array}{ccc} \beta \left(\mu_{k,ads}-\epsilon_{k}\right) & = & \ln K_{k}\left(\gamma ,k\right)+\left(k-1\right)\ln\left[1-\left(\frac{k-1}{k}\right)\theta_{k}-\left(\frac{l-1}{l}\right)\theta_{l}\right]+\ln\frac{\theta_{k}}{k}\\ & & -k\ln\left(1-\theta_{k}-\theta_{l}\right), \end{array}$$

and

(88) $$\begin{array}{ccc} \beta \left(\mu_{l,ads}-\epsilon_{l}\right) & = & \ln K_{l}\left(\gamma ,l\right)+\left(l-1\right)\ln\left[1-\left(\frac{k-1}{k}\right)\theta_{k}-\left(\frac{l-1}{l}\right)\theta_{l}\right]+\ln\frac{\theta_{l}}{l}\\ & & -l\ln\left(1-\theta_{k}-\theta_{l}\right), \end{array}$$

where $\theta_{i}=iN_{i}/M$ represents the partial coverage of species *i*{i=k,l}.

At equilibrium, the chemical potential of the adsorbed and gas phases are equal. Then,

(89) $$\mu_{k,ads}=\mu_{k,gas},$$

and

(90) $$\mu_{l,ads}=\mu_{l,gas},$$

where $\mu_{k,gas}$ ($\mu_{l,gas}$) corresponds to *k*-mers (*l*-mers) in gas phase.

The chemical potential of each kind of molecule in an ideal gas mixture, at temperature *T* and pressure *P*, is

(91) $$\beta \mu_{k,gas}=\beta \mu_{k}^{0}+\ln X_{k}P,$$

and

(92) $$\beta \mu_{l,gas}=\beta \mu_{l}^{0}+\ln X_{l}P,$$

where $\mu_{k}^{0}$ and $\mu_{l}^{0}$ ($X_{k}$ and $X_{l}$) are the standard chemical potentials (mole fractions) of *k*-mers and *l*-mers, respectively. In addition,

(93) $$\beta \mu_{i}^{0}=-\ln\left[{\left(\frac{2\pi m_{i}k_{B}T}{h^{2}}\right)}^{3/2}k_{B}T\right]\;\;\;\;\;\;\;\;\;\left\{i=k,l\right\}.$$

Then, equating Equation (87) with Equation (91) and Equation (88) with Equation (92), the partial adsorption isotherms can be obtained as

(94) $$\begin{array}{ccc} & & \ln K_{k}\left(\gamma ,k\right)+\left(k-1\right)\ln\left[1-\left(\frac{k-1}{k}\right)\theta_{k}-\left(\frac{l-1}{l}\right)\theta_{l}\right]+\ln\frac{\theta_{k}}{k}\\ & - & k\ln\left(1-\theta_{k}-\theta_{l}\right)+\beta \Phi_{k}=0, \end{array}$$

and

(95) $$\begin{array}{ccc} & & \ln K_{l}\left(\gamma ,l\right)+\left(l-1\right)\ln\left[1-\left(\frac{k-1}{k}\right)\theta_{k}-\left(\frac{l-1}{l}\right)\theta_{l}\right]+\ln\frac{\theta_{l}}{l}\\ & - & l\ln\left(1-\theta_{k}-\theta_{l}\right)+\beta \Phi_{l}=0, \end{array}$$

where

(96) $$\beta \Phi_{i}\equiv \beta \epsilon_{i}-\beta \mu_{i}^{0}-\ln X_{i}P\;\;\;\;\;\;\;\;\;\left\{i=k,l\right\}.$$

#### 3.2.2. GD Approximation

In this section, the factor $\Omega (M,N_{k},N_{l})$ is obtained using the DiMarzio’s lattice theory [9]. We start by calculating the number of distinct ways to pack $N_{k}$ rigid rods onto a lattice with *d* allowed orientations (directions):

(97) $$\begin{array}{cc} \Omega_{k}\left(M,{\left\{N_{k}\right\}}_{d}\right)= & \frac{\prod_{i=1}^{d}\left[M-\left(k-1\right)N_{k,i}\right]!}{\left(M-k\;N_{k}\right)!{\left(M!\right)}^{d-1}\prod_{i=1}^{d}\left(N_{k,i}\right)!}, \end{array}$$

where $N_{k,i}$ is the number of *k*-mers lying in direction *i* and $N_{k}=\sum_{i=1}^{d}N_{k,i}$ is the total number of *k*-mers on the surface.

Now, using the DiMarzio counting scheme, the number of ways to place the $\left(j_{1}+1\right)$th *l*-type molecule onto the lattice (the subscript reminds us that we are discussing orientation 1)—given that $j_{1}$ *l*-type molecules have already been placed in direction 1 and $N_{k}$ *k*-type molecules have already been placed—is seen to be [99],

(98) $$\nu_{j_{1}+1}=\left(M-k\;N_{k}-l\;j_{1}\right){\left[\frac{M-k\;N_{k}-l\;j_{1}}{M-\left(k-1\right)N_{k,1}-\left(l-1\right)j_{1}}\right]}^{l-1}.$$

The total number of ways to place $N_{l,1}$ indistinguishable molecules onto the lattice in this orientation is

(99) $$\frac{\prod_{j_{1}=0}^{N_{1}-1}\nu_{j_{1}+1}}{\left(N_{l,1}\right)!}=\frac{\left(M-k\;N_{k}\right)!\left[M-\left(k-1\right)N_{k,1}-\left(l-1\right)N_{l,1}\right]!}{\left(M-k\;N_{k}-l\;N_{l,1}\right)!\left[M-\left(k-1\right)N_{k,1}\right]!N_{l,1}!}.$$

Similar expressions can be obtained for the other orientations and total numbers of ways to place $N_{l}$ hard rod molecules of type *l* when $N_{k}$ molecules of type *k* have been placed in the surface:

(100) $$\begin{array}{cc} \Omega_{l}\left(M,{\left\{N_{l}\right\}}_{d}\right)= & \frac{\left(M-k\;N_{k}\right)!\prod_{i=1}^{d}\left[M-\left(k-1\right)N_{k,i}-\left(l-1\right)N_{l,i}\right]!}{\left(M-k\;N_{k}-l\;N_{l}\right)!\prod_{i=1}^{d}\left[M-\left(k-1\right)N_{k,i}\right]!\left(N_{l,i}\right)!}. \end{array}$$

Then, the product obtained from Equations (97) and (100) gives the total number of ways to pack the molecules in the mixture:

(101) $$\Omega \left(M,{\left\{N_{k}\right\}}_{d},{\left\{N_{l}\right\}}_{d}\right)=\frac{\prod_{i=1}^{d}\left[M-\left(k-1\right)N_{k,i}-\left(l-1\right)N_{l,i}\right]!}{\left(M-k\;N_{k}-l\;N_{l}\right)!{\left(M!\right)}^{d-1}\prod_{i=1}^{d}\left(N_{k,i}\right)!\left(N_{l,i}\right)!}.$$

Equation (101) is exact when all molecules are oriented in one direction [100]. For the case of an isotropic distribution of molecules—i.e., $N_{k(l),i}=\left(2/\gamma \right)N_{k(l)}$—then the appropriate generalization of Equation (101) is

(102) $$\Omega \left(M,N_{k},N_{l}\right)=\frac{M!}{(M-kN_{k}-lN_{l})!}{\left\{\frac{\left[M-\left(k-1\right)\frac{\gamma }{2}N_{k}-\left(l-1\right)\frac{\gamma }{2}N_{l}\right]!}{M!\;N_{k}!\;N_{l}!}\right\}}^{\frac{\gamma }{2}}.$$

In the canonical ensemble, the Helmholtz free energy $F(M,N_{k},N_{l},T)$ relates to $\Omega (M,N_{k},N_{l})$ as follows:

(103) $$\begin{array}{cc} \beta F(M,N_{k},N_{l},T) & =-\ln Q(M,N_{k},N_{l},T)\\ \; & =-\ln\Omega \left(M,N_{k},N_{l}\right)+\beta \epsilon \left(N_{k},N_{l}\right). \end{array}$$

The chemical potential of the adsorbed species *i*, $\mu_{i,ads}$, can be calculated as [80]:

(104) $$\mu_{i,ads}={\left(\frac{\partial F}{\partial N_{i}}\right)}_{N_{j}}\;\;\;\;\;\;\;\;\;\left\{i,j=k,l\right\}.$$

From Equations (102)–(104), it follows that

(105) $$\begin{array}{cc} \beta \left(\mu_{k,ads}-\epsilon_{k}\right)= & \left(k-1\right)\ln\left[\frac{\gamma }{2}-\frac{\left(k-1\right)}{k}\theta_{k}-\frac{\left(l-1\right)}{l}\theta_{l}\right]+\ln\left(\frac{\theta_{k}}{k}\right)\\ \; & -k\ln\left(1-\theta_{k}-\theta_{l}\right)-k\ln\left(\frac{\gamma }{2}\right), \end{array}$$

and

(106) $$\begin{array}{cc} \beta \left(\mu_{l,ads}-\epsilon_{l}\right)= & \left(l-1\right)\ln\left[\frac{\gamma }{2}-\frac{\left(k-1\right)}{k}\theta_{k}-\frac{\left(l-1\right)}{l}\theta_{l}\right]+\ln\left(\frac{\theta_{l}}{l}\right)\\ \; & -l\ln\left(1-\theta_{k}-\theta_{l}\right)-l\ln\left(\frac{\gamma }{2}\right), \end{array}$$

where $\theta_{i}=iN_{i}/M$ represents the partial coverage of the species *i*{i=k,l}. At equilibrium, the chemical potential of the adsorbed and gas phases are equal. Then,

(107) $$\mu_{k,ads}=\mu_{k,gas},$$

and

(108) $$\mu_{l,ads}=\mu_{l,gas},$$

where $\mu_{k,gas}$ ($\mu_{l,gas}$) corresponds to *k*-mers (*l*-mers) in gas phase and can be obtained using Equations (91)–(93).

#### 3.2.3. SE Approximation

We start by applying concepts previously introduced in Section 3.1.3 for the mixture problem.

In the grand canonical ensemble, the mean number of *i*-mers in adlayer ${\bar{N}}_{i}$ and the chemical potential $\mu_{i,ads}$ are related through the following general relationship:

(109) $${\bar{N}}_{i}=\lambda_{i}\;{\left[\frac{\partial \ln\Xi \left(M,\lambda_{k},\lambda_{l}\right)}{\partial \lambda_{i}}\right]}_{M}\;\left\{i=k,l\right\},$$

with $\lambda_{i}=\exp\left[\beta \left(\mu_{i,ads}-\epsilon_{i}\right)\right]$. As in Refs. [93,95], the solution of Equation (109) for $\lambda_{i}$ gives us the balance of occupancy for the system,

(110) $$\lambda_{i}^{-1}=\frac{R_{i}\left(M,\lambda_{k},\lambda_{l}\right)}{{\bar{N}}_{i}}\;\left\{i=k,l\right\},$$

where $R_{i}\left(M,\lambda_{k},\lambda_{l}\right)$ can be interpreted as the number of states available for a particle of species *i* to adsorb when the chemical potentials at the surface are $\mu_{k,ads}$ and $\mu_{l,ads}$. These states can be written as

(111) $$R_{i}\left(M,\lambda_{k},\lambda_{l}\right)=\left(\frac{\gamma }{2}M\right)\prod_{j=1}^{i}P_{j}\;\left\{i=k,l\right\}.$$

The term between parentheses corresponds to the total number of *i*-tuples on the surface. These *i*-tuples can be separated into three different groups: (1) full *i*-tuples (occupied by *i*-mers); (2) empty *i*-tuples (available for adsorption), and (3) frustrated *i*-tuples (partially occupied or occupied by segments belonging to different adsorbed *i*-mers). However, the additional factor represents the probability of having an empty linear *i*-tuple. As in Ref. [11], we suppose that this probability can be written as a product of *i* conditional probability functions $P_{j}$, representing the probability of finding the *j*-th site in the lattice empty when there are j−1 already vacant sites. The first of these functions (i.e., with j=1) represents the probability of finding an empty site when there are already $N=N_{k}+N_{l}$ adsorbed molecules on the lattice, such that

(112) $$P_{1}=1-\theta ,$$

in which $\theta =\theta_{k}+\theta_{l}$ is the total surface coverage and $\theta_{i}=iN_{i}/M$ is the surface coverage of species *i*.

Furthermore, the functions $P_{j}$ can be written as [11]

(113) $$P_{j}=\left(1-\theta \right)C_{j},$$

where $C_{j}$ are undetermined correction functions that must satisfy $C_{1}=1$ and $C_{j}\to 1$ as θ→0. From Equations (111) and (113), we obtain

(114) $$R_{i}\left(M,\lambda_{k},\lambda_{l}\right)=\frac{\gamma }{2}M{\left(1-\theta \right)}^{i}\prod_{j=2}^{i}C_{j}=\frac{\gamma }{2}M{\left(1-\theta \right)}^{i}{\tilde{C}}^{i-1},$$

and

(115) $$\tilde{C}={\left(\prod_{j=2}^{i}C_{j}\right)}^{\frac{1}{i-1}}\;\left\{i=k,l\right\},$$

where $\tilde{C}$ is the geometric mean of the $C_{j}$. Now, the adsorption isotherms can be easily calculated from Equations (110) and (114):

(116) $$\begin{array}{c} \lambda_{i}^{-1}=\frac{\gamma }{2}i\frac{{\left(1-\theta \right)}^{i}{\tilde{C}}^{i-1}}{\theta_{i}}\;\left\{i=k,l\right\}, \end{array}$$

or

(117) $$\begin{array}{cc} \beta \left(\mu_{i,ads}-\epsilon_{i}\right)= & \ln\left(\frac{\theta_{i}}{i}\right)-i\ln\left(1-\theta \right)-\ln\left(\frac{\gamma }{2}\right)-\left(i-1\right)\ln\tilde{C}\;\left\{i=k,l\right\}. \end{array}$$

In general, calculation of the adsorption isotherms requires the knowledge of an analytical expression for $\tilde{C}$ [see Equation (115)]. Now, let us consider the simplest approximation within this scheme; namely, when $\tilde{C}=1$ for all *k* and *l*. Then, from Equation (117), we obtain

(118) $$\begin{array}{cc} \beta \left(\mu_{i,ads}-\epsilon_{i}\right)= & \ln\left(\frac{\theta_{i}}{i}\right)-i\ln\left(1-\theta \right)-\ln\left(\frac{\gamma }{2}\right)\;\left\{i=k,l\right\}. \end{array}$$

Equation (118) represents the Flory–Huggins (FH) limit [6,7] for the adsorption of non-interacting binary mixtures of linear species adsorbed onto homogeneous surfaces. For single adsorption, Equation (118) reduces to the classical Flory–Huggins isotherm for non-interacting linear adsorbates on homogeneous surfaces [11].

In the case of EA and GD approximation, comparison between Equation (117) and Equations (87), (88), (105) and (106) allows us to obtain:

(119) $$\tilde{C}=\left[1-\frac{\left(k-1\right)}{k}\theta_{k}-\frac{\left(l-1\right)}{l}\theta_{l}\right]\;\;\left(\mathrm{EA}\right),$$

and

(120) $$\tilde{C}=\left[1-\frac{\left(k-1\right)}{k}\frac{2\theta_{k}}{\gamma }-\frac{\left(l-1\right)}{l}\frac{2\theta_{l}}{\gamma }\right]\;\;\left(\mathrm{GD}\right).$$

As was shown in the case of single-component adsorption, an excellent approximation can be obtained by combining exact calculations in 1D and the GD approximations with adequate weights [11]. Extending these arguments to the case of multicomponent adsorption and using the structure proposed in Equation (117), a new semi-empirical adsorption isotherm for polyatomic mixtures can be built:

(121) $$\begin{array}{cc} \beta \left(\mu_{i,ads}-\epsilon_{i}\right)= & \ln\left(\frac{\theta_{i}}{i}\right)-i\ln\left(1-\theta \right)-\ln\left(\frac{\gamma }{2}\right)\\ & -\left(1-\theta \right)\left(i-1\right)\ln\left[1-\frac{\left(k-1\right)}{k}\frac{2\theta_{k}}{\gamma }-\frac{\left(l-1\right)}{l}\frac{2\theta_{l}}{\gamma }\right]\\ & -\theta \left(i-1\right)\ln\left[1-\frac{\left(k-1\right)}{k}\theta_{k}-\frac{\left(l-1\right)}{l}\theta_{l}\right],\;\;i=\left\{k,l\right\}. \end{array}$$

The equation above can be interpreted as follows. The first line includes three terms that are identical in both EA and GD. The second and third lines represent a combination of the average correction functions corresponding to GD and EA, with (1−θ) and θ as weights, respectively.

### 3.3. Multilayer Adsorption in the Presence of Multisite Occupancy: Theoretical Approach for 2D Substrates

This section presents a semi-analytical model for describing the multilayer adsorption of polyatomic molecules on two-dimensional surfaces. The surface is idealized as a uniform grid of adsorption sites, while the adsorbing species are considered as linear chains composed of *k* identical segments, each occupying a single lattice site.

As established in Section 2, we adopt the following assumptions: (*i*) vertical stacking is allowed, such that each *k*-mer in a given layer may lie directly atop another from the layer beneath; (ii) lateral interactions between adsorbates are neglected; and (iii) the adsorption energy is constant for all layers beyond the first, being equal to the molar heat of liquefaction of the adsorbate. Consequently, the parameter $c=q_{1}/q_{i}=q_{1}/q$ with $q_{i}=q\;\;\;\left(i=2,\ldots ,\infty \right)$ represents the ratio of partition functions between the first and subsequent layers.

On a lattice with *M* sites, the highest number of vertical columns that can be formed is $n_{max}=M/k$. Let $\Omega_{k}\left(n,M\right)$ denote the total number of distinct arrangements of *n* such vertical columns. Assuming an unrestricted number of layers, the grand partition function $\Xi_{mul}$, representing the equilibrium between the surface phase and a gas phase with chemical potential μ and temperature *T*, is expressed as:

(122) $$\Xi_{mul}=\sum_{n=0}^{n_{max}}\Omega_{k}\left(n,M\right)\;\;\xi^{n},$$

where ξ denotes the grand partition function of a single column containing at least one *k*-mer in contact with the surface, which is given by:

(123) $$\xi =\sum_{i=1}^{\infty }q_{1}q^{i-1}\lambda_{mul}^{i}=c\sum_{i=1}^{\infty }q^{i}\lambda_{mul}^{i}=\frac{c\lambda_{mul}q}{1-\lambda_{mul}q}=\frac{cx}{1-x},$$

with the fugacity defined as $\lambda_{mul}=\exp\left(\mu /k_{B}T\right)$ and the reduced pressure expressed as $x=\lambda_{mul}q=P/P_{0}$, following standard thermodynamic conventions [37,101,102].

Likewise, the monolayer grand partition function, $\Xi_{mon}$, is defined as:

(124) $$\Xi_{mon}=\sum_{n=0}^{n_{max}}\Omega_{k}\left(n,M\right)\;\;\lambda_{mon}^{n},$$

where $\lambda_{mon}$ is the monolayer fugacity and *n* corresponds to the number of *k*-mers adsorbed in the first layer.

Comparing Equations (122) and (124), and considering the condition

(125) $$\begin{array}{ccc} \lambda_{mon} & = & \xi \\ & = & \frac{cP/P_{0}}{1-P/P_{0}}\Rightarrow \frac{P}{P_{0}}=\frac{1}{1+c\lambda_{mon}^{-1}}, \end{array}$$

we can determine the monolayer coverage, $\theta_{mon}$, as

(126) $$\theta_{mon}=\frac{k}{M}\tilde{n}=\frac{k}{M}\lambda_{mon}{\left(\frac{d\ln\Xi_{mon}}{d\lambda_{mon}}\right)}_{M,T}=\frac{k}{M}\xi {\left(\frac{d\ln\Xi_{mul}}{d\xi }\right)}_{M,T},$$

where $\tilde{n}$ is the mean number of columns.

The total surface coverage θ, which includes contributions from all layers, is defined as:

(127) $$\theta =\frac{k}{M}\tilde{N}=\frac{k}{M}\lambda_{mul}{\left(\frac{d\ln\Xi_{mul}}{d\lambda_{mul}}\right)}_{M,T},$$

with $\tilde{N}$ being the mean total number of adsorbed *k*-mers. After suitable algebraic manipulation, a functional relationship between total coverage and monolayer coverage can be established:

(128) $$\begin{array}{ccc} \theta & = & \frac{k}{M}\lambda_{mul}{\left(\frac{d\ln\Xi_{mul}}{d\xi }\right)}_{M,T}\frac{d\xi }{d\lambda_{mul}}\\ & = & \frac{\theta_{mon}}{\left(1-P/P_{0}\right)}. \end{array}$$

In summary, to determine the multilayer adsorption behavior, the following two-step method is employed [102]:

(1) Use $\theta_{mon}$ as an input parameter (ranging from 0 to 1), and determine the corresponding reduced pressure $P/P_{0}$ using Equation (125) and an analytical expression for the monolayer adsorption isotherm.
(2) Substitute the values of $\theta_{mon}$ and $P/P_{0}$ into the total coverage Equation (128) to compute θ.

Items (1) and (2) above are summarized in the following scheme:

(129) $$\theta_{mon}+\lambda_{mon}\left(\theta_{mon}\right)+\mathrm{Equation}\;(125)\to P/P_{0}\;\;\;\;\;\;\;\Rightarrow \;\;\;\;\;\;\;\theta_{mon}+P/P_{0}+\mathrm{Equation}\;(128)\to \theta$$

This approach enables an approximate, yet accurate description of multilayer adsorption for polyatomic species on 2D lattices.

From a theoretical standpoint, obtaining a closed-form solution for multilayer adsorption on a 2D surface that includes both vacancies and *k*-mers (for k>1) remains unresolved. However, a variety of approximate treatments have been developed. Among them, the Occupation Balance (OB) method [93] has shown strong agreement with simulation data. In the simplest case of dimers (k=2), the OB approach provides an analytical form for the monolayer isotherm on a honeycomb lattice:

(130) $${\lambda_{mon}}^{-1}=\frac{3}{\theta_{mon}}-5+\frac{4}{3}\theta_{mon}+\frac{2}{3}\theta_{mon}^{2}\;\;\;\;\;\;\;\;\;\;\;\left(\mathrm{honeycomb}\;\mathrm{lattice}\right).$$

For lattices of different connectivity, including square and triangular geometries, analogous expressions are available:

(131) $${\lambda_{mon}}^{-1}=\frac{4}{\theta_{mon}}-7+\frac{9}{4}\theta_{mon}+\frac{3}{4}\theta_{mon}^{2}\;\;\;\;\;\;\;\;\;\;\;\left(\mathrm{square}\;\mathrm{lattice}\right),$$

and

(132) $${\lambda_{mon}}^{-1}=\frac{6}{\theta_{mon}}-11+\frac{23}{6}\theta_{mon}+\frac{7}{6}\theta_{mon}^{2}\;\;\;\;\;\;\;\;\;\;\;\left(\mathrm{triangular}\;\mathrm{lattice}\right).$$

The relative pressures for honeycomb, square, and triangular lattices are obtained by inserting Equations (130)–(132), respectively, into Equation (125). In this way, we obtain

(133) $$\frac{P}{P_{0}}=\frac{3\theta_{mon}}{9c+\left(3-15c\right)\theta_{mon}+4c\theta_{mon}^{2}+2c\theta_{mon}^{3}}\;\;\;\;\;\;\;\;\;\;\;\left(\mathrm{honeycomb}\;\mathrm{lattice}\right),$$

(134) $$\frac{P}{P_{0}}=\frac{4\theta_{mon}}{16c+\left(4-28c\right)\theta_{mon}+9c\theta_{mon}^{2}+3c\theta_{mon}^{3}}\;\;\;\;\;\;\;\;\;\;\;\left(\mathrm{square}\;\mathrm{lattice}\right),$$

and

(135) $$\frac{P}{P_{0}}=\frac{6\theta_{mon}}{36c+\left(6-66c\right)\theta_{mon}+23c\theta_{mon}^{2}+7c\theta_{mon}^{3}}\;\;\;\;\;\;\;\;\;\;\;\left(\mathrm{triangular}\;\mathrm{lattice}\right).$$

The expressions (133)–(135), together with the coverage relation [Equation (128)], offer a consistent model for multilayer adsorption. This treatment incorporates entropic contributions stemming from molecular size, representing a significant improvement over classical multilayer models.

To generalize beyond dimers, the EA model [93] is employed. The monolayer isotherm for arbitrary *k*-mers is given by [20,93]:

(136) $$\lambda_{mon}=\frac{\theta_{mon}}{k\;K(\gamma ,k)}\frac{{\left[1-\frac{\left(k-1\right)}{k}\theta_{mon}\right]}^{k-1}}{{\left(1-\theta_{mon}\right)}^{k}}$$

where γ is the lattice connectivity and K(γ,k) denotes the number of configurations per site for a *k*-mer at vanishing coverage [as per Equation (34)].

Combining the EA monolayer isotherm with the multilayer adsorption framework outlined above, we derive an analytical expression for the multilayer adsorption of linear *k*-mers on 2D lattices with arbitrary connectivity γ,

(137) $$\frac{P}{P_{0}}=\frac{\theta_{mon}{\left[1-\frac{\left(k-1\right)}{k}\theta_{mon}\right]}^{k-1}}{k\;K\left(\gamma ,k\right)\;c{\left(1-\theta_{mon}\right)}^{k}+\theta_{mon}{\left[1-\frac{\left(k-1\right)}{k}\theta_{mon}\right]}^{k-1}}.$$

## 4. Two-Dimensional Lattice Gases of Interacting Polyatomics

Incorporating intermolecular interactions into the analysis opens the door to study the emergence of phase transitions [103,104,105,106,107]. Common examples of such transitions include gas condensation, solid melting, shifts from paramagnetic to ferromagnetic states, and various order–disorder transitions. Theoretically, when interactions between nearest neighbors are considered, an additional term representing interaction energy must be included in the system’s partition function. This modification means that the partition function can only be evaluated for the system as a whole, rather than for individual components.

While the one-dimensional lattice model was exactly solved by Ising in 1925 [108], beyond this case, most models are only tractable through series expansions [80,109,110]; with the exception of a two-dimensional lattice at half-coverage, which was analytically solved by Onsager in 1944 [111]. In one dimension, the absence of a true phase transition is well established. However, for higher-dimensional systems, approximate methods can provide valuable insights. Two of the most influential approximations are the Bragg–Williams approximation (BWA) [80] and the quasi-chemical approximation (QCA) [80,112]. Both predict phase transitions in two-dimensional systems, although the BWA incorrectly forecasts such behavior in one-dimensional systems.

These foundational models, along with more recent developments, have significantly advanced our understanding of adsorption phenomena where lateral interactions between adsorbates are relevant. A key simplifying assumption common to all these approaches is that each adsorbed molecule occupies a single adsorption site. In the present work, we extend the BWA and QCA frameworks to incorporate the case of adsorbates that span multiple adsorption sites (i.e., multisite occupancy) [30,31].

### 4.1. Mean-Field Approximation for Interacting k-mers Adsorbed onto 2D Substrates

The Bragg–Williams approach represents the most basic mean-field model applied to systems of adsorbed interacting particles, including those involving multisite occupancy scenarios [30]. Within this framework, the canonical partition function Q(N,M,T) describes a system composed of *N k*-mers adsorbed onto a substrate with *M* adsorption sites at temperature *T*, accounting for lateral interactions of strength *w* between neighboring adsorbates. It is defined as follows:

(138) $$Q\left(N,M,T\right)=\sum_{\left\{E_{k}\right\}}\Omega \left(E_{k}\right)e^{-\beta E_{k}\left(N,M\right)},$$

where $\Omega (E_{k})$ denotes the total number of states corresponding to energy $E_{k}$ for *N k*-mers arranged on *M* sites. Applying a mean-field assumption at this stage yields

(139) $$Q\left(N,M,T\right)=e^{-\beta \bar{E_{k}\left(N,M\right)}}\sum_{\left\{E_{k}\right\}}\Omega \left(E_{k}\right)=e^{-\beta \bar{E_{k}\left(N,M\right)}}\Omega \left(N,M,\gamma \right).$$

In this context, $\bar{E_{k}\left(N,M\right)}$ represents the average energy of the system, under the assumption that the kN occupied positions are randomly distributed across the lattice containing *M* sites. Additionally, the term Ω(N,M,γ) is influenced by both the structural properties of the *k*-mers and the geometry of the substrate. For even the relatively straightforward case of linear *k*-mers, an exact analytical form of Ω(N,M,γ) in two or more dimensions is not available. Nonetheless, as outlined in Section 3, various approximation methods have been proposed for the evaluation of Ω(N,M,γ). In this study, we calculate Ω(N,M,γ) based on the EA model described in Equation (43).

The mean total energy can be expressed as:

(140) $$\bar{E_{k}\left(N,M\right)}=kN\epsilon_{0}+\frac{1}{2}\lambda N\left(\frac{kN}{M}\right)w,$$

where the first term on the right-hand side accounts for the interaction between *k*-mers and the surface, while the second term captures the lateral interactions between adsorbed *k*-mers. The parameter λ, which denotes the number of neighboring lattice sites adjacent to a linearly adsorbed *k*-mer, is given by $\lambda =\left[2(\gamma -1)+(k-2)(\gamma -2)\right]$.

Consequently, the canonical partition function can be rewritten as:

(141) $$Q\left(N,M,T\right)=K{\left(\gamma ,k\right)}^{N}\frac{[M-(k-1)N]!}{N![M-kN]!}\;\;e^{-(kN\epsilon_{0}+\frac{1}{2}\lambda k\frac{N^{2}}{M}w)/k_{B}T}.$$

From this, the Helmholtz free energy F(N,M,T) can be obtained as:

(142) $$\begin{array}{ccc} \beta F(N,M,T) & = & \ln Q(N,M,T)\\ & = & \ln\Omega \left(N,M,\gamma \right)-\beta kN\epsilon_{0}-\frac{1}{2}\beta w\lambda k\frac{N^{2}}{M}\\ & = & \ln[M-(k-1)N]!-\ln N!-\ln[M-kN]!+N\ln K(\gamma ,k)\\ & & -\beta kN\epsilon_{0}-\frac{1}{2}\beta w\lambda k\frac{N^{2}}{M}. \end{array}$$

The Helmholtz free energy per site, as a function of surface coverage and temperature, is:

(143) $$\begin{array}{ccc} \beta f(\theta ,T) & = & -\left[1-\frac{k-1}{k}\theta \right]\ln\left[1-\frac{k-1}{k}\theta \right]+\frac{\theta }{k}\ln\frac{\theta }{k}+\left(1-\theta \right)\ln\left(1-\theta \right)-\frac{\theta }{k}\ln K\left(\gamma ,k\right)\\ & + & \beta \theta \epsilon_{0}+\frac{1}{2}\beta \lambda w.\frac{\theta^{2}}{k} \end{array}$$

Accordingly, *s* is given by

(144) $$\frac{s(\theta )}{k_{B}}=\left[1-\frac{k-1}{k}\theta \right]\ln\left[1-\frac{k-1}{k}\theta \right]-\frac{\theta }{k}\ln\frac{\theta }{k}-\left(1-\theta \right)\ln\left(1-\theta \right)+\frac{\theta }{k}\ln K\left(\gamma ,k\right),$$

and the isotherm equation takes the form

(145) $$C_{k}K\left(\gamma ,k\right)\exp\left[\beta (\mu -k\epsilon_{0})\right]=\frac{\theta {\left[1-\frac{\left(k-1\right)}{k}\theta \right]}^{\left(k-1\right)}}{{\left(1-\theta \right)}^{k}}e^{\beta \lambda w\theta }$$

where $C_{k}=k$.

### 4.2. Quasi-Chemical Approximation for Interacting k-mers Adsorbed onto 2D Substrates

In this section, we examine the general case of interacting adsorbed species modeled as linear chains within the framework of the quasi-chemical approximation (QCA) [30]. As discussed previously, two distinct energy contributions are involved in the adsorption process: (1) $\epsilon_{0}$, representing a constant energy associated with the interaction between a single unit of a *k*-mer and an adsorption site; and (2) *w*, denoting the lateral interaction energy between adjacent units from different *k*-mers. Under these assumptions, the canonical partition function can be expressed as [80]:

(146) $$Q\left(N,M,T\right)=\sum_{N_{11}}\Omega \left(N,M,N_{11}\right)\exp\left[-\beta \left(wN_{11}+kN\epsilon_{0}\right)\right],$$

where $N_{11}$ represents the number of nearest-neighbor pairs formed by units from different *k*-mers, while $\Omega (N,M,N_{11})$ denotes the number of possible configurations of *N k*-mers over *M* sites that result in exactly $N_{11}$ such occupied neighbor pairs.

Following the approach commonly used for single-site adsorption, it is convenient to rewrite the partition function in terms of $N_{01}$, which counts pairs of adjacent sites where one is occupied and the other is empty. To do this, the relationships among $N_{11}$, $N_{01}$, and $N_{00}$ (empty-empty pairs) must be determined,

(147) $$2N_{11}+N_{01}+2N\left(k-1\right)=\gamma kN,$$

(148) $$2N_{00}+N_{01}=\gamma \left(M-kN\right).$$

Due to lattice symmetry, the number of 01 and 10 pairs are equal; that is, the number of 01 pairs = the number of 10 pairs = $N_{01}/2$.

In the limiting case of k=1, the conventional expressions for single-site adsorption are recovered [80].

The canonical partition function can now be rewritten in terms of $N_{01}$:

(149) $$Q\left(N,M,T\right)=\exp\left[-\beta N\left(k\epsilon_{0}+\lambda w/2\right)\right]\sum_{N_{01}}\Omega \left(N,M,N_{01}\right)\exp\left(\beta wN_{01}/2\right),$$

with λ=(γ−2)k+2.

Using the standard formalism of the QCA, the number of ways to assign a total of $\left[\gamma M/2-N(k-1)\right]$ independent pairs (the term N(k−1) is subtracted as the total number of nearest-neighbor pairs, γM/2, includes the N(k−1) bonds belonging to the *N* adsorbed *k*-mers) to the four categories 11,10,01, and 00, with any number 0 through $\left[\gamma M/2-N(k-1)\right]$ per category consistent with the total, is

(150) $$\tilde{\Omega }\left(N,M,N_{01}\right)=\frac{\left[\gamma M/2-N(k-1)\right]!}{{\left[\left(N_{01}/2\right)!\right]}^{2}\left[\gamma \left(M-kN\right)/2-N_{01}/2\right]!\left[\lambda N/2-N_{01}/2\right]!}.$$

It is important to emphasize that $\Omega (N,M,N_{01})$ cannot be directly equated with the combinatorial count $\tilde{\Omega }\left(N,M,N_{01}\right)$, as this would include non-physical configurations arising from the assumption that all pairs are independent ([80], p. 253). Therefore, $\tilde{\Omega }$ must be normalized to reflect only physically valid configurations:

(151) $$\Omega \left(N,M,N_{01}\right)=C\left(N,M,\gamma \right)\;\tilde{\Omega }\left(N,M,N_{01}\right),$$

and

(152) $$\Omega \left(N,M,\gamma \right)=\sum_{N_{01}}\Omega \left(N,M,N_{01}\right)=C\left(N,M,\gamma \right)\sum_{N_{01}}\;\tilde{\Omega }\left(N,M,N_{01}\right).$$

Once an approximation for Ω(N,M,γ) has been adopted, as described in Section 3, the normalization factor C(N,M,γ) can be determined.

To evaluate C(N,M,γ), the sum $\sum_{N_{01}}\tilde{\Omega }\left(N,M,N_{01}\right)$ is approximated by its dominant contribution, $\tilde{\Omega }\left(N,M,N_{01}^{*}\right)$. Taking the logarithm of this term and applying Stirling’s approximation yields

(153) $$\begin{array}{ccc} \ln\tilde{\Omega }\left(N,M,N_{01}\right) & = & \left[\gamma M/2-(k-1)N\right]\ln\left[\gamma M/2-(k-1)N\right]-N_{01}\ln N_{01}/2\\ & & -\left[\gamma \left(M-kN\right)/2-N_{01}/2\right]\ln\left[\gamma \left(M-kN\right)/2-N_{01}/2\right]\\ & & -\left(\lambda N/2-N_{01}/2\right)\ln\left(\lambda N/2-N_{01}/2\right). \end{array}$$

Differentiating the last equation with respect to $N_{01}$, we obtain

(154) $${\tilde{\Omega }}^{\prime }\left(N,M,N_{01}\right)=\frac{\tilde{\Omega }\left(N,M,N_{01}\right)}{2}\ln\left\{\frac{\left[\gamma \left(M-kN\right)-N_{01}\right]\left(\lambda N-N_{01}\right)}{N_{01}^{2}}\right\}.$$

Setting ${\tilde{\Omega }}^{\prime }\left(N,M,N_{01}\right)=0$ and solving for $N_{01}^{*}$, the value of $N_{01}$ in the maximum term of $\tilde{\Omega }$,

(155) $$N_{01}^{*}=\frac{\gamma \lambda N\left(M-kN\right)}{\gamma M-2(k-1)N}=\lambda N-\frac{\lambda^{2}N^{2}}{\gamma B},$$

and

(156) B=M−2(k−1)N/γ.

Then,

(157) $$\tilde{\Omega }\left(N,M,N_{01}^{*}\right)=\frac{\left(\gamma B/2\right)!}{{\left[\left(\lambda N/2-\frac{\lambda^{2}N^{2}}{2\gamma B}\right)!\right]}^{2}\left(\gamma B/2-\lambda N+\frac{\lambda^{2}N^{2}}{2\gamma B}\right)!\left(\frac{\lambda^{2}N^{2}}{2\gamma B}\right)!}$$

and, by simple algebra,

(158) $$\tilde{\Omega }\left(N,M,N_{01}^{*}\right)={\left[\frac{B!}{\left(B-\lambda N/\gamma \right)!\left(\lambda N/\gamma \right)!}\right]}^{\gamma }.$$

Equation (158) allows us to calculate C(N,M,γ),

(159) $$\begin{array}{ccc} C(N,M,\gamma ) & = & \frac{\Omega (N,M,\gamma )}{\tilde{\Omega }\left(N,M,N_{01}^{*}\right)}\\ & = & \Omega \left(N,M,\gamma \right){\left[\frac{\left(B-\lambda N/\gamma \right)!\left(\lambda N/\gamma \right)!}{B!}\right]}^{\gamma }. \end{array}$$

Now, lnQ(N,M,T) [see Equation (149)] can be written as

(160) $$\ln Q\left(N,M,T\right)=-\beta N\left(k\epsilon_{0}+\lambda w/2\right)+\ln\left\{\sum_{N_{01}}C\left(N,M,\gamma \right)\tilde{\Omega }\left(N,M,N_{01}\right)\exp\left(\beta wN_{01}/2\right)\right\}.$$

As in Equation (152), we replace $\sum_{N_{01}}C\left(N,M,\gamma \right)\tilde{\Omega }\left(N,M,N_{01}\right)\exp\left(\beta wN_{01}/2\right)$ by the maximum term in the sum, $C\left(N,M,\gamma \right)\tilde{\Omega }\left(N,M,N_{01}^{**}\right)\exp\left(\beta wN_{01}^{**}/2\right)$. Thus,

(161) $$C\left(N,M,\gamma \right){\tilde{\Omega }}^{\prime }\left(N,M,N_{01}^{**}\right)\exp\left(\beta wN_{01}^{**}/2\right)+C\left(N,M,\gamma \right)\tilde{\Omega }\left(N,M,N_{01}^{**}\right)\exp\left(\beta wN_{01}^{**}/2\right)\beta w/2=0,$$

and

(162) $$\frac{{\tilde{\Omega }}^{\prime }\left(N,M,N_{01}^{**}\right)}{\tilde{\Omega }\left(N,M,N_{01}^{**}\right)}=-\beta w/2.$$

From Equations (154) and (162),

(163) $$\left(\gamma B-\lambda N-N_{01}^{**}\right)\left(\lambda N-N_{01}^{**}\right)={N_{01}^{**}}^{2}\exp\left(-\beta w\right)$$

and

(164) $$\left[1-\exp\left(-\beta w\right)\right]{N_{01}^{**}}^{2}-\gamma BN_{01}^{**}+\left(\gamma B-\lambda N\right)\lambda N=0.$$

Solving Equation (164), we obtain

(165) $$\frac{N_{01}^{**}}{\gamma B}=\frac{1-\sqrt{1-4A\left(1-\lambda N/\gamma B\right)\left(\lambda N/\gamma B\right)}}{2A}.$$

where $A=1-\exp\left(-\beta w\right)$. Note that the solution $N_{01}^{**}/\gamma B=\left(1+\sqrt{\ldots }\right)/2A$ is discarded for physical reasons.

Finally, the canonical partition function can be written in terms of $N_{01}^{**}$:

(166) $$\begin{array}{ccc} Q(N,M,T) & = & \exp\left[-\beta N(k\epsilon_{0}+\lambda w/2)\right]\Omega \left(N,M,\gamma \right){\left[\frac{\left(B-\lambda N/\gamma \right)!\left(\lambda N/\gamma \right)!}{B!}\right]}^{\gamma }\\ & & \tilde{\Omega }\left(N,M,N_{01}^{**}\right)\exp\left(\beta wN_{01}^{**}/2\right) \end{array}$$

As in the previous section, we use the following expression for Ω(N,M,γ):

(167) $$\Omega \left(N,M,\gamma \right)=K{\left(\gamma ,k\right)}^{N}\frac{\left(B-\lambda N/\gamma +N\right)!}{N!\left(B-\lambda N/\gamma \right)!},$$

which is an extension to two dimensions of the exact configurational factor obtained in one dimension [Equation (43)]. In the particular case of rigid straight *k*-mers, the simplest approximation provides K(γ,k)=γ/2 (k≥2).

Introducing Equation (167) into Equation (166), taking the logarithm and using the Stirling’s approximation, we obtain

(168) $$\begin{array}{ccc} \ln Q(N,M,T) & = & -\beta N\left(k\epsilon_{0}+\lambda w/2\right)+N\ln K\left(\gamma ,k\right)+\beta wN_{01}^{**}/2\\ & & +\left(B-\lambda N/\gamma +N\right)\ln\left(B-\lambda N/\gamma +N\right)-N\ln N\\ & & +\left(\gamma -1\right)\left(B-\lambda N/\gamma \right)\ln\left(B-\lambda N/\gamma \right)+\lambda N\ln\lambda N/\gamma \\ & & -\gamma B\ln B+\gamma B/2\ln\gamma B/2-N_{01}^{**}\ln N_{01}^{**}/2\\ & & -\left(\gamma B/2-\lambda N/2-N_{01}^{**}/2\right)\ln\left(\gamma B/2-\lambda N/2-N_{01}^{**}/2\right)\\ & & -\left(\lambda N/2-N_{01}^{**}/2\right)\ln\left(\lambda N/2-N_{01}^{**}/2.\right) \end{array}$$

From Equation (175), the Helmholtz free energy per site, f(N,M,T), can be obtained as a function of the surface coverage and temperature:

(169) $$\begin{array}{ccc} \beta f(\theta ,T) & = & -\frac{\theta }{k}\ln K\left(\gamma ,k\right)+\beta \epsilon_{0}\theta +\beta w\left(\frac{\lambda \theta }{2k}-\alpha \right)\\ & & -\left[\frac{\gamma }{2}-\left(\frac{k-1}{k}\right)\theta \right]\ln\left\{\frac{{\left[1-\left(\frac{k-1}{k}\right)\theta \right]}^{2/\gamma }{\left(1-\theta \right)}^{2(\gamma -1)/\gamma }\left[\frac{\gamma }{2}-\left(\frac{k-1}{k}\right)\theta \right]}{{\left[1-\frac{2}{\gamma }\left(\frac{k-1}{k}\right)\theta \right]}^{2}\left[\frac{\gamma }{2}\left(1-\theta \right)-\alpha \right]}\right\}\\ & & -\frac{\theta }{k}\ln\left\{\frac{{\left(\frac{\lambda \theta }{\gamma k}\right)}^{\lambda }{\left[\frac{\gamma }{2}\left(1-\theta \right)-\alpha \right]}^{\lambda /2}}{\frac{\theta }{k}{\left[1-\left(\frac{k-1}{k}\right)\theta \right]}^{(\lambda -\gamma )/\gamma }{\left(1-\theta \right)}^{(\lambda \gamma -\lambda )/\gamma }{\left[\frac{\lambda \theta }{2k}-\alpha \right]}^{\lambda /2}}\right\}\\ & & -2\alpha \ln\left\{\frac{{\left[\frac{\gamma }{2}\left(1-\theta \right)-\alpha \right]}^{1/2}{\left[\frac{\lambda \theta }{2k}-\alpha \right]}^{1/2}}{\alpha }\right\} \end{array}$$

where α is given by

(170) $$\alpha =\frac{N_{01}^{**}}{2M}=\frac{\lambda \gamma }{2k}\frac{\theta (1-\theta )}{\left[\frac{\gamma }{2}-\left(\frac{k-1}{k}\right)\theta +b\right]}$$

and

(171) $$b={\left\{{\left[\frac{\gamma }{2}-\left(\frac{k-1}{k}\right)\theta \right]}^{2}-\frac{\lambda \gamma }{k}A\theta \left(1-\theta \right)\right\}}^{1/2}.$$

The coverage dependence of the chemical potential arises straightforwardly from Equations (37) and (169):

(172) $$K\left(\gamma ,k\right){\left(\frac{2}{\gamma }\right)}^{2(k-1)}\exp\left[\beta \left(\mu -k\epsilon_{0}-w\lambda /2\right)\right]=\frac{\theta }{k}\frac{{\left(1-\theta \right)}^{k(\gamma -1)}{\left[k-(k-1)\theta \right]}^{k-1}{\left[\frac{\lambda \theta }{2k}-\alpha \right]}^{\lambda /2}}{{\left[\frac{\gamma k}{2}-\left(k-1\right)\theta )\right]}^{k-1}{\left[\frac{\gamma }{2}\left(1-\theta \right)-\alpha \right]}^{k\gamma /2}{\left(\frac{\lambda \theta }{\gamma k}\right)}^{\lambda }}.$$

The configurational energy per site, *u*, can be calculated as

(173) $$\begin{array}{ccc} u & = & \frac{kN\epsilon_{0}}{M}+w\frac{N_{01}^{**}}{M}=\frac{kN\epsilon_{0}}{M}+w\left(\frac{\lambda N}{2M}-\frac{N_{01}^{**}}{2M}\right)\\ & = & \epsilon_{0}\theta +w\left(\frac{\lambda \theta }{2k}-\alpha \right). \end{array}$$

In addition, f=u−Ts and the entropy per site, *s*, can be obtained from Equations (169) and (173) as

(174) $$\begin{array}{ccc} \frac{s}{k_{B}} & = & \frac{\theta }{k}\ln K\left(\gamma ,k\right)+\left[\frac{\gamma }{2}-\left(\frac{k-1}{k}\right)\theta \right]\ln\left\{\frac{{\left[1-\left(\frac{k-1}{k}\right)\theta \right]}^{2/\gamma }{\left(1-\theta \right)}^{2(\gamma -1)/\gamma }\left[\frac{\gamma }{2}-\left(\frac{k-1}{k}\right)\theta \right]}{{\left[1-\frac{2}{\gamma }\left(\frac{k-1}{k}\right)\theta \right]}^{2}\left[\frac{\gamma }{2}\left(1-\theta \right)-\alpha \right]}\right\}\\ & & +\frac{\theta }{k}\ln\left\{\frac{{\left(\frac{\lambda \theta }{\gamma k}\right)}^{\lambda }{\left[\frac{\gamma }{2}\left(1-\theta \right)-\alpha \right]}^{\lambda /2}}{\frac{\theta }{k}{\left[1-\left(\frac{k-1}{k}\right)\theta \right]}^{(\lambda -\gamma )/\gamma }{\left(1-\theta \right)}^{(\lambda \gamma -\lambda )/\gamma }{\left[\frac{\lambda \theta }{2k}-\alpha \right]}^{\lambda /2}}\right\}\\ & & +2\alpha \ln\left\{\frac{{\left[\frac{\gamma }{2}\left(1-\theta \right)-\alpha \right]}^{1/2}{\left[\frac{\lambda \theta }{2k}-\alpha \right]}^{1/2}}{\alpha }\right\} \end{array}$$

#### General Expression of the Thermodynamic Functions in Terms of the Configurational Factor Ω(N,M,γ)

In Section 4.2, a theoretical model describing the adsorption of interacting polyatomic species was formulated based on the quasi-chemical approximation (QCA). This formulation was derived by integrating two key components: (i) the exact expression for the partition function of non-interacting linear *k*-mers in one dimension and its generalization to higher-dimensional systems, and (ii) an extended version of the classical QCA that accounts for adsorbates occupying multiple sites. In the present section, we broaden that framework to incorporate a variety of configurational terms which are relevant to the adsorption of non-interacting *k*-mers [31]. This extension enables a more flexible theoretical treatment, allowing different configurational entropy factors to be embedded directly into the model.

Let us start from Equation (166). Taking the logarithm and using Stirling’s approximation yields:

(175) $$\begin{array}{ccc} \ln Q(N,M,T) & = & -\beta N\left(\lambda w/2+k\epsilon_{0}\right)+\beta wN_{01}^{**}/2+\lambda N\ln\lambda N/\gamma \\ & & -\gamma B\ln B+\left(\gamma B/2\right)\ln\left(\gamma B/2\right)-N_{01}^{**}\ln N_{01}^{**}/2\\ & & +\gamma \left(B-\lambda N/\gamma \right)\ln\left(B-\lambda N/\gamma \right)\\ & & -\left(\gamma B/2-\lambda N/2-N_{01}^{**}/2\right)\ln\left(\gamma B/2-\lambda N/2-N_{01}^{**}/2\right)\\ & & -\left(\lambda N/2-N_{01}^{**}/2\right)\ln\left(\lambda N/2-N_{01}^{**}/2\right)+\ln\Omega \left(N,M\right). \end{array}$$

The Helmholtz free energy per site, f(N,M,T)=F(N,M,T)/M [βF(N,M,T)=−lnQ(N,M,T)], can be obtained from Equation (175) as a function of surface coverage, θ=kN/M, and temperature,

(176) $$\begin{array}{ccc} \beta f(\theta ,T) & = & \beta \frac{\theta }{k}\left(\frac{\lambda w}{2}+k\epsilon_{0}\right)-\lambda \frac{\theta }{k}\ln\frac{\lambda \theta }{\gamma k}+\left[\gamma -2\left(\frac{k-1}{k}\right)\theta \right]\ln\left[1-\frac{2}{\gamma }\left(\frac{k-1}{k}\right)\theta \right]\\ & & -\left[\frac{\gamma }{2}-\left(\frac{k-1}{k}\right)\theta \right]\ln\left[\frac{\gamma }{2}-\left(\frac{k-1}{k}\right)\theta \right]+\frac{\lambda \theta }{2k}\ln\left(\frac{\lambda \theta }{2k}-\alpha \right)\\ & & -c\left[1-\frac{2}{\gamma }\left(\frac{k-1}{k}\right)\theta -\frac{\lambda \theta }{\gamma k}\right]\ln\left[1-\frac{2}{\gamma }\left(\frac{k-1}{k}\right)\theta -\frac{\lambda \theta }{\gamma k}\right]\\ & & +\left[\frac{\gamma }{2}-\left(\frac{k-1}{k}\right)\theta -\frac{\lambda \theta }{2k}\right]\ln\left[\frac{\gamma }{2}-\left(\frac{k-1}{k}\right)\theta -\frac{\lambda \theta }{2k}-\alpha \right]\\ & & -\ln\Gamma \end{array}$$

where α and *b* can be obtained from Equations (170) and (171), respectively, and

(177) $$\Gamma =\Omega {\left(N,M\right)}^{1/M}.$$

The equilibrium properties of the adlayer can be obtained from Equation (175), along with the differential form of *F* in the canonical ensemble

(178) dF=−SdT−ΠdM+μdN

where *S*, Π, and μ represent the entropy, the spreading pressure, and the chemical potential, respectively.

The coverage dependence of the chemical potential, μ $\left[={\left(\partial F/\partial N\right)}_{M,T}\right]$, arises in a straightforward manner from Equations (175) and (178)

(179) $$\begin{array}{ccc} \beta \mu & = & \beta \left(\frac{\lambda w}{2}+k\epsilon_{0}\right)-\lambda \ln\frac{\lambda \theta }{\gamma k}-2\left(k-1\right)\ln\left(1-\frac{2\theta }{\gamma }+\frac{2\theta }{\gamma k}\right)\\ & & +\left(k-1\right)\ln\left(\frac{\gamma }{2}-\theta +\frac{\theta }{k}\right)+\gamma \;k\ln\left(1-\theta \right)-\frac{\gamma \;k}{2}\ln\left[\frac{\gamma }{2}\left(1-\theta \right)-\alpha \right]\\ & & +\frac{\lambda }{2}\ln\left(\frac{\lambda \theta }{2k}-\alpha \right)-k\frac{\partial \ln\Gamma }{\partial \theta }. \end{array}$$

The resulting expressions allow for the inclusion of any configurational factor associated with the entropy of adsorbed non-interacting *k*-mers, such as the Flory–Huggins term [6,7], the Guggenheim–DiMarzio approach [9,10], the FSTA approximation [12,13], or other semi-empirical corrections [11]. Consequently, the main thermodynamic quantities can be explicitly expressed in terms of Ω or Γ, depending on the specific configurational framework employed. This generalized model is thus capable of describing adsorption processes involving molecules of arbitrary geometry and size.

### 4.3. Quasi-Chemical Approximation for Interacting Mixtures Adsorbed onto 2D Substrates

In this section, we introduce a theoretical framework to describe the adsorption behaviors of interacting binary mixtures composed of polyatomic molecules [113]. This approach combines two essential components: (i) the exact partition function for mixtures of non-interacting polyatomic species adsorbed in one-dimensional systems and its extension to higher-dimensional lattices, and (ii) a generalized version of the QCA that accounts for binary mixtures where the adsorbed entities occupy multiple adjacent sites; specifically, *k*-mers (covering *k* lattice positions) and *l*-mers (covering *l* sites).

**Figure 5:**
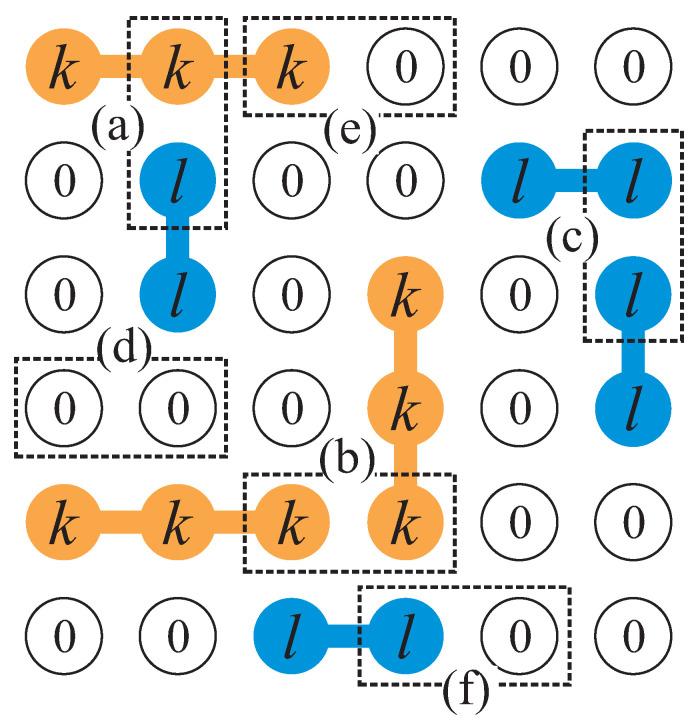
Schematic illustration of a lattice model featuring adsorbed dimers (l=2, blue circles) and trimers (k=3, orange circles) on a square lattice with connectivity γ=4. The image depicts various types of neighboring site pairs: (**a**) kl (orange–blue contact), (**b**) kk, (**c**) ll, (**d**) 00 (vacant–vacant), (**e**) k0 (occupied by *k*-mer and adjacent to empty), and (**f**) l0 (occupied by *l*-mer and adjacent to empty).

We consider a surface represented by a regular lattice with coordination number γ. The adsorbing gas phase consists of two types of linear molecules: *k*-mers and *l*-mers, which occupy *k* and *l* contiguous lattice sites, respectively, upon adsorption. The energetic contributions to the adsorption process include: (1) a constant site-binding energy $\epsilon_{k}$ for each unit of a *k*-mer and $\epsilon_{l}$ for each unit of an *l*-mer, and (2) lateral interaction energies $w_{kl}$ between neighboring units from different species (i.e., a *k*-mer adjacent to an *l*-mer) and, similarly, $w_{kk}$ and $w_{ll}$ for interactions between like species. The number of neighboring pairs corresponding to each interaction type is denoted as follows: $N_{kl}$ for contacts between *k*-mers and *l*-mers, $N_{kk}$ for contacts between two *k*-mers, and $N_{ll}$ for contacts between two *l*-mers. These quantities represent the number of nearest-neighbor site pairs where the respective interactions occur (see Figure 5).

The total energy of the system when $N_{k}$ *k*-mers and $N_{l}$ *l*-mers are adsorbed, with $N_{kk}$, $N_{ll}$, and $N_{kl}$ nearest-neighbor pairs, is given by

(180) $$E\left(N_{k},N_{l},N_{kk},N_{ll},N_{kl}\right)=N_{k}k\epsilon_{k}+N_{l}l\epsilon_{l}+N_{kk}w_{kk}+N_{ll}w_{ll}+N_{kl}w_{kl}.$$

The canonical partition function for a two-dimensional system can be written as

(181) $$\begin{array}{cc} Q= & \sum_{N_{kk}}\sum_{N_{ll}}\sum_{N_{kl}}g\left(N_{k},N_{l};N_{kk},N_{ll},N_{kl};M\right)\\ & \times \exp[-\beta \left(N_{k}k\epsilon_{k}+N_{l}l\epsilon_{l}+N_{kk}w_{kk}+N_{ll}w_{ll}+N_{kl}w_{kl}\right)], \end{array}$$

where $N_{k}$ ($N_{l}$) is the number of molecules of species *k* (*l*) adsorbed onto the surface, and $g(N_{k},N_{l};N_{kk},N_{ll},N_{kl};M)$ is the number of ways to arrange $N_{k}$ *k*-mers and $N_{l}$ *l*-mers on *M* sites while maintaining $N_{kk}$, $N_{ll}$, and $N_{kl}$ pairs of occupied sites.

In a similar way to the QCA for only one species (Section 4.2), here we calculate the expressions relating $N_{kk}$, $N_{ll}$, $N_{kl}$, $N_{k0}$, $N_{l0}$, and $N_{00}$:

(182) $$\gamma kN_{k}=2N_{kk}+N_{kl}+N_{0k}+2\left(k-1\right)N_{k},$$

(183) $$\gamma lN_{l}=2N_{ll}+N_{kl}+N_{0l}+2\left(l-1\right)N_{l},$$

(184) $$\gamma \left(M-kN_{k}-lN_{l}\right)=2N_{00}+N_{0k}+N_{0l}.$$

The number of total pairs is

(185) $$\mathrm{Number}\;\mathrm{of}\;\mathrm{total}\;\mathrm{pairs}=\frac{\gamma M}{2}-N_{k}\left(k-1\right)-N_{l}\left(l-1\right),$$

where “number of 0k pairs” = “number of k0 pairs” = $N_{0k}/2$ (the same for the number of l0 and kl pairs).

The number of ways of assigning a total of $\frac{\gamma M}{2}-N_{k}\left(k-1\right)-N_{l}\left(l-1\right)$ independent pairs to the nine categories 0k,k0,0l,l0,kl,lk,kk,ll, and 00 is

(186) $$\begin{array}{cc} \; & \tilde{g}\left(N_{k},N_{l};N_{kk},N_{ll},N_{kl},N_{0k},N_{0l},N_{00};M\right)=\\ & \frac{[\frac{\gamma M}{2}-N_{k}\left(k-1\right)-N_{l}\left(l-1\right)]!}{{\left[\left(\frac{N_{0k}}{2}\right)!\right]}^{2}{\left[\left(\frac{N_{0l}}{2}\right)!\right]}^{2}{\left[\left(\frac{N_{kl}}{2}\right)!\right]}^{2}N_{kk}!N_{ll}!N_{00}!}. \end{array}$$

Taking the logarithm in Equation (186), using the Stirling’s approximation, and rearranging, we obtain

(187) $$\begin{array}{cc} \; & \ln\tilde{g}\left(N_{k},N_{l};N_{kk},N_{ll},N_{kl},N_{0k},N_{0l},N_{00};M\right)=\\ & \left[\frac{\gamma M}{2}-N_{k}\left(k-1\right)-N_{l}\left(l-1\right)\right]\ln\left[\frac{\gamma M}{2}-N_{k}\left(k-1\right)-N_{l}\left(l-1\right)\right]\\ & -N_{0l}\ln\frac{N_{0l}}{2}-N_{0k}\ln\frac{N_{0k}}{2}-N_{lk}\ln\frac{N_{lk}}{2}-N_{kk}\ln N_{kk}-N_{ll}\ln N_{ll}\\ & -N_{00}\ln N_{00}. \end{array}$$

It is convenient to write $\tilde{g}$ as a function of $N_{kk}$, $N_{ll}$, and $N_{lk}$. For this purpose, we obtain $N_{0k}$, $N_{0l}$, and $N_{00}$ in terms of $N_{kk}$, $N_{ll}$, and $N_{kl}$ [using Equations (182–184)], and replace them in Equation (187). Then, we have

(188) $$\begin{array}{cc} \; & \ln\tilde{g}\left(N_{k},N_{l};N_{kk},N_{ll},N_{lk};M\right)=\\ & \left[\frac{\gamma M}{2}-N_{k}\left(k-1\right)-N_{l}\left(l-1\right)\right]\times \ln\left[\frac{\gamma M}{2}-N_{k}\left(k-1\right)-N_{l}\left(l-1\right)\right]\\ & -\left[\gamma lN_{l}-2N_{ll}-N_{lk}-2\left(l-1\right)N_{l}\right]\times \ln\left\{\frac{1}{2}\left[\gamma lN_{l}-2N_{ll}-N_{lk}-2\left(l-1\right)N_{l}\right]\right\}\\ & -\left[\gamma kN_{k}-2N_{kk}-N_{rk}-2\left(k-1\right)N_{k}\right]\times \ln\left\{\frac{1}{2}\left[\gamma kN_{k}-2N_{kk}-N_{rk}-2\left(k-1\right)N_{k}\right]\right\}\\ & -N_{lk}\ln\frac{N_{rk}}{2}-N_{kk}\ln N_{kk}-N_{ll}\ln N_{ll}\\ & -\left[\frac{\gamma M}{2}+N_{k}\left(k-\gamma k-1\right)+N_{l}\left(l-\gamma l-1\right)+N_{ll}+N_{lk}+N_{kk}\right]\\ & \times \ln\left[\frac{\gamma M}{2}+N_{k}\left(k-\gamma k-1\right)+N_{l}\left(l-\gamma l-1\right)+N_{ll}+N_{lk}+N_{kk}\right]. \end{array}$$

$\tilde{g}\left(N_{k},N_{l};N_{kk},N_{ll},N_{lk};M\right)$ cannot be set equal to $g(N_{k},N_{l};N_{kk},N_{ll},N_{kl};M)$ in Equation (181), because treating the pairs as independent entities leads to some non-physical configurations (see Ref. [80]). To take care of this, we must normalize $\tilde{g}$ with a proportionality constant $C(N_{k},N_{l},M)$

(189) $$g\left(N_{k},N_{l};N_{kk},N_{ll},N_{kl};M\right)=C\left(N_{k},N_{l},M\right)\tilde{g}\left(N_{k},N_{l};N_{kk},N_{ll},N_{kl};M\right),$$

and

(190) $$\begin{array}{cc} \Omega_{\gamma }\left(N_{k},N_{l},M\right) & =\sum_{N_{kk}}\sum_{N_{ll}}\sum_{N_{kl}}g\left(N_{k},N_{l};N_{kk},N_{ll},N_{kl};M\right)\\ & =C\left(N_{k},N_{l},M\right)\sum_{N_{kk}}\sum_{N_{ll}}\sum_{N_{kl}}\tilde{g}\left(N_{k},N_{l};N_{kk},N_{ll},N_{kl};M\right), \end{array}$$

where $\Omega_{\gamma }\left(N_{k},N_{l},M\right)$ is the number of ways to arrange $N_{k}$ *k*-mers and $N_{l}$ *l*-mers on a lattice of *M* sites with connectivity γ.

For a one-dimensional lattice (γ=2), $\Omega_{\gamma =2}\left(N_{k},N_{l},M\right)$ can be exactly calculated as the total number of permutations of the $N_{k}$ indistinguishable *k*-mers and $N_{l}$ indistinguishable *l*-mers out of $n_{e}$ entities, being

(191) $$\begin{array}{ccc} n_{e} & = & \mathrm{number}\;\mathrm{of}\;k-\mathrm{mers}\;+\mathrm{number}\;\mathrm{of}\;l-\mathrm{mers}\;+\;\mathrm{number}\;\mathrm{of}\;\mathrm{empty}\;\mathrm{sites}\\ & = & N_{k}+N_{l}+M-kN_{k}-lN_{l}=M-\left(k-1\right)N_{k}-\left(l-1\right)N_{l}. \end{array}$$

Accordingly,

(192) $$\Omega_{\gamma =2}\left(N_{k},N_{l},M\right)=\frac{\left[M-\left(k-1\right)N_{k}-\left(l-1\right)N_{l}\right]!}{N_{k}!N_{l}!\left(M-kN_{k}-lN_{l}\right)!}.$$

In general, there is no exact expression for $\Omega_{\gamma }\left(N_{k},N_{l},M\right)$ in two (or more) dimensions (even in the simplest case of single dimers on *M* sites, there does not exist an exact form of $\Omega_{\gamma }$ in two or more dimensions). However, $\Omega_{\gamma }\left(N_{k},N_{l},M\right)$ can be accurately approximated by applying the arguments presented in the previous sections, which relate the configurational factor $\Omega_{\gamma }\left(N_{k},N_{l},M\right)$ for any γ, with the same quantity in one dimension (γ=2). Thus,

(193) $$\Omega_{\gamma }\left(N_{k},N_{l},M\right)\approx {\left[K(\gamma ,k)\right]}^{N_{k}}{\left[L(\gamma ,l)\right]}^{N_{l}}\Omega_{\gamma =2}\left(N_{k},N_{l},M\right),$$

where K(γ,k) [L(γ,l)] represents the number of available configurations (per lattice site) for a *k*-mer [*l*-mer] at zero coverage. K(γ,k) [L(γ,l)] is, in general, a function of the connectivity and the size of the adsorbate.

The terms K(γ,k) and L(γ,l) take into account the degrees of freedom of the adsorbed particles on a lattice of connectivity γ. Thus, in the particular case of straight rigid adsorbates, it follows that K(γ,k)=L(γ,l)=γ/2 (k≥2). This scheme has been successfully used by many researchers [6,8,11,16,20,33,34,93,114], and will be used in this paper as well.

Once $\Omega_{\gamma }\left(N_{k},N_{l},M\right)$ is obtained from Equations (192) and (193)—as is usual in the quasi-chemical formalism—$C(N_{k},N_{l},M)$ can be calculated using the maximum-term method [80] in Equation (190). The method allows us to replace $\sum_{N_{kk}}\sum_{N_{ll}}\sum_{N_{kl}}\tilde{g}(N_{k},N_{l};N_{kk},$$N_{ll},N_{kl};M)$ with the maximum term in the sum, $\tilde{g}\left(N_{k},N_{l};N_{kk}^{*},N_{ll}^{*},N_{kl}^{*};M\right)$. From the condition $\nabla \ln\tilde{g}\left(N_{kk},N_{ll},N_{lk}\right)=0$, we obtain

(194) $$\begin{array}{cc} \frac{\partial \ln\tilde{g}\left(N_{kk},N_{ll},N_{lk}\right)}{\partial N_{kk}}= & 2\ln\left\{\frac{1}{2}\left[\gamma kN_{k}-2N_{kk}-N_{lk}-2\left(k-1\right)N_{k}\right]\right\}-\ln N_{kk}\\ & -\ln\left[\frac{\gamma M}{2}+N_{k}\left(k-1-\gamma k\right)+N_{l}\left(l-1-\gamma l\right)+N_{ll}+N_{kk}+N_{lk}\right]=0, \end{array}$$

(195) $$\begin{array}{cc} \frac{\partial \ln\tilde{g}\left(N_{kk},N_{ll},N_{lk}\right)}{\partial N_{ll}}= & 2\ln\left\{\frac{1}{2}\left[\gamma lN_{l}-2N_{ll}-N_{lk}-2\left(l-1\right)N_{l}\right]\right\}-\ln N_{ll}\\ & -\ln\left[\frac{\gamma M}{2}+N_{k}\left(k-1-\gamma l\right)+N_{l}\left(l-1-\gamma l\right)+N_{ll}+N_{kk}+N_{lk}\right]=0, \end{array}$$

(196) $$\begin{array}{cc} \frac{\partial \ln\tilde{g}\left(N_{kk},N_{ll},N_{lk}\right)}{\partial N_{lk}}= & \ln\left\{\frac{1}{2}\left[\gamma lN_{l}-2N_{ll}-N_{lk}-2\left(l-1\right)N_{l}\right]\right\}-\ln\frac{N_{lk}}{2}+\ln\left\{\frac{1}{2}\left[\gamma kN_{k}-2N_{kk}-N_{lk}-2\left(k-1\right)N_{k}\right]\right\}\\ & -\ln\left[\frac{\gamma M}{2}+N_{k}\left(k-1-\gamma k\right)+N_{l}\left(l-1-\gamma l\right)+N_{ll}+N_{lk}+N_{kk}\right]=0, \end{array}$$

and the corresponding values of $N_{kk}$, $N_{ll}$, and $N_{kl}$ giving the maximum term in the sum in Equation (190) can be obtained by solving the equations

(197) $$N_{kk}^{*}=\frac{{\left\{\left[\left(\gamma -2\right)k+2\right]N_{k}-2N_{kk}^{*}-N_{kl}^{*}\right\}}^{2}}{4\left\{\gamma M/2+\left[\left(1-\gamma \right)k-1\right]N_{k}+\left[\left(1-\gamma \right)l-1\right]N_{l}+N_{kk}^{*}+N_{ll}^{*}+N_{kl}^{*}\right\}},$$

(198) $$N_{ll}^{*}=\frac{{\left\{\left[\left(\gamma -2\right)l+2\right]N_{l}-2N_{ll}^{*}-N_{kl}^{*}\right\}}^{2}}{4\left\{\gamma M/2+\left[\left(1-\gamma \right)k-1\right]N_{k}+\left[\left(1-\gamma \right)l-1\right]N_{l}+N_{kk}^{*}+N_{ll}^{*}+N_{kl}^{*}\right\}},$$

(199) $$N_{lk}^{*}=\frac{\left\{\left[\left(\gamma -2\right)k+2\right]N_{k}-2N_{kk}^{*}-N_{kl}^{*}\right\}\left\{\left[\left(\gamma -2\right)l+2\right]N_{l}-2N_{ll}^{*}-N_{kl}^{*}\right\}}{2\left\{\gamma M/2+\left[\left(1-\gamma \right)k-1\right]N_{k}+\left[\left(1-\gamma \right)l-1\right]N_{l}+N_{kk}^{*}+N_{ll}^{*}+N_{kl}^{*}\right\}}.$$

Then, from Equations (190) and (197)–(199), and using simple algebra, we have that $C(N_{k},N_{l},M)$ and, consequently, $g(N_{k},N_{l};N_{kk},N_{ll},N_{kl};M)$ can be calculated as follows:

(200) $$\begin{array}{ccc} C(N_{k},N_{l},M) & = & \frac{\Omega (N_{k},N_{l},M)}{\sum_{N_{kk}}\sum_{N_{ll}}\sum_{N_{kl}}\tilde{g}\left(N_{k},N_{l};N_{kk},N_{ll},N_{kl};M\right)}\\ & = & \frac{\Omega (N_{k},N_{l},M)}{\tilde{g}\left(N_{k},N_{l};N_{kk}^{*},N_{ll}^{*},N_{kl}^{*};M\right)}. \end{array}$$

Then,

(201) $$g\left(N_{k},N_{l};N_{kk},N_{ll},N_{kl};M\right)=\frac{\Omega \left(N_{k},N_{l},M\right)\tilde{g}\left(N_{k},N_{l};N_{kk},N_{ll},N_{kl};M\right)}{\tilde{g}\left(N_{k},N_{l};N_{kk}^{*},N_{ll}^{*},N_{kl}^{*};M\right)}.$$

Now, the partition function can be written as

(202) $$Q=\frac{\Omega (N_{k},N_{l},M)}{\tilde{g}\left(N_{k},N_{l};N_{kk}^{*},N_{ll}^{*},N_{kl}^{*};M\right)}\sum_{N_{kk}}\sum_{N_{ll}}\sum_{N_{kl}}\tilde{g}\left(N_{k},N_{l};N_{kk},N_{ll},N_{kl};M\right)e^{-\beta E}.$$

The sum in Equation (202) can be solved by applying the maximum-term method again. Thus, $\sum_{N_{kk}}\sum_{N_{ll}}\sum_{N_{kl}}\tilde{g}\left(N_{k},N_{l};N_{kk},N_{ll},N_{kl};M\right)e^{-\beta (N_{k}k\epsilon_{k}+N_{l}l\epsilon_{l}+N_{kk}w_{kk}+N_{ll}w_{ll}+N_{kl}w_{kl})}$ can be replaced by $\tilde{g}\left(N_{k},N_{l};N_{kk}^{**},N_{ll}^{**},N_{kl}^{**};M\right)e^{-\beta (N_{k}k\epsilon_{k}+N_{l}l\epsilon_{l}+N_{kk}^{**}w_{kk}+N_{ll}^{**}w_{ll}+N_{kl}^{**}w_{kl})}$. The corresponding values of $N_{kk}^{**}$, $N_{ll}^{**}$ and $N_{kl}^{**}$ are obtained by solving the equations

(203) $$N_{kk}^{**}e^{\beta w_{kk}}=\frac{{\left\{\left[\left(\gamma -2\right)k+2\right]N_{k}-2N_{kk}^{**}-N_{kl}^{**}\right\}}^{2}}{4\left\{\gamma M/2+\left[\left(1-\gamma \right)k-1\right]N_{k}+\left[\left(1-\gamma \right)l-1\right]N_{l}+N_{kk}^{**}+N_{ll}^{**}+N_{kl}^{**}\right\}},$$

(204) $$N_{ll}^{**}e^{\beta w_{ll}}=\frac{{\left\{\left[\left(\gamma -2\right)l+2\right]N_{l}-2N_{ll}^{**}-N_{kl}^{**}\right\}}^{2}}{4\left\{\gamma M/2+\left[\left(1-\gamma \right)k-1\right]N_{k}+\left[\left(1-\gamma \right)l-1\right]N_{l}+N_{kk}^{**}+N_{ll}^{**}+N_{kl}^{**}\right\}},$$

(205) $$N_{lk}^{**}e^{\beta w_{lk}}=\frac{\left\{\left[\left(\gamma -2\right)k+2\right]N_{k}-2N_{kk}^{**}-N_{kl}^{**}\right\}\left\{\left[\left(\gamma -2\right)l+2\right]N_{l}-2N_{ll}^{**}-N_{kl}^{**}\right\}}{2\left\{\gamma M/2+\left[\left(1-\gamma \right)k-1\right]N_{k}+\left[\left(1-\gamma \right)l-1\right]N_{l}+N_{kk}^{**}+N_{ll}^{**}+N_{kl}^{**}\right\}}.$$

In a similar way as done with the sum in Equation (190), the expressions (203)–(205) were obtained via the process of differentiating and equating the term in the sum in Equation (202) to zero. Finally,

(206) $$\begin{array}{ccc} Q & = & \frac{\Omega \left(N_{k},N_{l},M\right)\tilde{g}\left(N_{k},N_{l};N_{kk}^{**},N_{ll}^{**},N_{kl}^{**};M\right)}{\tilde{g}\left(N_{k},N_{l};N_{kk}^{*},N_{ll}^{*},N_{kl}^{*};M\right)}\\ & & \times \exp\left[-\beta (N_{k}k\epsilon_{k}+N_{l}l\epsilon_{l}+N_{kk}^{**}w_{kk}+N_{ll}^{**}w_{ll}+N_{kl}^{**}w_{kl})\right]. \end{array}$$

The chemical potential of each adsorbed species can be calculated from the free energy F=−lnQ,

(207) $$\beta \mu_{k,ads}={\left(\frac{\partial \beta F}{\partial N_{k}}\right)}_{N_{l},M,T}=k{\left(\frac{\partial \beta f}{\partial \theta_{k}}\right)}_{\theta_{l},T},$$

and

(208) $$\beta \mu_{l,ads}={\left(\frac{\partial \beta F}{\partial N_{l}}\right)}_{N_{k},M,T}=l{\left(\frac{\partial \beta f}{\partial \theta_{l}}\right)}_{\theta_{k},T},$$

where f=F/M and $\theta_{x}=xN_{x}/M$ (x=k,l).

On the other hand, the chemical potential of each kind of molecule in an ideal gas mixture, at temperature *T* and pressure *P*, is given as

(209) $$\beta \mu_{x,gas}=\beta \mu_{x}^{0}+\ln X_{x}P,\;\;\;\;\left\{x=k,l\right\},$$

where $X_{x}$ is the mole fraction and $\mu_{x}^{0}$ is the standard chemical potential of the *x*-mer.

At equilibrium, the chemical potential of the adsorbed and gas phase are equal; that is, $\mu_{x,ads}=\mu_{x,gas}$. Then,

(210) $$\beta \mu_{k}^{0}+\ln X_{k}P=k{\left(\frac{\partial \beta f}{\partial \theta_{k}}\right)}_{\theta_{l},T},$$

and

(211) $$\beta \mu_{l}^{0}+\ln X_{l}P=l{\left(\frac{\partial \beta f}{\partial \theta_{l}}\right)}_{\theta_{k},T}.$$

The theoretical procedure described in this section can be summarized as follows:

(1) Given the complete set of lateral interactions and temperature, the values of $N_{kk}^{*}$, $N_{ll}^{*}$, and $N_{kl}^{*}$ are obtained by solving Equations (197)–(199).
(2) Once $N_{kk}^{*}$, $N_{ll}^{*}$, and $N_{kl}^{*}$ are calculated, $C(N_{k},N_{l},M)$ and $g(N_{k},N_{l};N_{kk},N_{ll},N_{kl};M)$ can be obtained [see Equations (200) and (201)], and the partition function can be written as in Equation (202).
(3) The partition function *Q* is calculated using the maximum-term method. For this purpose, $N_{kk}^{**}$, $N_{ll}^{**}$, and $N_{kl}^{**}$ are obtained by solving Equations (203)–(205), and are introduced into Equation (206).
(4) f=−(lnQ)/M is calculated, and the partial adsorption isotherms of the system are obtained from Equations (210) and (211).

Steps (3) and (4) are numerically (and simultaneously) solved through a standard computing procedure.

To conclude Section 4, we would like to note that our study of lateral interactions focused primarily on the classical BWA and QCA, as these are more closely related to the research carried out by our group. However, we must refer to other developments that are refinements or extensions of mean-field approximations to this problem. Gujrati emphasized that while these methods account for local correlations, they fail to capture longer-range interactions. As a result, the quasi-chemical approach is insufficient to correctly describe the thermodynamics and phase transitions of complex systems [85].

To address these deficiencies, Gujrati proposed a more robust framework based on recursive lattice methods, such as those using Bethe lattices. He demonstrated that these models successfully incorporate local correlations beyond nearest-neighbors and overcome the intrinsic limitations of traditional mean-field theories. In particular, the bulk free energy can be calculated exactly via fixed-point recursive equations, significantly improving the reliability of theoretical predictions [84,85,86]. These contributions have profoundly reshaped the boundaries of validity and applicability for conventional polymer models, underscoring the need for more accurate methodologies—such as recursive lattice techniques—for the reliable study of phase transitions in polymer solutions and lattice gases [68,69,87,115,116,117]. For branched polymers, this approximation has proven particularly effective in modeling collapse transitions and solvation phenomena, where mean-field approaches often fail due to oversimplified representations of polymer architecture [118,119]. Moreover, the Bethe lattice method has been successfully applied to inhomogeneous systems, such as polymers near interfaces or confined geometries, where it accurately captures local structural variations and correlations [86].

## 5. Latest Developments, Part I: Multiple Exclusion Statistics for Spatially Correlated Single Species

It has recently been proposed that the complex problem of interacting particles with arbitrary size and shape can be addressed using concepts from fractional statistics, extending them to classical systems of particles with spatially correlated states [12]. This approach reframes the counting of allowed configurations in terms of exclusion principles that go beyond Pauli-type constraints, providing a new route to approximate thermodynamic functions in structured lattice gases.

We briefly revisit the foundational arguments and structure of the formalism for a single particle species in a homogeneous external field. The generalization to mixtures of species is presented in the following section of this review (Section 6).

### 5.1. Multiple Exclusion Statistics Formalism

We consider the equilibrium of identical particles distributed over a set of states within a volume *V*. These states are, in general, spatially correlated—meaning that the allowed equilibrium positions (i.e., the state spectrum) are distributed over space with correlation lengths smaller than the size of a particle (typical examples include lattice gases with excluded volume interactions, where particles occupy multiple sites depending on their shape and size). As a result, a particle occupying a particular state not only excludes that state, but also prevents occupation of additional states due to the spatial extent of the particle relative to the distribution of states.

In systems where each particle excludes a constant number *g* of states, this process has a classical analogy to the quantum exclusion principle, as introduced in Ref. [14]. However, due to the spatial arrangement of states (or the lattice topology, in the case of lattice systems), multiple particles may simultaneously exclude the same state. This occurs, for example, in regular lattices with structured particles spanning more than one site. We refer to this statistical phenomenon as multiple exclusion (ME) of states, which significantly influences the entropy and thermodynamic behavior of the system.

Given that the mean exclusion (ME) is intrinsically tied to the spatial arrangement of particles, we propose an approximate formulation for the partition function based on a state-counting ansatz. This approach incorporates the configurational dependence of ME while remaining suitable for both analytical treatment and numerical evaluation of thermodynamic properties. When the statistical exclusion is configuration-independent and uniform, the ME framework simplifies to the fractional statistics formalism originally introduced by Wu [15]. Our aim is to construct a general thermodynamic model which is capable of describing lattice systems composed of structured particles, considering exclusion effects arising from particle geometry. This model is then extended to heterogeneous systems involving mixtures of hard-core particles of arbitrary size and shape occupying discrete lattice sites.

We consider a system of *N* indistinguishable particles that can occupy *G* accessible single-particle states within a volume *V* at temperature *T*, with each particle possessing an energy level $\epsilon_{0}$. The canonical partition function is written as $Q\left(N,V,T\right)=\sum_{i}e^{-\beta H_{i}\left(N\right)}=W\left(N\right)\;e^{-\beta N\epsilon_{0}}\;q_{i}^{N}$, where $H_{i}\left(N\right)$ is the Hamiltonian associated with the *i*th configuration, $\beta =1/(k_{B}T)$, W(N) represents the total number of accessible microstates for *N* particles, and $q_{i}$ denotes the internal partition function of a single particle.

In systems where particles exclude certain configurations due to geometric constraints or interactions, such that the *G* states are not mutually independent, the total number of configurations can be approximated by $W\left(N\right)=\frac{(d_{N}+N-1)!}{N!(d_{N}-1)!}$, where $d_{N}$ represents the number of available states for the *N*th particle after placing the previous N−1 particles in the system. This form becomes exact when the available states are independent and the exclusion imposed by each particle is uniform, as originally derived in Ref. [15]. In the more general case involving correlations between states, the expression provides a reasonable approximation, with $d_{N}$ varying based on configuration.

To evaluate $d_{N}$, a statistical ansatz is introduced. In the thermodynamic limit, this leads to a well-defined density of accessible states per particle, expressed as $\tilde{d}\left(n\right)=\lim_{N,G\to \infty ,\;N/G\to n}d_{N}/G$, where $n=\lim_{N,G\to \infty }\frac{N}{G}$ denotes the occupation number.

Applying Stirling’s approximation lnx!≈xlnx−x, the Helmholtz free energy $F\left(N,T,V\right)=-k_{B}T\ln Q=N\epsilon_{0}-TS\left(N,V,T\right)$ leads to the intensive functions per state $\tilde{F}\left(n,T\right)=\lim_{N,G\to \infty }F\left(N,T,V\right)/G$ and $\tilde{S}\left(n,T\right)=\lim_{N,G\to \infty }S\left(N,T,V\right)/G$, with

(212) $$\beta \tilde{F}\left(n,T\right)=\beta \epsilon_{0}\;n+\tilde{d}\left(n\right)\ln\tilde{d}\left(n\right)+n\ln n-\left[\tilde{d}\left(n\right)+n\right]\ln\left[\tilde{d}\left(n\right)+n\right],$$

(213) $$\frac{\tilde{S}\left(n,T\right)}{k_{B}}=\left[\tilde{d}\left(n\right)+n\right]\ln\left[\tilde{d}\left(n\right)+n\right]-\tilde{d}\left(n\right)\ln\tilde{d}\left(n\right)-n\ln n.$$

The chemical potential $\beta \mu \left(n,T\right)={\left(\partial \beta \tilde{F}/\partial n\right)}_{T}$ takes the form

(214) $$\beta \mu \left(n,T\right)=\beta \epsilon_{0}+\ln n+\tilde{d^{\prime }}\left(n\right)\ln\tilde{d}\left(n\right)-\left[\tilde{d^{\prime }}\left(n\right)+1\right]\ln\left[\tilde{d}\left(n\right)+n\right]$$

or, alternatively, as a ratio between occupied and unoccupied states:

(215) $$K\left(T\right)\;e^{\beta \mu }=\frac{n\;{\left[\tilde{d}\left(n\right)\right]}^{\tilde{d^{\prime }}\left(n\right)}}{{\left[\tilde{d}\left(n\right)+n\right]}^{\tilde{d^{\prime }}\left(n\right)+1}}=\frac{n}{P_{o}\left(n\right)},$$

where $\tilde{d^{\prime }}\left(n\right)=d\tilde{d}\left(n\right)/dn$, $K\left(T\right)=e^{-\beta \epsilon_{0}}q_{i}$, and $P_{o}\left(n\right)$ is the fraction of unoccupied states. For simplicity, we assume $q_{i}=1$.

If the system exchanges particles with a reservoir at μ and *T*, the time evolution of the occupation number is $\frac{dn}{dt}=P_{o}W_{o\to •}-P_{•}W_{•\to o}$, where $P_{o}$ and $P_{•}$ are the probabilities of a state being empty or occupied, and $W_{o\to •}$, $W_{•\to o}$ are the transition rates.

At equilibrium, $\frac{dn}{dt}=0$, which implies

(216) $$\frac{W_{o\to •}}{W_{•\to o}}=\frac{P_{•}}{P_{o}}=e^{\beta (\mu -\epsilon_{0})}.$$

As $P_{•}=n$, Equation (215) yields the expression

(217) $$P_{o}\left(n\right)=\frac{{\left[\tilde{d}\left(n\right)+n\right]}^{\tilde{d^{\prime }}\left(n\right)+1}}{{\left[\tilde{d}\left(n\right)\right]}^{\tilde{d^{\prime }}\left(n\right)}},$$

which is approximate as long as $\tilde{d}\left(n\right)$ is not exact.

We now define the exclusion spectrum function [17]:

(218) $$G\left(n\right)=\frac{1-P_{o}\left(n\right)}{n},$$

which gives the average number of excluded states per particle at occupation *n*. Note that G(n) includes the occupied state itself.

Additionally, the exclusion density function e(n) is defined through the excluded fraction $E\left(n\right)=1-P_{o}\left(n\right)$, such that e(n)=dE(n)/dn. Then, e(n) measures the number of states excluded by inserting a particle at occupation *n*.

The normalization $E\left(n_{m}\right)=\int_{0}^{n_{m}}e\left(n^{\prime }\right)dn^{\prime }=1$ defines $n_{m}=1/g$, where *g* is the number of states excluded per particle at saturation. Thus, $e\left(0\right)=f_{0}$ corresponds to the exclusion caused by a single isolated particle and $e(n_{m})=0$.

From Equations (215) and (218), we obtain:

(219) $$G\left(n\right)=\frac{E(n)}{n}=\frac{1}{n}-\frac{1}{e^{\beta (\mu -\epsilon_{0})}},$$

(220) $$e\left(n\right)=G\left(n\right)+n\;G^{\prime }\left(n\right)=\frac{n\beta \mu^{\prime }-1}{e^{\beta (\mu -\epsilon_{0})}},$$

where $\mu^{\prime }\left(n\right)=d\mu \left(n\right)/dn$. These spectral functions provide a route to obtain detailed statistical information about the exclusion spectrum from thermodynamic observables such as the adsorption isotherm βμ(n), as will be further explored in Section 5.7. All quantities are expressed in terms of the occupation number *n*, which facilitates interpretation in this framework. They can be converted to lattice coverage θ through the transformation θ=ng.

### 5.2. States Counting Ansatz: Density of States

Determining the exact number of configurations for systems of this kind remains one of the longstanding challenges in statistical mechanics and represents a complex analytical problem. In this work, we propose a self-consistent thermodynamic approximation rooted in a novel form of ME statistics, inspired by concepts from fractional statistics [14,15]. This framework is applied to classical systems involving a single species with spatially correlated configurations; for example, structured particles adsorbed on regular lattices. We derive a general approximation for the density of accessible states, denoted as $\tilde{d}\left(n\right)$, which incorporates the effects of spatial correlations in the occupation of available sites. When applied to the classical lattice–gas problem of linear *k*-mers adsorbed on square lattices, the resulting ME formalism yields predictions that align closely with MC simulation results. The methodology has also been extended to cover systems of adsorbed squares and rectangles on square lattices [18]. Further developments, including its extension to multicomponent systems and a more detailed description of *k*-mer phase transitions, have been discussed in [19] and are briefly summarized in the following section.

All exclusion-related and thermodynamic functions introduced in Section 5.1 depend fundamentally on the density of states. We now outline the proposed approximation for $\tilde{d}\left(n\right)$.

The exclusion-based state-counting strategy for a single species is constructed as follows. Let *G* denote the total number of single-particle states available within a system of volume *V*. As *N* indistinguishable particles are added one by one, each new particle occupies a state while simultaneously rendering some other states inaccessible due to geometric or spatial constraints. Notably, the number of states excluded by each added particle is not constant; instead, it evolves with *N* due to the presence of correlations in the system. This gives rise to the concept of multiple exclusion (ME), as introduced earlier.

The following recursion relations define the number of available states for the *N*th particle: $d_{1}=G$, $d_{2}=d_{1}-N_{1}$, …, $d_{N}=d_{N-1}-N_{N-1}$, where $N_{j}=1+G_{cj}$ represents the state occupied by the particle plus $G_{cj}$, the number of states excluded uniquely by the *j*th particle [17]. Note that $G_{cj}$ accounts only for the states excluded by particle *j*, not by particles 1,…,j−1. The recursion can be interpreted as representing the most probable configurations at thermodynamic equilibrium.

In the thermodynamic limit j→N, N,G→∞ and N/G→n; thus, the exclusion $G_{c}$ depends only on *n*. Along with the recursion, we introduce a counting ansatz to evaluate $G_{cj}$, assuming that $G_{cj}=g_{c}\;d_{j}/G$, where $g_{c}$ is a system-dependent parameter called the exclusion correlation parameter, where $d_{j}/G$ represents the fraction of states still accessible to the *j*th particle, and $g_{c}\;d_{j}/G$ resembles a mean-field-like approximation over single-particle states. In principle, one could consider $g_{c}=g_{c}\left(n\right)$. The assumption that $g_{c}=\mathrm{constant}$ corresponds to a first-order approximation in ME statistics [17].

Thus, the recursion becomes:

(221) $$\begin{array}{cc} d_{1} & =G,\\ d_{2} & =d_{1}-\left[1+g_{c}\left(d_{1}/G\right)\right],\\ d_{3} & =d_{2}-\left[1+g_{c}\left(d_{2}/G\right)\right]\\ \; & =G{\left(1-g_{c}/G\right)}^{2}-\left(1-g_{c}/G\right)-1,\\ \ldots \\ d_{N} & =d_{N-1}-\left[1+g_{c}\left(d_{N-1}/G\right)\right],\\ d_{N} & =G{\left(1-g_{c}/G\right)}^{N-1}-\sum_{i=0}^{N-2}{\left(1-g_{c}/G\right)}^{i} \end{array}$$

In the limit $\tilde{d}\left(n\right)=\lim_{N,G\to \infty }d_{N}/G$ with $n=\lim_{N,G\to \infty }N/G$. Retaining the first term of the sum yields $\tilde{d}\left(n\right)=e^{-ng_{c}}-n$, which describes the fraction of states (relative to total *G*) accessible to a particle at occupation *n*.

More generally, $\tilde{d}\left(n\right)$ takes the form $\tilde{d}\left(n\right)=C_{1}e^{-ng_{c}}-C_{2}n$, where the constants $C_{1}$, $C_{2}$ are determined according to the boundary conditions: $\tilde{d}\left(0\right)=1$ and $\tilde{d}\left(n_{m}\right)=\tilde{d}\left(1/g\right)=d_{s}$ at saturation. This gives $C_{1}=1$ and $C_{2}=g\left(e^{-g_{c}/g}-{\tilde{d}}_{s}\right)$, with ${\tilde{d}}_{s}$ being the state density at maximum occupation $n_{m}=1/g$. Hence,

(222) $$\tilde{d}\left(n\right)=e^{-ng_{c}}-g\left(e^{-g_{c}/g}-{\tilde{d}}_{s}\right)n.$$

For systems where the entropy per state vanishes at saturation (i.e., $\tilde{S}\left(n_{m}\right)=0$), such as symmetric *k*-mers on a 1D lattice, one has ${\tilde{d}}_{s}=0$. However, in most structured particle systems on lattices, $\tilde{S}\left(n_{m}\right)>0$ and, therefore ${\tilde{d}}_{s}>0$.

For independent particles with uncorrelated states, $g_{c}=0$, ${\tilde{d}}_{s}=0$, and Equation (222) reduces to $\tilde{d}\left(n\right)=1-gn$, corresponding to Haldane–Wu fractional statistics for constant state exclusion *g* per particle. While Haldane’s generalization of the Pauli principle was originally introduced for quantum particles with 0≤g≤1 [14], structured classical particles with excluded-volume interactions can behave as super-fermions with g>1.

To better illustrate how the ME framework generalizes known statistics, from Equations (215) and (217), the ME distribution function n(μ) can be written as

(223) $$n\left(\mu \right)=\frac{e^{-g_{c}n}}{w\left(\xi \right)+g\left(e^{-g_{c}/g}-{\tilde{d}}_{s}\right)},$$

where $\xi =e^{\beta (\epsilon_{0}-\mu )}$ and $w\left(\xi \right)=\tilde{d}\left(n\right)/n$ satisfies

(224) $$\xi ={\left[w\left(\xi \right)+1\right]}^{{\tilde{d}}^{\prime }}{\left[w\left(\xi \right)\right]}^{-{\tilde{d}}^{\prime }}.$$

In the limiting cases: for independent states ($g_{c}=0$, ${\tilde{d}}_{s}=0$) and g=1, ${\tilde{d}}^{\prime }\left(n\right)=-1$, such that ξ=w(ξ) and Equation (223) becomes the Fermi–Dirac distribution. For g=0, ${\tilde{d}}^{\prime }\left(n\right)=0$, ξ=w(ξ)+1, recovering the Bose–Einstein statistics. For constant exclusion 0<g<1, with ${\tilde{d}}^{\prime }\left(n\right)=-g$, Equation (224) reduces to Wu’s equation $\xi ={\left[w\left(\xi \right)+1\right]}^{1-g}{\left[w\left(\xi \right)\right]}^{g}$, and Equation (223) yields the Haldane–Wu fractional *g*-statistics [14,15].

In general, for particles with spatially correlated states (as studied here), one finds g>1, $g_{c}\geq 0$, and ${\tilde{d}}_{s}\geq 0$, and Equation (224) must be solved for *n* at a given chemical potential. In this work, we alternatively compute μ as a function of *n* directly using Equation (214).

### 5.3. Density of States Parameters

In this section, we examine the statistical and physical interpretations of the parameters involved in the density of states function $\tilde{d}\left(n\right)$, and discuss how these parameters are practically determined through the relationship between lattice topology and particle structure (e.g., size and shape) via the thermodynamic limits of the exclusion spectrum.

In Section 5.2, the constant ${\tilde{d}}_{s}$ was introduced such that the density of states satisfies the boundary condition $\lim_{n\to n_{m}}\tilde{d}\left(n\right)={\tilde{d}}_{s}$. Physically, ${\tilde{d}}_{s}$ represents the ratio between the number of states available to a particle at saturation and the number of states available to an isolated particle. Generally, for a phase of structured particles on a lattice at saturation, a finite number of configurations per particle is expected, such that ${\tilde{d}}_{s}\geq 0$. The saturation entropy per state ${\tilde{S}}_{s}=\lim_{n\to n_{m}}\tilde{S}\left(n\right)$ is related to ${\tilde{d}}_{s}$ via Equations (213) and (222), yielding

(225) $$\frac{{\tilde{S}}_{s}}{k_{B}}=\left({\tilde{d}}_{s}+\frac{1}{g}\right)\ln\left({\tilde{d}}_{s}+\frac{1}{g}\right)-{\tilde{d}}_{s}\ln{\tilde{d}}_{s}-\frac{1}{g}\ln\frac{1}{g}.$$

The parameter ${\tilde{d}}_{s}$ (or, alternatively, the saturation entropy ${\tilde{S}}_{s}$) is the only free parameter of the ME statistics needed to describe a wide class of complex lattice gases. However, in the analysis of the *k*-mers problem on the square lattice (developed in subsequent sections), ${\tilde{d}}_{s}$ is not treated as a free parameter. Instead, it is fixed for each value of *k* using Equation (225), in order to match the Monte Carlo values of ${\tilde{S}}_{s}$ reported in [116,117].

As an example, for *k*-mers on a square lattice, assuming that the entropy vanishes at saturation (${\tilde{S}}_{s}=0$) implies ${\tilde{d}}_{s}=0$. With this minimal approximation, the ME formalism predicts an isotropic–nematic (I-N) transition at intermediate coverage for k≥6. However, both the I-N and a high-density nematic–isotropic (N-I) transition arise only for k≥7; even for small positive values of ${\tilde{d}}_{s}$. This behavior is discussed further in the following section.

Next, we determine the exclusion correlation parameter $g_{c}$ from the lattice and particle characteristics. For a given particle–lattice system, the total number of distinguishable single-particle states *G* and the number of particles at saturation $N_{m}$ (i.e., the maximum number of particles that fit without overlap) are first computed. Then, $g=G/N_{m}$ gives the number of states excluded per particle at full coverage. For instance, for rod-like *k*-mers on a 1D lattice with *M* sites, G=M, $N_{m}=M/k$, and g=k. On a 2D square lattice with $M=L^{2}$ sites, G=2M (two orientations per site) and $N_{m}=M/k$, leading to g=2k.

As each particle occupies one state, the occupation number n=N/G (i.e., fraction of occupied states) is related to the lattice coverage θ=kN/M=2kN/G=gn.

The exclusion correlation parameter $g_{c}$ can be determined from the configurational boundary condition at infinite dilution. In this limit, G(n→0) represents the number of states excluded by an isolated particle, denoted by $f_{0}$. From the definitions of $P_{o}\left(n\right)$, E(n), e(n), and $\tilde{d}\left(n\right)$ [Equations (217)–(222)], we find that $\lim_{n\to 0}G\left(n\right)=\lim_{n\to 0}E\left(n\right)/$$n=\lim_{n\to 0}e\left(n\right)=f_{0}$, which gives

(226) $$\lim_{n\to 0}G\left(n\right)=2g\left(e^{-g_{c}/g}-{\tilde{d}}_{s}\right)+2g_{c}-1=f_{0}.$$

This fundamental ME equation relates model parameters to the number of states excluded by an isolated particle. As $f_{0}$ is known for a given species on a lattice, Equation (226) can be solved to determine $g_{c}$. The solution has an analytical form:

(227) $$g_{c}=\frac{1}{2}\left[1+f_{0}+2g\left(W\left(z\right)-{\tilde{d}}_{s}\right)\right],$$

where W(z) is the principal branch of the Lambert function, with $z=-\exp\left(-\frac{1}{2g}-\frac{f_{0}}{2g}-{\tilde{d}}_{s}\right)$. Note that W(z) satisfies $z=W\left(z\right)e^{W(z)}$, with W(−1/e)=−1, W(0)=0, and W(z)→∞ as z→∞. The function was introduced by Lambert in 1758 [120], and is present in various problems in modern physics [121,122,123]. Details of the Lambert function are given in the following section.

Moreover, $\lim_{n\to n_{m}}G\left(n\right)=g+O\left({\tilde{d}}_{s}\right)$, such that the limits of G(n) at n→0 and $n\to n_{m}$ provide a full characterization of state exclusion.

In 1D systems of ideal *k*-mers, we have g=k, $f_{0}=2k-1=2g-1$, and ${\tilde{d}}_{s}=0$, which yield $g_{c}=0$ for any *k*, reproducing the exact results of Ref. [20].

In contrast, for straight rigid *k*-mers on square lattices with G=2M and $N_{m}=M/k$, it follows that $n_{m}=1/\left(2k\right)=1/g$, so g=2k. The number of excluded states is $f_{0}=k^{2}+2k-1=g^{2}/4+g-1$ for k≥2, while g=1 and $f_{0}=1$ for monomers (k=1). Here, $k^{2}$ accounts for exclusion across the particle, and 2k−1 for exclusion along it. The solution for $g_{c}$ becomes:

(228) $$g_{c}=g\;W\left(z\right)+\frac{g^{2}}{8}+\frac{g}{2}+g\;{\tilde{d}}_{s},$$

with $z=-\exp\left(-\frac{g}{8}-\frac{1}{2}-{\tilde{d}}_{s}\right)$.

Statistically, $g_{c}$ originates from the ME state-counting ansatz in Section 5.2. The exponential decay term $e^{-g_{c}n}$ in $\tilde{d}\left(n\right)$ becomes $e^{-g_{c}\theta /g}$ in terms of the lattice coverage θ=gn. The ratio $g/g_{c}$ thus defines the typical coverage at which the ME term in the density of states decays. For instance, in the *k*-mer model discussed in the following, the isotropic–nematic transition occurs around the point where $\tilde{d}\left(n\right)∼e^{-1.5}$, indicating that most of the single-particle states are excluded, as well as most of the isotropic-phase configurations. Hence, the ratio $g_{c}/g$ has a clear statistical and physical meaning.

### 5.4. Lambert Function

The Lambert function W(z), introduced in 1758 [120], is defined by the equation $z=W\left(z\right)e^{W(z)}$, with notable values being W(−1/e)=−1, W(0)=0, and W(z)→∞ as z→∞.

The solution of Equation (226), $2g\left(e^{-g_{c}/g}-{\tilde{d}}_{s}\right)+2g_{c}-1=f_{0}$, yields:

(229) $$g_{c}=\frac{1}{2}\left[1+f_{0}+2g\left(W_{+}\left(z\right)-{\tilde{d}}_{s}\right)\right],$$

with $z=-\exp\left(-\frac{1}{2g}-\frac{f_{0}}{2g}-{\tilde{d}}_{s}\right)$, which is valid for $0<{\tilde{d}}_{s}<1$, $f_{0}\geq 1$, and $1\leq g\leq \frac{1+f_{0}}{2(1-{\tilde{d}}_{s})}$. The function $W_{+}\left(z\right)$ denotes the principal (positive) branch. In particular, $g_{c}=0$ for $g=\frac{1+f_{0}}{2(1-{\tilde{d}}_{s})}$. For *k*-mers in 1D, g=k, $f_{0}=2k-1=2g-1$, which results in $g_{c}=0$.

For equations of the form $e^{ax}=bx+c$, with b,c≠0, a substitution $t=-ax-\frac{ac}{b}$ transforms it into $te^{t}=z=-\frac{a}{b}e^{-ac/b}$, with solution t=W(z), yielding $x=-\frac{t}{a}-\frac{c}{b}$. Specifically, for *k*-mers, setting a=−1/g, b=−1/g, and $c=\frac{f_{0}+1}{2g}+{\tilde{d}}_{s}=\frac{g}{8}+\frac{1}{2}+{\tilde{d}}_{s}$, we obtain:

(230) $$g_{c}=g\;W\left(-e^{-(g/8+1/2+{\tilde{d}}_{s})}\right)+\frac{g^{2}}{8}+\frac{g}{2}+g\;{\tilde{d}}_{s},$$

which is valid for g≥4, where W(z) is the principal branch.

### 5.5. Entropy of k-mers on Square Lattices: Orientational Phase Transitions

This section explores the phase transitions exhibited by rigid, linear *k*-mers adsorbed onto a square lattice. The earliest comprehensive investigation of this system was conducted in Ref. [124], where MC simulations provided compelling evidence for the emergence of nematic order at intermediate coverage when k≥7, beyond a threshold density $\theta_{1c}$. Additionally, through high-density series expansions, Ghosh and Dhar [124] proposed the existence of a second transition—that is, from a nematic to a disordered phase—at a critical coverage $\theta_{2c}$ that scales as $\theta_{2c}\propto 1-k^{-2}$ for large *k*.

Following this foundational work, multiple studies have analyzed the phase behaviors of long, rigid rods on two-dimensional lattices with discrete orientational degrees of freedom [68,125,126,127,128,129,130,131,132,133]. These investigations have confirmed that, for lengths below a certain threshold $k_{\min}$, no phase transition takes place. However, when $k\geq k_{\min}$, the system undergoes two distinct phase transitions as the density increases: a disordered isotropic phase at low coverage, a nematic phase at intermediate coverage characterized by orientational alignment of *k*-mers, and a second disordered phase at high coverage where alignment is lost. The threshold length $k_{\min}$ is geometry-dependent, with $k_{\min}=7$ on square [124,125] and triangular [126] lattices, and $k_{\min}=11$ on honeycomb lattices [127]. The first transition, from isotropic to nematic order, is continuous and occurs at $\theta =\theta_{1c}$. On square lattices, this transition falls into the universality class of the two-dimensional Ising model [125] whereas, for triangular and honeycomb geometries, it belongs to the three-state Potts model class [125,127]. In all cases, the critical density $\theta_{1c}$ scales approximately as $\theta_{1c}\left(k\right)\propto k^{-1}$ [126]. The existence of this isotropic–nematic (I-N) transition has also been established rigorously [128].

The second transition—from nematic to a high-density disordered phase—is less well understood. Earlier work [129] has indicated a continuous character for k≥7, whereas more recent MC studies [133] have reported evidence of a first-order transition, including phase coexistence for k=9.

This section focuses specifically on the square lattice case. For k=7, the isotropic–nematic transition occurs around θ≈0.745, corresponding to n≈θ/(2k)=0.0532, while the nematic–disordered transition takes place near θ≈0.917, or n≈0.0655 [129,131].

To characterize these transitions, we examine the entropy per site as a function of the particle density *n*, as derived from ME statistics for both isotropic and nematic phases. Two approximation schemes are considered: (i) a first-order model in which the combinatorial factor $g_{c}$ is assumed constant, and the entropy at saturation for the isotropic phase is fitted to MC data such that $\tilde{S}s=\tilde{S}MC$, while ${\tilde{d}}_{s}>0$ is determined using Equation (225). We also examine the limit ${\tilde{S}}_{s}\to 0$, ${\tilde{d}}_{s}\to 0$, where entropy vanishes at saturation, and its impact on the phase behavior; and (ii) a second-order model, where $g_{c}\equiv g_{c}\left(n\right)$ varies linearly and decays slowly with density.

**Figure 6:**
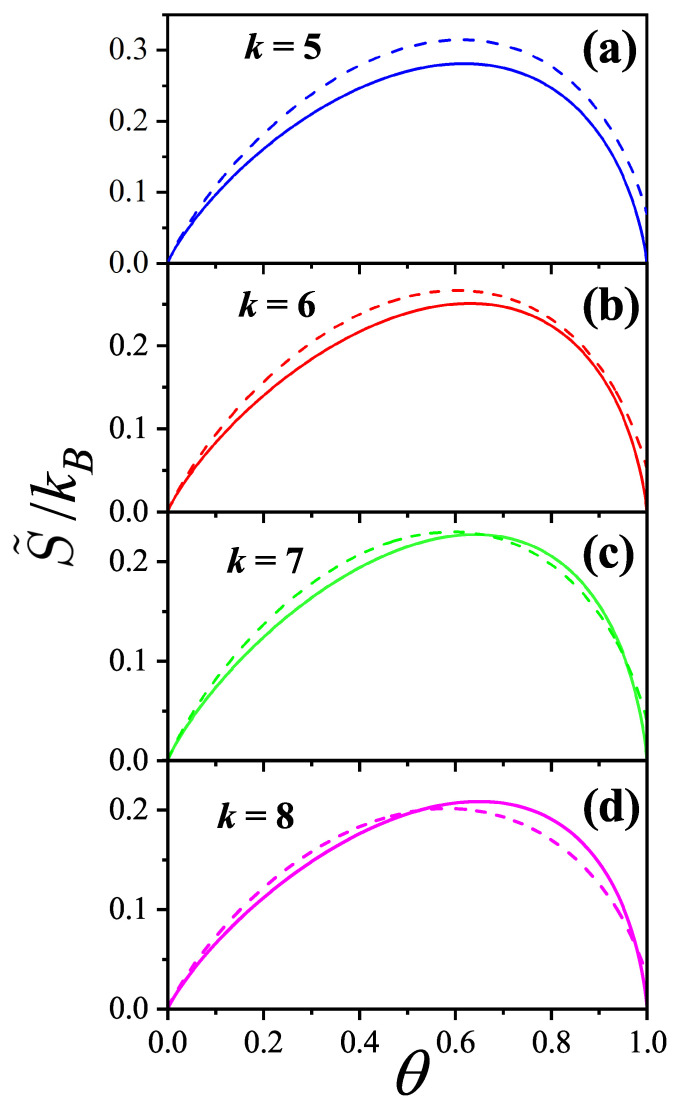
(**a**) Entropy per site vs. lattice coverage θ for k=5. Dashed lines: isotropic phase (I). Solid lines: fully aligned nematic phase (N). First-order ME approximation through Equations (213), (222) and (228). (**b**) Same as part (**a**) but for k=6. (**c**) Same as part (**a**) but for k=7. (**d**) Same as part (**a**) but for k=8.

In both approaches, critical points are identified by comparing the entropy per site for the isotropic and nematic states at equal occupation *n*—${\tilde{S}}_{N}\left(n\right)=\tilde{S}\left(n\right)$ for the nematic phase and ${\tilde{S}}_{I}\left(n\right)=2\tilde{S}\left(n\right)$ for the isotropic phase—thus accounting for the two orientational degrees of freedom per site in the isotropic case versus one in the aligned state. Figure 6 presents the entropy per site as a function of surface coverage θ for k=5,6,7, and 8, as calculated via Equations (213), (222), (225), and (226).

For *k*-mers in a fully aligned nematic phase, the parameters are $g_{N}=k$, $f_{0,N}=2k-1$, ${\tilde{S}}_{s,N}=0$, ${\tilde{d}}_{s,N}=0$, and $g_{c,N}=0$, according to Equation (226). For the isotropic phase, we have $g_{I}=2k$, $f_{0,I}=k^{2}+2k-1$, and ${\tilde{S}}_{s,I}={\tilde{S}}_{MC}$; ${\tilde{d}}_{s,I}$ and $g_{c,I}$ are computed using Equations (225) and (226). The entropy values ${\tilde{S}}_{s,I}={\tilde{S}}_{MC}$ are taken from [116,117,134] for k=2 to 10 and, for k>10, ${\tilde{S}}_{s,I}=k^{-2}\ln k$ and the corresponding values of ${\tilde{d}}_{s,I}$ have been reported in [19]. The critical coverages $\theta_{c}$ are determined by solving the equation ${\tilde{S}}_{I}\left(\theta_{c}/g_{I}\right)={\tilde{S}}_{N}\left(\theta_{c}/g_{N}\right)$.

This equation yields two solutions, $\theta_{c,I-N}$ and $\theta_{c,N-I}$, only for k≥7; for k<7, there is no solution other than the trivial equality at zero coverage. This behavior is shown in Figure 6.

In particular, for k=6, Figure 6b shows that ${\tilde{S}}_{I}>{\tilde{S}}_{N}$ for 0<θ≤1, indicating that the isotropic phase is the only stable phase. The same holds for k<6. Notably, for k=6, the smallest entropy difference occurs near θ≈0.9.

For k=7, there are two critical coverages: $\theta_{c,I-N}=0.658$ and $\theta_{c,N-I}=0.954$ in the first-order approximation. This dual-transition pattern is found for all k≥7, consistent with the results discussed, where a mixture of cross-exclusion of differently oriented species is considered. Although the nature of the transitions is not addressed here, it has been shown that the I→N transition is continuous, indicating that this formalism does not matches a typical mean-field approach. For the sake of reference, in Bethe lattices with coordination *q*, a first-order transition occurs for k≥4 depending on *q* [67] for this nematic transition.

In the second-order approximation, $g_{c}\left(n\right)$ is assumed to decay slowly with density as $g_{c}\left(n\right)=g_{c}-bg_{c}\theta$, with $g_{c}\left(0\right)=g_{c}$ and $g_{c}\left(1\right)=g_{c}\left(1-b\right)$. For b=0.22, the critical coverages for k=7 are $\theta_{c,I-N}=0.746$ and $\theta_{c,N-I}=0.920$, which agree remarkably well with MC values ($\theta_{MC,I-N}=0.745$ and $\theta_{MC,N-I}=0.917$) [129].

**Figure 7:**
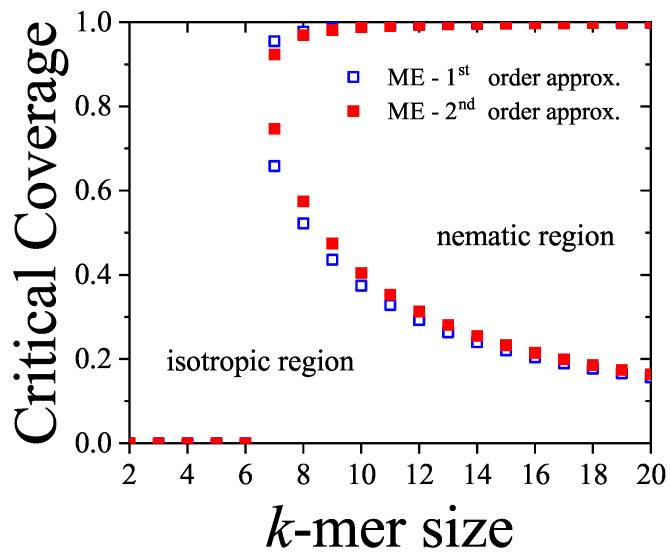
Critical coverages $\theta_{c,I-N}$ (lower branch) and $\theta_{c,N-I}$ (upper branch) as functions of *k*. Open squares: first-order ME approximation. Solid squares: second-order ME approximation.

Figure 7 shows the variation of $\theta_{c}$ with *k*. As k→∞, $\theta_{c,I-N}\to 0$ and $\theta_{c,N-I}\to 1$.

This model predicts a sequence of phases: isotropic at low/intermediate density, nematic at higher density, and isotropic again at high coverage, in agreement with the MC results for k≥7. Even assuming vanishing saturation entropy, the ME formalism still predicts an I-N transition for k≥6, although no N-I transition occurs.

**Figure 8:**
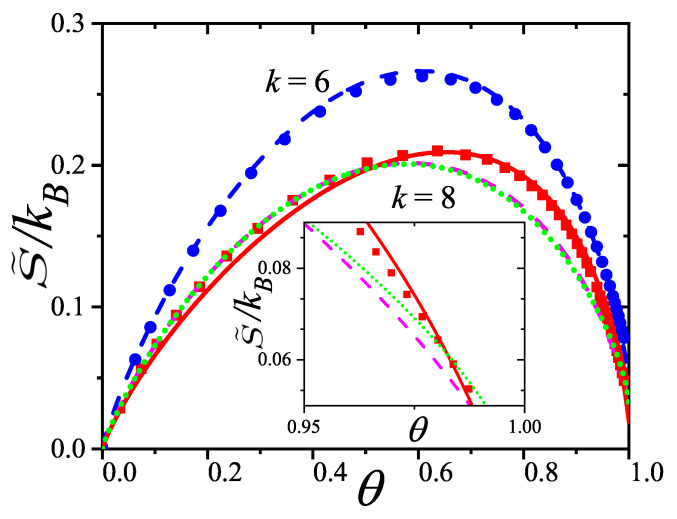
Entropy per site vs. lattice coverage θ for k=6 and 8. Symbols: MC data [135]. Dashed: ME model (isotropic phase) with ${\tilde{d}}_{s}=0.007545$ (k=6), ${\tilde{d}}_{s}=0.004316$ (k=8). Solid: ME result for nematic phase (${\tilde{d}}_{s}=0$). Dotted: empirical correction ${\tilde{S}}_{E}$. Inset: high coverage behavior for k=8.

Figure 8 compares the ME results with MC data for k=6 and 8, showing good agreement in both phases, especially at intermediate coverage. Discrepancies at high density explain differences in βμ, as it is equal to the derivative of entropy with respect to θ (in $k_{B}$ units).

For ${\tilde{S}}_{s}=0$, Equation (222) becomes exact only in 1D. In 2D systems, $\tilde{S}\left(n\to n_{m}\right)>0$ due to allowed local configurations at high coverage [116,117]. Thus, ${\tilde{d}}_{s}=0$ underestimates entropy at high density. This distinction is critical for understanding the N-I transition for k≥7, as seen in the inset of Figure 8.

While ME already captures high coverage entropy via ${\tilde{d}}_{s}$, an empirical correction improves quantitative agreement. We define ${\tilde{S}}_{E}\left(\theta \right)=\tilde{S}\left(\theta ;{\tilde{d}}_{s}=0\right)+\Delta \tilde{S}\left(\theta \right)$, where $\Delta \tilde{S}\left(\theta \right)={\tilde{S}}_{s}\;\theta^{\alpha }\exp\left[\left(\theta -1\right)/\gamma \right]$. The term ${\tilde{S}}_{s}$ matches the MC saturation entropy, $\theta^{\alpha }$ ensures $\tilde{S}\left(0\right)=0$, and the exponential captures high coverage behavior.

Appropriate values of α≪1 and γ∼0.05--0.06 reproduce the MC results in both isotropic and nematic regimes. As γ≪1, the correction is significant only when very close to saturation. Saturation entropy values are taken as ${\tilde{S}}_{s}={\tilde{S}}_{MC}/2=0.1465,0.0795,0.0505,\ldots$ for k=2,…,7, and ${\tilde{S}}_{s}=\left(\ln k/k^{2}\right)/2$ for k>7 [116,117,134].

This empirical correction also accurately reproduces the entropy of more complex particles, such as trimers (straight or bent) and triangles on triangular lattices [136].

**Figure 9:**
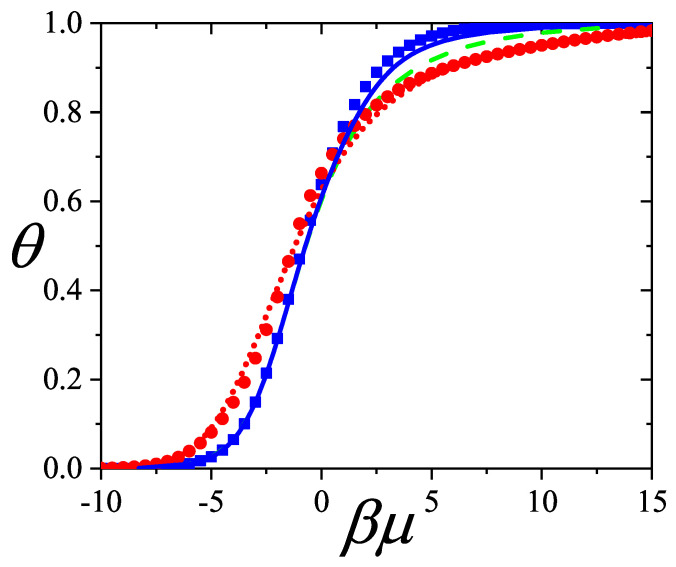
Lattice coverage θ versus βμ for *k*-mers on a square lattice. k=2: g=4, $f_{0}=7$, $g_{c}=1.42$, ${\tilde{d}}_{s}=0$ (dashed line), ${\tilde{d}}_{s}=0.0560$ (solid line); k=10: g=20, $f_{0}=81$, ${\tilde{d}}_{s}=0.00264$, $g_{c}=39$ (dotted line). Symbols denote MC simulation results: squares for k=2, circles for k=10.

Concerning the coverage dependence of the chemical potential from ME statistics in the following, we reproduce the results of μ(n) for two very different *k*-mer sizes, k=2 and k=10, as illustrative examples in Figure 9 [18].

The variables are expressed, in terms of coverage θ, via the substitution n→θ/g. For further details on the simulations, see Refs. [17,63,129,137].

In Figure 9, MC data for *k*-mers are compared with two analytical predictions. The dashed line corresponds to the limiting case where entropy vanishes at saturation; i.e., ${\tilde{S}}_{s}=0$, ${\tilde{d}}_{s}=0$. When the correct value ${\tilde{d}}_{s}>0$ is used, the ME approximation (through Equation (222)) provides better agreement with the MC data at high coverage, as seen in the solid line.

The discrepancy at high coverage for k=2 between theory and MC is attributable to the behavior of the entropy $\tilde{S}\left(n\right)$ near $n\to n_{m}$ in Equation (213). A qualitatively similar result is obtained when using the empirical form ${\tilde{S}}_{E}$ introduced in the previous section.

For k≥7, MC simulations show that *k*-mers undergo a continuous I-N transition at intermediate θ [124], consistent with predictions from the ME model based on entropy comparison between nematic and isotropic phases. Despite this, Equation (214) still gives a fair approximation for μ(θ) if a value of $g_{c}$ smaller than that from Equation (228) is used.

This phenomenon can be understood as follows: nematic ordering forms compact bundles of neighboring *k*-mers. For a fixed particle number *N*, such alignment leads to more multiple excluded states and fewer total excluded states per particle, effectively reducing the parameter $g_{c}$. A simple illustration of this can be made by comparing the number of states excluded when two *k*-mers are perpendicular vs. parallel and aligned.

For instance, in Figure 9, the case k=10 with $g_{c}=39$ yields a good fit to the MC data. This value of $g_{c}$ is significantly smaller than the isotropic value $g_{c}=59$ obtained from Equation (228), reflecting bundle-like configurations of the lattice gas.

The MC simulation results presented in this section were obtained using the efficient algorithm introduced by Kundu et al. [63,129,137] and described in Section 8.5. Simulations were performed on L×L square lattices with periodic boundary conditions. The ratio L/k was set to 120—a value for which finite-size effects were found to be negligible. Equilibrium was typically reached after $10^{7}$ MCSs, and observables were computed by averaging over $10^{7}$ configurations.

Illustrative results for μ(n) from ME statistics for squares and rectangles on square lattices have been presented and compared with fast-relaxation Monte Carlo simulations in Ref. [18]. A comprehensive study of the various phases formed by rectangles on square lattices, as well as other hard-core lattice gases, can be found in Refs. [61,62,63,138].

### 5.6. Exclusion Spectrum Functions

A singular outcome of this formalism is the thermodynamic characterization of the configuration space or state exclusion spectrum through the exclusion per particle frequency function e(n) and the cumulative exclusion per particle function G(n), which we refer to collectively as exclusion spectrum functions.

From Equations (219) and (220), both G(n) and e(n) can be expressed in terms of the density dependence of the chemical potential, providing a thermodynamic description of equilibrium particle configurations.

**Figure 10:**
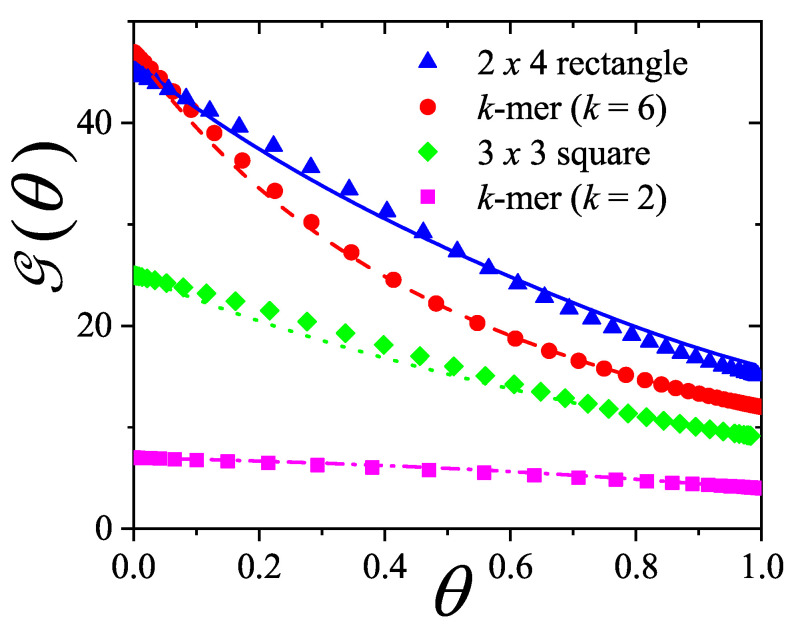
Exclusion spectrum function G(θ) vs. lattice coverage θ. Lines: ME theory from Equation (219); dot-dashed (k=2), dashed (k=6), solid (2×4 rectangles), dotted (3×3 squares). Symbols: MC data as indicated.

**Figure 11:**
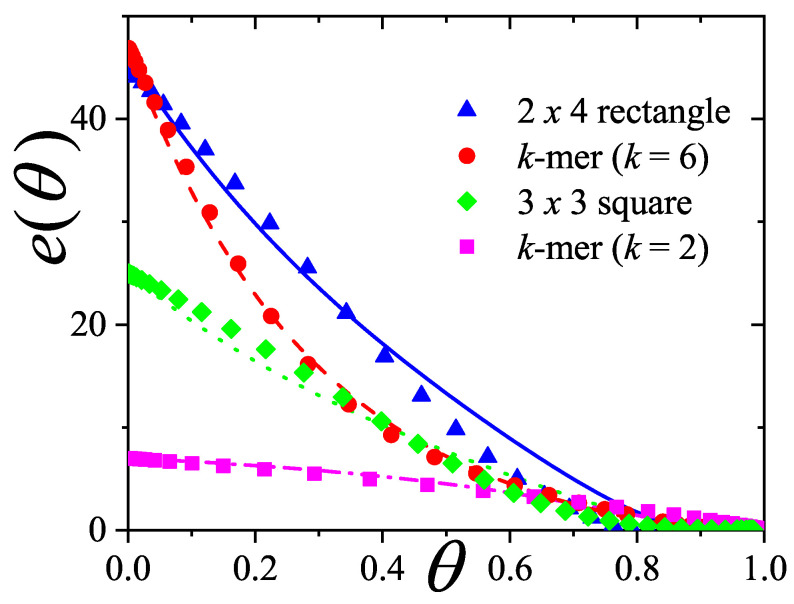
Same as Figure 10, but for the exclusion per particle frequency function e(θ).

Illustrative results for G(θ) and e(θ) for *k*-mers, squares, and rectangles in the isotropic phase are shown in Figure 10 and Figure 11. Analytical results are shown as lines, and MC simulation data are indicated with symbols: 2×4 rectangles (triangles), k=6 *k*-mers (circles), 3×3 squares (diamonds), and k=2 *k*-mers (squares). The simulations were performed following the same scheme and using the same parameters as in Section 5.5.

For k=6 with $g_{c}=29.83$, both G(θ) and e(θ) decrease rapidly with increasing coverage: G(0)=47→G(1)=12 and e(0)=47→e(1)=0. In contrast, in a 1D lattice with $g_{c}=0$, the decay is slower: G(0)=11→G(1)=6 and e(0)=11→e(1)=0 (not shown in Figure 10 and Figure 11 for clarity).

In the other cases shown: for 2×4 rectangles, G(0)=46, G(1)=16, e(0)=46, e(1)=0; for 3×3 squares, G(0)=25, G(1)=9, e(0)=25, e(1)=0; for k=2 *k*-mers, G(0)=7, G(1)=4, e(0)=7, e(1)=0. A good agreement can be observed between analytical predictions and MC data for both G(θ) and e(θ), particularly for $e\left(0\right)=G\left(0\right)=f_{0}$. At saturation, G(1)=g as each particle excludes on average *g* states, and e(1)=0 as all single-particle states are either occupied or excluded.

As shown in Figure 10 and Figure 11, ME statistics provide an accurate description of the exclusion spectrum functions across the full range of θ. In particular, the results for *k*-mers show excellent agreement for both small and large *k*. Moreover, particles with higher *g* tend to exhibit larger values of G(θ), regardless of shape. In contrast, e(θ) captures more detailed configuration-specific features, as evidenced in Figure 11. For instance, while isolated k=6*k*-mers exclude more states than 3×3 squares, there exists an intermediate coverage range 0.54<θ<0.85 where they exclude fewer states per particle. This indicates local alignment among *k*-mers at high θ, reducing exclusion relative to a disordered configuration.

These results confirm that ME statistics capture the thermodynamic signature of configurational exclusion with remarkable accuracy across all densities. A more in-depth analysis of the exclusion spectrum and its behavior near phase transitions is provided and discussed in the following sections.

### 5.7. Adsorption of Polyatomics: Relationships Between Exclusion Functions, Thermodynamic Observables, and Adsorption Field Topology

In this section, we explore potential applications of the exclusion spectrum functions defined in Section 5.1 in connection with experimental thermodynamic measurements. This relation provides insights into how adsorbed particles occupy and exclude states based on the spatial distribution of local minima in the adsorption field; generically referred to here as the adsorption field topology.

The average exclusion spectrum function G(n) connects a configurational property, related to the spatial correlation of states and influenced by particle geometry, to the density dependence of a thermodynamic observable such as the chemical potential. From the relation

(231) $$G\left(n\right)=E\left(n\right)/n=\frac{1}{n}-\frac{1}{e^{\beta (\mu -\epsilon_{0})}}=\frac{1}{n}-\frac{1}{K\left(T\right)e^{\beta \mu }}$$

or, equivalently, $E\left(n\right)=1-\left[ne^{-\beta \mu }/K\left(T\right)\right]$, it becomes apparent that these exclusion functions can—in principle—be inferred from experiments via the dependence of μ on *n*.

A more refined exclusion description is provided by the frequency function e(n)=dE(n)/dn. Introducing $\zeta \left(n\right)=ne^{-\beta \mu }$, we have $G\left(n\right)=\left[1-\zeta (n)/K(T)\right]/n$ and $e\left(n\right)=-\frac{1}{K(T)}\;d\zeta \left(n\right)/dn$. Hence, the analytical or experimental form of ζ(n) encapsulates the configurational exclusion information. Thus, for an adsorbate molecule of given shape and size, the number of states at very low coverage could be determined and the spatial arrangement of the adsorption potential minima could be inferred; which we refer to here as the adsorption potential topology [18]. Additionally, the complete configuration changes on density are embodied in G(n) and e(n) through ζ(n). A more detailed experimental analysis of adsorption isotherms on well-defined particle–substrate systems is needed to assess the feasibility and value of this configurational framework, which lies beyond the scope of the present review.

## 6. Latest Developments, Part II: Multiple Exclusion Statistics Formulation for Mixtures

In this section, the ME statistical thermodynamics framework is extended to describe mixtures of particles with arbitrary size and shape, each having a spectrum of topologically correlated states and subject to statistical exclusion. A generalized distribution is obtained from a configuration space ansatz recently proposed for single species, accounting for the multiple exclusion phenomenon, where correlated states can be simultaneously excluded by more than one particle. Statistical exclusion on correlated state spectra is characterized by the parameters $\beta_{cij}$, which are self-consistently determined. Self- and cross-exclusion spectral functions $e_{ij}\left(n\right)$ and $G_{ij}\left(n\right)$ are introduced to describe density-dependent exclusion behaviors. In the limit of uncorrelated states, the formalism recovers Haldane’s statistics and Wu’s distribution for single species and for mixtures of mutually excluding species with constant exclusion.

The formalism is applied to *k*-mers on the square lattice, modeled as a mixture of two orthogonally oriented self- and cross-excluding pseudo-species. This approach offers a general and consistent framework for entropy-complex lattice gases. It reproduces *k*-mer phase transitions and provides access to configurational information through the exclusion spectrum functions. Here, we summarize the basis of this approach for mixtures and the relevant analytical predictions for rigid *k*-mers on square lattices (as discussed in the section devoted to applications).

### 6.1. State-Counting Approximation and Density of States for Mixtures with Multiple State Exclusion

The general self-consistent formulation for the thermodynamics of mixtures consisting of an arbitrary number of species in a volume *V* is summarized in the following subsections. Each of the species is assumed to exclude accessible states to itself and to the others, a phenomenon intensified by spatial correlations among the states; this is referred to as Multiple Exclusion Statistics (ME) [17]. This leads to a particularly challenging statistical problem, especially in lattice models involving linear or arbitrarily shaped particles.

**Figure 12:**
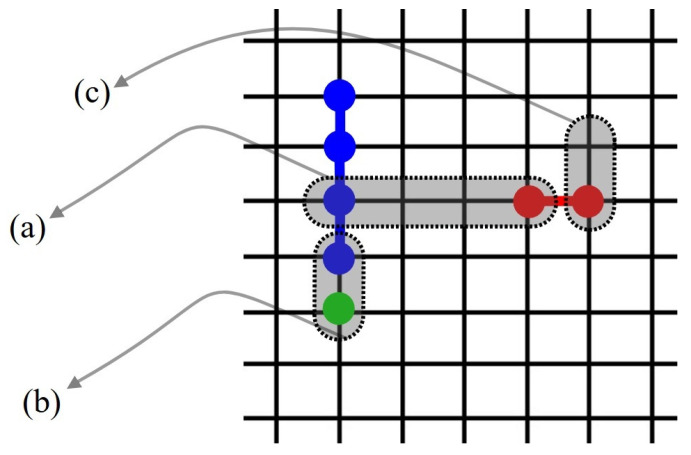
A schematic representation of a ternary mixture: a tetramer (blue), a dimer (red), and a monomer (green). The dotted lines indicate examples of multiply excluded states within the spectrum of (**a**) tetramers, (**b**) dimers due to tetramers and monomers, and (**c**) dimers via self-exclusion. This emphasizes the multiplicity of state exclusions even at finite densities.

Figure 12 illustrates ME in a ternary mixture of monomers, dimers, and tetramers. The single-species theory of Ref. [18] was extended to mixtures, and state density and exclusion distribution functions were derived [19] in order to formulate an analytical thermodynamic model which is applicable to *k*-mers on lattices with correlated spectra, as well to other particle shapes.

We define the self- and cross-exclusion parameters $g_{ii}$ and $g_{ij}$ based on the number of states excluded per particle at saturation. Let $G_{i}$ be the total states available to an isolated particle of species *i* and $N_{i}$ its number in *V*. The occupation number is $n_{i}=\lim_{N_{i},G_{i}\to \infty }N_{i}/G_{i}$, with $n_{i,m}=N_{i,m}/G_{i}$ its maximum, and $g_{ii}=1/n_{i,m}$.

Cross-exclusion is rather more subtly quantified by $g_{ij}=\left(G_{i}-G_{ij,m}\right)/N_{j,m}$, where $G_{ij,m}$ are the non-excluded states of *i* when *j* saturates the system. Expressed in terms of fractions, we have $g_{ij}=\Delta_{ij}\left(1-{\tilde{G}}_{ij,m}\right)/n_{j,m}$, with ${\tilde{G}}_{ij,m}=G_{ij,m}/G_{i}$ and $\Delta_{ij}=G_{i}/G_{j}$.

The canonical partition function is $Z\left(N,T,V\right)=W_{1,\ldots ,s}\left(N\right)\prod_{i=1}^{s}q_{i}^{N_{i}}e^{-\beta \sum N_{i}\epsilon_{i}}$, where $\epsilon_{i}$ denotes the energy per particle (eventually, due to an external field such as the interaction with the lattice) and $q_{i}=1$ henceforth. The configurational term $W_{1,\ldots ,s}\left(N\right)$ captures how particles distribute over their respective sets of accessible states.

We define $d_{i}\left(N\right)$ as the number of states available to a particle of species *i* given an occupation vector $N\equiv (N_{1},\ldots ,N_{s})$ (and analogously for n). Generalizing the form introduced in [18] for single species, the configuration count is:

(232) $$W_{1,\ldots ,s}\left(N\right)=\prod_{i=1}^{s}\frac{[d_{i}\left(N\right)+N_{i}-1]!}{N_{i}!\left[d_{i}\left(N\right)-1\right]!}.$$

Using Stirling’s approximation and defining βF=−lnZ, the Helmholtz free energy becomes:

(233) $$\begin{array}{cc} \beta F(N) & =\beta \sum_{i}N_{i}\epsilon_{i}+\sum_{i}[d_{i}\left(N\right)\ln d_{i}\left(N\right)+N_{i}\ln N_{i}\\ \; & \;-\left(d_{i}\left(N\right)+N_{i}\right)\ln\left(d_{i}\left(N\right)+N_{i}\right)]. \end{array}$$

In the thermodynamic limit, we define the free energy per state of species *i* as $\beta f_{i}\left(n\right)=\beta \lim_{G_{i}\to \infty }F/G_{i}$. This leads to

(234) $$\begin{array}{c} \beta f_{i}\left(n\right)=\beta \epsilon_{i}n_{i}+{\tilde{d}}_{i}\ln{\tilde{d}}_{i}+n_{i}\ln n_{i}-\left({\tilde{d}}_{i}+n_{i}\right)\ln\left({\tilde{d}}_{i}+n_{i}\right)\\ +\sum_{j\neq i}\Delta_{ji}\left[\beta \epsilon_{j}n_{j}+{\tilde{d}}_{j}\ln{\tilde{d}}_{j}+n_{j}\ln n_{j}-\left({\tilde{d}}_{j}+n_{j}\right)\ln\left({\tilde{d}}_{j}+n_{j}\right)\right], \end{array}$$

where ${\tilde{d}}_{i}\left(n\right)=\lim_{G_{i}\to \infty }d_{i}\left(N\right)/G_{i}$.

The entropy per state (in units of $k_{B}$) follows from Equation (234):

(235) $$\begin{array}{c} \frac{S_{i}\left(n\right)}{k_{B}}=\left({\tilde{d}}_{i}+n_{i}\right)\ln\left({\tilde{d}}_{i}+n_{i}\right)-{\tilde{d}}_{i}\ln{\tilde{d}}_{i}-n_{i}\ln n_{i}\\ +\sum_{j\neq i}\Delta_{ji}\left[\left({\tilde{d}}_{j}+n_{j}\right)\ln\left({\tilde{d}}_{j}+n_{j}\right)-{\tilde{d}}_{j}\ln{\tilde{d}}_{j}-n_{j}\ln n_{j}\right]. \end{array}$$

In general, $d_{i}\left(N\right)$ depends not only on the occupation vector but also on the microstates, which are inaccessible analytically. Thus, we postulate a functional form based on average occupations, which is sufficient for thermodynamic descriptions. The following section formalizes the derivation of ${\tilde{d}}_{i}\left(n\right)$ based on a counting ansatz and pairwise exclusion analysis.

It is worth noting that the expression for $W_{1,2,\ldots ,s}\left(N\right)$ is exact only if particles can occupy states that are completely independent of one another. This is the case in the Haldane–Wu *g*-statistics framework discussed in Ref. [18], where each particle excludes *g* states regardless of *N* or the specific configuration. For a single species, this leads to d(N)=G−gN with constant *g* while, for a mixture, the generalized form becomes $d_{i}\left(N\right)=G_{i}-\sum_{j=1}^{s}g_{ij}N_{j}$ with constants $g_{ij}$. In general, however, $d_{i}\left(N\right)$ in Equation (232) is only approximate, as the actual number of available states for species *i* should depend not only on N but also on the specific microscopic configuration of the ensemble; that is, it should be configuration-dependent.

Yet, as exact configuration-counting is intractable, we develop a general approximation for $d_{i}\left(N\right)$ based on a state-counting ansatz. In the thermodynamic limit, this approach yields the density of states ${\tilde{d}}_{i}\left(n\right)$ as a function only of the average occupation numbers at equilibrium. This can be interpreted as the effective density of states corresponding to typical equilibrium configurations which contribute most significantly to the system’s entropy.

We now provide the derivation of the functional form of ${\tilde{d}}_{i}\left(n\right)$. Let $G_{i}$ denote the set of available states for a single particle of species *i*, with cardinality $G_{i}=\left|G_{i}\right|$. We define the tuple $G=(G_{1},\ldots ,G_{s})$, whose components correspond to the total number of states for each species.

To quantify how the presence of other species modifies the state space of species *i*, we denote by $G_{i}^{*}\left(N\right)$ the number of available states for a particle of species *i* when the system contains N particles in total, satisfying $G_{i}^{*}\left(N\right)=G_{i}$ when $N_{j}=0$ for all *j*. We first isolate the effect of species *j* on species *i* by defining $G_{ij}\left(N_{j}\right)\equiv G_{i}^{*}\left(N\right)$ under the condition $N=(0,\ldots ,0,N_{j},0,\ldots ,0)$, with only species *j* present.

The recursion relation introduced in Section 5 for single species can be extended to such pair interactions. The function $G_{ij}\left(N_{j}\right)$ can be defined recursively as follows:

$$G_{ij}\left(0\right)=G_{i},\;G_{ij}\left(1\right)=G_{i}-N_{i,0},\;G_{ij}\left(2\right)=G_{ij}\left(1\right)-N_{i,1}\;\ldots$$

and, in general,

(236) $$G_{ij}\left(N_{j}\right)=G_{ij}\left(N_{j}-1\right)-N_{i,N_{j}-1},$$

where $N_{i,N_{j}}$ denotes the number of states of species *i* excluded by the $N_{j}$-th particle of species *j* added to the system.

Following the analogy with the single-species case, we posit that $N_{i,N_{j}}=1+G_{cij}$. Here, the term 1 accounts for the exclusion of at least one state, while $G_{cij}$ represents the additional number of excluded states due to spatial correlations between the state spectra of species *i* and *j*.

The correlation term $G_{cij}$ is defined by the state-counting ansatz [17] as $G_{cij}=g_{cij}G_{ij}\left(N_{j}\right)/G_{i}$. Substituting this into the recursive definition yields:

$$G_{ij}\left(1\right)=G_{i}-\left[1+g_{cij}\frac{G_{i}}{G_{i}}\right]=G_{i}\left(1-\frac{g_{cij}}{G_{i}}\right)-1,$$

$$G_{ij}\left(2\right)=G_{ij}\left(1\right)-\left[1+g_{cij}\frac{G_{ij}\left(1\right)}{G_{i}}\right],$$

and so forth. The general expression becomes:

(237) $$G_{ij}\left(N_{j}\right)=G_{i}{\left(1-\frac{g_{cij}}{G_{i}}\right)}^{N_{j}-1}-\sum_{l=0}^{N_{j}-2}{\left(1-\frac{g_{cij}}{G_{i}}\right)}^{l},$$

which is valid for $N_{j}\geq 2$.

For convenience, we introduce into Equation (237) the rescaled exclusion parameters $\beta_{cij}=\left(G_{j}/G_{i}\right)\;g_{cij}=\Delta_{ji}\;g_{cij}$ and, similarly $\beta_{ij}=\left(G_{j}/G_{i}\right)\;g_{ij}=\Delta_{ji}\;g_{ij}$, where $\Delta_{ji}=G_{j}/G_{i}$ and $\Delta_{ij}=1/\Delta_{ji}$.

Retaining only the leading term of the summation in Equation (238) and taking the thermodynamic limit ${\tilde{G}}_{ij}\left(n_{j}\right)=\lim G_{ij}\left(N_{j}\right)/G_{i}$, with $G_{i},G_{j}\to \infty$, $N_{j}\to \infty$, and $N_{j}/G_{j}\to n_{j}$, we obtain:

(238) $$\begin{array}{cc} {\tilde{G}}_{ij}\left(n_{j}\right) & =\lim_{L}\frac{G_{ij}\left(N_{j}\right)}{G_{i}}=\lim_{L}{\left[1-\frac{\beta_{cij}}{G_{j}}\right]}^{N_{j}-1}\\ \; & \;-\Delta_{ji}\sum_{l=0,\;N_{j}\geq 2}^{N_{j}-2}\frac{1}{G_{j}}{\left[1-\frac{\beta_{cij}}{G_{j}}\right]}^{l}, \end{array}$$

which yields ${\tilde{G}}_{ij}\left(n_{j}\right)=e^{-\beta_{cij}n_{j}}-\Delta_{ji}\;n_{j}$; in agreement with the approximation proposed in Ref. [17]. Here, ${\tilde{G}}_{ij}\left(n_{j}\right)$ represents the fraction of states of species *i* that remain available in the presence of a concentration $n_{j}$ of species *j*, under the condition that all other species are absent ($n_{k}=0$ for k≠j). Statistically, this function encodes the depletion of the state spectrum of species *i* due to the presence of species *j*, and can be interpreted as a pairwise statistical interaction function.

The function ${\tilde{G}}_{ij}\left(n_{j}\right)$ must satisfy the boundary conditions ${\tilde{G}}_{ij}\left(n_{j}\to 0\right)=1$ and ${\tilde{G}}_{ij}\left(n_{j}\to n_{j,m}\right)={\tilde{G}}_{ij,m}$. To ensure this, we define constants $C_{ij,1}$ and $C_{ij,2}$ such that ${\tilde{G}}_{ij}\left(n_{j}\right)=C_{ij,1}\;e^{-\beta_{cij}n_{j}}-C_{ij,2}\;\Delta_{ij}\;n_{j}$. Imposing the boundary conditions yields $C_{ij,1}=1$ and $C_{ij,2}=\left(e^{-\beta_{cij}n_{j,m}}-{\tilde{G}}_{ij,m}\right)/n_{j,m}$. Therefore, the final expression for the pair function becomes:

(239) $${\tilde{G}}_{ij}\left(n_{j}\right)=e^{-\beta_{cij}n_{j}}-\left[e^{-\beta_{cij}n_{j,m}}-{\tilde{G}}_{ij,m}\right]\frac{n_{j}}{n_{j,m}}.$$

**Figure 13:**
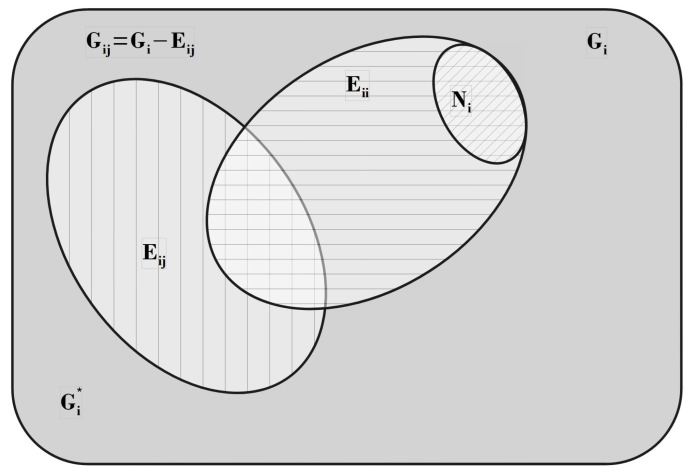
Symbolic representation of the state set of species *i* $G_{i}$ (whole framed area), whose elements are the states accessible to species *i* when $n_{j}=0$ for j=1,…,s, with $\left|G_{i}\right|=G_{i}$ denoting its cardinality. $E_{i1},E_{i2},\ldots ,E_{is}$ represent the sets of states of a particle of species *i* excluded by the particles of species 1,…,s, respectively (shown generically for $E_{ij}\;j\neq i$ and $E_{ii}$ by the areas filled by vertical and horizontal lines), with cardinalities $\left|E_{ij}\right|=E_{ij}$. The states occupied by species *i* are represented by the set $N_{i}$ (oblique lines area). $G_{ij}=G_{i}-E_{ij}$ represents the set of states for particles of species *i* not excluded by particles of species *j*. The intersection $G_{i}^{*}=\cap_{j=1}^{s}G_{ij}$ is the set of states for a particle of species *i* non-excluded by any of the species j=1,…,s (dark gray area), with cardinality $\left|G_{i}^{*}\right|$ and fraction ${\tilde{G}}_{i}=\left|G_{i}^{*}\right|/G_{i}\approx \prod_{j=1}^{s}{\tilde{G}}_{ij}$.

Due to the spatial self- and cross-correlations, state multiple exclusions occur for a given microscopic configuration of the statistical ensemble (as shown in Figure 13). Then, the total fraction of states excluded for a given species *i* by the other species is not merely $\sum_{j=1}^{s}\left[1-{\tilde{G}}_{ij}\left(n_{j}\right)\right]=\sum_{j=1}^{s}{\tilde{E}}_{ij}$, with ${\tilde{E}}_{ij}$ being the fraction of states of *i* excluded by *j* at $n_{j}$. Accordingly, the fraction of states for a particle of species *i* when all the species are coexisting—namely, ${\tilde{G}}_{i}$—corresponds to the ratio between the number of states in the intersection sets $G_{i}^{*}=\cap_{j=1}^{s}G_{ij}$ and the total number of states $G_{i}$. Ultimately, the total fraction of states for a single particle of species *i* at occupation n of all the species is given by ${\tilde{G}}_{i}\equiv {\tilde{G}}_{i}\left(n\right)=\lim_{G_{i}\to \infty ,N_{j}/G_{j}\to n_{j}\;\forall j\neq i}\left|G_{i}^{*}\right|/G_{i}$, which can be approximated by ${\tilde{G}}_{i}\approx \prod_{j=1}^{s}{\tilde{G}}_{ij}$.

It is worth noting that the fraction ${\tilde{G}}_{ij}$ can also be interpreted as the probability for a state of species *i* being non-excluded by particles of species *j*. Accordingly, ${\tilde{G}}_{i}\left(n\right)=\left|G_{i}^{*}\right|/\left|G_{i}\right|$ represents the probability that a state of species *i* is simultaneously non-excluded by all species. Then, assuming as a first approximation that the pairwise cross-exclusion events are independent, ${\tilde{G}}_{i}\left(n\right)$ can be written as

(240) $${\tilde{G}}_{i}\left(n\right)\approx \prod_{j=1}^{s}{\tilde{G}}_{ij}\;i=1,\ldots ,s,$$

where the functions ${\tilde{G}}_{ij}$ are given by Equation (239).

In order to finally obtain ${\tilde{d}}_{i}\left(n\right)$ as defined in Equation (234) from ${\tilde{G}}_{i}\left(n\right)$ in Equation (240), and denoting ${\tilde{G}}_{i0}\equiv {\tilde{G}}_{i}\left(n_{i}\to 0\right)=\prod_{j\neq i}{\tilde{G}}_{ij}$, it is required that ${\tilde{d}}_{i}\left(n\right)$ satisfies ${\tilde{d}}_{i}\left(n_{i}\to 0\right)={\tilde{G}}_{i0}$ and ${\tilde{d}}_{i}\left(n_{i,m}^{*}\right)=0$ (note that this is a restrictive condition implying that the entropy of species *i* vanishes at its saturation $n_{i,m}^{*}$; a more general condition ${\tilde{d}}_{i}\left(n_{i,m}^{*}\right)={\tilde{d}}_{s,i}>0$ is discussed in Section 7.8.3), where $n_{i,m}^{*}=n_{i,m}^{*}\left(n\right)\geq n_{i}$ is the maximum occupation of species *i* when other species are at densities $n_{j}$.

Introducing normalization constants $C_{i,1}$ and $C_{i,2}$ such that ${\tilde{G}}_{ii}\left(n_{i}\right)=C_{i,1}e^{\beta_{cii}n_{i}}-C_{i,2}\Delta_{ii}n_{i}$, and using ${\tilde{d}}_{i}\left(n\right)=\prod_{j=1}^{s}{\tilde{G}}_{ij}\left(n_{j}\right)=\left(\prod_{j\neq i}{\tilde{G}}_{ij}\left(n_{j}\right)\right){\tilde{G}}_{ii}\left(n_{i}\right)$, the boundary conditions are fulfilled by setting $C_{i,1}=1$ and $C_{i,2}=e^{\beta_{cii}n_{i,m}^{*}}/n_{i,m}^{*}$. Then, the density of states is

(241) $${\tilde{d}}_{i}\left(n\right)=\prod_{j\neq i}^{s}{\tilde{G}}_{ij}\left(n_{j}\right)\left(e^{\beta_{cii}n_{i}}-e^{\beta_{cii}n_{i,m}^{*}}\frac{n_{i}}{n_{i,m}^{*}}\right),$$

for i=1,…,s, with ${\tilde{G}}_{ij}\left(n_{j}\right)$ given by Equation (239).

The Helmholtz free energy for the generalized mixture on spatially correlated states can now be computed from Equations (234), (239) and (241), providing the full thermodynamic behavior.

As discussed in the section on applications, the quantities $n_{i,m}^{*}$ can be determined a priori in model systems such as *k*-mer mixtures on regular lattices from the system symmetry (see Ref. [19] for a general procedure to determine $n_{i,m}^{*}\left(n\right)$).

Finally, note that the set cardinalities in this derivation do not depend on specific microstates, but represent effective values for configurations at equilibrium minimizing the Helmholtz free energy at given *T* and *V* (i.e., given $G_{i}$).

The parameters $\beta_{cij}$ can be consistently determined from particle and lattice properties (e.g., size, shape, connectivity), as well as from the thermodynamic boundary conditions, by generalizing the analysis developed introduced for single-component systems [18].

### 6.2. Statistical Thermodynamics of Mixtures

From the Helmholtz free energy in Equation (234), we derive the density dependence of the chemical potential βμ:

(242) $$\beta \mu_{i}\left(n\right)={\left(\frac{\partial \beta F(N)}{\partial N_{i}}\right)}_{N_{j\neq i},T,V}={\left(\frac{\partial \beta f_{i}\left(n\right)}{\partial n_{i}}\right)}_{n_{j\neq i},T}.$$

Consequently, from Equations (234) and (242),

(243) $$\begin{array}{c} \beta \mu_{i}\left(n\right)=\beta \epsilon_{i}+\partial_{i}{\tilde{d}}_{i}\ln{\tilde{d}}_{i}+\ln n_{i}-\left(\partial_{i}{\tilde{d}}_{i}+1\right)\ln\left({\tilde{d}}_{i}+n_{i}\right)\\ +\sum_{j\neq i}^{s}\Delta_{ji}\left[\partial_{i}{\tilde{d}}_{j}\ln{\tilde{d}}_{j}-\partial_{i}{\tilde{d}}_{j}\ln\left({\tilde{d}}_{j}+n_{j}\right)\right]\;i=1,\ldots ,s, \end{array}$$

where, for the sake of shortness, the explicit dependence of ${\tilde{d}}_{i}$ on n is implicit in Equation (243) and $\partial_{i}\equiv {\left(\partial /\partial n_{i}\right)}_{n_{j\neq i},T}$. We can write Equation (243) more conveniently as

(244) $$K_{i}\left(T\right)\;e^{\beta \mu_{i}}=\frac{n_{i}{\left[{\tilde{d}}_{i}\right]}^{\partial_{i}{\tilde{d}}_{i}}}{{\left[{\tilde{d}}_{i}+n_{i}\right]}^{\partial_{i}{\tilde{d}}_{i}+1}}\prod_{j=1,j\neq i}^{s}{\left[\frac{{\tilde{d}}_{j}}{{\tilde{d}}_{j}+n_{j}}\right]}^{\Delta_{ji}\partial_{i}{\tilde{d}}_{j}},$$

with $K_{i}\left(T\right)=q_{i}\;e^{-\beta \epsilon_{i}}$ for i=1,…,s, which straightforwardly give the chemical potentials $\beta \mu_{i}$ as a function of the species state occupation numbers $n=(n_{1},\ldots ,n_{s})$ or species density. Equation (244) also represents a system of *s*-coupled equations whose solutions are the species occupation numbers $n_{1},n_{2},\ldots ,n_{s}$ for given chemical potentials $\beta \mu_{1},\beta \mu_{2},\ldots ,\beta \mu_{s}$.

Defining $\omega_{i}={\tilde{d}}_{i}/n_{i}$, Equation (244) can be rewritten as

(245) $$e^{\beta (\epsilon_{i}-\mu_{i})}={\left[\omega_{i}+1\right]}^{\partial_{i}{\tilde{d}}_{i}+1}{\left[\omega_{i}\right]}^{-\partial_{i}{\tilde{d}}_{i}}\prod_{j=1,j\neq i}^{s}{\left[\frac{\omega_{j}}{\omega_{j}+1}\right]}^{-\Delta_{ji}\partial_{i}{\tilde{d}}_{j}}$$

or

(246) $$e^{\beta (\epsilon_{i}-\mu_{i})}=\left[\omega_{i}+1\right]\prod_{j=1}^{s}{\left[\frac{\omega_{j}}{\omega_{j}+1}\right]}^{-\Delta_{ji}\partial_{i}{\tilde{d}}_{j}},$$

which are the coupled equations within the ME statistics from which the equilibrium distributions $n_{i}={\tilde{d}}_{i}/\omega_{i}$ can be determined.

It is worth noting that Equation (246) reduces to Wu’s distribution for fractional exclusion statistics [15] when the species states are spatially uncorrelated. An alternative picture of Wu’s limiting case of spatially uncorrelated species here is that the numbers of self-excluded and cross-excluded states per particle are constants in Wu’s formalism [15] and density-dependent in ME statistics.

### 6.3. State Exclusion Spectrum Functions: Determination of Exclusion Correlation Parameters

This section is devoted to determining the ME parameters $\beta_{cij}$ from the thermodynamic limits of the exclusion spectrum functions, which quantify the average cumulative number of self-excluded and cross-excluded states per particle as functions of the occupation numbers n. For model systems where both the size/shape of the particles and the spatial distribution of accessible states (e.g., the lattice geometry) are known, the values of $\beta_{cij}$ can be determined within the ME statistics framework, enabling a complete thermodynamic description.

Assume that species i=1,…,s in volume *V* can exchange particles with a reservoir at temperature *T* and chemical potentials $\mu_{1},\ldots ,\mu_{s}$. The time evolution of the mean occupation number $n_{i}$ follows:

(247) $$\frac{dn_{i}}{dt}=P_{io}W_{i,o\to •}-P_{i•}W_{i,•\to o},\;i=1,\ldots ,s,$$

where $P_{io}$ and $P_{i•}$ denote the average fractions of empty and occupied states for species *i*, and $W_{i,o\to •}$ and $W_{i,•\to o}$ are the respective transition rates. At equilibrium, $dn_{i}/dt=0$ and the detailed balance condition gives $W_{i,o\to •}/W_{i,•\to o}=P_{i•}/P_{io}=e^{\beta (\mu_{i}-\epsilon_{i})}$. As $P_{i•}\left(n\right)=n_{i}$, by Equation (244), we have $P_{io}=n_{i}e^{-\beta (\mu_{i}-\epsilon_{i})}.$ Substituting from Equation (244), we obtain:

(248) $$P_{io}=\frac{{\left[{\tilde{d}}_{i}+n_{i}\right]}^{\partial_{i}{\tilde{d}}_{i}+1}}{{\left[{\tilde{d}}_{i}\right]}^{\partial_{i}{\tilde{d}}_{i}}}\prod_{j\neq i}^{s}{\left[\frac{{\tilde{d}}_{j}+n_{j}}{{\tilde{d}}_{j}}\right]}^{\Delta_{ji}\partial_{i}{\tilde{d}}_{j}}.$$

The generalized exclusion spectrum function is defined as $E_{i}\left(n\right)=1-P_{io}\left(n\right)$, which measures the total average fraction of states excluded to a particle of species *i* at given occupations n (including both self- and cross-exclusion). The cumulative number of excluded states of species *i* per particle of species *j*—i.e., the spectrum function $G_{ij}\left(n\right)$—is defined as:

(249) $$G_{ij}\left(n\right)=\frac{E_{i}\left(n\right)}{n_{j}}=\frac{1-P_{io}\left(n\right)}{n_{j}}.$$

Additionally, the rate of excluded states per particle due to self- or cross-exclusion is given by the partial derivatives:

(250) $$e_{ij}\left(n\right)={\left|\frac{\partial E_{i}\left(n\right)}{\partial n_{j}}\right|}_{n_{l}\neq n_{j}}=-{\left|\frac{\partial P_{io}\left(n\right)}{\partial n_{j}}\right|}_{n_{l}\neq n_{j}}.$$

Let $e_{oii}=\lim_{n_{i}\to 0,n_{j\neq i}=0}e_{ii}\left(n\right)$, $e_{oji}=\lim_{n_{i}\to 0,n_{l\neq i}=0}e_{ji}\left(n\right)$, and $e_{oi}=\lim_{n_{i}\to 0,n_{j\neq i}=0}e_{i}\left(n\right)$, which yield:

(251) $$e_{oi}=e_{oii}+\sum_{j\neq i}\Delta_{ji}e_{oji}.$$

From Equations (248) and (250), it can be shown that

(252) $$e_{oii}=2\beta_{ii}e^{-\beta_{cii}/\beta_{ii}}+2\beta_{cii}-1.$$

Solving for the self-exclusion parameters $\beta_{cii}$,

(253) $$\beta_{cii}=\frac{1}{2}[1+e_{oii}+2\beta_{ii}W\left(-e^{-\frac{e_{oii}}{2\beta_{ii}}-\frac{1}{2\beta_{ii}}}\right)],$$

where W refers to Lambert function (see Section 5.4).

For the cross-exclusion case,

(254) $$\begin{array}{cc} e_{oij} & =\beta_{cij}+\beta_{ij}\left(e^{-\beta_{cij}/\beta_{ij}}-{\tilde{G}}_{ij,m}\right)\\ \; & \;+\Delta_{ji}\left[\beta_{cji}+\beta_{ji}\left(e^{-\beta_{cji}/\beta_{ji}}-{\tilde{G}}_{ji,m}\right)\right]. \end{array}$$

For uncorrelated states ($\beta_{cij}=0$), $e_{oii}=2g_{ii}-1$ and $e_{oij}=\beta_{ij}+\Delta_{ji}\beta_{ji}$, as in Wu’s formalism [15].

The exclusion spectrum functions $G_{ij}\left(n\right)$ are related to the density and chemical potential through

(255) $$G_{ii}\left(n\right)=\frac{1}{n_{i}}-\frac{1}{e^{\beta (\mu_{i}-\epsilon_{i})}},\;G_{ij}\left(n\right)=\frac{1}{n_{j}}-\frac{n_{i}}{n_{j}e^{\beta (\mu_{i}-\epsilon_{i})}}.$$

Although this formulation links measurable thermodynamic quantities to exclusion spectrum functions, its experimental applicability—especially for mixtures—requires further work. In discussing applications further on (see Section 7.8), we illustrate how $e_{oii}$ and $e_{oij}$ can be computed for *k*-mers on a square lattice, their behavior through the *k*-mer transitions, and their usefulness in displaying and characterizing the order of transitions.

### 6.4. The k-mers Problem as a Mixture Model: Basic Definitions

Preliminary to applications of ME statistics (as discussed in Section 7.8), we introduce the problem of *k*-mers on square lattice of *M* sites rationalized as a mixture of two differently oriented species.

As discussed in Section 5.5, the adsorption of straight rigid *k*-mers onto square lattices for k≥7 exhibits two distinct phase transitions: (1) a continuous, entropy-driven isotropic-to-nematic (I-N) transition occurring at intermediate surface coverage [124,129,131], and (2) a nematic-to-isotropic (N-I) transition taking place at densities approaching lattice saturation [133].

In Ref. [18] *k*-mers on the square lattice were modeled as a binary mixture of species aligned along the horizontal (H) and vertical (V) lattice directions, denoted as species 1 and 2, respectively. Both species occupy *k* consecutive lattice sites along their respective directions. According to our definitions, $G_{1}=G_{2}=M$, $\Delta_{12}=\Delta_{21}=1$, $N_{1,m}=N_{2,m}=M/k$, and $n_{1,m}=n_{2,m}=1/k$. The exclusion parameters are $\beta_{11}=\beta_{22}=\beta_{12}=\beta_{21}=k$, and the saturation values satisfy ${\tilde{G}}_{12,m}={\tilde{G}}_{21,m}=0$.

By Equation (241), the saturation occupations under coexistence are:

(256) $$n_{1,m}^{*}\left(n_{2}\right)=\frac{1}{k}-n_{2},\;n_{2,m}^{*}\left(n_{1}\right)=\frac{1}{k}-n_{1}.$$

The self-exclusion at infinite dilution is $e_{o11}=e_{o22}=2k-1=2\beta_{11}-1$, leading to $\beta_{c11}=\beta_{c22}=0$. For cross-exclusion, each *k*-mer excludes $k^{2}$ states orthogonal to its direction, of which 2k−1 are shared. Thus, $e_{o12}=e_{o21}=k^{2}-\left(2k-1\right)={\left(k-1\right)}^{2}$.

Using Equation (254), the cross-exclusion correlations are

(257) $$\beta_{c12}=\beta_{c21}=\frac{1}{2}\left[e_{o12}+2kW\left(-e^{-e_{o12}/2k}\right)\right],$$

where W is the Lambert function. Solving this yields $\beta_{c12}=11.63$ for k=6, $\beta_{c12}=17.41$ for k=7, $\beta_{c12}=24.10$ for k=8, and $\beta_{c12}=84.26$ for k=14.

Based upon this elementary definition of the mixture parameter, as well as the thermodynamic and exclusion functions in the ME statistics, a comprehensive treatment of the problem is given in Section 7.8, leading to the entropy surface, equilibrium paths, density branches, order parameters, transition critical points, and state exclusion spectrum for various values of *k*.

## 7. Applications

In this section, we analyze the scope and limitations of the theoretical models developed in previous sections through comparison with Monte Carlo simulations and experimental data available in the literature.

### 7.1. Two-Dimensional Adsorption: Comparison Between Theory and Monte Carlo Simulations

In this section, adsorption isotherms are calculated for the theoretical models introduced in Section 3 (i.e., FH, GD, EA, FSTA, and SE) and compared both among themselves and against Monte Carlo simulations performed within the grand canonical ensemble framework [see Section 8]. These comparisons are conducted for honeycomb, square, and triangular lattices.

Monte Carlo simulations were carried out on honeycomb, square, and triangular lattices of size L×L, with L=144,144, and 150, respectively, using periodic boundary conditions. This lattice size ensures that finite-size effects are negligible. In addition, $m^{\prime }=m=10^{6}$ MCSs were performed.

**Figure 14:**
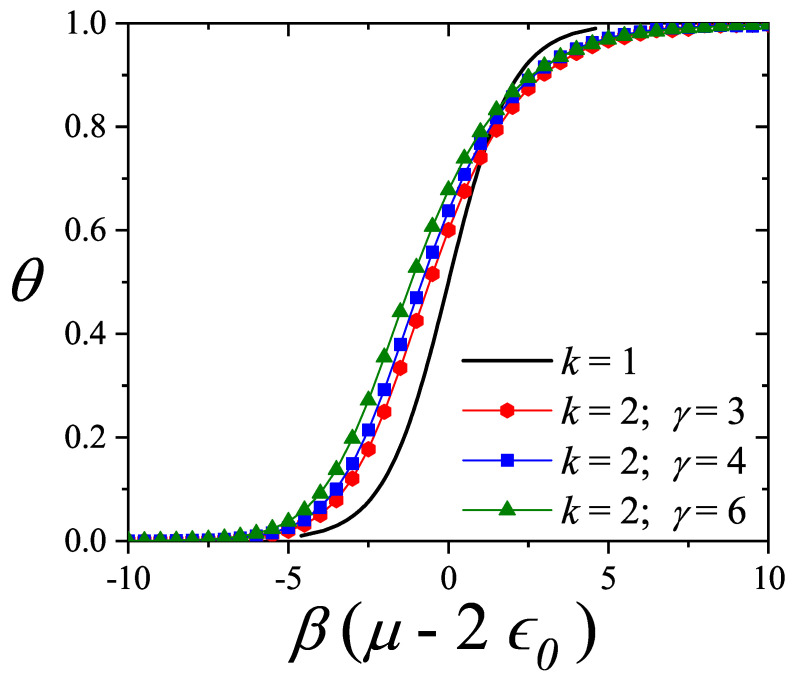
Comparison between the exact adsorption isotherm of monomers and the simulation adsorption isotherms of dimers on honeycomb, square, and triangular lattices.

We begin by discussing some fundamental features of the adsorption isotherms. Figure 14 presents a comparison between the exact adsorption isotherm for monomers and the simulated isotherms for dimers on honeycomb, square, and triangular lattices. As observed, the particle–vacancy symmetry—which holds for monoatomic adsorbates—breaks down when k≥2. Furthermore, while the dimer adsorption isotherms appear similar across the different lattice types, the curves shift to lower values of $\beta (\mu -2\epsilon_{0})$ as the connectivity γ increases. In other words, for a given value of $\beta (\mu -2\epsilon_{0})$, the equilibrium surface coverage rises with increasing γ. This behavior can be explained using the following relation:

(258) $$\ln\theta =\ln\gamma +\beta \left(\mu -k\epsilon_{0}\right),$$

which is valid for linear *k*-mers at low concentrations [see Equation (72)]. As the chemical potential increases, this effect diminishes and, consequently, the slope of the isotherms decreases with increasing γ.

We now consider the case of linear adsorbates larger than dimers. For honeycomb lattices, *k*-mers adsorb as described in Section 3.1.1. When a site is selected, there are six possible equilibrium orientations for a single *k*-mer (k≥2) at extremely low coverage, resulting in a total number of *k*-tuples equal to 3M (i.e., γ=6), as is also the case for triangular lattices.

**Figure 15:**
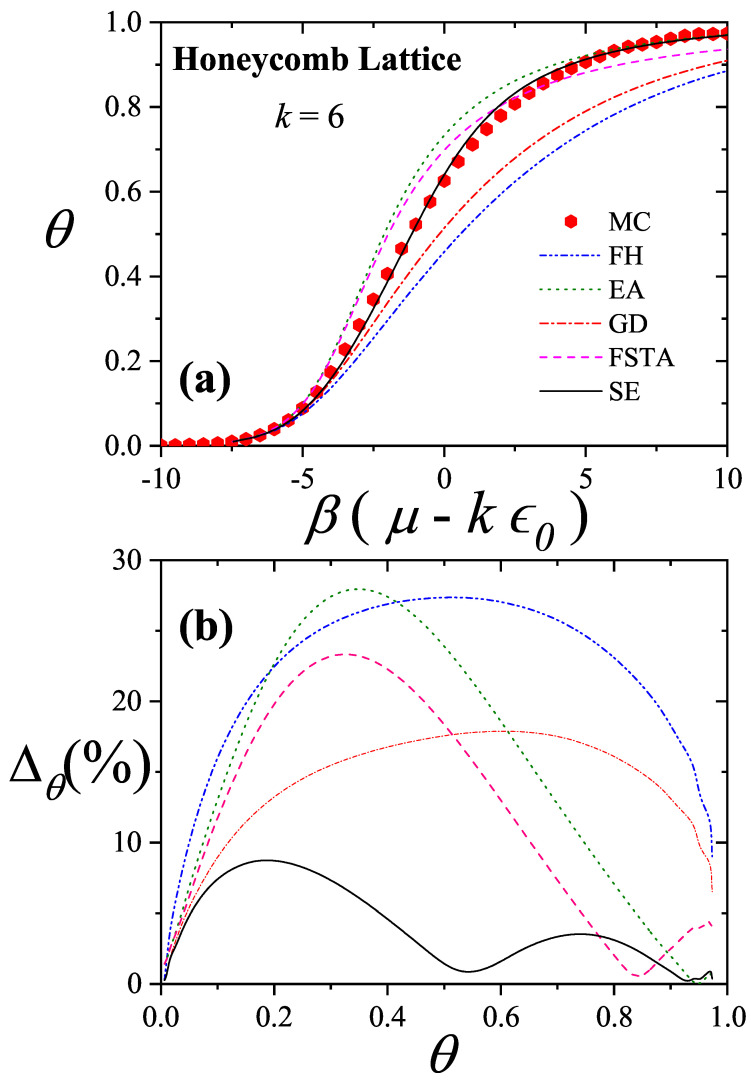
(**a**) Adsorption isotherms of 6-mers on a honeycomb lattice. Symbols represent MC results and lines correspond to different approaches (see inset). (**b**) Percentage reduced coverage, $\Delta_{\theta }\left(\%\right)$, versus surface coverage. The symbols are defined as in part (**a**).

**Figure 16:**
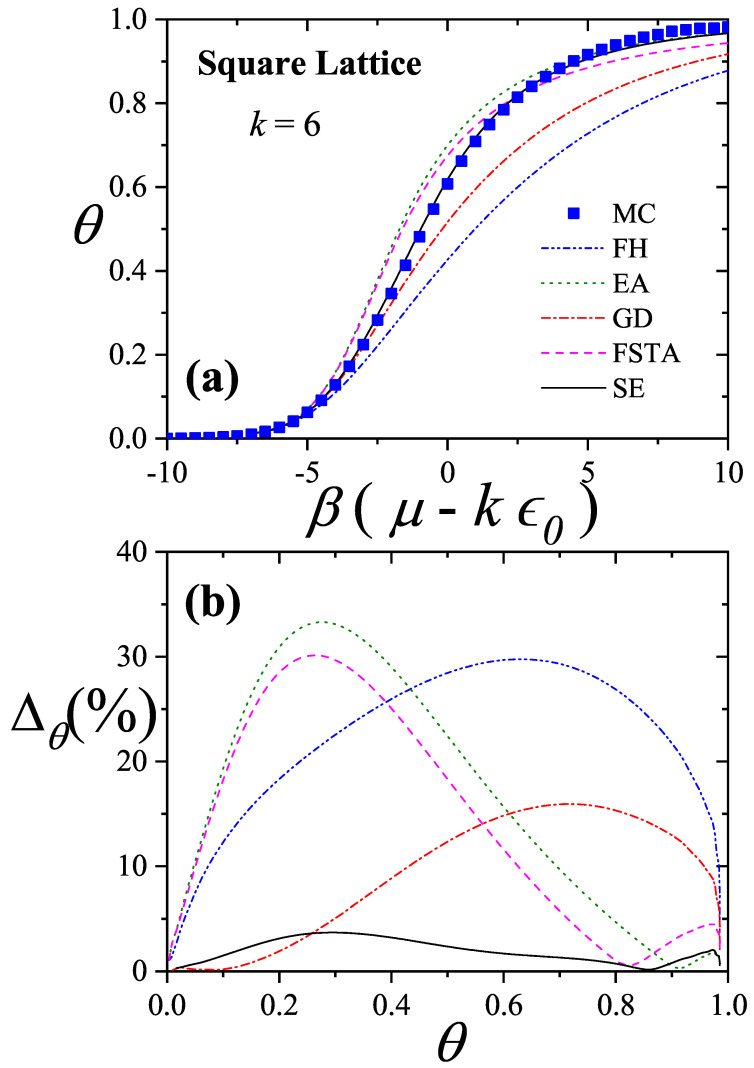
Similar to Figure 15, but for a square lattice.

**Figure 17:**
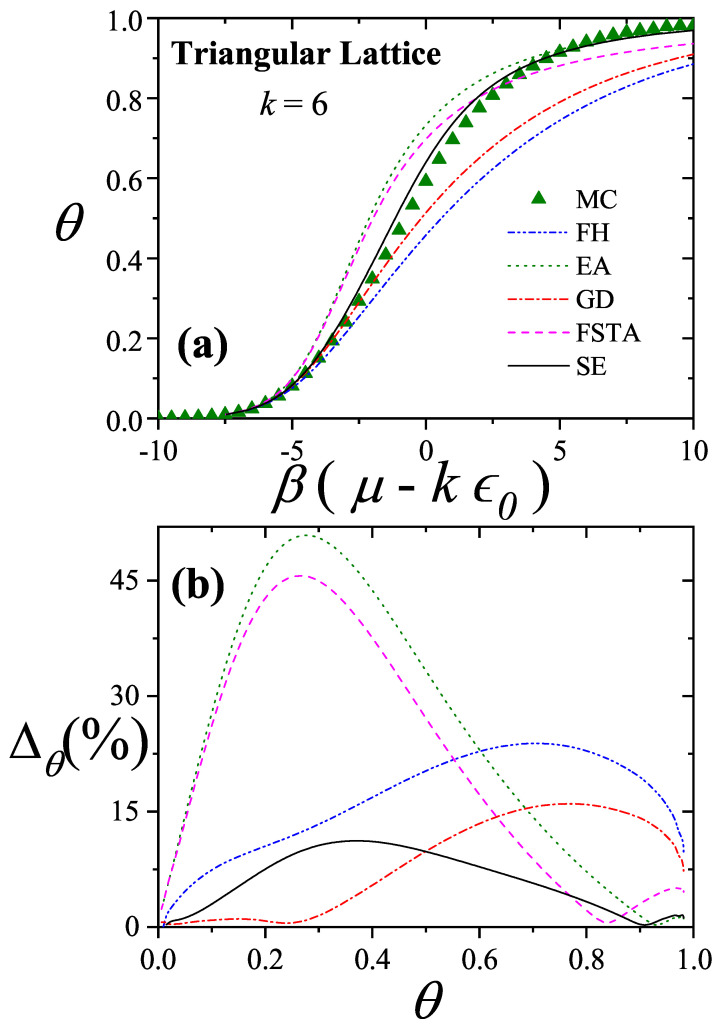
Similar to Figure 15, but for a triangular lattice.

Based on these conditions, extensive simulations were conducted for linear adsorbates with *k* ranging from 2 to 10. As illustrative examples, Figure 15a, Figure 16a, Figure 17a compare simulation isotherms with theoretical predictions for 6-mers on honeycomb, square, and triangular lattices, respectively. In all cases, the theoretical models agree well with simulations at low coverages, but deviate significantly as the surface coverage increases.

The discrepancies between simulation and theory can be quantified by the percentage reduced coverage, defined as [93]

(259) $$\Delta_{\theta }\left(\%\right)=100{\left|\frac{\theta_{sim}-\theta_{appr}}{\theta_{sim}}\right|}_{\mu }$$

where $\theta_{sim}$ ($\theta_{appr}$) represents the coverage obtained using MC simulation (analytical approach). Each pair of values ($\theta_{sim},\theta_{appr}$) is obtained at fixed μ.

**Figure 18:**
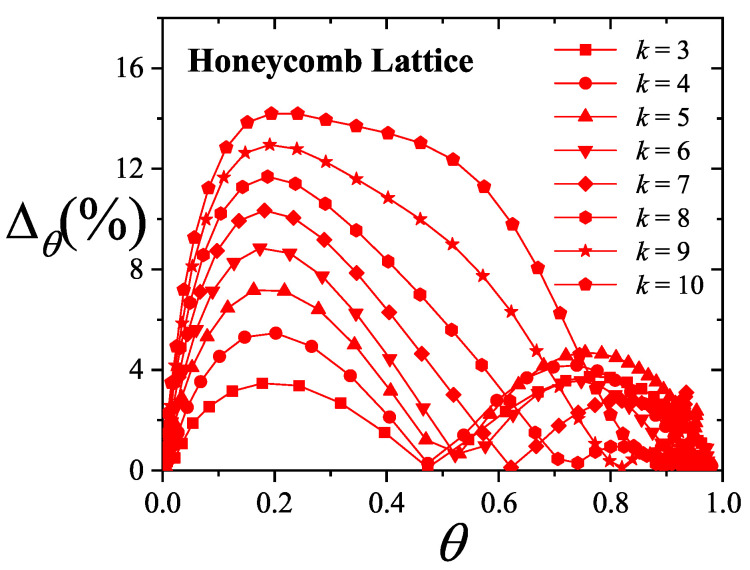
Percentage reduced coverage versus concentration for *k*-mers adsorbed onto a honeycomb lattice and SE approximation. Symbols are indicated in the inset.

**Figure 19:**
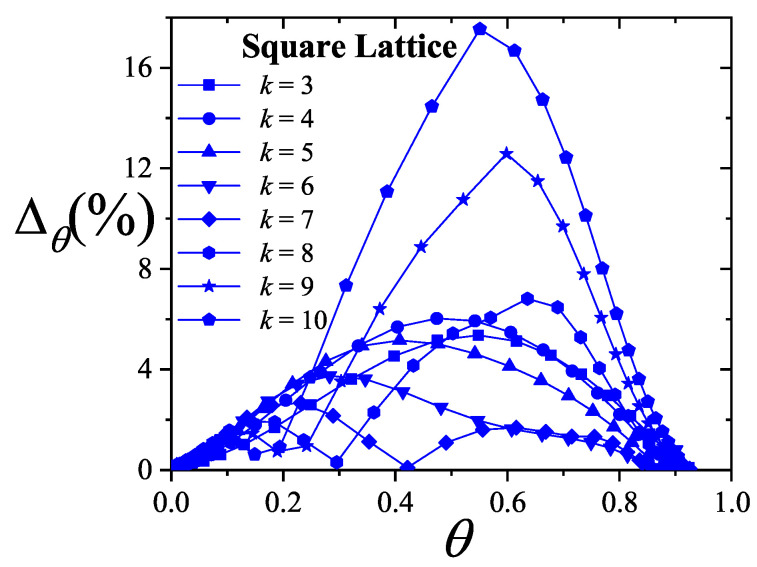
Similar to Figure 18, but for a square lattice.

**Figure 20:**
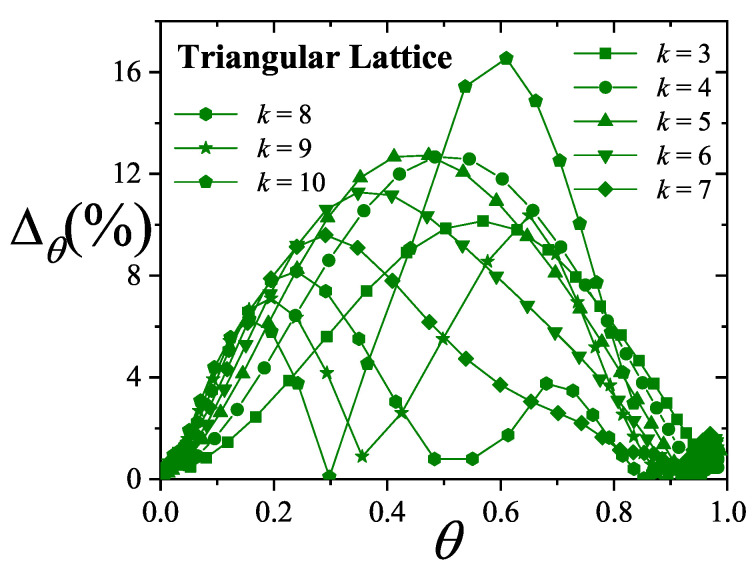
Similar to Figure 18, but for a triangular lattice.

Figure 15b, Figure 16b, Figure 17b show how $\Delta_{\theta }\left(\%\right)$ varies with surface coverage for the different lattice types. The performance of each theoretical model is as follows: the FSTA model (dashed line) shows very good agreement with simulation results, with minimal discrepancies. Both the FH (dash-dot-dot line) and GD (dash-dot line) models tend to underestimate the coverage across the entire range. The EA model (dotted line) performs poorly at intermediate coverages but improves at high coverages. With respect to lattice connectivity, the accuracies of EA and FSTA improve as γ decreases, while the opposite trend is observed for FH and GD. The behavior of the GD and EA models supports the formulation of the semi-empirical (SE) isotherm (solid line) given in Equation (78). This trend is also illustrated in Figure 18, Figure 19 and Figure 20, which show the percentage reduced coverage for the SE model as a function of concentration for various values of γ and *k*.

**Figure 21:**
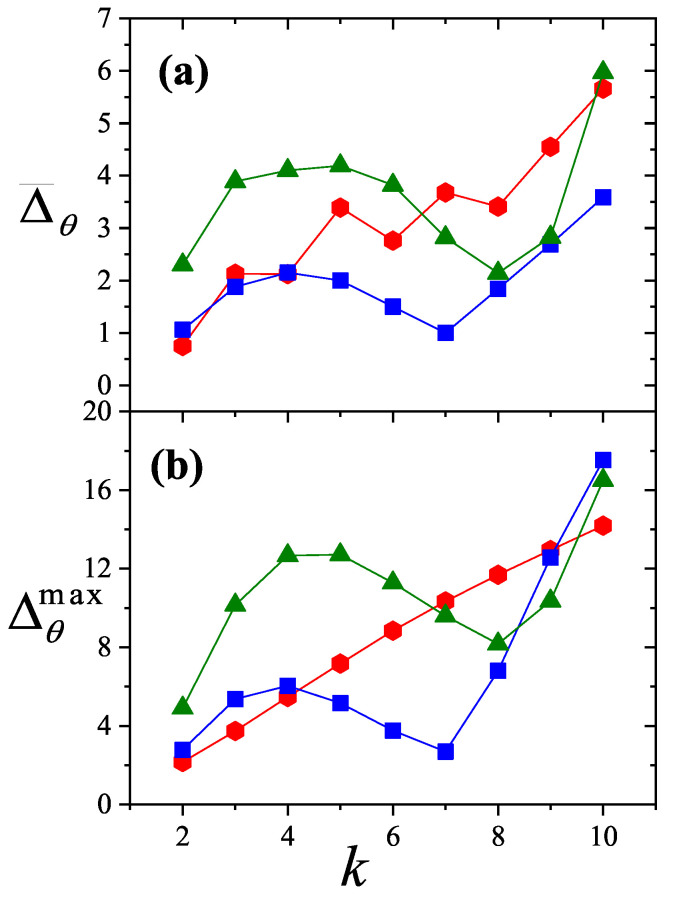
(**a**) Average percentage reduced coverage, ${\bar{\Delta }}_{\theta }$, as a function of *k* for different connectivities. (**b**) As in part (**a**) for the maximum percentage reduced coverage $\Delta_{\theta }^{\max}$. Hexagons, squares, and triangles correspond to data obtained for honeycomb, square, and triangular lattices, respectively.

To better interpret the data shown in Figure 18, Figure 19 and Figure 20, we consider two summary metrics: (1) the average absolute difference between simulation and theoretical coverage, $\bar{\Delta }\theta$; and (2) the maximum value of the percentage reduced coverage, $\Delta \theta^{\max}$. These metrics are presented in Figure 21. Several conclusions can be drawn: (i) the theoretical models generally perform better on square lattices; (ii) both $\bar{\Delta }\theta$ and $\Delta \theta^{\max}$ remain nearly constant for *k* values between 2 and 8; and (iii) both quantities increase for k>8. Finally, as the values of ${\bar{\Delta }}_{\theta }$ remain below 6%, the SE model can be considered a reliable approximation for describing multisite occupancy adsorption, at least for the *k* values analyzed here.

### 7.2. Two-Dimensional Adsorption of Binary Mixtures: Comparison Between Theory and Monte Carlo Simulations

In this section, we analyze the main characteristics of the theoretical approximations developed in Section 3.2 in comparison with MC simulation results and experimental data.

We consider a gas mixture composed of rigid rods of lengths *k* and *l*, with each component present in equal molar fraction in the gas phase. Adsorption occurs on a regular, homogeneous lattice with connectivity γ=4 (square lattice) and 6 (triangular lattice). The parameters used in the HPTMC simulations (see Section 8.3) were M=120×120 and $m^{\prime }=m=10^{6}$ MCSs. For simplicity, we assume the standard chemical potentials are zero and that the equilibrium constants are $K_{i}=1$ for all species.

**Figure 22:**
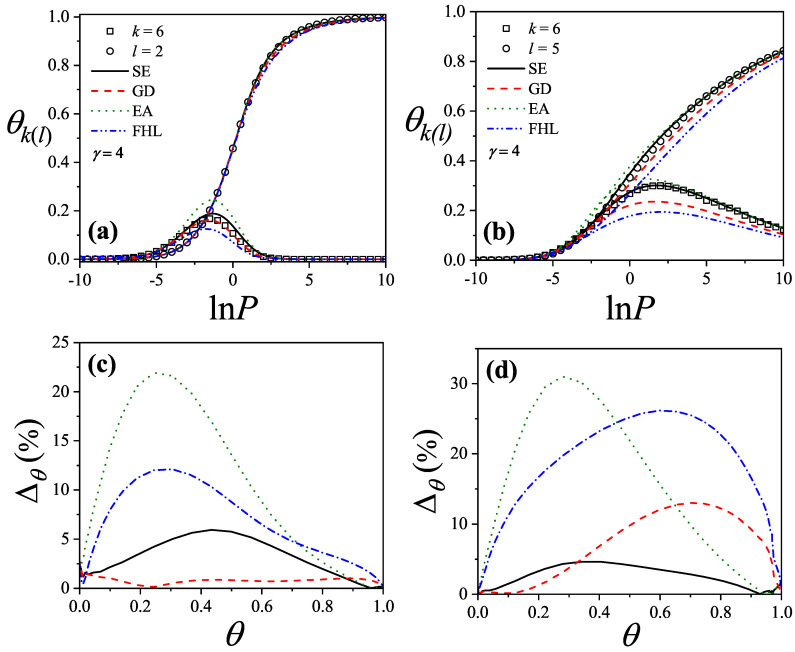
Adsorption isotherms for rigid molecules on a square lattice: (**a**) k=6 and l=2; (**b**) k=6 and l=5. (**c**,**d**) Reduced coverage error Δθ(%) vs. the total surface coverage for the data in part (**a**,**b**). Symbols represent MC results, and lines correspond to different theoretical approaches as indicated.

The partial adsorption isotherms for γ=4, k=6, and two values of l=(2,5) are shown in Figure 22a and Figure 22b, respectively. Symbols denote MC simulation data, while the lines represent various theoretical approaches as indicated.

A characteristic feature observed in binary mixtures of polyatomic species is evident in both figures: at higher pressures, the smaller species are displaced by the larger ones. This phenomenon—known as adsorption preference reversal (APR)—arises from entropic competition between adsorbed species. A detailed investigation of the APR effect, focusing on the impact of molecular size difference, is presented in Section 7.4.

In Figure 22a, the most significant discrepancies between simulation and theoretical predictions occur in the partial adsorption isotherm of the larger species. As clearly shown in Figure 22b, the magnitude of these deviations depends on the level of approximation used when evaluating $\tilde{C}$ [11].

To quantitatively assess the agreement between the simulation and analytical results, we use the reduced coverage error defined in previous section [93]:

(260) $$\Delta_{\theta }\left(\%\right)=100\times \left|\frac{\theta_{\mathrm{MC}}-\theta_{\mathrm{appr}}}{\theta_{\mathrm{MC}}}\right|,$$

where $\theta_{\mathrm{MC}}$$\left(\theta_{\mathrm{appr}}\right)$ represents the value of the total coverage obtained via MC simulation (analytical approach). Each pair ($\theta_{\mathrm{MC}}$, $\theta_{\mathrm{appr}}$) corresponds to the same pressure value *P*.

The results obtained from Equation (260) are shown in Figure 22c,d. In Figure 22c, better agreement is observed at surface coverage above θ≈0.6, where only dimers are present—a case where all theoretical models perform well [16]. Conversely, for larger adsorbates, classical approaches fail to accurately describe the adsorption behavior across the entire range of coverage, as illustrated in Figure 22d. In contrast, the SE approximation yielded satisfactory results, with errors remaining below 7% in both cases.

**Figure 23:**
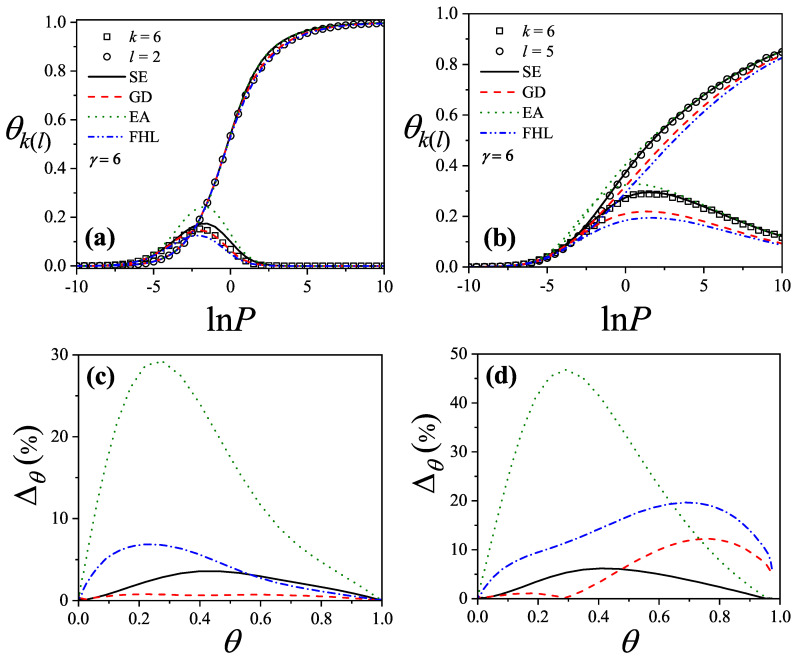
Similar to Figure 22, but for triangular lattices.

The same analysis presented in Figure 22 is repeated for triangular lattices, with the corresponding results shown in Figure 23. In most cases, the error increases with lattice connectivity. However, the SE approximation consistently yielded an error below 7%, indicating that it is a highly reliable method for modeling binary mixture adsorption with multisite occupancy, at least for the molecular sizes considered in this study.

To complete our analysis, we examine partial isotherms for mixtures with varying sizes of *l*, keeping k=6 fixed. For each pair (*k*, *l*), the discrepancies between theoretical predictions and simulation results are assessed using the average error across the full coverage range, defined as

(261) $${\bar{\Delta }}_{\theta }=\frac{1}{R}\sum_{\theta }\Delta_{\theta }\left(\%\right),$$

where *R* is the total number of isotherm points (or the number of replicas, as described in Section 8.3).

**Figure 24:**
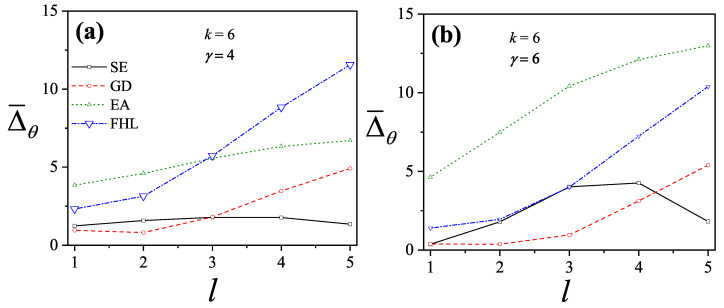
Average error ${\bar{\Delta }}_{\theta }$ for fixed *k* and different values of *l*: (**a**) square lattice, γ=4; and (**b**) triangular lattice, γ=6. The meaning of the lines is indicated in the figure.

Figure 24 illustrates the dependence of ${\bar{\Delta }}_{\theta }$ on the size of the *l*-mers and the lattice geometry for the various theoretical approaches evaluated in this work. As shown, ${\bar{\Delta }}_{\theta }$ increases monotonically with *l*, indicating that the divergence between MC simulations and analytical models becomes more significant for larger adsorbates. In contrast, the error associated with the SE approximation remains nearly constant: around 1.8% for square lattices and 4.3% for triangular lattices. This excellent agreement across all values of *l* highlights the robustness of the SE method and underscores the importance of accurately computing the correction function [Equation (115)] for understanding the adsorption behavior of rigid rod mixtures.

Finally, to evaluate the applicability of the proposed model, we analyzed experimental data extracted from Ref. [139]. Specifically, adsorption isotherms of hydrocarbon mixtures—methane–ethane and ethane–propylene—on activated carbon (AC-40) at ${20\;}^{\circ }$C were examined using the SE adsorption model introduced in this work. As the experimental data were reported as adsorbed amounts (in moles/g) versus pressure (in mmHg), the theoretical isotherms were reformulated in terms of pressure *P* and adsorbed amount *g* to enable direct comparison and fitting.

Assuming equilibrium between the adsorbed phase and an ideal gas-phase mixture, the chemical potentials $\mu_{k(l)}$ were related to the system pressure and molar fractions. Additionally, the coverage was defined as $\theta =g/g_{max}$, where $g_{max}$ represents the maximum adsorption capacity of the surface.

Following a common approach in the literature, a “bead segment” chain model was employed, in which each ${\mathrm{CH}}_{n}$ group (bead) occupies one adsorption site on the surface. Accordingly, each hydrocarbon species $C_{m}$ was modeled as a rigid rod of length k(l)=m [140].

**Figure 25:**
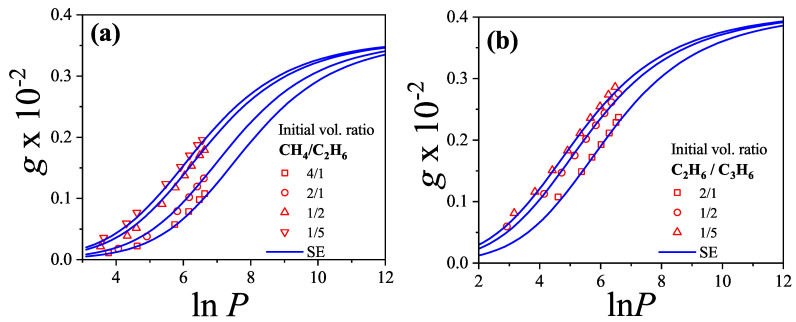
Comparison between experimental and theoretical adsorption isotherms for a binary mixture: (**a**) CH4/C2H6 and (**b**) C2H6/C3H6 adsorbed onto commercial activated carbon. Symbols represent experimental data from Ref. [139] and lines correspond to results from Equation (121). The parameters used in the fitting procedure are listed in Table 1.

Within this framework, the experimental isotherms for methane–ethane and ethane–propylene mixtures at ${20\;}^{{}^{\circ}}$C and varying molar fractions were fitted using a single value of $g_{max}$ and temperature-dependent equilibrium constants $K_{k(l)}\left(T\right)$ as adjustable parameters. The results of the fitting procedure are shown in Figure 25, and the corresponding parameter values are summarized in Table 1. A very good agreement can be observed between experimental data (symbols) and theoretical predictions (solid lines).

While a more extensive analysis of experimental adsorption isotherms is still needed, these results suggest that the SE theory provides a promising and accurate framework for describing the adsorption thermodynamics of interacting polyatomic species.

### 7.3. Two-Dimensional Adsorption of Interacting k-mers: Comparison Between Theory and Monte Carlo Simulations

In this section, we analyze the main features of the thermodynamic functions derived from the models presented in Section 4.1 (Bragg–Williams Approximation, BWA) and Section 4.2 (Quasi-Chemical Approximation, QCA), in comparison with Monte Carlo simulation results for a lattice–gas of interacting dimers on honeycomb, square, and triangular lattices. (In one dimension, the QCA reduces to the exact solution for interacting chains adsorbed flat on a one-dimensional lattice. With respect to BWA, a characteristic van der Waals loop appears in the isotherm under attractive interactions, erroneously predicting a phase transition for γ=2. For strong repulsive interactions, the BWA deviates from the exact results and fails to reproduce the plateau observed in the adsorption isotherm. These limitations can be better understood through the entropy per site. The key assumption of the BWA is that both the configurational degeneracy and the average nearest-neighbor interaction energy are treated as if molecules are randomly distributed across the lattice. As a result, the entropy per site becomes independent of *w* and takes the form given by Equation (42).

As in Section 7.1, simulations were conducted on honeycomb, square, and triangular lattices of size L×L, with L=144, 144, and 150, respectively, using periodic boundary conditions. Moreover, the lattice size *L* was carefully selected to avoid perturbation of the adlayer structure.

**Figure 26:**
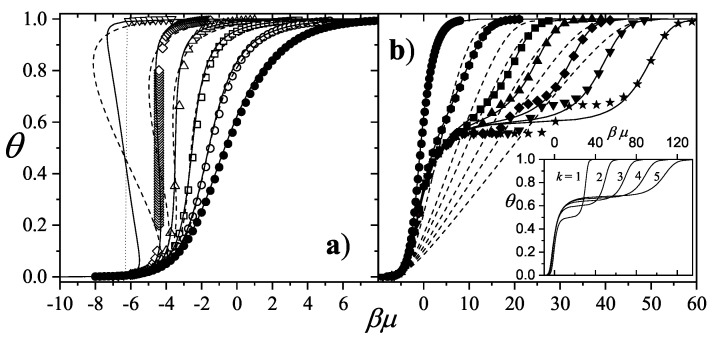
Adsorption isotherms for homonuclear dimers placed on a honeycomb lattice, taking into account nearest-neighbor interactions. The data points correspond to Monte Carlo simulations, while the solid and dashed curves represent theoretical predictions from the QCA and the BWA, respectively. Dotted lines are included for visual reference only. (**a**) Attractive interactions: filled circles denote βw=0; open circles, βw=−0.5; open squares, βw=−1.0; open upward triangles, βw=−1.5; open diamonds, βw=−2.0; and open downward triangles, βw=−3.0. (**b**) Repulsive interactions: filled circles correspond to βw=0; filled hexagons, βw=2.0; filled squares, βw=4.0; filled upward triangles, βw=5.0; filled diamonds, βw=6.5; filled downward triangles, βw=8.0; and filled stars, βw=7.0. Inset: Isotherms calculated using QCA for βw=7.0, showing various values of *k* as labeled.

**Figure 27:**
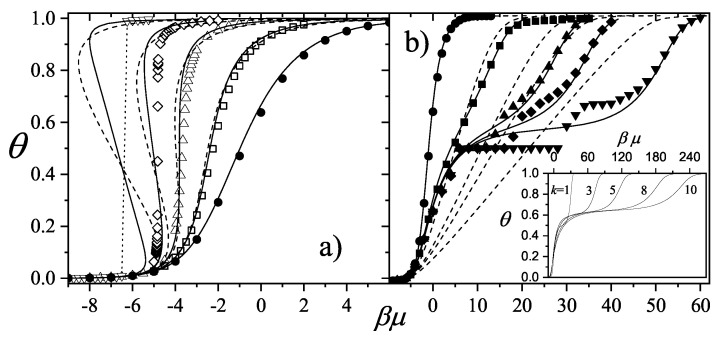
Adsorption isotherms for homonuclear dimers on a square lattice with nearest-neighbor interactions. Symbols correspond to Monte Carlo simulation data, while solid and dashed lines represent predictions from the QCA and the BWA, respectively. Dotted lines are included solely to guide the reader’s eye. (**a**) Attractive interactions: filled circles indicate βw=0; open squares, βw=−0.5; open upward triangles, βw=−1.0; open diamonds, βw=−1.4; and open downward triangles, βw=−2.0. (**b**) Repulsive interactions: filled circles represent βw=0; filled squares, βw=2.0; filled upward triangles, βw=4.0; filled diamonds, βw=5.0; and filled downward triangles, βw=7.5. Inset: QCA-derived adsorption isotherms for βw=7.5 at various values of *k*, as labeled.

**Figure 28:**
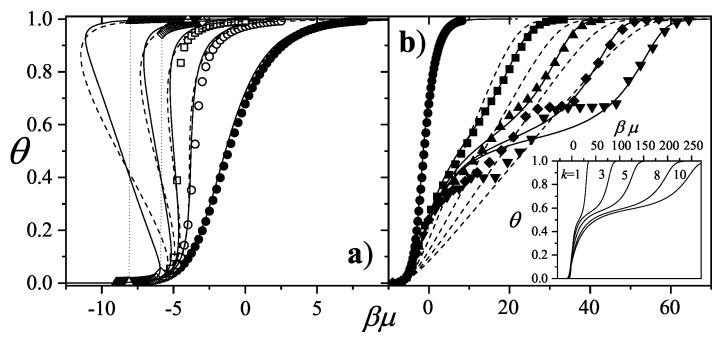
Adsorption isotherms for homonuclear dimers on a triangular lattice with nearest-neighbor interactions. Data points represent Monte Carlo simulation results, while solid and dashed curves correspond to predictions from the QCA and the BWA, respectively. Dotted lines are added to assist in visual interpretation. (**a**) Attractive interactions: filled circles indicate βw=0; open circles, βw=−0.5; open squares, βw=−0.75; open diamonds, βw=−1.0; and open upward triangles, βw=−1.5. (**b**) Repulsive interactions: filled circles represent βw=0; filled squares, βw=2.0; filled upward triangles, βw=3.0; filled diamonds, βw=4.0; and filled downward triangles, βw=5.0. Inset: QCA-calculated adsorption isotherms for βw=5.0 with different values of *k*, as labeled.

Representative adsorption isotherms obtained from Monte Carlo simulations in the grand canonical ensemble (symbols), along with their comparison to the QCA (solid lines) and BWA (dashed lines), are shown in Figure 26, Figure 27 and Figure 28 for honeycomb, square, and triangular lattices, respectively.

For attractive interactions (Figure 26a, Figure 27a, Figure 28a), a first-order phase transition occurs as temperature decreases, evidenced by the discontinuity in the simulated isotherms and the appearance of characteristic loops in the theoretical curves. This behavior, which has been experimentally observed in many systems [103], corresponds to a low-coverage lattice–gas phase coexisting with a higher-coverage “lattice–fluid” phase. The lattice–fluid can be considered as a diluted version of the registered (1×1) phase, where all lattice sites are occupied except for some vacancies.

This two-dimensional gas-to-liquid condensation closely resembles that of a monomeric lattice gas with attractive interactions. However, for *k*-mers, the particle–vacancy symmetry (valid for monomers) is broken, resulting in adsorption isotherms that are asymmetric with respect to θ=0.5.

**Figure 29:**
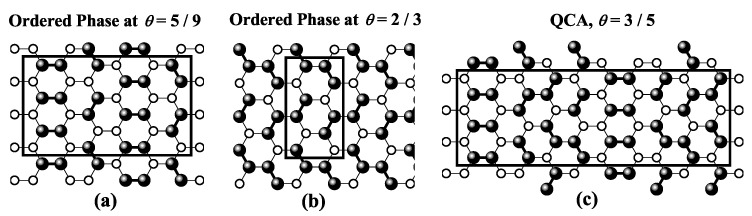
Snapshots of the ordered phases corresponding to repulsive dimers adsorbed on a honeycomb lattice. (**a**) Low-coverage ordered structure (LCOP); (**b**) high-coverage ordered structure (HCOP); and (**c**) LCOP–HCOP mixture according to the predictions of QCA.

**Figure 30:**
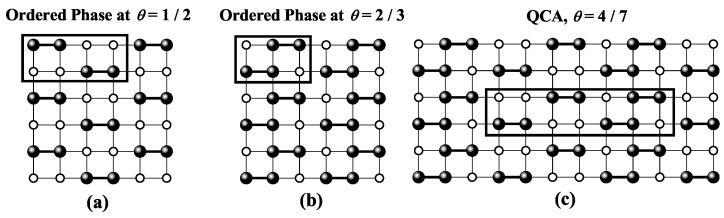
Similar to Figure 29, but for square lattices.

**Figure 31:**
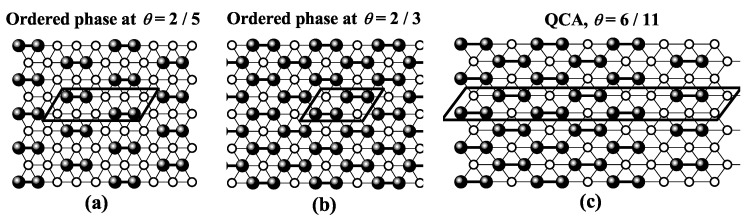
Similar to Figure 29, but for triangular lattices.

In the case of repulsive interactions (Figure 26b, Figure 27b, Figure 28b), the isotherms exhibit more complex features due to the formation of ordered structures in the adsorbed layer. These ordered arrangements are indicative of sub-critical behavior, where continuous phase transitions occur from disordered to ordered phases [141,142]. At high temperatures, isotherms remain featureless while, at low temperatures, they display distinct steps corresponding to the emergence of ordered phases. The specific form of these steps depends strongly on the lattice connectivity. As the chemical potential μ increases and the surface coverage θ spans from 0 to 1, two ordered phases are typically observed: (1) a low-coverage ordered phase (LCOP), characterized by site occupancies of 5/9, 1/2, and 2/5 for honeycomb, square, and triangular lattices, respectively; and (2) a high-coverage ordered phase (HCOP), with 2/3 site occupancy across all three geometries. Snapshots of the LCOP [part (a)] and HCOP [part (b)] configurations for each lattice type are presented in Figure 29, Figure 30 and Figure 31. For a detailed discussion of these phases, refer to Refs. [141,142].

Under attractive interactions, both theoretical models yield qualitatively similar results and the isotherms from BWA and QCA are nearly indistinguishable. However, it is known that isotherms derived from fundamentally different approximations can appear deceptively similar [73]. To better assess the accuracy of each model, we calculated the absolute error in the chemical potential, $\varepsilon^{a}\left(\theta \right)$, which is defined as

(262) $$\varepsilon^{a}\left(\theta \right)={\left|\mu_{theor}-\mu_{sim}\right|}_{\theta }$$

where $\mu_{sim}$ ($\mu_{theor}$) represents the chemical potential obtained via MC simulation (analytical approach). Each pair of values ($\mu_{sim}$, $\mu_{theor}$) is obtained at fixed θ.

**Figure 32:**
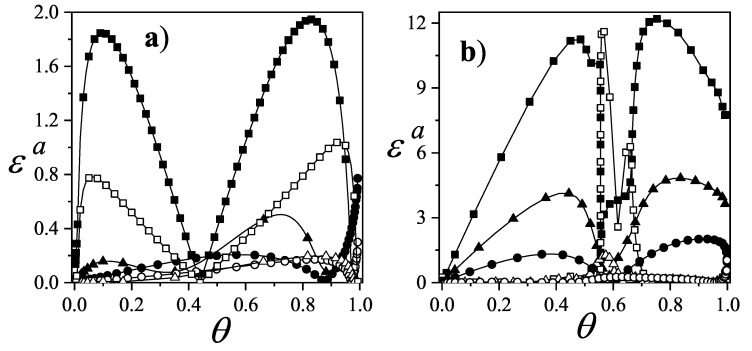
Absolute error $\varepsilon^{a}$ versus surface coverage for adsorption isotherms of dimers. The symbols are defined is as follows: (**a**) Squares, βw=−3.0; triangles, βw=−1.5; and circles, βw=−0.5. (**b**) Squares, βw=8.0; triangles, βw=4.0; and circles, βw=2.0. Full and open symbols correspond to comparisons with QCA and BWA, respectively.

As an example, Figure 32a presents $\varepsilon^{a}\left(\theta \right)$ for three representative attractive interaction strengths: squares for βw=−3.0, triangles for βw=−1.5, and circles for βw=−0.5. Solid and open symbols correspond to the BWA and QCA results, respectively. In all cases, QCA was found to outperform BWA.

The corresponding analysis for repulsive interactions is shown in Figure 32b, which includes βw=8.0 (squares), 4.0 (triangles), and 2.0 (circles). Again, solid and open symbols represent BWA and QCA, respectively. Here, the differences between the two models are both quantitative and qualitative. While BWA fails to predict any ordered structures, QCA captures the formation of a pronounced plateau at low temperature. This critical coverage, $\theta_{c}^{QCA}$, appearing between the LCOP and HCOP, depends on both the lattice geometry and adsorbate size. The adsorbate configuration at $\theta_{c}^{QCA}$ can be interpreted as a mixture of LCOP and HCOP phases (see part (c) in Figure 29, Figure 30 and Figure 31).

The curves in Figure 32 correspond to a honeycomb lattice. However, the behavior of $\varepsilon^{a}\left(\theta \right)$ for square and triangular lattices is very similar.

To quantify the overall deviation between theory and simulation across the full coverage range, we define the integral error $\varepsilon^{i}$ as:

(263) $$\varepsilon^{i}=\int_{0}^{1}\varepsilon^{a}\left(\theta \right)d\theta .$$

**Figure 33:**
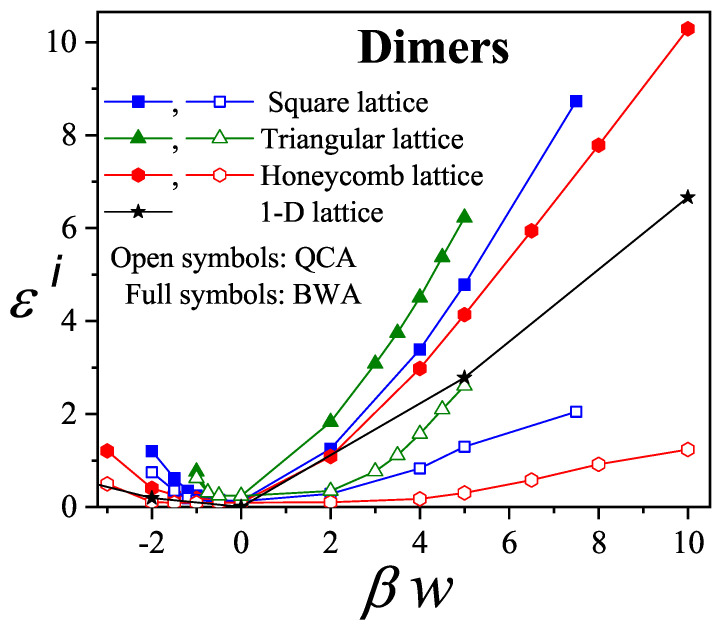
Integral error $\varepsilon^{i}$ versus lateral interaction (in β units) for different geometries, as indicated.

Figure 33 shows $\varepsilon^{i}$ for all lattice geometries and a broad range of βw values. Several key conclusions can be drawn: (1) in all cases, QCA provides a significantly better fit to the simulation data than BWA. This is particularly true for repulsive interactions, where BWA shows large discrepancies, while QCA remains the simplest yet more effective model for describing multisite occupancy adsorption. (2) The value of $\varepsilon^{i}$ increases with lattice connectivity. This may be attributed to a loss in accuracy of Ω(N,M,γ) as γ increases [93]. (3) There exists a broad range of interaction strengths (−1≤βw≤4) for which QCA matches the simulation data extremely well. Notably, most surface science experiments fall within this range of interaction energies.

Therefore, QCA not only represents a clear improvement over the BWA in modeling *k*-mer adsorption, but also provides a solid theoretical framework and compact expressions for the interpretation of thermodynamic adsorption data of polyatomic species—such as alkanes, alkenes, and other hydrocarbons—on regular surfaces.

### 7.4. Application of FSTA to the Adsorption of $C_{3}H_{8}$ and $O_{2}$ in Zeolites 13X and 5A: Determination of the Adsorption Configuration

One interesting application of the theoretical framework presented in Section 3.1.2 on a lattice gas model involves the interpretation of experimental adsorption isotherms for propane [143] and oxygen [144,145] in 13X and 5A zeolites, as well as in simulation-based systems. In our approach, we employed Equation (48) under two main assumptions: (*i*) as *g* is constant, if a single molecule has *m* distinct ways to adsorb per lattice site at zero coverage, then the presence of an adsorbed *k*-mer, occupying $k^{\prime }$ sites, effectively excludes $g=mk^{\prime }$ states from being accessible to other molecules [thus, $a=1/(mk^{\prime })$]; and (ii) the energetic contribution from adsorbate–adsorbate interactions is accounted for using a mean-field approximation, as described in Section 4.1. This analysis highlights the physical interpretation of the parameters *g* and *a*, linking them to the spatial configuration of adsorbed molecules and the geometric structure of the surface.

As the experimental data are presented as adsorbed volume *v* against pressure *p*, we can rewrite Equation (48) in a more convenient form:

(264) $$K\left(T\right)\;P/P_{0}=\frac{\left(v/v_{m}\right){\left[g-\left(g-1\right)v/v_{m}\right]}^{g-1}}{{\left[g-g\left(v/v_{m}\right)\right]}^{g}}\exp\left[\beta w\left(v/v_{m}\right)\right],$$

where $\theta =v/v_{m}$ ($v_{m}$ is the volume corresponding to monolayer completion); $\exp\left(\beta \mu \right)=P/P_{0}$; K(T) is the equilibrium constant $K\left(T\right)=\exp(-\beta \epsilon_{0})$; and $\exp\left[\beta w\left(v/v_{m}\right)\right]$ is the mean-field term. In addition, $\epsilon_{0}$ can be associated with the isosteric heat of adsorption $H_{st}$.

**Figure 34:**
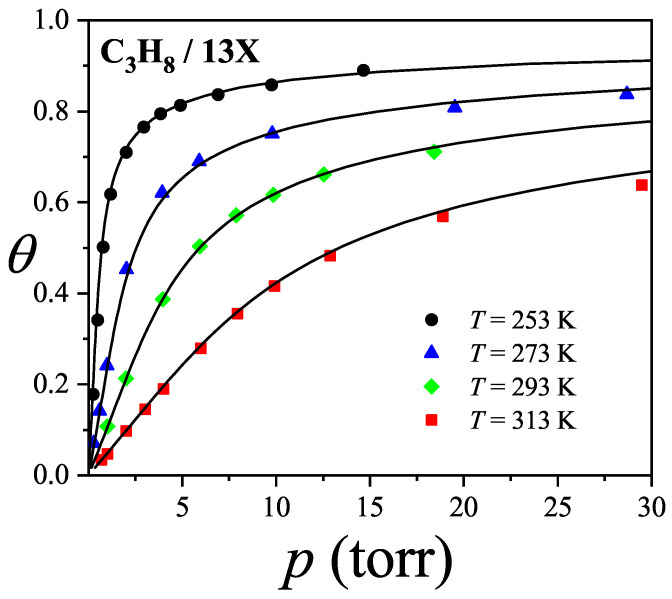
Adsorption isotherms of $C_{3}H_{8}$ on 13X zeolite modeled using the FSTA approach. Data points are taken from Ref. [143], while the curves illustrate the theoretical predictions based on Equation (264).

Figure 34 presents the adsorption isotherms of propane ($C_{3}H_{8}$) in 13X zeolite. Solid lines represent theoretical predictions using the FSTA model, while symbols show experimental data from Ref. [143]. Following conventional modeling, alkane chains are treated as “bead segments,” where each methyl group corresponds to one adsorption site. Accordingly, propane is modeled as a trimer with k=3. Given that the propane molecule (6.7) is relatively large compared to the cavity diameter (11.6), it likely adsorbs along a preferred orientation. Otherwise, accommodating 5–6 molecules per cavity would be unfeasible. We thus assign g=3 (with $k^{\prime }=k=3$ and m=1, mimicking a 1D configuration). The best fit to the experimental data over the full temperature and pressure range was obtained by simultaneously optimizing K(T), $v_{m}$, and *w* (see Table 2).

Consistent with the experimental findings, the resulting $v_{m}$ was slightly under 6 molecules per cavity. A value of $v_{m}$(=5.75) suggests that some molecules may partially span across the cavity windows. Regarding the lateral interaction parameter *w*, its ratio to the known liquid-phase interaction energy ϵ for propane is about w/ϵ≈2.5, indicating that each molecule interacts with 2.5 neighbors at full coverage on average. This supports the approximation of a quasi-one-dimensional system.

**Figure 35:**
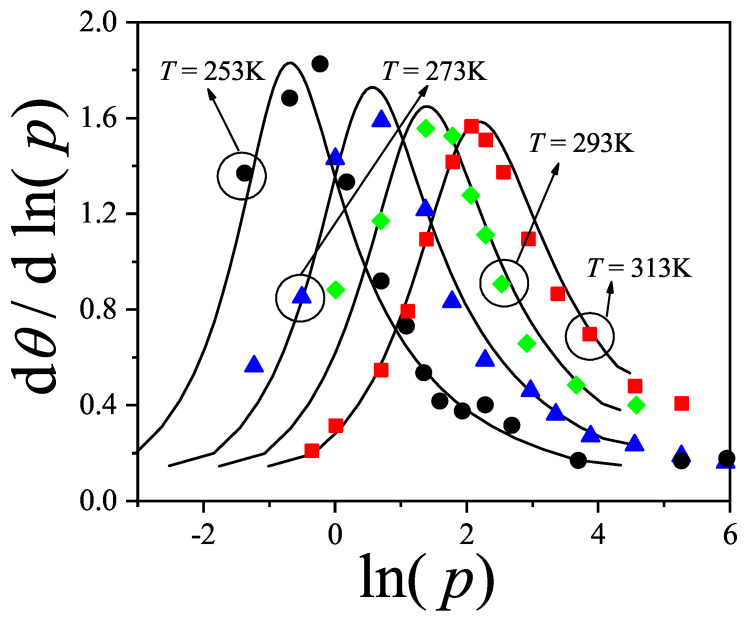
Plot of the derivative of the adsorbed quantity with respect to pressure (expressed as lnp), corresponding to the data shown in Figure 34.

Figure 35 also indicates strong agreement between theory and experimental data for a system with non-monotonic adsorption behavior. In this case, the derivative of the adsorbed volume with respect to pressure was fitted, again yielding excellent correlation.

To quantify model accuracy, we define the deviation *D* as the average relative discrepancy (in percent) between theoretical ($v_{theo}^{i}$) and experimental ($v_{exp}^{i}$) data:

(265) $$D=\sum_{i=1}^{n_{s}}\left\{100.\left|\frac{v_{theo}^{i}-v_{exp}^{i}}{v_{exp}^{i}}\right|\right\},$$

where *i* runs over the total set of data.

**Table 2:** Summary of fitting parameters corresponding to the data presented in Figure 34 and Figure 36. The quantities $H_{st}^{FSTA}$, $H_{st}^{exp}$, $w^{FSTA}$, and $w^{exp}$ are reported in kcal/mol (absolute values). The parameter $v_{m}$ is given in molecules per cavity for case (a), and in $cc_{STP}/$g of adsorbent for case (b), based on data from Refs. [144,145], respectively. Entries (c) and (d) correspond to experimental measurements from Refs. [143,144]; (e) gives simulation results from Ref. [146], whle (f) provides the $C_{3}H_{8}-C_{3}H_{8}$ interaction energy in the liquid state as reported in Ref. [143].

System	k	m	g	$v_{m}$	$H_{\mathrm{st}}^{\mathrm{FSTA}}$	$H_{\mathrm{st}}^{\exp}$	$w^{\mathrm{FSTA}}$	$w^{\exp}$	D (%)
$O_{2}/5A$	2	2	4	${12\;}^{\left(a\right)}–{130.9\;}^{\left(b\right)}$	3.10	${3.37\;}^{\left(c\right)}$	0.72	${0.54\;}^{\left(e\right)}$	5.60
$C_{3}H_{8}/13X$	3	1	3	5.75	6.94	${6.81\;}^{\left(d\right)}$	1.27	${0.50\;}^{\left(f\right)}$	2.08

For propane on 13X, *D* was found to be 2.08% (Table 2), which is within the bounds of experimental error, underscoring the robustness of the FSTA approach. In contrast, a previous model from Ref. [143] required eight parameters to describe similar systems. Here, the complexity of polyatomic adsorption is captured through a single, physically meaningful parameter *g*, reflecting the spatial configuration of the adsorbate.

**Figure 36:**
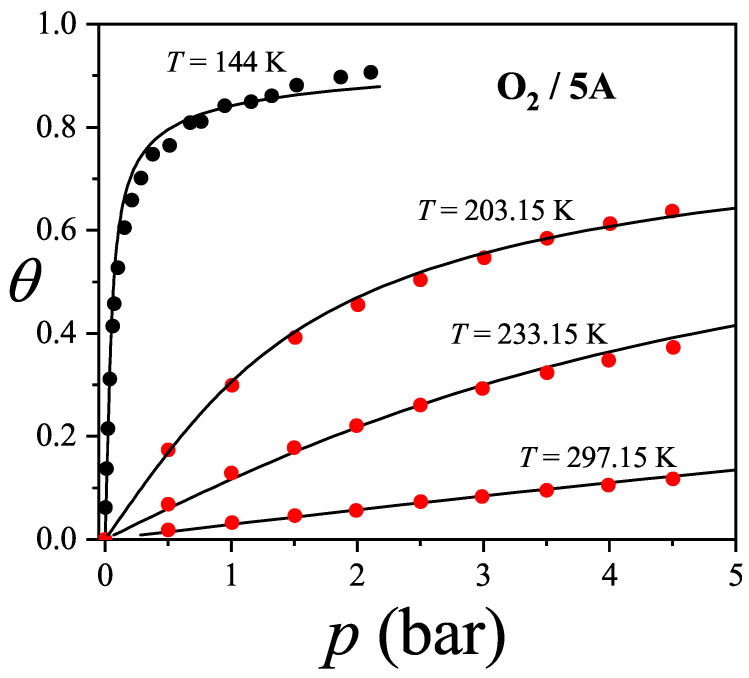
Adsorption isotherms for $O_{2}$ on 5A zeolite. Open and filled markers represent experimental data from Refs. [144,145], respectively. The solid curves depict the theoretical isotherm described by Equation (264).

We now examine the oxygen adsorption isotherms in 5A zeolite, as illustrated in Figure 36. Experimental data points are shown as symbols, while the curves are theoretical predictions based on Equation (264). Measurements were obtained from two independent sources: Miller et al. [144] and Danner et al. [145]. In the former (open symbols), adsorption quantities were reported in molecules per cavity; in the latter (filled symbols), adsorption was expressed in $cc_{STP}$ per gram of adsorbent.

The fitting procedure was carried out in two main steps: (i) using prior simulation findings [146], the exclusion parameter was fixed at g=4 (with $k=k^{\prime }=2$, and m=2). Based on this assumption, the analytical curves in Figure 36 were generated by simultaneously fitting K(T), $v_{m}$, and *w*, similar to the method used for Figure 34. The value of *w* obtained aligns closely with the simulation results reported in Ref. [146]. As for $v_{m}$, no direct comparison could be made with the value reported by Razmus et al. [147], as it was not specified in their study. However, $v_{m}$ was later confirmed through a second stage of analysis: (ii) keeping *g*, K(T), and *w* fixed from the first step, $v_{m}$ was adjusted to best fit the isotherm data from Danner et al. [145]. The resulting value was consistent with the monolayer volume they reported. The overall deviation between experimental data and the fitted curve was 5.60% (refer to Table 2). These findings suggest that $O_{2}$ likely adsorbs in a planar configuration with two orientation possibilities, forming a 2D layer along the cavity walls.

**Figure 37:**
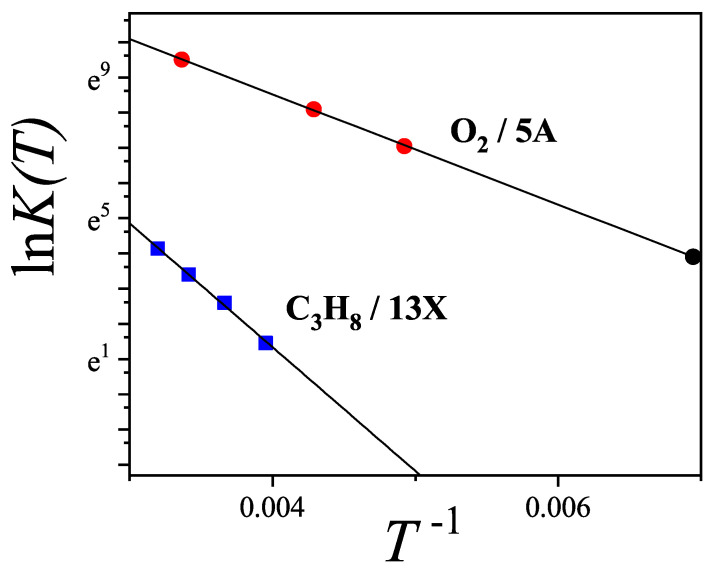
The variation of the equilibrium constant K(T) with temperature, as obtained through the fitting procedure. Square symbols, red circles, and the black circle correspond to fits based on data from Refs. [143,144,145], respectively. The quantity $H_{st}^{FSTA}$ listed in Table 2 corresponds to the absolute value of the slope of the fitted line.

The isosteric heat of adsorption, $H_{st}$, was calculated from the slope of lnK(T) versus 1/T, as plotted in Figure 37. The values listed in Table 2 are in good agreement with experimental results for propane on 13X [143] and for oxygen on 5A [147].

**Figure 38:**
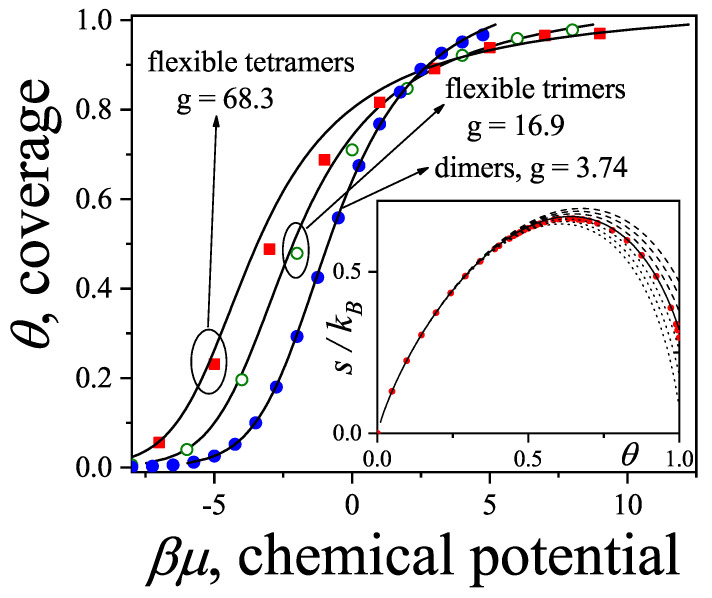
Comparison of Monte Carlo simulation results for the adsorption of dimers, trimers, and tetramers onto square lattices with the theoretical isotherms derived from the FSTA model [Equation (48)]. The optimal values of *g* used in each fit are noted in the figure. In the inset: configurational entropy plotted as a function of surface coverage for dimer adsorption onto square lattices. Simulation points are shown as symbols, while the solid lines correspond to theoretical predictions for various *g* values, as discussed in the text.

To demonstrate the broader applicability of the FSTA model beyond the experimental systems considered, Figure 38 presents fits (solid lines) to Monte Carlo adsorption isotherms (symbols) for dimers, flexible trimers, and flexible tetramers adsorbed flat onto square lattices. The solid lines represent the best fits obtained using the simplest version of the model, where *g* is assumed to be constant [Equation (48)], applied to the general isotherm of Equation (47). The optimized values of *g* are all consistent with the theoretical estimate g=mk. Notably, even at this basic approximation level, FSTA outperforms both Flory’s classical theory [6] for adsorbed chains and the multisite adsorption models presented in Refs. [20,33].

For the dimer case, the inset in Figure 38 compares the configurational entropy per lattice site, *s*, as a function of coverage between simulations and FSTA predictions. Here, entropy is defined as $S=-{\left(\frac{\partial F}{\partial T}\right)}_{N,M}$ and s=S/M. The best agreement occurs for g=3.74 (solid line). Dashed and dotted lines show the effects of increasing or decreasing *g* by 2%, 4%, and 6%, respectively. These results highlight the strong sensitivity of the entropy profile to small changes in *g*, demonstrating that the exclusion parameter is closely linked to the spatial arrangement of adsorbed particles. Tighter, more compact configurations yield lower *g* values; for example, g=6 (g=8) for straight trimers (tetramers), in contrast to g=18 (g=72) for flexible ones.

In summary, the analysis presented in this section demonstrates how the concept of statistical exclusion (g≥1) serves as a powerful tool to quantify the entropy associated with polyatomic adsorption, effectively capturing both the configuration and interaction of adsorbates. Furthermore, Equation (47) lays the groundwork for analyzing configurational changes as a function of coverage [through configurational spectroscopy $G^{\prime }\left(N\right)$] from thermodynamic data. Overall, FSTA offers a compact and insightful framework for interpreting adsorption phenomena on structured surfaces, ranging from simple gases to complex hydrocarbons.

### 7.5. Adsorption of Methane–Ethane Mixtures in Zeolites: Reversal Adsorption Phenomena

The adsorption of molecular mixtures presents a significantly greater challenge than that of pure components, both experimentally and theoretically. While the number of adsorbed molecules in a pure gas system can be accurately determined by measuring the weight gain of the adsorbent sample, studying mixtures requires additional experimental techniques to determine the composition of the adsorbed phase. This complexity is one of the primary reasons for the scarcity of experimental data on the adsorption of polyatomic mixtures.

Nonetheless, several simulation studies have investigated the behaviors of hydrocarbon mixtures [148,149,150,151,152,153,154,155]. A particularly striking phenomenon has been observed in methane–ethane mixtures [148,149]: at low pressures, the adsorbed phase is dominated by ethane but, as pressure increases, methane gradually displaces ethane. This inversion in adsorption preference has also been reported in mixtures of linear hydrocarbons adsorbed in silicalite [150,151,152,153,154,155], as well as in carbon nanotube bundles [156] and metal–organic frameworks [157]. In all these cases, selectivity shifts from favoring the larger molecule at low pressure to favoring the smaller one at higher pressure—a behavior known as Adsorption Preference Reversal (APR) [158].

From a theoretical perspective, the underlying mechanisms of APR are complex and not immediately apparent. In the context of single-site adsorption models, where each adsorbed molecule occupies only one lattice site, prior investigations conducted by Ayache et al. [158] and Dunne et al. [159] have demonstrated that species competition under conditions of lateral repulsion can result in one component displacing another from the surface. The study by Ayache et al. employed a mean-field approach, whereas Dunne et al. performed exact analyses.

However, when dealing with hydrocarbons adsorbed on solid substrates, it is more appropriate to consider multisite adsorption models. These frameworks better capture the influence of configurational entropy arising from molecular size, shape, and flexibility, on the thermodynamics of the adsorbed layer. Structural differences and variations in density between species introduce entropic contributions that are likely to govern a wide array of non-trivial phenomena, including complex adsorption behaviors and phase transitions.

Understanding how molecular size and structure affect the thermodynamic properties of the adsorbed layer is of fundamental importance in statistical physics. Furthermore, developing accurate theoretical descriptions of these systems has practical relevance in designing and optimizing separation processes in petrochemical applications. In this context, we present the first exact model for adsorption of molecular mixtures in zeolites that accounts for multisite occupancy. Using a rigorous statistical thermodynamic approach, we analyze mixtures of *s*-mers and *k*-mers (molecules occupying *s* and *k* lattice sites, respectively) on one-dimensional substrates. Our results reveal that the APR phenomenon emerges naturally from differences in molecular size (i.e., number of occupied sites) between the two species.

Following the line of Ref. [158], we start by calculating the parameter $A\equiv \exp\left[\beta \left(\Phi_{k}-\Phi_{s}\right)\right]$, which is obtained from the equilibrium equations [Equations (94) and (95) for γ=2 in Section 3.2]:

(266) $$\begin{array}{ccc} A & = & \frac{X_{s}}{X_{k}}\exp\left[\beta \left(\epsilon_{k}-\epsilon_{s}\right)-\beta \left(\mu_{k}^{0}-\mu_{s}^{0}\right)\right]\\ & = & \frac{k\theta_{s}}{s\theta_{k}}\frac{{\left(1-\theta_{s}-\theta_{k}\right)}^{k-s}}{{\left[1-\left(\frac{s-1}{s}\right)\theta_{s}-\left(\frac{k-1}{k}\right)\theta_{k}\right]}^{k-s}}. \end{array}$$

To explore the fundamental aspects of the phenomenon, we begin by analyzing a binary mixture of monomers (s=k=1), assuming equal molar fractions for both species in the gas phase ($X_{s}=X_{k}$). Under this condition, Equation (266) simplifies to

(267) $$A=\exp\left[\beta \left(\epsilon_{k}-\epsilon_{s}\right)-\beta \left(\mu_{k}^{0}-\mu_{s}^{0}\right).\right]=\frac{\theta_{s}}{\theta_{k}}.$$

**Figure 39:**
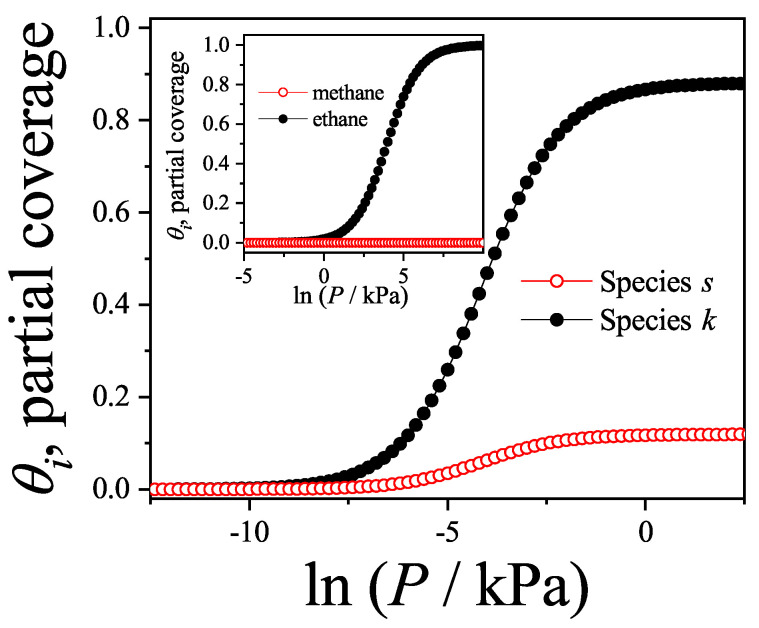
Partial adsorption isotherms for an equimolar monomer (s=1)–monomer (k=1) mixture on a one-dimensional lattice. Parameter values (figure): $\beta (\epsilon_{s}-\mu_{s}^{0})=-2$ and $\beta (\epsilon_{k}-\mu_{k}^{0})=-4$. Parameter values (inset): $\beta \epsilon_{s}=-14.77$, $\beta \epsilon_{k}=-22.81$, $\beta \mu_{s}^{0}=-25.83$, and $\beta \mu_{k}^{0}=-26.77$.

This expression implies that the ratio $\theta_{s}/\theta_{k}$ remains constant, meaning the respective adsorption isotherms for species *s* and *k* do not cross and, thus, no APR takes place. This behavior is illustrated in Figure 39, where the system is examined using the parameters $\beta (\epsilon_{s}-\mu_{s}^{0})=-2$ and $\beta (\epsilon_{k}-\mu_{k}^{0})=-4$. The inset provides a complementary example, using the adsorption energy values reported by Ayache et al. [158]: $\beta \epsilon_{s}=-14.77$ (equivalent to $\epsilon_{s}=-5.1\times 10^{-20}$ J at T=250 K) and $\beta \epsilon_{k}=-21.81$ (i.e., $\epsilon_{k}=-7.875\times 10^{-20}$ J at the same temperature). The standard chemical potentials, $\beta \mu_{s}^{0}$ and $\beta \mu_{k}^{0}$, were computed using Equation (93) by assigning molecular masses corresponding to methane and ethane; that is, $m_{s}=16.04$ u and $m_{k}=30.07$ u (with 1 u = $1.660531\times 10^{-27}$ kg) [160]. This yields values of $\beta \mu_{k}^{0}=-26.77$ and $\beta \mu_{s}^{0}=-25.83$.

The results presented in Figure 39 align with earlier studies on monomer–monomer mixtures [158], where the occurrence of APR required the introduction of a complex set of lateral interactions.

We now turn to the more general case of an equimolar mixture, composed of *s*-mers and *k*-mers. As indicated by Equation (266), when both species have the same size (s=k), the individual adsorption isotherms do not intersect and the resulting curves resemble those observed in Figure 39.

However, when s≠k, the system exhibits a broader range of behaviors that depend on both the value of the interaction parameter *A* and the size relationship between the two species. Without loss of generality, we consider the case where s<k. In evaluating the role of *A*, we rely on two key physical assumptions regarding the adsorption of linear alkanes: the heats of adsorption (1) are attractive and (2) increase (in absolute value) linearly with the length of the hydrocarbon chain. It is important to emphasize that these are not merely theoretical assumptions: they reflect well-established experimental findings on the energetics of alkane adsorption in zeolites, supported by extensive literature evidence [140,161,162,163].

Taking these assumptions into account, the exponential term in Equation (266) becomes negative, implying that the parameter *A* lies in the range between 0 and 1. Under such conditions, there exists a specific coverage value $\theta^{*}$ ($0<\theta^{*}<1$) at which the adsorption isotherms for species *s* and *k* intersect ($\theta_{s}=\theta_{k}=\theta^{*}$), signaling the occurrence of the APR effect. This intersection point can be readily determined from Equation (266) through straightforward algebraic manipulation:

(268) $$\theta^{*}=\frac{1-{\left(\frac{sA}{k}\right)}^{1/(k-s)}}{2-\left(\frac{k-1}{k}+\frac{s-1}{s}\right){\left(\frac{sA}{k}\right)}^{1/(k-s)}}.$$

The coordinate $\left(\mu^{*},\theta^{*}\right)$ defines the boundary between two distinct adsorption regimes. When $\mu <\mu^{*}$, species *k* dominates the adsorbed phase ($\theta_{k}>\theta_{s}$) whereas, for $\mu >\mu^{*}$, species *s* becomes predominant ($\theta_{s}>\theta_{k}$). Therefore, the emergence of the crossover at $\theta^{*}$ is intrinsically linked to the substitution of species *k* by species *s*, which characterizes the APR phenomenon.

**Figure 40:**
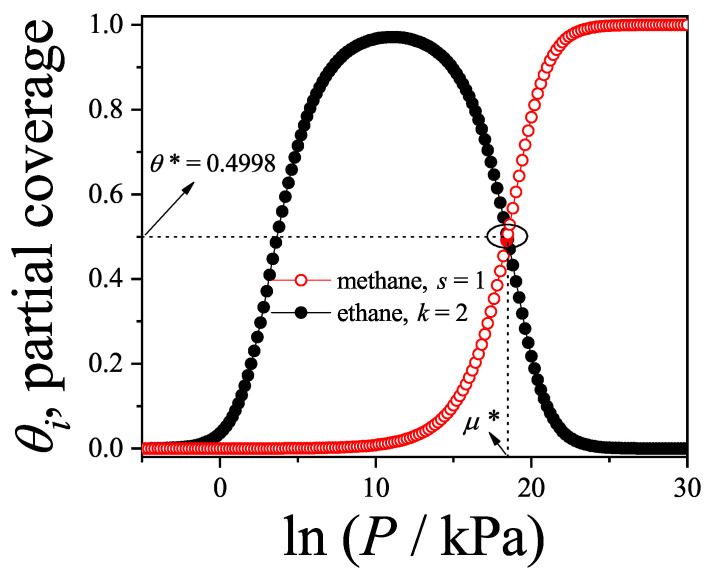
Partial adsorption isotherms for an equimolar methane (s=1)–ethane (k=2) mixture on an one-dimensional lattice. Parameter values: $\beta \epsilon_{s}=-14.77$, $\beta \epsilon_{k}=-22.81$, $\beta \mu_{s}^{0}=-25.83$, and $\beta \mu_{k}^{0}=-26.77$.

This behavior is clearly illustrated in Figure 40, which examines a monomer–dimer mixture (s=1 and k=2) with equal concentrations of each species in the gas phase. The parameter values for $\beta \epsilon_{s}$, $\beta \epsilon_{k}$, $\beta \mu_{s}^{0}$, and $\beta \mu_{k}^{0}$ are the same as those given in the inset of Figure 39. This monomer–dimer case is particularly insightful because: (*i*) it represents the simplest model of binary adsorption involving species of different sizes; (ii) it captures the essential characteristics of multisite occupancy adsorption; and (iii) it serves as a useful model for interpreting experimental adsorption of methane–ethane mixtures in silicalite. Following a widely used approach, we adopt the bead-segment model, where each methyl group is represented by a single unit (or “bead”) equivalent in size to one adsorption site. Within this framework, methane and ethane are modeled as s=1 and k=2, respectively.

Given that ethane molecules possess a stronger affinity for the surface (higher adsorption energy), they adsorb preferentially at low pressures. However, as pressure increases, adsorption of the smaller methane molecules becomes more favorable, eventually displacing the previously adsorbed ethane. As a result, the partial isotherms intersect at a critical coverage $\theta^{*}$, indicating the occurrence of the APR phenomenon. In this specific case, $\theta^{*}=0.4998$.

The exact results presented here offer a significant contribution to understanding the APR mechanism, demonstrating that it naturally arises from size disparity (or, more precisely, the number of occupied sites) between different adsorbed species. It is worth highlighting that, in earlier theoretical treatments—such as the monomer–monomer model presented in Ref. [158]—reproducing APR required a highly parameterized model involving six variables (adsorption energies for methane and ethane, pairwise interaction energies among methane–methane, methane–ethane, and ethane–ethane, plus temperature). This complexity led to poorly constrained parameters and only qualitative conclusions. In fact, extremely small changes in parameter values (much smaller than typical experimental uncertainties) produced vastly different predictions. For example, a mere 5% change in ethane’s adsorption energy could determine whether APR appears or not in the model of Ref. [158].

Supporting this point, Equation (268) demonstrates that APR can emerge solely due to size asymmetry (s≠k), even in the absence of any energetic contributions or lateral interactions ($\beta \epsilon_{s}=\beta \epsilon_{k}=\beta \mu_{s}^{0}=\beta \mu_{k}^{0}=0$, and A=1). This reveals that introducing complex lateral interactions in previous models may be seen as an artificial or effective way to account for purely geometric or steric effects through energetic means.

In summary, this analysis leads to two key conclusions: (1) the entropic effects arising from the non-spherical nature of polyatomic adsorbates play a crucial role in surface phenomena, and cannot be neglected in comparison with monoatomic adsorption; and (2) failing to incorporate the polyatomic nature of adsorbates into thermodynamic models can lead to a serious misinterpretation of experimental data, particularly regarding phenomena such as APR.

### 7.6. Alkanes Adsorbed in Carbon Nanotube Bundles: Surface Area Characterization

The multilayer adsorption theory for polyatomic molecules, as discussed in Section 2, is utilized here to evaluate the surface area of a HiPco single-walled carbon nanotube (SWCNT) sample. This analysis was based on adsorption isotherms obtained using a series of four alkanes; namely, methane through butane.

The SWCNT sample analyzed in this study was procured from Carbon Nanotechnology Inc. (CNI). No post-processing was applied beyond evacuating the sample under vacuum (better than $1\times 10^{-6}$ Torr) for 72 h before each measurement cycle. Although the purification method used by the manufacturer may have opened some nanotube ends, these openings were effectively sealed by chemical groups introduced during that same process [164]. To unblock these caps, heating to at least 650 K under vacuum is necessary [165,166]—a procedure not undertaken in this work. As a result, internal adsorption was excluded and the sample effectively behaved as a non-porous substrate. Adsorption primarily occurred on the external surfaces of nanotube bundles and, to a lesser extent, on large-diameter, defect-related interstitial sites [167,168].

The isotherms were recorded using a custom-built volumetric adsorption setup [169]. Low temperatures were achieved using a helium closed-cycle refrigeration system, and temperature regulation was maintained with two controllers. Pressure measurements were conducted using three capacitance manometers with upper limits of 1, 10, and 1000 Torr, respectively. These gauges were housed in the room-temperature gas-handling system. Data acquisition and gas dosing were managed by a LabView-based program developed in-house. All gases employed were of ultra-high purity, and were sourced from Matheson Gas.

To determine the surface area, we calculated the product of the molecular area of the adsorbate [170,171,172] and the monolayer capacity of the substrate. The monolayer capacity was derived from experimental adsorption isotherms using two approaches: the BET method [71,78] and the point B method [173,174]. The specific surface area was then calculated by dividing the total surface area by the sample’s mass.

**Figure 41:**
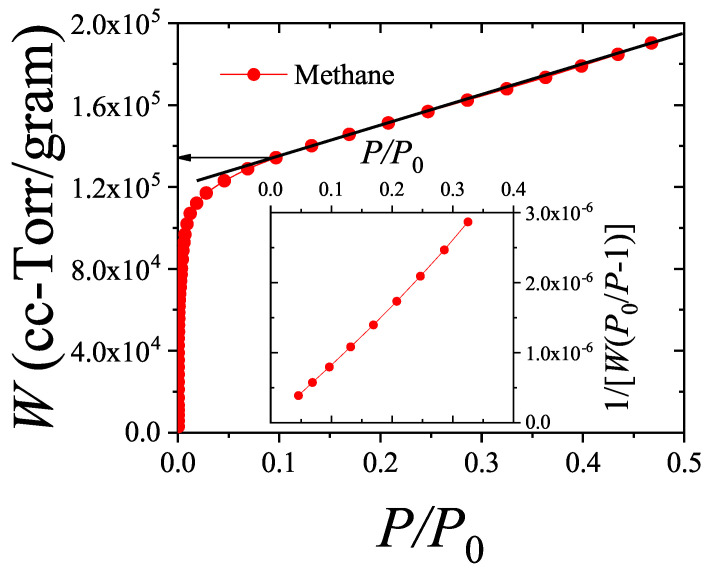
The adsorption isotherm for methane on single-walled carbon nanotubes, with the coverage (expressed in cm^3^ Torr/g) plotted on the *Y*-axis against the relative pressure on the *X*-axis. Initially, there is a steep rise in coverage at low pressures, followed by a region where the coverage increases linearly with pressure. The point at which the isotherm first diverges from this linear behavior (indicated by the arrow aligned with the *X*-axis in the figure) is identified as point B. This point is interpreted as corresponding to the completion of the monolayer.

The point B method involves plotting the adsorption isotherm as adsorbed amount versus pressure on a linear scale. Initially, coverage increases steeply with pressure, eventually reaching a pronounced inflection point. Beyond this point, coverage increases more gradually and linearly, until multilayer adsorption begins. Point B is identified as the lowest pressure at which linear extrapolation aligns with this intermediate linear region between the completion of the first layer and the start of the second [173,174]. The coverage at point B corresponds to monolayer completion, as illustrated in Figure 41. Generally, this method yields a slightly higher monolayer capacity than the BET equation.

**Figure 42:**
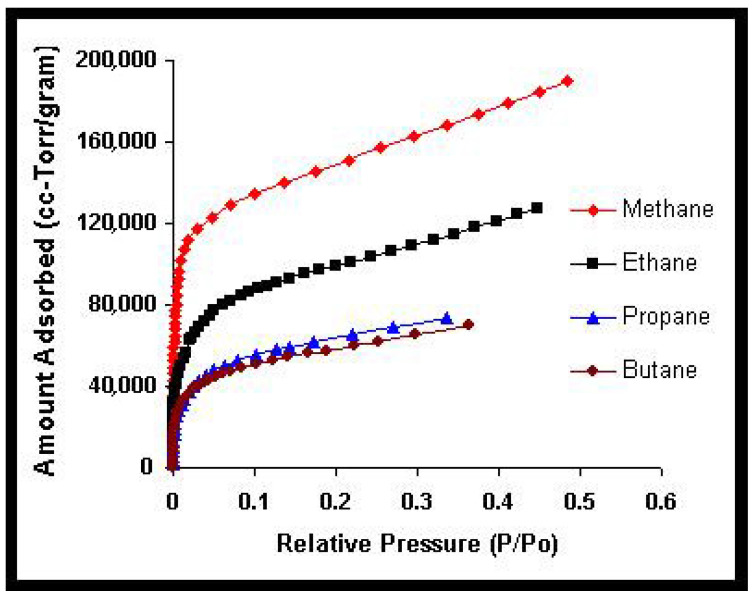
Adsorption isotherms for methane (77.3 K), ethane (165 K), propane (190 K), and butane (220 K) adsorption on single-walled carbon nanotubes. The coverage in cm^3^ Torr/g (*Y* axis) is presented as a function of relative pressure (*X* axis).

Figure 42 shows the isotherms obtained for the HiPco nanotube sample using methane, ethane, propane, and butane. Different temperatures were used for each alkane, due to their differing adsorption energies. Methane requires low temperatures whereas other alkanes exhibit vapor pressures too low for reliable measurement in our setup [175,176]. Conversely, the higher temperatures that are suitable for measuring butane adsorption produce pressures for methane that are too high to resolve the monolayer region effectively [176].

To ensure comparability across different gases, isotherm temperatures were scaled by each adsorbate’s bulk critical temperature ($T/T_{c3D}$), aligning the scaled temperatures across the data sets.

Table 3 compiles the molecular areas of the alkanes used, which were obtained from published neutron scattering results for alkane films adsorbed on graphite [170,171,172].

**Figure 43:**
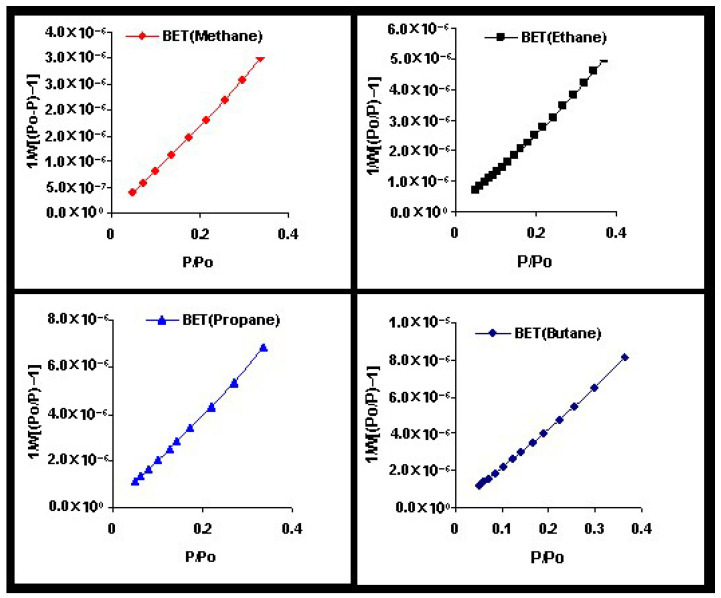
BET analysis for the adsorption isotherms of methane, ethane, propane, and butane on single-walled carbon nanotubes shown in Figure 42.

Figure 43 presents plots of the linearized BET equation for the four different adsorbates used. In particular, the linearized BET equation is

(269) $$\left(P/P_{0}\right)/\left[(1-P/P_{0})\right]=1/Cn_{m}+\left[\left(C-1\right)P/P_{0}\right]/Cn_{m}.$$

In this context, *P* refers to the pressure at a specific point on the adsorption isotherm, $P_{0}$ is the saturated vapor pressure of the adsorbate at the isotherm temperature, *n* denotes the number of molecules adsorbed at pressure *P*, $n_{m}$ represents the monolayer capacity of the substrate, and *C* is a constant that reflects the interaction strength between the adsorbate and the substrate.

The BET equation is typically applied over a range of relative pressures from approximately $P/P_{0}\approx$ 0.05 to $P/P_{0}\approx$ 0.3 [71,78,174]. This pressure range was used to plot the isotherm data in Figure 42, which yielded the results shown in Figure 43.

**Figure 44:**
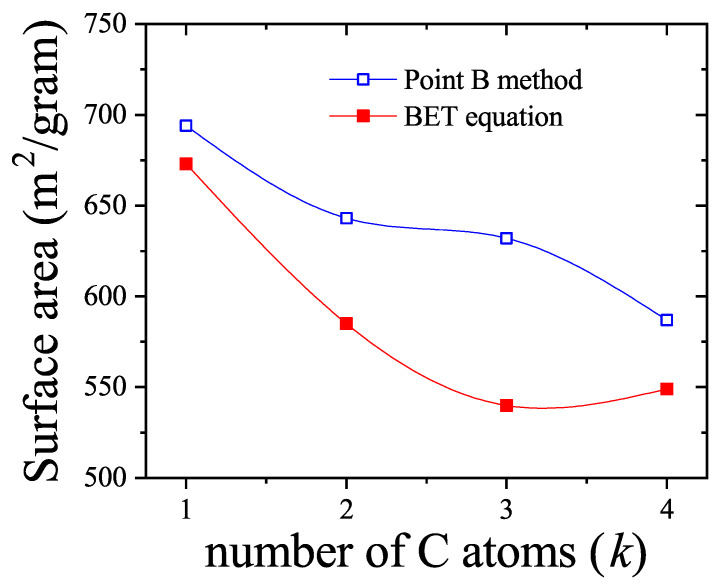
Specific surface area of single-walled carbon nanotubes computed using the BET and the point B methods. The specific surface area in m^2^/g (*Y* axis) is presented as a function of number of carbon atoms in the adsorbate (*X* axis).

Figure 44 displays the specific surface area values for the HiPco nanotube sample, as determined using both the point B method and the BET equation for different adsorbates. Notably, both methods revealed the same trend: as the molecular length of the adsorbate increases, the specific surface area calculated for the same sample decreases. Within the range of molecule sizes examined, this decrease is approximately 20% for each method.

In an earlier study, we measured the specific surface area of an SWCNT sample using several spherical adsorbates (neon, argon, methane, and xenon) and found that the value was essentially independent of the size of the adsorbate [177]. In the analysis, the point B method was employed to determine the monolayer capacity. These results differ markedly from the findings reported here for the linear alkane series (methane through butane). The key distinction lies in molecular geometry: unlike the spherical adsorbates used previously, the alkanes are linear, which enhances their tendency for multisite occupancy during adsorption.

We now proceed to analyze the experimental data using the theoretical framework for multilayer adsorption of polyatomic molecules, as developed in Section 2 and Section 3.3. The model outlined in Section 2 will be referred to hereafter as the “modified BET model” (or simply the MBET model). As previously mentioned, in the one-dimensional (1-D) case, the MBET equation reduces to the standard BET equation when considering monomer adsorption (k=1). The explicit expression derived for the adsorption of dimers (k=2) in 1-D within the MBET framework is given by:

(270) $$n=\frac{n_{m}}{\left(1-P/P_{0}\right)}\left\{1-{\left[\frac{\left(1-P/P_{0}\right)}{1+\left(4c-1\right)P/P_{0}}\right]}^{1/2}\right\}.$$

The variables appearing in Equation (270) carry the same meaning as those defined in Equation (269). Equation (270) is applicable for determining the monolayer capacity and specific surface area in the case of ethane adsorption, where ethane molecules can be effectively treated as dimers. Unlike the standard BET equation, the MBET expression for dimers is non-linear. When applied to ethane, the MBET model yields a larger surface area than the value obtained using the BET equation on the same dataset. Notably, the MBET model for dimers involves only two adjustable parameters—the same number as in the BET formulation.

**Figure 45:**
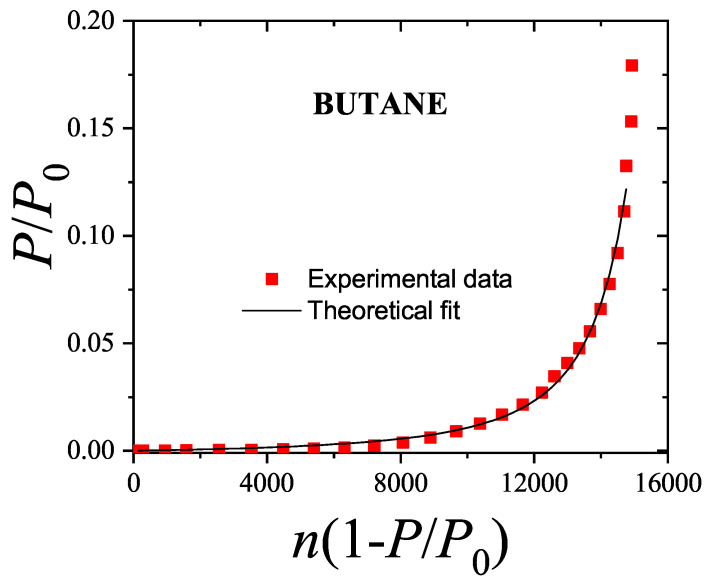
Fit of the low-pressure region of the butane isotherm data to Equation (271) in the text (obtained using the MBET approach). The value of the monolayer capacity is extracted from this fit.

As previously mentioned, for systems with k>1 in two dimensions (2-D), no exact analytical expressions exist for adsorption isotherms; i.e., for the fractional coverage *n* as a function of the relative pressure $P/P_{0}$. To address this challenge, we adopted two different strategies to extract the monolayer capacity from experimental data:

(1) In this first approach (procedure A), we used the one-dimensional MBET equations for all four adsorbates to account for the linear geometry of the molecules. For methane, we employed the standard BET equation, which corresponds to the MBET expression for monomers. For ethane, we applied the exact 1-D MBET formula for dimers [Equation (270)]. For propane and butane, we utilized the same dimer equation but adjusted its parameters to fit the experimental data in the low-pressure, low-coverage regime—specifically, the same region typically used in BET analysis.
  Using Procedure A, the calculated specific surface areas for ethane, propane, and butane were consistently higher than those obtained using the BET method. In addition to producing improved results, this method remains relatively straightforward to implement.
  However, we also observed a consistent trend: as the length of the alkane chain increases, the derived specific surface area decreases. This behavior is illustrated in Figure 6, which includes data obtained from both this and other approaches.
  Although not perfect, the results from Procedure A represent a clear enhancement over the standard BET approach.
(2) The second strategy (procedure B) involved fitting the isotherm data for all four adsorbates to the approximate MBET expression developed for the two-dimensional case [Equation (137) in Section 3.3]:
  (271) $$\frac{P}{P_{0}}=\frac{\frac{n(1-P/P_{0})}{n_{m}}{\left\{1-\frac{\left(k-1\right)}{k}\left[\frac{n(1-P/P_{0})}{n_{m}}\right]\right\}}^{k-1}}{kC_{ef}{\left[1-\frac{n(1-P/P_{0})}{n_{m}}\right]}^{k}+\frac{n(1-P/P_{0})}{n_{m}}{\left\{1-\frac{\left(k-1\right)}{k}\left[\frac{n(1-P/P_{0})}{n_{m}}\right]\right\}}^{k-1}}.$$
  In this formulation, $C_{ef}$ is a constant that reflects both the interaction strength between the adsorbate and the substrate, as well as the connectivity of the adsorption lattice. The parameter *k* denotes the number of units in the *k*-mer molecule.
  The experimental adsorption isotherms were fitted to this model using the appropriate *k* value for each adsorbate—1 for methane, 2 for ethane, 3 for propane, and 4 for butane—within the same low-pressure range typically employed for BET analysis. Figure 45 shows the fit obtained for butane, which demonstrates excellent agreement with the data; similarly accurate fits were achieved for the other three alkanes. The fits yielded values for the monolayer capacity, $n_{m}$, which were subsequently used to compute the specific surface area of the sample.

**Figure 46:**
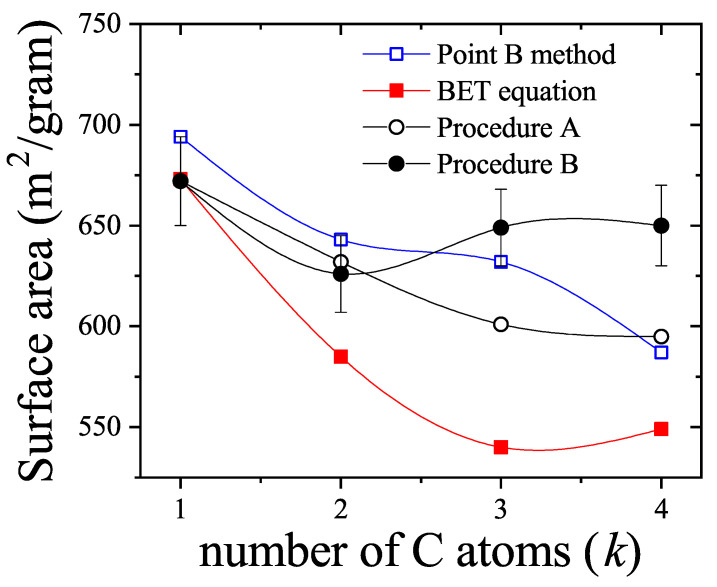
Specific surface area values for single-walled carbon nanotubes calculated using four different methods: BET, point B, procedure A, and procedure B. The results, expressed in m^2^/g (*Y*-axis), are plotted as a function of the number of carbon atoms in the adsorbate molecules (*X*-axis). To maintain clarity in the figure, error bars are shown only for the values obtained using procedure B.

Figure 46 presents the key findings of this study, including the specific surface area values of the substrate calculated using the four methods discussed: the point B method, the BET equation, the 1-D MBET model (Procedure A), and the 2-D MBET model (Procedure B). These values are plotted against the number of carbon atoms in the alkane adsorbates used in the isotherm measurements. Among the four approaches, only the 2-D MBET model (Procedure B) produced surface area values that remained nearly constant across the range of adsorbates. In contrast, the other three methods showed a decreasing trend in surface area with increasing alkane chain length.

This result has practical significance, as it offers a consistent approach for determining surface area using linear adsorbates. Specifically, the 2-D MBET model allows for surface area measurements with linear molecules that closely mirror the consistency typically achieved when using spherical adsorbates with the BET or point B methods.

From a more fundamental standpoint, our findings underscore the significance of the additional entropy contribution that emerges in monolayer films of linear molecules due to multisite occupancy.

In summary, we presented adsorption isotherm measurements for a series of four alkanes (methane, ethane, propane, and butane) in this section. These data were then used to estimate the specific surface area of a single substrate using four different methodologies: the traditional BET equation, the point B method, and two versions (1-D and 2-D) of a more recent model for the adsorption of linear molecules, known as the MBET method. Our key conclusion is that the 2-D MBET approach (procedure B) yields consistent surface area values across different linear adsorbates, in contrast to the other methods evaluated (point B, BET, or the 1-D MBET model), which did not exhibit such consistency.

This result holds both practical and theoretical significance. Practically, it offers a reliable method for determining the specific surface area using longer linear molecules, producing values that align well with those obtained from spherical adsorbates using the BET equation. This, in turn, enhances the comparability of results obtained for the same substrate across different types of adsorbates.

### 7.7. Crystal Growth from Aqueous Solution in the Presence of Structured Impurities

The process of crystal growth plays a vital role in both biological systems and industrial technologies. It begins with the formation of stable nuclei, followed by the gradual addition of atoms or molecules onto the crystal’s surface. These growth units—comprising atoms or molecules identical to those in the crystal—migrate from the bulk solution and attach to specific surface sites, eventually leading to the formation of macroscopic structures with well-defined surfaces.

It is well established that the adsorption of foreign atomic or molecular species—known as impurities—can significantly influence the rate of crystal growth [178,179,180]. This phenomenon has been widely utilized to control crystal morphology and enhance the properties of crystalline materials, powders, and granulated substances. Understanding the mechanisms through which impurities exert their effects is therefore of considerable importance.

Numerous studies have explored the influences of impurities on both the growth rate and morphology of single crystals [181,182,183,184,185,186]. Theoretical models addressing these influences typically assume that impurities (ions, atoms, or molecules) adsorb at specific surface features such as kinks, steps, and terraces during crystal growth. Early theoretical efforts in this area were made by Bliznakov [187,188,189,190], who focused on impurity adsorption at step edges, and by Cabrera and Vermilyea [191], who considered adsorption on flat surface terraces. Experimental data on crystal growth rates have supported the predictions made using these models [185].

In a key contribution, Davey and Mullin (DM) [192] developed a theoretical framework to describe how impurities affect the growth rate of crystals from aqueous solutions. Their model posits that the adsorption of impurities is limited to a thin layer adjacent to the crystal surface, and assumes no lateral or vertical interaction between adsorbed impurity molecules. In this context, the velocity of step movement in the presence of impurities, *V*, is given by:

(272) $$\frac{V}{V_{0}}=1-\theta_{eq},$$

where $V_{0}$ is the step velocity in the absence of impurities and $\theta_{eq}$ denotes the fraction of surface sites occupied by impurities. Consequently, $1-\theta_{eq}$ reflects the proportion of sites available for the adsorption of pure growth units. As $\theta_{eq}$ approaches 1, the surface becomes fully covered with impurity species, and crystal growth effectively halts.

The ability of an impurity to inhibit crystal growth is also believed to depend on factors such as its size, shape, and orientation—a phenomenon referred to as stereochemical effect. To incorporate these aspects, Kubota and Mullin (KM) [193] proposed an improved kinetic model. Their approach considers the adsorption of impurities along step edges and introduces a parameter, α, which represents the efficiency of impurity blockage. The step velocity in this model is given by:

(273) $$\frac{V}{V_{0}}=1-\alpha \theta_{eq}\;\;\;\;\;\;\;\;\;\left(\alpha \geq 0\right).$$

This equation indicates that the overall impact of impurities on growth rate is governed by two factors: the surface coverage by impurities ($\theta_{eq}$) and the effectiveness of each adsorbed molecule (α) in hindering growth.

The relationship between impurity concentration ($C_{I}$) and surface coverage ($\theta_{eq}$) is typically described using adsorption isotherms. The Langmuir isotherm [194] (or its extended versions [195]) is often used due to its mathematical simplicity. Then,

(274) $$\theta_{eq}=\frac{KC_{I}}{1+KC_{I}},$$

where K is the Langmuir constant. Introducing Equation (274) into Equations (272) and (273), the relative step velocity can be written in terms of the impurity concentration:

(275) $$\frac{V}{V_{0}}=1-\left(\frac{KC_{I}}{1+KC_{I}}\right)\;\;\;\;\;\;\left(\mathrm{DM}\;\mathrm{model}\right),$$

and

(276) $$\frac{V}{V_{0}}=1-\alpha \left(\frac{KC_{I}}{1+KC_{I}}\right)\;\;\;\;\;\;\left(\mathrm{KM}\;\mathrm{model}\right).$$

The formulations provided in Equations (275) and (276) are based on the premise that each impurity molecule occupies a single adsorption site, without considering the spatial distribution or arrangement of these sites on the surface. Consequently, these models are unable to distinguish between impurities of different structural complexity or between surfaces with varying lattice topologies. This drawback has prompted the development of more refined theoretical approaches which are capable of capturing the equilibrium adsorption behavior of polyatomic adsorbates with distinct geometries.

In this framework, we introduce a new extension of the DM model [192] for predicting crystal growth rates, which explicitly incorporates both the molecular dimensions and shapes of the impurities, along with the geometric characteristics of the adsorption surface. Similar to the KM model, our theoretical description is grounded in the step-pinning mechanism originally proposed by Cabrera and Vermilyea [191] to explain the retardation of step propagation. Building upon the model presented in Section 3.1.1, we utilize the EA adsorption isotherm [Equation (43)] to quantify the equilibrium surface’s coverage $\theta_{eq}$ by impurities.

Prior to comparing predictions with experimental data—and in order to adequately describe adsorption from liquid-phase solutions—it is helpful to reformulate the theoretical isotherm from Section 3.1.1 into a more suitable form. Invoking the equilibrium condition $\mu =\mu_{sol}$, Equation (43) can be rewritten as follows:

(277) $$kK(\gamma ,k)\exp\left[\beta \left(\mu_{sol}-k\epsilon_{0}\right)\right]=\frac{\theta {\left[1-\frac{\left(k-1\right)}{k}\theta \right]}^{k-1}}{{\left(1-\theta \right)}^{k}},$$

where $\mu_{sol}$ is the chemical potential of an ideal solution. Hence,

(278) $$\beta \mu_{sol}=\beta \mu_{0}+\ln\rho_{I},$$

where $\mu_{0}$ is the standard chemical potential, which is defined as

(279) $$\begin{array}{cc} \mu_{0} & =-k_{B}T\ln\left\{{\left(\frac{2\pi mk_{B}T}{h^{2}}\right)}^{3/2}k_{B}T\right\}, \end{array}$$

where *h* is the Planck constant ($h=6.6260\times 10^{-34}$ J·s) and *m* is the mass of the *k*-mer. Taking into account that the mass of a *k*-mer is directly proportional to the mass of the monomer unit—that is, $m=km_{0}$, with as $m_{0}$ the mass of the monomer unit—Equation (278) can be rewritten as:

(280) $$\begin{array}{c} \beta \mu_{sol}=-\frac{3}{2}\ln k-\ln\left\{{\left(\frac{2\pi m_{0}k_{B}T}{h^{2}}\right)}^{3/2}k_{B}T\right\}+\ln\rho_{I}, \end{array}$$

and

(281) $$\begin{array}{c} \beta \mu_{sol}=-\frac{3}{2}\ln k+\ln C_{I}, \end{array}$$

where $C_{I}$ is the impurity concentration, $C_{I}=\rho_{I}/\rho_{I}^{*}$, and $\rho_{I}^{*}=\left\{{\left(\frac{2\pi m_{0}k_{B}T}{h^{2}}\right)}^{3/2}k_{B}T\right\}$. This expression relates the chemical potential in solution with the size(s) of the impurity and its concentration in the bulk. Introducing Equation (281) into Equation (277), the following expression is obtained:

(282) $$K(\gamma ,k)K_{k}C_{I}=\frac{\theta {\left[1-\frac{\left(k-1\right)}{k}\theta \right]}^{k-1}}{{k\left(1-\theta \right)}^{k}},$$

where $K_{k}=\exp\left(-\frac{3}{2}\ln k-k\epsilon_{0}\right)$.

Equation (34) is valid for k≥2 (K(γ,k)=1 for k=1).

To evaluate the relative step velocity $V/V_{0}$ for impurity molecules of varying geometry and size, we substitute the equilibrium surface coverage $\theta_{eq}$ with θ in Equation (272). This requires first expressing Equation (282) in terms of the impurity concentration $C_{I}$; that is, rewriting it in the form $\theta =\theta (C_{I})$. This transformation has been carried out for linear-shaped impurities, where the function K(γ,k) takes the value of 1 for k=1, and γ/2 for k≥2. Here, *k* denotes the length of the impurity molecule, ranging from 1 to 4, and adsorption is considered to occur on a square lattice with coordination number γ=4. (For impurity sizes k≥5, the relationship $\theta (C_{I})$ cannot be expressed analytically, but numerical evaluations can be easily performed using symbolic computation tools.) The resulting expressions for θ as a function of $C_{I}$ under these conditions are given below:

(283) $$\begin{array}{ccc} \frac{V}{V_{0}} & = & 1-\theta_{eq}\\ & = & 1-\theta_{1}\\ & = & 1-\frac{K_{1}C_{I}}{1+K_{1}C_{I}}\;\;\;\;\;\;\left(k=1\right), \end{array}$$

(284) $$\begin{array}{ccc} \frac{V}{V_{0}} & = & 1-\theta_{eq}\\ & = & 1-\theta_{2}\\ & = & \frac{1}{\sqrt{1+8K_{2}C_{I}}}\;\;\;\;\;\;\left(k=2\right), \end{array}$$

(285) $$\begin{array}{ccc} \frac{V}{V_{0}} & = & 1-\theta_{eq}\\ & = & 1-\theta_{3}\\ & = & \frac{1}{2}\left(\frac{\sqrt[{3}]{54K_{3}C_{I}A+6\sqrt{3}\sqrt{K_{3}C_{I}{\left(A-2\right)}^{3}}+8}}{A-2}\right)\\ & + & \frac{1}{2}\left(\frac{2^{2/3}}{\sqrt[{3}]{27K_{3}C_{I}A+3\sqrt{3}\sqrt{K_{3}C_{I}{\left(A-2\right)}^{3}}+4}}-2\right)\;\;\;\left(k=3\right), \end{array}$$

with $A=(27K_{3}C_{I}+4)$. In addition,

(286) $$\begin{array}{ccc} \frac{V}{V_{0}} & = & 1-\theta_{eq}\\ & = & 1-\theta_{4}\\ & = & \sqrt{\frac{E}{3D}}+\frac{1}{2}\sqrt{\frac{4}{D}\left(F+2\sqrt{\frac{3}{DE}}\right)}\;\;\;\;\;\;\left(k=4\right), \end{array}$$

where

(287) $$E=\left(-4\sqrt[{3}]{3\sqrt{3}\sqrt{K_{4}^{2}C_{I}^{2}D}-27K_{4}C_{I}}+\frac{32\;3^{2/3}K_{4}C_{I}}{\sqrt[{3}]{\sqrt{3}\sqrt{K_{4}^{2}C_{I}^{2}D}-9K_{4}C_{I}}}+9\right),$$

(288) $$F=\frac{4\sqrt[{3}]{\sqrt{3}\sqrt{K_{4}^{2}C_{I}^{2}D}-9K_{4}C_{I}}}{3^{2/3}}-\frac{32K_{4}C_{I}}{\sqrt[{3}]{\sqrt{3}\sqrt{K_{4}^{2}C_{I}^{2}D}-27K_{4}C_{I}}}+6,$$

where $D=512K_{4}C_{I}+27$.

The framework outlined in Equations (283)–(286) represents a refinement of the earlier KM model [193], which incorporated the effects of impurity geometry solely through a single proportionality factor known as the effectiveness parameter α (α≥0). Unlike the KM formulation, the present model introduces expressions for $V/V_{0}$ that: (1) explicitly account for the entropic impact of non-spherical impurity shapes, and (2) enable the analysis of how the spatial configuration of adsorbed molecules affects crystal growth. These effects are represented through physically meaningful parameters that can be linked directly to thermodynamic observables and which reflect the geometric arrangement of the impurity species on the surface.

In the following, we compare our theoretical predictions with results from MC simulations and apply the model to interpret experimental observations of growth rate variations for the 100 faces of KBr crystals under different impurity concentrations.

**Figure 47:**
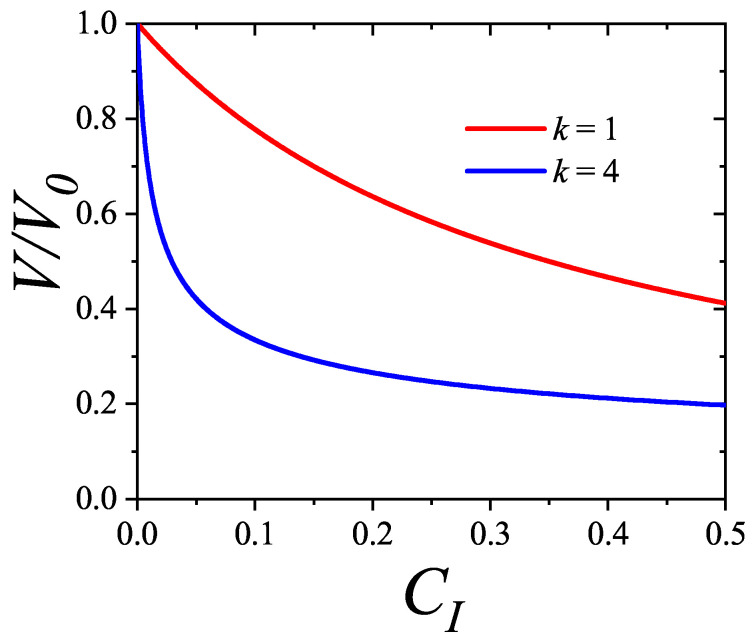
Relative step velocity $V/V_{0}$ as a function of the concentration $C_{I}$ for monomers [k=1, red line, Equation (283)] and tetramers [k=4, blue line, Equation (286)]. In the two cases, $\varepsilon_{0}/k_{B}T=-1$.

We begin by analyzing the relationship between the normalized step velocity $V/V_{0}$ and the impurity concentration $C_{I}$, considering various impurity sizes. Figure 47 presents this comparison for two specific cases: k=1 (solid line), described by Equation (283); and k=4 (dashed line), described by Equation (286). In both scenarios, the interaction energy parameter is set to $\varepsilon_{0}/k_{B}T=-1$.

As shown in Figure 47, the curve corresponding to k=4 exhibits a steeper decline at low impurity concentrations and remains consistently below the k=1 curve throughout the low- and medium-concentration regimes. As anticipated, at high impurity concentrations $C_{I}$, the curves converge and the step velocity approaches zero (this regime is omitted from the figure for sake of clarity). These results demonstrate that the model defined by Equations (283)–(286) effectively captures the essential behavior of the system. Specifically, in the low to intermediate concentration range, the ability of an impurity to suppress crystal growth is strongly influenced by its size and shape, with larger or more complex impurities exhibiting greater effectiveness. However, as $C_{I}$ increases and the surface becomes nearly saturated with impurities, the roles of size and shape become less significant and the growth suppression effect levels off.

**Figure 48:**
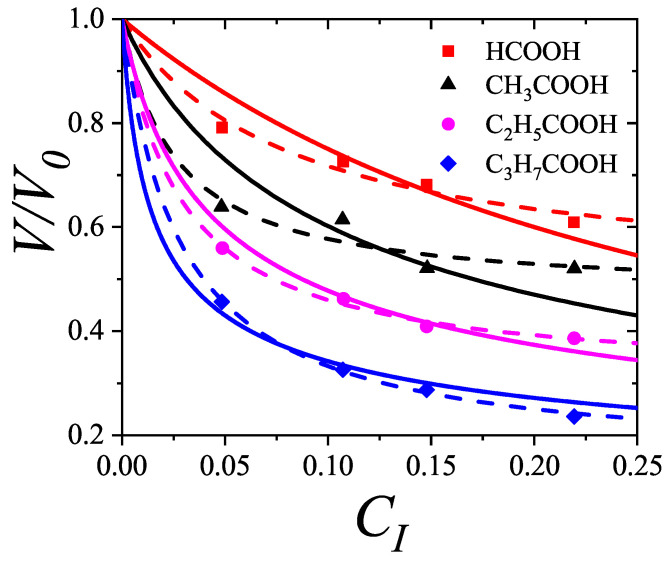
Relative growth rates of the {100} faces of KBr crystals as a function of impurity concentration. Aliphatic carboxylic acids, HCOOH, ${\mathrm{CH}}_{3}$COOH, $C_{2}$$H_{5}$COOH, and $C_{3}$$H_{7}$COOH were used as impurities. Symbols correspond to experimental data [196], solid lines represent results from Equations (283)–(286), and dashed lines correspond to KM model [193]. The parameters used in the theoretical models are listed in Table 4.

To further assess the applicability of Equations (283)–(286), we further examine an experimental case study, as illustrated in Figure 48. This analysis provides an opportunity to evaluate both the practical relevance and predictive power of the theoretical framework proposed in this work.

The experimental observations used in this analysis are drawn from the work of Bliznakov and Nikolaeva [196], who explored how aliphatic carboxylic acid impurities influence the relative growth rates of KBr crystal 100 surfaces. They reported two principal findings: (1) the growth rate of the crystal face decreases as the impurity concentration increases, and (2) the magnitude of this inhibition is correlated with the impurity size, as characterized by the number of carbon atoms present in the carboxylic acid molecule.

Subsequently, Kubota and Mullin [193] modeled these experimental results using their kinetic scheme [Equation (273)]. The KM model provided a good fit to the data, using the parameters α and K listed in Table 4. Figure 48 presents this comparison: dashed lines represent the theoretical predictions of the KM model, while the experimental data are indicated by symbols (squares for HCOOH, triangles for ${\mathrm{CH}}_{3}$COOH, diamonds for $C_{2}$$H_{5}$COOH, and circles for $C_{3}$$H_{7}$COOH). Kubota and Mullin concluded that the relatively constant values of K suggest that adsorption occurs primarily through the carboxyl group. Regarding the effectiveness factor α, they proposed that its increase with the number of carbon atoms could be attributed to the growing size or bulkiness of the impurity molecules.

We now apply our multisite adsorption model to the experimental data reported in Ref. [196], with the aim of offering an alternative perspective on the effects of impurity size. Leveraging the inclusion of the impurity size parameter *k* in Equations (283)–(286), we assign specific values of *k* based on the number of carbon atoms in the carboxylic acid molecules: HCOOH corresponds to k=1, ${\mathrm{CH}}_{3}$COOH to k=2, $C_{2}$$H_{5}$COOH to k=3, and $C_{3}$$H_{7}$COOH to k=4.

It is important to emphasize that this assignment of k=1 to 4 is a practical method to account for differences in impurity size, and does not imply any specific assumptions about the adsorption mechanism (as in the Kubota and Mullin model [193], molecular configuration in the adsorbed state is incorporated through the adsorption constant $K_{k}$). Furthermore, this approach enables a fully analytical treatment of the problem via the explicit expressions provided in Equations (283)–(286). An alternative strategy would be to treat the size parameter *k* as a fitting variable; however, the absence of a general analytical expression for $\theta (C_{I})$ makes such an analysis significantly more challenging.

Conversely, the values of $K_{k}$ for each experimental adsorption isotherm were determined by applying a standard least-squares fitting method. The analysis was carried out for four distinct scenarios:

- HCOOH: Equation (283) was employed as the fitting model, with k=1 and $K_{1}$ treated as the adjustable parameter.
- $H_{3}$COOH: The fit was performed using Equation (284), corresponding to k=2, with $K_{2}$ optimized.
- $C_{2}$$H_{5}$COOH: Equation (285) was selected for this case, with k=3 and $K_{3}$ fitted accordingly.
- $C_{3}$$H_{7}$COOH: Equation (286) served as the fitting function, with k=4 and $K_{4}$ as the fitting parameter.

The resulting fits are illustrated a solid curves in Figure 48, while the corresponding fitted values of $K_{k}$ are summarized in Table 4. It is evident that the theoretical expressions given by Equations (283)–(286) closely reproduce the experimental measurements.

The values of $K_{k}$ obtained (see the fifth column in Table 4) are notably consistent across the different impurities, supporting the conclusion that adsorption occurs primarily through the carboxylic functional groups. Additionally, the analysis based on Equations (283)–(286) strengthens the interpretation proposed by Kubota and Mullin [193]. Specifically, although the carboxylic acids are unlikely to lie flat on the surface, variations in impurity size (i.e., steric effects) have a significant impact on the behavior of the relative step velocity, $V/V_{0}$. As anticipated in Ref. [193], the effectiveness of growth inhibition increases with the molecular size of the impurity.

In summary, this section introduced a novel theoretical framework for analyzing crystal growth from aqueous solutions in the presence of structured impurities. The model is based upon physically grounded equations, involving parameters with clear physical interpretations. These parameters are experimentally accessible via thermodynamic measurements and directly reflect the spatial configuration of the impurity molecules in the adsorbed state.

The theoretical predictions were validated against Monte Carlo simulations and successfully applied to describe experimental data on the relative growth rates of the 100 faces of KBr crystals in the presence of various aliphatic carboxylic acids. These findings confirm and further support previous conclusions regarding the critical role of impurity size in modulating crystal growth behavior [193].

### 7.8. Application to k-mer Phase Transitions

In this part of the study, we utilize multiple exclusion (ME) statistics to examine the adsorption behavior of linear *k*-mers on a square lattice comprising *M* sites. For values of k≥7, this system exhibits two distinct phase transitions [124]: (1) a continuous, entropy-driven isotropic-to-nematic (I-N) transition occurring at intermediate surface coverage [124,129,131], and (2) a first-order nematic-to-isotropic (N-I) transition taking place at high densities, near full lattice occupation [133].

It is important to highlight that, for large *k*-mers (k≥7 in the case of square lattices), spontaneous orientational order emerges from purely entropic effects without the need for explicit attractive interactions between the rods. This behavior exemplifies how entropy alone can induce phase transitions in systems constrained by hard-core exclusion.

The ME statistics provide a natural and powerful framework to model the thermodynamics of these transitions, as they inherently incorporate the excluded volume effects and the reduced configurational entropy associated with rod alignment at higher densities.

#### 7.8.1. Basic Definitions

As discussed in Section 6, the problem regarding *k*-mers on the square lattice is modeled as a mixture of two species, each aligned along one of the two lattice directions: horizontal (H) and vertical (V), referred to as Hk-mers and Vk-mers, respectively. We assign Hk-mers →1 and Vk-mers →2. Both species occupy *k* consecutive sites along their respective axes.

According to the definitions established earlier, the following identifications hold: $G_{1}=M$, $G_{2}=M$, $\Delta_{12}=\Delta_{21}=1$, $N_{1,m}=G_{1}/k$, $N_{2,m}=G_{2}/k$, ${\tilde{G}}_{12,m}={\tilde{G}}_{21,m}=0$, $g_{11}=\beta_{11}=G_{1}/N_{1,m}=k$, $g_{22}=\beta_{22}=G_{2}/N_{2,m}=k$, $g_{12}=\beta_{12}=G_{1}/N_{2,m}=k$, and $g_{21}=\beta_{21}=G_{2}/N_{1,m}=k$. Furthermore, the saturation densities are $n_{1,m}=N_{1,m}/G_{1}=1/g_{11}=1/\beta_{11}=1/k$ and $n_{2,m}=N_{2,m}/G_{2}=1/g_{22}=1/\beta_{22}=1/k$, with all exclusion coefficients equal: $\beta_{12}=\beta_{21}=\beta_{11}=\beta_{22}=k$. It is important to note that, as $G_{1}=M$ and $G_{2}=M$, the states available to each species are restricted to those lying along their characteristic direction.

Regarding the saturation occupation numbers $n_{1,m}^{*}$ and $n_{2,m}^{*}$ satisfying ${\tilde{d}}_{1}\left(n_{1,m}^{*},n_{2}\right)={\tilde{d}}_{2}\left(n_{1},n_{2,m}^{*}\right)=0$ (according to Equation (241)), a fully covered lattice must fulfill

(289) $$N_{1}g_{11}+N_{2}g_{12}=G_{1}.$$

We assume that, in any macroscopic region *V* filled with $N_{1}$ particles of species 1, the average number of self-excluded states is $g_{11}$; analogously, $g_{12}$ represents the cross-excluded states from species 2 on species 1. Given a vertical occupation number $n_{2}=N_{2}/G_{2}$ for Vk-mers, the maximum horizontal occupation number is

(290) $$n_{1,m}^{*}\left(n_{2}\right)=\frac{1}{\beta_{11}}-\frac{\beta_{12}n_{2}}{\beta_{11}}$$

and, analogously,

(291) $$n_{2,m}^{*}\left(n_{1}\right)=\frac{1}{\beta_{22}}-\frac{\beta_{21}n_{1}}{\beta_{22}}.$$

For *k*-mers on the square lattice, as $\beta_{11}=\beta_{12}=\beta_{21}=\beta_{22}=k$, these simplify to

(292) $$n_{1,m}^{*}\left(n_{2}\right)=\frac{1}{k}-n_{2}=n_{1,m}-n_{2},\;n_{2,m}^{*}\left(n_{1}\right)=\frac{1}{k}-n_{1}=n_{2,m}-n_{1}.$$

The self-exclusion per particle for each species at infinite dilution corresponds to the number of states excluded by an isolated *k*-mer along its axis:

(293) $$e_{o11}=e_{o22}=2k-1=2\beta_{11}-1=2\beta_{22}-1,$$

identical to the 1D case. From Equation (252), the solutions yield $\beta_{c11}=\beta_{c22}=0$.

The number of cross-excluded states between species 1 and 2 is

(294) $$e_{o12}=k^{2}-\left(2k-1\right).$$

The first term, $k^{2}$, accounts for the initial total exclusion of perpendicular states by an isolated *k*-mer, while the second term, 2k−1, corrects for the non-independent states between directions. Two isolated *k*-mers of species 1 and 2 jointly exclude $e_{o12}+e_{o21}+2\left(2k-1\right)=2k^{2}$ states out of the G=2M total cross-states. By symmetry, $e_{o12}=e_{o21}$ and, thus,

(295) $$e_{o12}=e_{o21}=k^{2}-\left(2k-1\right)={\left(k-1\right)}^{2}.$$

This follows formally from the general relation in Equation (251), where $e_{o11}+e_{o21}+e_{o11}=k^{2}+e_{o11}=k^{2}+2k-1$. Therefore, $e_{o21}=k^{2}-\left(2k-1\right)={\left(k-1\right)}^{2}=e_{o12}$.

From the solutions to Equations (252) and (254) with $\beta_{11}=\beta_{22}=k$, the exclusion correlation parameters are $\beta_{c11}=\beta_{c22}=0$ and

(296) $$\beta_{c12}=\beta_{c21}=\frac{1}{2}\left[e_{o12}+2kW\left(-e^{-e_{o12}/2k}\right)\right],$$

where W(z) denotes the Lambert function, taking its main branch for $k\leq e_{o12}/2$ or the lower branch otherwise.

The numerical solutions for $\beta_{c12}$ are: $\beta_{c12}=11.63$ for k=6 ($e_{o12}=e_{o21}=25$), $\beta_{c12}=17.41$ for k=7 ($e_{o12}=e_{o21}=36$), $\beta_{c12}=24.10$ for k=8 ($e_{o12}=e_{o21}=49$), and $\beta_{c12}=84.26$ for k=14 ($e_{o12}=e_{o21}=169$).

#### 7.8.2. Entropy Surface, Equilibrium Path, and Order Parameter

The Helmholtz free energy per lattice site, denoted as $\beta f_{i}\left(n_{1},n_{2}\right)$, can be fully mapped within the $\left(n_{1},n_{2}\right)$ space. The average site occupancy *n*, which represents the overall occupation of the system, is related to the partial occupations of each species on their respective sublattices through $n_{1}=N_{1}/G_{1}$ and $n_{2}=N_{2}/G_{2}$, with $G_{1}=G_{2}$. This implies that $n=\left(N_{1}+N_{2}\right)/G=\left(n_{1}+n_{2}\right)/2$. The maximum achievable value for *n* is $n_{m}=1/\left(2k\right)$, which defines a straight line in the $\left(n_{1},n_{2}\right)$ plane given by $n_{m}=\left(n_{1,m}^{*}+n_{2}\right)/2=\left(n_{1}+n_{2,m}^{*}\right)/2=1/2k$, referred to as the saturation line.

To identify equilibrium states at a fixed mean occupation *n*, the free energy function $\beta f_{1}\left(n_{1},n_{2}\right)=\beta f_{1}\left(n_{1},2n-n_{1}\right)$ is minimized numerically. The resulting coordinates $\left(n_{1},n_{2}\right)$ where local minima occur correspond to equilibrium configurations.

Given the athermal nature of the system, where only hard-core (excluded volume) interactions are considered and the interaction energies $\epsilon_{1}=\epsilon_{2}=0$, the internal energy vanishes. Consequently, the free energy minima $\beta f_{1}\left(n_{1},n_{2}\right)$ are directly associated with maxima in the entropy per site (in units of $k_{B}$). An orientational order parameter O(n) is defined using the difference in occupation between the two species as a measure of nematic alignment.

(297) $$O\left(n\right)=\frac{\left|n_{2}-n_{1}\right|}{n_{1}+n_{2}},\;0\leq O\left(n\right)\leq 1$$

where O(n)=0 indicates isotropic *k*-mer distribution, while 0<O(n)≤1 reflects nematic ordering.

For k≤5, all solutions satisfy $n_{1}=n_{2}$ for any *n*, implying O(n)=0 and absence of phase separation: both species distribute equally.

In contrast, for k≥7, phase separation occurs beyond a critical density $n_{c}$. A nematic phase emerges, where one direction becomes favored. The particular case of k=6 deserves a detailed discussion.

**Figure 49:**
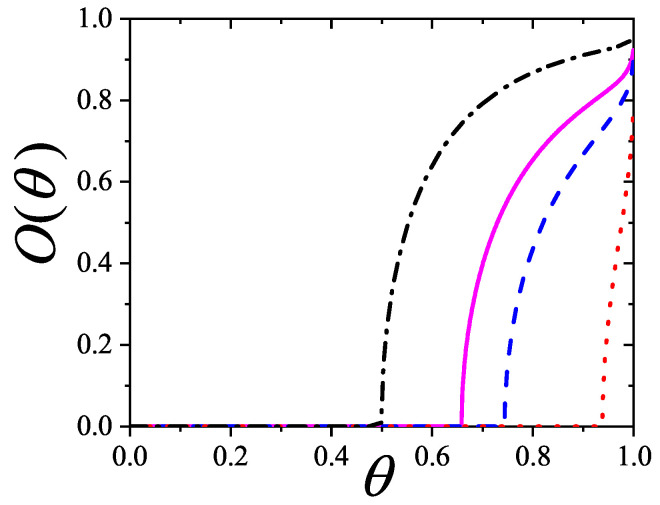
The orientational order parameter O(θ) for several cases: k=6 with $e_{o12}=24$ and $\beta_{c12}=11.04$ (dotted line); k=7 with $e_{o12}=36$ and $\beta_{c12}=17.41$ (dashed line); another case for k=7 with $e_{o12}=34$ and $\beta_{c12}=16.00$ (solid line); and k=8 with $e_{o12}=49$ and $\beta_{c12}=24.10$ (dash-dotted line). The approximate critical coverages for the nematic phase transition in these scenarios are $\theta_{c}\to 1$, 0.659, 0.744, and 0.5, respectively. In all instances, the corresponding density of states functions ${\tilde{d}}_{1}\left(n_{1},n_{2}\right)$ and ${\tilde{d}}_{2}\left(n_{1},n_{2}\right)$ (and, thus, the entropy surface $S(n_{1},n_{2})$) are defined to vanish along the saturation boundary in the $\left(n_{1},n_{2}\right)$ plane.

Although the model predicts phase separation at very high coverage for k=6 (see Figure 49), this behavior results from the Helmholtz free energy vanishing at saturation—i.e., $\beta f_{1}\left(n_{1,m}^{*},n_{2,m}^{*}\right)=\beta f_{1}\left(n_{1},1/k-n_{1}\right)=\beta f_{2}\left(1/k-n_{2},n_{2}\right)=0$ and $S_{1}\left(n_{1,m}^{*},n_{2,m}^{*}\right)=S\left(n_{1},1/k-n_{2}\right)=S\left(1/k-n_{2},n_{2}\right)=0$—according to Equations (234) and (235), due to the restrictive boundary conditions ${\tilde{d}}_{1}\left(n_{1,m}^{*},n_{2,m}^{*}\right)={\tilde{d}}_{2}\left(n_{1,m}^{*},n_{2,m}^{*}\right)=0$ imposed at saturation. It is worth noting that, for k=7,8, the order parameter do not show any transition to an isotropic high-coverage regime as expected from MC simulations. However, if the entropy reaches a finite value $S_{1}\left(n_{1,m}^{*},n_{2,m}^{*}\right)\geq 0$ at full coverage instead of vanishing, this anomalous phase separation for k=6 would not occur and, as shown latter, the high-coverage nematic-to-isotropic transition does occur for k≥7.

When adopting either a more general form for the density of states (as introduced in Section 7.8.3) or adding an *ad hoc* high-density correction to the entropy surface, this spurious behavior is eliminated. Consequently, the system does not exhibit phase transitions for k=6, consistent with prior single-species analyses of nematic transitions discussed earlier in Section 5. For k=6, the entropy at high coverage is exceedingly close to—but still higher than—that under the nematic regime. Thus, a highly accurate approximation is required to capture the MC simulation result that nematic ordering only occurs for k≥7. The subtle dependence of entropy at high coverage critically determines the absence of nematic transition for k≤6 and its appearance for k≥7 [129,197].

For k≥7, there exists a critical state occupation $n_{c}$ such that, for $n\geq n_{c}$, phase separation ($n_{2}\neq n_{1}$) occurs and *k*-mers preferentially align along a lattice direction, forming a nematic phase. Even under the restrictive condition of vanishing entropy at saturation, the model predicts, for k=7, $e_{o12}=36$, $\beta_{c12}=\beta_{c21}=17.41$, a critical state occupation $n_{c}\approx 0.0471$, corresponding to $\theta_{c}=2kn_{c}\approx 0.659$, and for $e_{o12}\approx 34$, $\beta_{c12}=\beta_{c21}=16.00$, a critical value $n_{c}\approx 0.0532$, $\theta_{c}\approx 0.7448$. These values are very close to the known MC simulation results $n_{c}\approx 0.053214$ and $\theta_{c}\approx 0.745$ [129], and also agree with the earlier estimates reviewed in Section 5 [18].

Remarkably, the complex many-body correlations are captured by a single cross-exclusion parameter $\beta_{c12}=\beta_{c21}$, yielding surprisingly accurate predictions. The input boundary values that best match the MC results, $\beta_{c12}=\beta_{c21}=16$ ($e_{o12}=e_{o21}=34$), are close to—but slightly lower than—the analytical solution value $\beta_{c12}=\beta_{c21}=17.41$ ($e_{o12}=e_{o21}=36$) obtained from Equation (254). This highlights the already significant accuracy of the first-order approximation assuming constant $\beta_{c12}$ with vanishing entropy constraints, although it also suggests that $\beta_{12}$ may vary slowly with *n*, as proposed in the second-order approximation of ME statistics discussed in the previous section.

Thus, the critical density predicted by the model is highly sensitive to the cross-exclusion parameter $\beta_{12}$. In this elaborated approach, compared to the one presented in Section 5, the first-order approximation is kept and $\beta_{12}=\beta_{21}$ is set to be a constant, as many essential conclusions can already be drawn. Overall, the present formalism reveals that the emergence of nematic order for k≥7 is mainly driven by cross-excluded volume effects encoded in $\beta_{12}$, with critical values finely tuned by its magnitude.

To better match the observed behavior at high coverage, an empirical entropy contribution $\Delta S(n_{1},n_{2})$ was introduced to the theoretical entropy $S(n_{1},n_{2})$ derived previously. This correction accounts for the finite entropy observed in simulations even at full lattice coverage, as the lattice exhibits local rearrangement of *k*-mers patches.

The proposed empirical correction is given by:

(298) $$\Delta S\left(n_{1},n_{2}\right)=\Delta S_{hc}\left(n_{1},n_{2}\right)\left[1-{\left(\frac{|\frac{n_{1}}{2}-\frac{n_{2}}{2}|}{n}\right)}^{\delta }\right],$$

where

(299) $$\Delta S_{hc}\left(n_{1},n_{2}\right)=S_{DR}{\left(\frac{n}{n_{m}^{*}}\right)}^{\alpha }\exp\left[-\frac{n-n_{m}^{*}}{\gamma n_{m}^{*}}\right].$$

Here, $n=(n_{1}+n_{2})/2$ is the mean occupation, $n_{m}^{*}=1/\left(2k\right)$ the maximum occupation, and $S_{DR}$ the saturation entropy at full coverage. The parameters α, δ, and γ control the behavior near saturation. Specifically, $S_{DR}$ follows $S_{DR}=c\left(k\right)\ln k/k^{2}$ with c(k) fitted from MC simulations: c(k)=1.69,1.30,1.16,1.08,1.04,1.01 for k=2 to k=7 respectively, and c(k)≈1 for k≥8.

The correction satisfies ΔS(0,0)=0, $\Delta S\left(n_{m}^{*},n_{m}^{*}\right)=S_{DR}$ for isotropic full coverage, and ΔS=0 for fully aligned *k*-mers at saturation (either $n_{1}=1/k$, $n_{2}=0$, or vice versa), thus recovering the one-dimensional limit.

Consequently, the corrected entropy surface $S_{c}\left(n_{1},n_{2}\right)$ becomes:

(300) $$S_{c}\left(n_{1},n_{2}\right)=S\left(n_{1},n_{2}\right)+\Delta S\left(n_{1},n_{2}\right),$$

with $S\left(n_{1},n_{2}\right)/k_{B}=-\beta f_{1}\left(n_{1},n_{2}\right)$ again being the entropy surface vanishing at the saturation line $n_{m}=\left(n_{1,m}^{*}+n_{2}\right)/2=\left(n_{1}+n_{2,m}^{*}\right)/2=1/2k$. This correction becomes significant at high occupations ($n\to n_{m}^{*}$), especially for small γ values.

The values α=1, δ = 1.65–1.75, and γ = 0.05–0.06 were adjusted to reproduce the chemical potentials and transition coverages observed in simulations, as discussed below.

**Figure 50:**
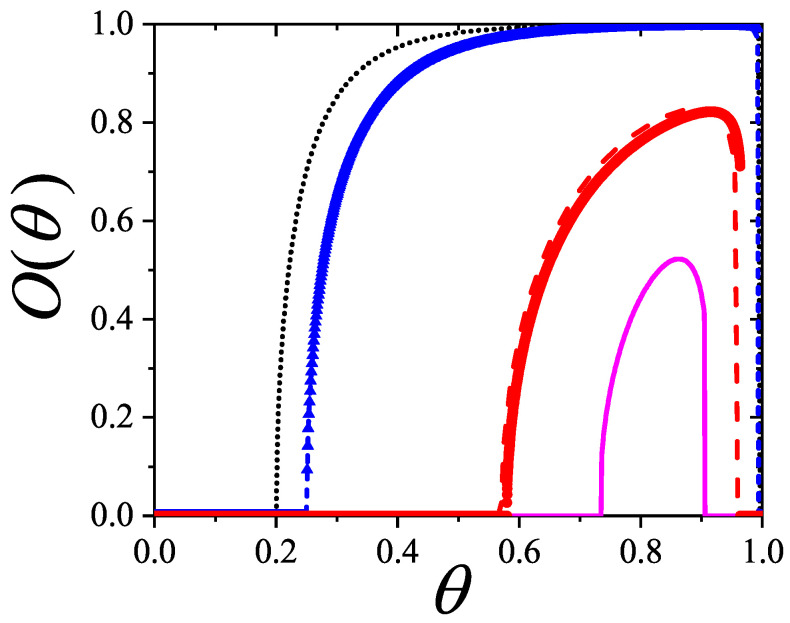
O(θ) as a function of coverage for k=7 (solid curve, $\beta_{c12}=17.41$), k=8 (dashed, $\beta_{c12}=22.0$), k=12 (short-dashed, $\beta_{c12}=60.42$), and k=14 (dotted, $\beta_{c12}=84.26$). These results incorporate the empirical correction for high-density entropy, $\Delta S_{hc}\left(n_{1},n_{2}\right)$, following Equations (298)–(299), with parameters set to δ=1.75, α=1, and γ ranging from 0.05 to 0.06. Superimposed solid markers for k=7 and k=12 correspond to analytical predictions obtained using the generalized density of states from Equation (301) within the ME statistics framework. Specifically, these refer to k=8, $\beta_{c12}=22.5$ and k=12, $\beta_{c12}=60.42$.

Figure 50 shows the resulting order parameter O(θ) as a function of coverage θ for k=7, 8, 12, and 14, when the high-coverage empirical entropy correction $\Delta S(n_{1},n_{2})$ is included. The high-coverage entropy contribution not only predicts the isotropic-nematic transition at intermediate densities but also captures the nematic-to-isotropic transition at high densities, as observed in MC simulations.

The critical densities for the isotropic–nematic transition obtained from the model were very close to the simulation results. For instance, for k=7, the model predicted $n_{c}\approx 0.0531$ ($\theta_{c}\approx 0.744$) while, for k=8, $n_{c}\approx 0.0312$ ($\theta_{c}\approx 0.5$). These values are in excellent agreement with MC simulations reported in Refs. [129,131].

Furthermore, the model predicted a sharp drop in the order parameter for k=7 at $n_{c}=0.065$ (corresponding to θ≈0.917), associated with the nematic-to-isotropic transition. Predictions for k=12 and k=14 are also provided, yielding $\theta_{c}\approx 0.25$ and $\theta_{c}\approx 0.2$, respectively. Although simulation data for these higher *k*-values are scarce, the model results suggest that the critical coverages should be slightly lower, as already observed for smaller *k*.

Although the curves in Figure 50 appear to show continuous transitions at high density, a more detailed analysis (discussed later in Section 7.8.4) reveals that the nematic-to-isotropic transition at high coverage is indeed of first order, as indicated by a discontinuous jump in the chemical potential.

**Figure 51:**
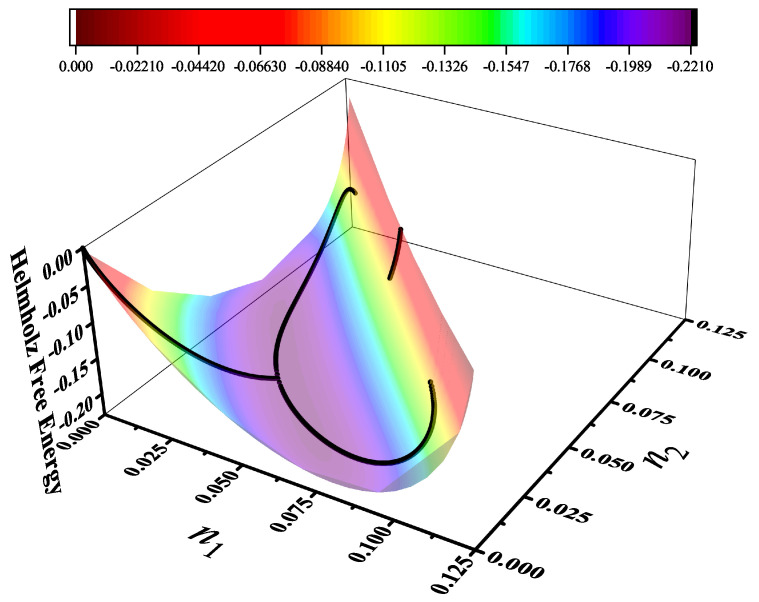
The full Helmholtz free energy landscape for k=8, plotted as a function of occupation numbers $n_{1}$ and $n_{2}$ along both lattice directions. Equilibrium states are highlighted as black dots, and the distinct branches represent the coexistence of low- and high-density phases associated with the nematic transition.

Additionally, the full Helmholtz free energy surface $\beta f_{1}\left(n_{1},n_{2}\right)$ can be derived analytically. Figure 51 displays this surface for k=8. The equilibrium states follow the black solid curve shown over the surface. For $n<n_{c}$, the equilibrium corresponds to isotropic configurations with $n_{1}=n_{2}$. As *n* increases beyond $n_{c}$, two symmetric branches emerge, which correspond to nematic ordering along either the horizontal or vertical direction. These branches are symmetric with respect to the isotropic bisector $n_{1}=n_{2}$ and represent coexistence between a high-density aligned phase and a low-density dilute phase.

Specifically, for $n>n_{c}$, two minima of $\beta f_{1}\left(n_{1},n_{2}\right)$ exist at points $\left(n_{1},2n-n_{1}\right)$ and $\left(2n-n_{2},n_{2}\right)$ with $n_{1}\neq n_{2}$, which are symmetrically located with respect to the line $n_{1}=n_{2}$. If $n_{2}>n_{1}$, the high-density phase is oriented along the vertical direction; otherwise, it is aligned horizontally. This analytical description of the density branches in the nematic phase constitutes an important result of the present formalism.

The Helmholtz free energy difference between the isotropic and nematic phases is very small, indicating that the nematic phase is only weakly more stable. It is also highly sensitive to perturbations, such as weak additional interactions, which could further stabilize or destabilize the nematic phase depending on their nature.

The discussed results and predictions remain valid even when the empirical entropy correction $\Delta S(n_{1},n_{2})$ is not explicitly included. As detailed below, the general formulation of ME statistics allows one to obtain equivalent behavior by simply relaxing the constraint of zero entropy at saturation, setting non-zero values for the density of states at full coverage.

Thus, the empirical entropy correction serves primarily as a practical and illustrative tool for matching MC data and providing physical insights, while the general ME formalism ensures the robustness of the theoretical predictions.

#### 7.8.3. Generalized Density of States Function in Multiple Exclusion Statistics

Beyond the analysis of the high-coverage phase transitions conducted in the previous section through introducing an empirical entropy correction in the $\left(n_{1},n_{2}\right)$ plane via Equations (298) and (299), an analogous behavior naturally arises from the ME statistics when adopting a less restrictive boundary condition for the density of states functions in Equation (241). Instead of enforcing ${\tilde{d}}_{i}\left(n_{1,m}^{*},n_{2,m}^{*}\right)=0$, corresponding to a fully oriented phase at saturation (as in the previous approximation), we now consider ${\tilde{d}}_{i}\left(n_{1,m}^{*},n_{2,m}^{*}\right)>0$.

Indeed, the strict condition ${\tilde{d}}_{i}=0$ is valid only when the lattice is saturated entirely by Hk-mers or Vk-mers, with occupations $\left(n_{1,m},0\right)$ or $\left(0,n_{2,m}\right)$, respectively. However, for other saturation states along the line $n_{1}+n_{2}=n_{1m}=n_{2m}$, where both types of *k*-mers coexist, the density of states at saturation is expected to remain finite.

To demonstrate that similar results regarding the order parameter and phase transitions emerge from this generalization, we adopt the following expressions for the density of states functions:

(301) $$\begin{array}{cc} {\tilde{d}}_{1}\left(n_{1},n_{2}\right) & ={\tilde{G}}_{12}\left(n_{2}\right)\left[e^{\beta_{c11}n_{1}}-\left(e^{\beta_{c11}n_{1,m}^{*}}-{\tilde{d}}_{s,1}\right)\frac{n_{1}}{n_{1,m}^{*}}\right],\\ {\tilde{d}}_{2}\left(n_{1},n_{2}\right) & ={\tilde{G}}_{21}\left(n_{1}\right)\left[e^{\beta_{c22}n_{2}}-\left(e^{\beta_{c22}n_{2,m}^{*}}-{\tilde{d}}_{s,2}\right)\frac{n_{2}}{n_{2,m}^{*}}\right], \end{array}$$

where ${\tilde{d}}_{s,1}$ and ${\tilde{d}}_{s,2}$ denote the finite density of states per particle for species 1 and 2 at the saturation line, corresponding respectively to $n_{1,m}^{*}\left(n_{2}\right)=n_{1,m}-n_{2}$ and $n_{2,m}^{*}\left(n_{1}\right)=n_{2,m}-n_{1}$.

It should be noted that ${\tilde{d}}_{s,1},\;{\tilde{d}}_{s,2}$ increase smoothly along the saturation line from zero for a fully oriented phase, $\left(n_{1},n_{2}\right)=\left(n_{1,m},0\right)$ or $\left(n_{1},n_{2}\right)=\left(0,n_{1,m}\right)$, up to ${\tilde{d}}_{s,I}$ for an isotropic phase $\left(n1,n2\right)=\left(n_{1,m}/2,n_{1,m}/2\right)$. Moreover, ${\tilde{d}}_{s,1}$ and ${\tilde{d}}_{s,2}$ vary according to a functional form analogous to Equation (298) along the saturation line, with the first term being ${\tilde{d}}_{s,1}\left(n_{1,m}^{*},n_{2,m}^{*}\right)={\tilde{d}}_{s,2}\left(n_{1,m}^{*},n_{2,m}^{*}\right)={\tilde{d}}_{s,I}$.

(302) $$\begin{array}{cc} {\tilde{d}}_{s,1}\left(n_{1},n_{2}\right) & ={\tilde{d}}_{s,I}\left[1-{\left(\frac{\left|\frac{n_{1}}{2}-\frac{n_{2}}{2}\right|}{n}\right)}^{\delta }\right],\\ {\tilde{d}}_{s,2}\left(n_{1},n_{2}\right) & ={\tilde{d}}_{s,I}\left[1-{\left(\frac{\left|\frac{n_{1}}{2}-\frac{n_{2}}{2}\right|}{n}\right)}^{\delta }\right], \end{array}$$

here, the equations are meant to be valid for (n1,n2) along the saturation line. Thus, given $n_{2}$, $n_{1}=n_{1,m}^{*}\left(n_{2}\right)=n_{1,m}-n_{2}$ is fixed or, conversely, given $n_{1}$, we have $n_{2}=n_{2,m}^{*}\left(n_{1}\right)=n_{2,m}-n_{1}$.

The values ${\tilde{d}}_{s,I}={\tilde{d}}_{s,1}\left(n_{1,m}/2,n_{2,m}/2\right)={\tilde{d}}_{s,2}\left(n_{1,m}/2,n_{2,m}/2\right)={\tilde{d}}_{s,I}$ for an isotropic saturated phase are as follows: ${\tilde{d}}_{s,I}$ = 0.0560, 0.0276, 0.0162, 0.0106, 0.0075, 0.0055, 0.0043, 0.0035, 0.0026 for k=2 to k=10, respectively, as introduced in [18] of this series to match the MC values for the entropy at full coverage [116,117,134].

Thus, the entropy functions from Equation (300), the one from ME statistics (Equation (235)) for the general density of states forms, and Equations (301) and (302) evaluated along the saturation line cases differ with respect to each other by less than 4% of ${\tilde{d}}_{s,I}$; meanwhile, at the fully aligned or fully isotropic limits, they take the identical desired values. Ultimately, the two free energy surfaces involved in the calculations compared in this section take approximately the same values along the saturation line in the plane (n1,n2).

As elaborated in the following section, employing the generalized density of states functions from Equation (301) yields an entropy surface that successfully reproduces both the qualitative and quantitative features of the phase transitions observed in MC simulations. This analytical formulation captures the nematic branches, reveals the nature (continuous or discontinuous) of the transitions, and accurately reflects the first-order character of the high-density transition recently verified for k≥8 (and likely for k=7 as well) in Ref. [133].

#### 7.8.4. Nematic-Phase Density Branches and Phase Transitions

With regard to adsorption isotherms, this theoretical framework provides expressions for both the low- and high-density branches. To the best of our knowledge, this represents the first time such results have been derived analytically for *k*-mers adsorbed onto a square lattice. These theoretical predictions are compared with corresponding data obtained from accelerated MC simulations.

As described in Section 5.5, simulations were performed in the grand canonical ensemble using the method introduced by Kundu et al. [63,129,137], which is particularly effective in overcoming the slowdown associated with high-density configurations. For each value of βμ, the system evolves by means of non-local insertion and removal of *k*-mers. Specifically, each MC step begins by removing all *k*-mers aligned along one direction (e.g., horizontal), while keeping those in the orthogonal direction (vertical) unchanged. The exact probabilities of forming empty segments of various lengths are pre-computed and used to repopulate the lattice with *k*-mers accordingly. A symmetric process is then applied in the other direction. The algorithm preserves detailed balance and is fully ergodic.

**Figure 52:**
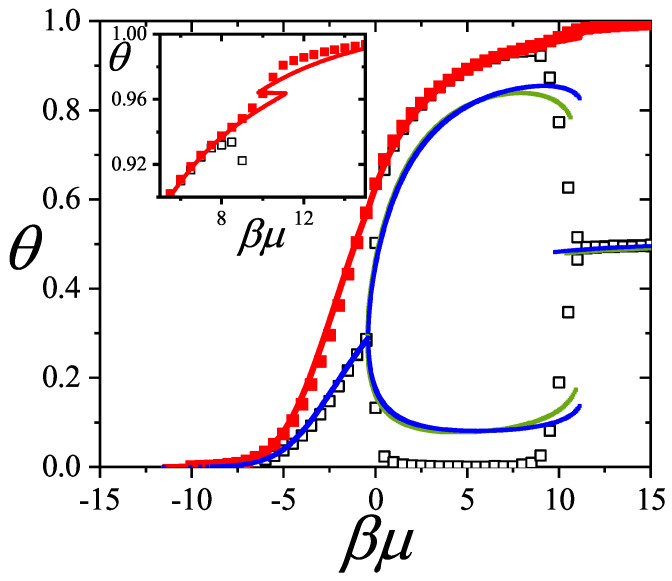
The variation of lattice coverage θ with respect to βμ for k=8. Theoretical predictions derived from the ME approach using the generalized density of states Equation (301) are illustrated: blue curves indicate the low-density (lower) and high-density (upper) branches, while the red curve shows the total coverage. The inset emphasizes the first-order nature of the high-coverage transition. Green lines (which nearly overlap with the theoretical curves) correspond to results incorporating the empirical entropy correction. Simulation data from Monte Carlo calculations are plotted as symbols. The model parameters used were: $\beta_{11}=\beta_{22}=8$, $e_{o11}=e_{o22}=15$, $\beta_{c11}=\beta_{c22}=0$, $\beta_{12}=\beta_{21}=8$, $e_{o12}=e_{o21}=45$, $\beta_{c12}=\beta_{c21}=22.0$, δ=1.75, α=1.0, γ=0.05.

**Figure 53:**
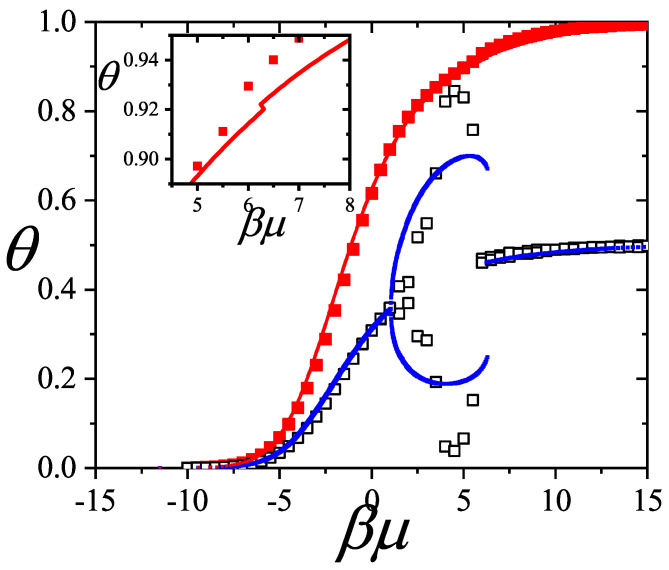
Similar to Figure 52, but for k=7. Parameters: $\beta_{11}=\beta_{22}=7$, $e_{o11}=e_{o22}=13$, $\beta_{c11}=\beta_{c22}=0$, $\beta_{12}=\beta_{21}=7$, $e_{o12}=e_{o21}=35-36$, $\beta_{c12}=\beta_{c21}=17.0$, δ=1.75, α=1.0, γ=0.06.

Simulations were carried out on square lattices of size L×L, with L/k=120, under periodic boundary conditions. Finite-size effects were found to be negligible. Typically, equilibrium was reached after $10^{7}$ MC steps. The resulting data for the adsorption branches, as shown in Figure 52 and Figure 53, were obtained by averaging the coverage (or occupation number) over $10^{7}$ MC steps, regardless of the *k*-mer orientation in individual configurations.

The analytical densities for the high- and low-density phases, $n_{1}$ and $n_{2}$, were obtained as functions of the chemical potential of the gas-phase, βμ, by solving for $n_{1}$ and $n_{2}$ in the pair of coupled equations $\beta \mu_{1}\left(n_{1},n_{2}\right)=\beta \mu_{2}\left(n_{1},n_{2}\right)=\beta \mu$ [Equations (242) and (243)], assuming equilibrium between the two *k*-mer species and the *k*-mer gas in the reservoir. The mean occupation and lattice coverage are given by $n=(n_{1}+n_{2})/2$ and $\theta =2kn=kn_{1}+kn_{2}=\theta_{1}+\theta_{2}$, where $\theta_{1}$ and $\theta_{2}$ represent coverage along the horizontal and vertical directions, respectively.

The analytical results are compared to MC simulations for k=8 and k=7 in Figure 52 and Figure 53. Two theoretical approximations are shown: (1) the results obtained when adding the empirical high-density entropy correction, Equation (298), to the basic entropy surface corresponding to density of states functions vanishing at full coverage [Equation (241)], resulting in the entropy surface of Equation (300), displayed as green lines; and (2) the results from the entropy surface corresponding to generalized density of states functions [Equation (301)], shown as blue lines. As can be seen from the figures, both approaches yielded very similar results. It worth noting that we refer to entropy surfaces instead of the Helmholtz Free Energy surface many times, given that $\beta f\left(n_{1},n_{2}\right)=-S\left(n_{1},n_{2}\right)/k_{B}$ in this problem as $\epsilon_{1}=\epsilon_{2}=0$.

The chemical potential versus density curves (adsorption isotherms) were very well reproduced up to saturation. At low coverage, an isotropic phase was found to prevail; as density increases, the nematic branches emerge and, finally, a transition to a disordered isotropic phase occurs. The nematic branches and coexistence region are qualitatively well captured.

The isotropic–nematic transition is clear for k=7 (Figure 53) and k=8 (Figure 52), with critical points at approximately βμ=−0.417, θ=0.576 (k=8) and βμ=1.27, θ=0.74 (k=7). The transition is continuous, as evidenced by the continuous chemical potential dependence, matching the MC results.

**Figure 54:**
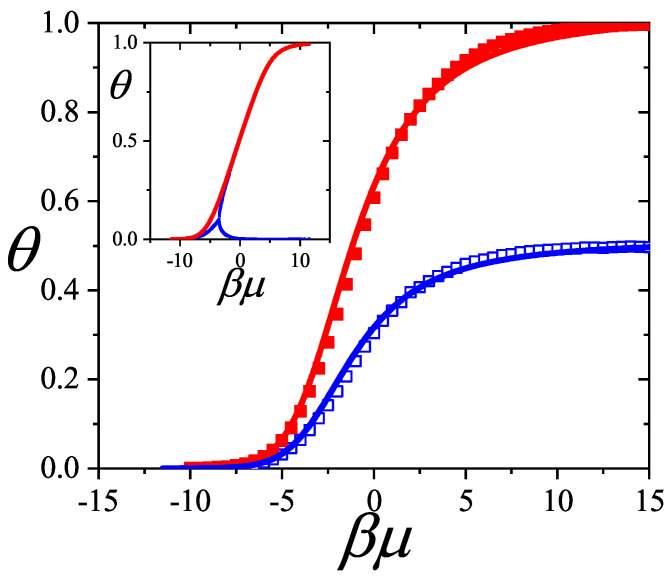
Coverage θ vs. βμ for k=6 (main figure) and k=14 (inset). Lines show theoretical ME statistics results with generalized density of states Equation (301). MC data are shown as symbols.

No transition occurs for k≤6 (Figure 54). In that case, the theoretical and MC results show that $\theta_{1}=\theta_{2}$ for all densities, confirming the absence of ordering. The inset shows k=14 as an example, where the branching is much more pronounced.

At high coverage, a nematic-to-isotropic transition is predicted. For k=8, the branches collapse around βμ≈ 9.78–11.16, corresponding to a coverage jump Δθ≈0.015 indicating a first-order transition. The critical chemical potential estimated via Maxwell construction is $\beta \mu_{c}\approx 10.5$, close to the prediction βμ≈kln(k/lnk) from Ref. [133]. While the predicted coverage jump (≈0.015) is smaller than the value ≈0.028 reported by Shah et al. [133], the trend is consistent.

For k=7, the behavior is more sensitive: a small discontinuous jump appears, depending critically on $\beta_{c12}$. Analytical predictions give $\beta \mu_{c}\approx 5.72$ (θ≈0.91) for $\beta_{c12}=16.7$, and $\beta \mu_{c}\approx 6.27$ (θ≈0.92) for $\beta_{c12}=17.0$.

Overall, while a more detailed finite-size scaling analysis is needed through MC simulations, these results suggest a first-order nematic-to-isotropic transition for k≥8, consistent with the MC results and previous theoretical work, and also for k=7 (although less conclusively).

#### 7.8.5. State Exclusion Spectrum Functions of *k*-mers: Coverage Dependence

The self- and cross-exclusion spectrum functions, represented by $e_{ij}$ and $G_{ij}$, respectively, are formally linked to the density dependence of the chemical potential. These functions serve as a refined tool for capturing the effects of spatial correlations on statistical exclusion phenomena. They allow for a deeper understanding of the system’s thermodynamic behaviors across the isotropic–nematic transition and within the coexistence region. Additionally, they suggest a new route for experimentally detecting spatial ordering in adsorbed particles through thermodynamic observables; something that traditional adsorption isotherms alone often fail to reveal, as is already evident from the *k*-mer model data.

**Figure 55:**
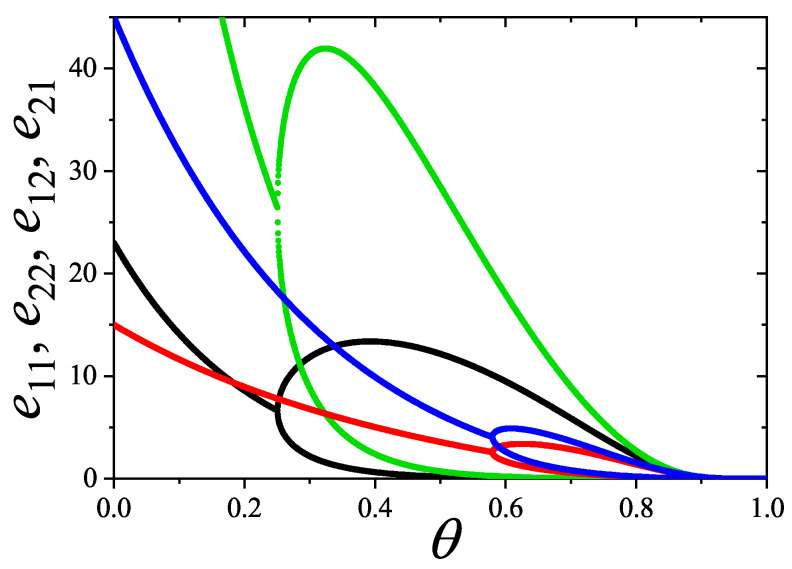
Self-and cross-exclusion frequency functions: red (k=8) $e_{22}$ (lower branch) and $e_{11}$ (upper branch); blue (k=8) $e_{12}$ (lower) and $e_{21}$ (upper); black (k=12) $e_{22}$ and $e_{11}$; green (k=12) $e_{12}$ and $e_{21}$. Analytical results from Equation (250) with density of states from Equation (301).

**Figure 56:**
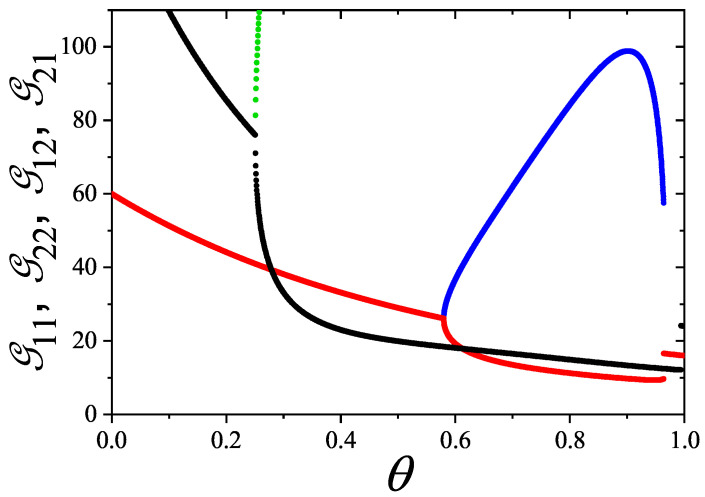
Average exclusion per particle spectrum functions for k=8 and k=12. Red and black: $G_{22}=G_{12}$; blue and green: $G_{11}=G_{21}$. Analytical results from ME statistics formalism.

We now turn to a statistical analysis of phase behavior in the *k*-mer lattice system, using the frequency-based self- and cross-exclusion measures per particle—namely, $e_{11}$, $e_{22}$, $e_{12}$, and $e_{21}$ (Figure 55)—along with the corresponding integrated exclusion per particle functions G11, G22, G12, and G21 (Figure 56). These were defined earlier in Section 6.3 via Equations (249) and (250).

For k<7, all exclusion functions decrease monotonically with coverage from their infinite dilution limits, $e_{o11}=e_{o22}$ and $e_{o12}=e_{o21}$, down to zero. Moreover, $e_{11}=e_{22}$ and $e_{12}=e_{21}$ at all densities, indicating an isotropic phase where the average number of self-excluded and cross-excluded states by a H- or V-oriented *k*-mer are identical for each species. As a result, the average number of states excluded per particle decreases with increasing coverage, although the decrease is not linear due to the ME statistical effects.

For k≥7, the exclusion functions $e_{11}$, $e_{22}$, $e_{12}$, and $e_{21}$ also decrease from θ=0 up to the critical coverage $\theta_{c}$, maintaining $e_{11}=e_{22}$ and $e_{12}=e_{21}$. At $\theta_{c}$, they split into two distinct branches, remaining continuous but with a discontinuous first derivative. If we assume, for simplicity, that the high-density nematic phase aligns along the V direction (species 2), then $e_{22}$ represents self-exclusion along V per V-oriented particle, while $e_{11}$ corresponds to the same along H for H-oriented particles. Similarly, $e_{21}$ refers to cross-exclusion in V by a H-oriented particle, and $e_{12}$ is the reciprocal.

After the transition at $\theta_{c}$, the behavior is as follows: (1) the decreasing branch of $e_{22}$ indicates stronger alignment and denser packing along V; (2) the decreasing $e_{12}$ reflects compact transversal packing; (3) the branch of $e_{11}$ increases sharply beyond $\theta_{c}$, indicating more dispersed H-oriented particles; and (4) $e_{11}$ reaches a maximum and then decreases slightly as density increases.

As $e_{22}<e_{11}$ and $e_{12}<e_{21}$ for $\theta >\theta_{c}$, the V-oriented phase is always more aligned and compact than the H one.

While all $e_{ij}$ vanish at full coverage, the cumulative exclusion functions $G_{ij}$, as defined in Section 6.3, are highly sensitive to changes around the transitions. They split at $\theta_{c}$ and show a discontinuous jump at the high-density transition, consistent with its first-order character.

Thus, the functions $G_{ij}$ provide sensitive and valuable analytical tools to trace phase transitions and characterize lattice configurations. Importantly, $G_{21}$ and $G_{12}$ can be easily computed via MC simulations.

Regarding the cross-exclusion parameters $\beta_{c12}=\beta_{c21}$, they quantify the strength of the statistical interaction between H- and V-oriented *k*-mers. The leading term ${\tilde{G}}_{12}\left(n_{2}\right)∼e^{-\beta_{c12}n_{2}}$ shows that $\beta_{c12}$ controls the exponential decay of available states for H particles as V particles fill the lattice. Changing variables to $n_{2}=\theta_{2}/\beta_{22}$ and $\theta =\theta_{1}+\theta_{2}=2\theta_{2}$ in the isotropic phase, we have

(303) $${\tilde{G}}_{12}\left(\theta_{2}\right)∼e^{-\beta_{c12}\theta /2\beta_{22}}$$

and, thus,

(304) $$\theta_{c}∼\frac{2\beta_{22}}{a\beta_{c12}},$$

where a≈1−1.3 for k=6−14. For k=6, $2\beta_{22}/\beta_{c12}=12/11.6>1$; thus, no nematic transition is expected.

Qualitatively, in terms of state exclusion statistics, it is the strong depletion of available states for a given orientation—driven by the density of particles in the perpendicular direction—that induces reordering into a nematic phase coexisting with a less dense transverse phase.

In summary, the exclusion statistics approach outlined in Section 6 was employed to tackle the complex problem of *k*-mer adsorption onto a square lattice, particularly for large values of *k* (k≥7), where the system exhibits at least two distinct phase transitions. The system was described statistically as a binary mixture of two species with different orientations that exhibit both self- and mutual exclusion. Alternatively, this can be interpreted as a population of identical particles distributed across two distinct state sets, subject to mutual and self-exclusion constraints imposed by their spatial correlations. The analytical framework based on multiple exclusion (ME) statistics predicts a continuous isotropic-to-nematic transition at intermediate surface coverage, which arises only for k≥7 and, importantly, a first-order nematic-to-isotropic transition at high densities, which was clearly present for k≥8, and less definitively for k=7. The theoretical predictions match well with simulation data, both qualitatively and quantitatively, capturing the behavior of the chemical potential, the location of the phase transitions, and the shape of the density branches in the nematic region, including the coexistence line. This formalism provides reliable analytical approximations for the full Helmholtz free energy and entropy landscapes as functions of the occupation numbers $n_{1}$ and $n_{2}$, corresponding to horizontally and vertically oriented *k*-mers. Equilibrium configurations are obtained by minimizing the free energy at a given mean lattice occupation *n*. The model reproduces the low- and high-density segments of the adsorption isotherms, with good agreement when compared to Monte Carlo results. Moreover, a unified thermodynamic framework was established through the introduction of averaged state exclusion functions. These functions are expressed in terms of the chemical potential and coverage of *k*-mers, allowing for the derivation of key statistical correlation parameters from the behavior at phase boundaries.

## 8. Monte Carlo Simulation Method Applied to the Problem of Adsorption with Multisite Occupancy

In the following sections, we present different computational algorithms of interest for the study of adsorption problems involving multisite occupancy. Most of these algorithms were used throughout the present work.

### 8.1. Metropolis MC Algorithms for Adsorption of Interacting *k*-mers

#### 8.1.1. Grand Canonical Ensemble

In order to simulate the adsorption/desorption process of *k*-mers in the grand canonical ensemble, we use a generalized MC algorithm based on the Metropolis scheme of transition probabilities [198].

The procedure begins with a system composed of *M* sites characterized by a connectivity γ, operating under conditions of temperature *T* and pressure *P* (or, alternatively, chemical potential μ). The simulation process involves repetition of the following elementary steps (MCS):

(i) Specify the chemical potential μ and the temperature *T*.
(ii) Randomly select a linear group of *k* adjacent sites.
(iii) If all the *k* sites chosen in step ii) are vacant, a rod insertion is attempted with probability $W_{ads}$. If instead the selected sites are fully occupied by segments of the same *k*-mer, a removal (desorption) attempt is made with probability $W_{des}$. In any other scenario, the attempt is rejected. Here, $W_{ads}$ and $W_{des}$ correspond to the transition probabilities for increasing or decreasing the particle count from *N* to N+1 or N−1, respectively. According to the Metropolis algorithm [199], these probabilities are defined as $W_{ads(des)}=\min\left\{1,\exp\left(-\beta \Delta H\right)\right\}$, where $\Delta H=H_{f}-H_{i}$ represents the change in the system’s Hamiltonian between the final and initial configurations.
(iv) Repeat steps (ii) and (iii) a total of *M* times.

The first $m^{\prime }$ MCSs in each run are discarded to allow for equilibrium and the next *m* MCSs are used to compute averages. In the low-temperature regime, where ordered phases are expected to develop, displacement (diffusional relaxation) of adparticles to nearest-neighbor positions—by either jumps along the *k*-mer axis or reptation by rotation around the *k*-mer end—must be allowed in order to reach equilibrium in a reasonable time.

#### 8.1.2. Canonical Ensemble

In the canonical ensemble framework, thermodynamic equilibrium is achieved using Kawasaki dynamics [198], extended to accommodate polyatomic species. The procedure for executing a single Monte Carlo step (MCS) is as follows:

Consider a square lattice composed of *M* identical adsorption sites:

(i) Define the system temperature *T*.
(ii) Fix the surface coverage θ=kN/M by placing N=M/2k linear molecules onto the lattice, each occupying *k* adjacent sites.
(iii) Randomly choose one *k*-mer and a linear sequence of *k* unoccupied lattice sites. Once their positions are determined, an exchange of their occupancy states is attempted. The acceptance of this move follows the Metropolis criterion [199]:
  (305) $$W=\min\left\{1,\exp\left(-\beta \Delta H\right)\right\}$$
  where $\Delta H=H_{f}-H_{i}$ represents the energy difference between the final and initial configurations.
(iv) Select a *k*-mer at random and attempt a movement to neighboring sites. This movement may be either a translational shift along the molecule’s axis or a reptation move involving rotation around one of its units. These diffusion steps are governed by the Metropolis rule and are essential for ensuring that the system relaxes toward equilibrium within practical simulation timeframes.
(v) Repeat steps (iii) and (iv) a total of *M* times.

Similar to the grand canonical Monte Carlo simulations discussed in Section 8.1.1, the system was allowed to reach equilibrium by discarding the initial $m^{\prime }$ Monte Carlo steps, after which statistical averages were computed over the subsequent *m* steps.

### 8.2. Parallel Tempering MC Algorithm for Adsorption of Interacting k-mers

When simulating *k*-mers of increasing length, the standard grand canonical Monte Carlo (MC) algorithm (Section 8.1.1) typically suffers from slow dynamics, particularly at medium to high coverage. This limitation necessitates the use of alternative algorithms. An example of such an approach is the hyper-parallel tempering Monte Carlo (HPTMC) algorithm [200]. This technique involves constructing a composite ensemble made up of *R* independent replicas of the original system. Each replica operates at a distinct pressure $P_{i}$, selected from a carefully chosen set $P_{i}$ [201]. The number of pressure values sampled is determined by targeting an acceptance rate of 0.5 for exchange attempts between neighboring replicas. Once the values of the gas pressure or the chemical potential are established, the simulation process consist in two major subroutines: *replica-update* and *replica-exchange*.

#### 8.2.1. Replica-Update

The adsorption–desorption procedure is as follows: (i) One out of *R* replicas is randomly selected; (ii) a linear *k*-tuple of nearest-neighbor sites is selected. Then, if the *k* sites are empty, an attempt is made to deposit a rod with probability $W_{ads}$; if the *k* sites are occupied by units belonging to the same *k*-mer, an attempt is made to desorb this *k*-mer with probability $W_{des}$ and, otherwise, the attempt is rejected. As in the previous section, $W_{ads(des)}=\min\left\{1,\exp\left(-\beta \Delta H\right)\right\}$.

#### 8.2.2. Replica-Exchange

The exchange of two configurations $\chi_{i}$ and $\chi_{j}$, corresponding to the *i*-th and *j*-th replicas, respectively, is tried and accepted with probability $W_{accep}\left(\chi_{i}\to \chi_{j}\right)=\min\left\{1,\exp\left(\beta \Delta \right)\right\}$, where Δ in a non-thermal grand canonical ensemble is given by

(306) $$\Delta =-\left[\left(\mu_{k}\left(j\right)-\mu_{k}\left(i\right)\left(N_{k}\left(j\right)-N_{k}\left(i\right)\right)\right)\right].$$

### 8.3. Parallel Tempering MC Algorithm for Adsorption of Binary Mixtures of Interacting Species of Polyatomics

The algorithms described above—particularly the HPTMC method—can be extended to handle binary mixtures composed of two different species. To model the adsorption of such mixtures consisting of *k*-mers and *l*-mers, we consider a substrate represented by a regular lattice with connectivity *c*. The HPTMC approach involves constructing a set of *R* non-interacting replicas of the system, each associated with a distinct pressure $P_{i}$ selected from a carefully designed set $P_{i}$ [201]. The number of pressure levels is determined by aiming for an exchange acceptance probability of approximately 0.5 between neighboring replicas.

Once the total pressure of the gas mixture and the molar fractions $X_{x}$ are specified, the chemical potential for each component is calculated assuming ideal mixture behavior. Specifically, the chemical potential of species *x* (where x=k or *l*) is given by $\mu_{x}=\mu_{x}^{0}+\ln\left(X_{x}P\right)$, with $\mu_{x}^{0}$ denoting the standard chemical potential at temperature *T*.

Based on these parameters, the simulation proceeds through two principal stages: the *replica-update*, which governs local changes within each replica, and the *replica-exchange*, which facilitates swaps between neighboring replicas to enhance sampling efficiency.

#### 8.3.1. Replica-Update

The adsorption–desorption procedure is as follows: (i) One out of *R* replicas is randomly selected; (ii) the species *x* is selected with equal probability from the two species *k* and *l*; (iii) a linear *x*-tuple of nearest-neighbor sites is selected. Then, if the *x* sites are empty, an attempt is made to deposit a rod with probability $W_{ads}$; if the *x* sites are occupied by units belonging to the same *x*-mer, an attempt is made to desorb this *x*-mer with probability $W_{des}$ and, otherwise, the attempt is rejected.

#### 8.3.2. Replica-Exchange

The exchange of two configurations $\chi_{i}$ and $\chi_{j}$, corresponding to the *i*-th and *j*-th replicas, respectively, is tried and accepted with probability $W_{accep}\left(\chi_{i}\to \chi_{j}\right)=\min\left\{1,\exp\left(\beta \Delta \right)\right\}$, where Δ in a non-thermal grand canonical ensemble is given by

(307) $$\Delta =-\left[\left(\mu_{k}\left(j\right)-\mu_{k}\left(i\right)\left(N_{k}\left(j\right)-N_{k}\left(i\right)\right)\right)+\left(\mu_{l}\left(j\right)-\mu_{l}\left(i\right)\left(N_{l}\left(j\right)-N_{l}\left(i\right)\right)\right)\right].$$

The complete simulation procedure is as follows: (1) replica-update, (2) replica-exchange, and (3) repeat from step (a) R×M times. This is the elementary step in the simulation process or Monte Carlo step (MCS). Typically, the equilibrium state is reached after discarding the first $m^{\prime }$ MCSs. Then, the next *m* MCSs are used to compute averages.

For each value of pressure $P_{i}$, the corresponding surface coverages are determined via simple averages

(308) $$\theta_{x}\left(j\right)=\frac{1}{r}\sum_{t=1}^{r}\theta_{x}\left[\chi_{j}\left(t\right)\right]\;\left\{x=k,l\right\},$$

where $\chi_{j}\left(t\right)$ represents the state of replica *j* at the Monte Carlo time *t*.

### 8.4. Improving the Update Algorithm Through the Use of Lists of Full and Empty k-tuples

The efficiency of the abovementioned algorithms can be significantly enhanced by employing lists that track fully occupied and completely empty *k*-tuples. In the replica-update procedure, a linear *k*-tuple is randomly selected from the system. This selected group may be fully occupied by a single *k*-mer, entirely vacant, or partially filled (by one or several *k*-mers). In cases of partial occupancy, the update attempt must be rejected. However, by using separate lists of full and empty *k*-tuples, one can carry out this selection in a rejection-free manner. The overhead associated with maintaining these lists is minimal compared to the computational benefit of avoiding failed updates due to partial occupancy.

For the HPTMC approach, the update process proceeds as follows: (i) one of the *R* replicas is chosen at random; (ii) a *k*-tuple is then randomly selected from the combined list of full and empty *k*-tuples associated with the chosen replica. This selected *k*-tuple is either entirely empty or fully occupied by a single *k*-mer. If the *k*-tuple is unoccupied, a rod insertion is attempted with probability $W_{ads}$; if it is occupied, a removal attempt is made with probability $W_{des}$. After a successful transition, the lists of *k*-tuples are updated accordingly to reflect the new state.

### 8.5. Non-Local Update Kundu’s Algorithm for Adsorption of Non-Interacting Large k-mers (Only Excluded Volume Interaction)

Simulations of *k*-mers lattice gases were carried out in the Grand Canonical Ensemble through an efficient algorithm introduced by Kundu et al. [63,129,137], in order to overcome the slowdown at very high density due to jamming effects. The temperature, chemical potential βμ, and system size are kept fixed and the number of particles on the lattice is allowed to fluctuate through non-local changes; i.e, insertion and deletion of many *k*-mers at a time (in contrast to the standard Metropolis rule used in previous algorithms).

**Figure 57:**
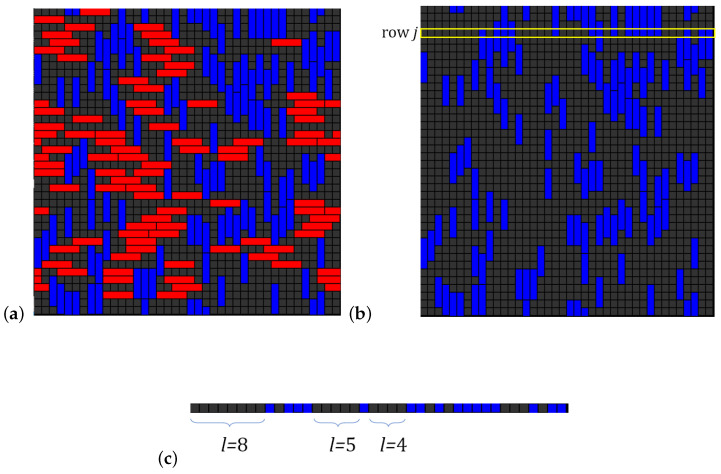
Example of a system of tetramers on a square lattice. (**a**) Current state. (**b**) Horizontal tetramers removed. (**c**) Detail of row *j* with three segments (only segments with l≥4 are showed).

Given a specific arrangement of *k*-mers on a square lattice, a Monte Carlo step (MCS) is performed by first removing all horizontally oriented *k*-mers while leaving the vertical ones unchanged. This step is illustrated in Figure 57a,b. When viewing the system along the horizontal direction, one observes sequences of empty sites of varying lengths, each bounded by vertical *k*-mers; as can be seen, for instance, in row *j* of Figure 57b.

**Figure 58:**
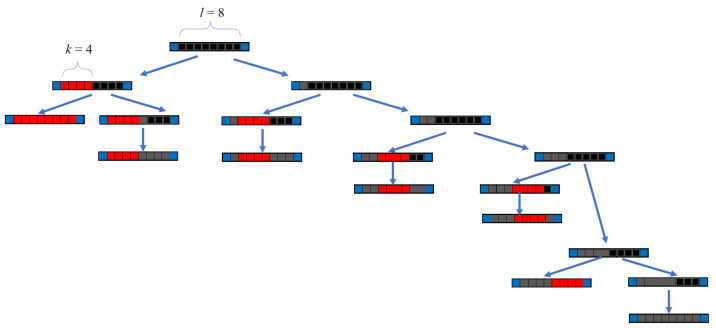
Refilling process of an l=8 segment by tetramers. Each branch is made with pre-calculated probabilities of the 1D case that depend on *T* and μ. Red represents a deposited *k*-mer and grey represents a deposited empty site.

The probabilities of finding horizontal segments of unoccupied sites can be computed exactly for all the possible segment lengths, as the problem effectively reduces to a one-dimensional case. These probabilities are pre-computed and stored. Subsequently, each horizontal segment is refilled with *k*-mers and empty sites based on the corresponding probabilities (as depicted in Figure 58). The entire procedure is then repeated along the vertical direction, this time removing vertical *k*-mers and keeping the horizontal ones.

Figure 57 shows the process of removal of horizontal (red) *k*-mers, as well as the identification of three segments in a row. Figure 58 shows the process of occupation of an l=8 segment. The algorithm can be easily generalized to other geometries and dimensions. A detailed discussion of the algorithm can be found in the original work Refs. [63,129,137]. The algorithm has shown to be ergodic, such that it satisfies the Detailed Balance Principle and equilibrium is typically reached after $10^{7}$ MCSs.

### 8.6. Thermodynamic Integration Method in Canonical Ensemble: Artificial Hamiltonian Method

The benefits of applying Monte Carlo simulations to compute thermal averages of thermodynamic properties are well established [202]. Quantities such as the total energy, its fluctuations, and various correlation functions can be readily obtained by averaging over a sufficiently large ensemble of microstates sampled from the system’s phase space. However, the direct evaluation of free energy and entropy remains challenging, as these quantities are not straightforwardly accessible from standard Monte Carlo sampling.

To overcome this limitation, a variety of techniques have been proposed. These include the thermodynamic integration method (TIM) [94,95,202,203,204,205], Ma’s coincidence-counting approach [206], the stochastic models technique introduced by Alexandrowicz [207], Meirovitch’s local states method [208], as well as the multistage and umbrella sampling methods developed by Valleau and collaborators [209,210,211,212]. Other notable approaches include Salsburg’s method [213] and the strategy of Yip et al. [214], which combines the coupling parameter technique with adiabatic switching in an optimized framework. Among these, the thermodynamic integration method stands out for its broad applicability and practical utility. A brief overview of this method is presented below.

Consider a lattice–gas system composed of *N* interacting particles distributed over a regular lattice of *M* sites at temperature *T*. Starting from the fundamental relation

(309) $${\left(\partial S/\partial T\right)}_{N,M}=\frac{1}{T}{\left(\partial U/\partial T\right)}_{N,M}$$

it follows that

(310) $$S\left(N,M,T\right)=S\left(N,M,T_{o}\right)+\int_{T_{o}}^{T}\frac{dU}{T}$$

where *U* is the mean total energy of the system.

The entropy S(N,M,T) can be readily determined if the entropy at a reference state $S(N,M,T_{0})$ is known, as the integral in the second term can be accurately evaluated using Monte Carlo simulations. However, in practice, obtaining the entropy in a reference state through analytical methods is only feasible in very limited scenarios. While some extreme cases—such as the low-density limit where S→0 as N→0—yield trivial entropy values, these are often impractical for computational purposes, as they require simulating an open thermodynamic system to extrapolate the entropy at finite density.

An alternative strategy involves performing integration along a thermodynamic path within a closed (mechanically isolated) system, maintaining constant density throughout. This approach becomes viable if a suitable reference state is selected; that is, one for which the entropy $S(N,M,T_{0})$ can be directly calculated.

In the case of monomers (k=1), the determination of the entropy in the reference state is trivial. In fact, for a monoatomic lattice–gas,

(311) $$S\left(N,M,T_{o}=\infty \right)=k_{B}\ln\left(\begin{array}{c} M\\ N \end{array}\right).$$

This equation holds for any finite value of the lateral interactions between the adparticles.

As S(N,M,∞) cannot be exactly calculated for *k*-mer adsorption (k≥2) by analytical means, in the following, we present a general numerical methodology to obtain the entropy of generalized lattice–gas in a reference state.

If an artificial lattice–gas is defined from the system of interest (henceforth referred to as the original system), such that it fulfills the condition

(312) $$S_{A}\left(N,M,\infty \right)=S\left(N,M,\infty \right)$$

(313) $$S_{A}\left(N,M,0\right)=0,$$

then the integral in Equation (310) can be separated into two terms. Thus,

(314) $$\begin{array}{ccc} S(N,M,T) & = & S_{A}\left(N,M,\infty \right)+\int_{\infty }^{T}dU/T\\ & = & S_{A}\left(N,M,0\right)+\int_{0}^{\infty }dU_{A}/T+\int_{\infty }^{T}dU/T\\ & = & \int_{0}^{\infty }dU_{A}/T+\int_{\infty }^{T}dU/T \end{array}$$

where $U_{A}$ and *U* are the mean total energy of the artificial and original system, respectively (both integrals can be evaluated via MC in the canonical ensemble). The general definition of the artificial reference system follows.

Consider a discrete system composed of *N* particles distributed over *M* lattice sites, where the system’s Hamiltonian is defined as H(N,M,i)=U(N,M,i) with i∈γ. Here, U(N,M,i) represents the potential energy associated with the *i*th configuration in the set of all accessible microstates γ, and is assumed to be finite for all i∈γ. The system is constrained to explore only the configurations within this set. The total number of accessible configurations is denoted by $G_{T}\left(N,M\right)$. For instance, in a lattice gas composed of *N* monomers with single-site occupancy on *M* sites, the total number of configurations is given by $G_{T}\left(N,M\right)=M!/\left[N!\left(M-N\right)!\right]$.

Now, let us define an auxiliary (or artificial) system with a modified Hamiltonian $H_{A}$, constructed as follows:

Definition 1: The Hamiltonian of the auxiliary system is given by $H_{A}\left(N,M,j\right)=U_{A}\left(N,M,j\right)$, where $j\in \gamma_{A}$ and $U_{A}$ is finite for all configurations in $\gamma_{A}$. The sets γ and $\gamma_{A}$ are assumed to be identical, meaning that both systems share the same configuration space. However, while the configuration sets are equivalent, the potential energy values assigned to each configuration may differ between the original and auxiliary systems.

Definition 2: The potential energy values $U_{A}\left(N,M,j\right)$ assigned to each configuration $j\in \gamma_{A}$ in the artificial system are defined as follows:

(315) $$\begin{array}{c} U_{A}\left(N,M,j_{o}\right)=0\;\;\;\;\;\;\;j_{o}\in \gamma_{A}\;\;\;\;\;\;\;\;\;\;\;\;\\ U_{A}\left(N,M,j\right)>0\;\;\;\;\;\;j\neq j_{o}\;\;\;\;j\in \gamma_{A}. \end{array}$$

Definition 2 means that a given configuration (the $j_{0}$ th) is selected arbitrarily from $\gamma_{A}$ and defined as the non-degenerate ground state of the artificial lattice–gas; hence, $S_{A}\left(N,M,0\right)=0$. In practice, the configuration $j_{0}$ can be easily defined.

**Figure 59:**
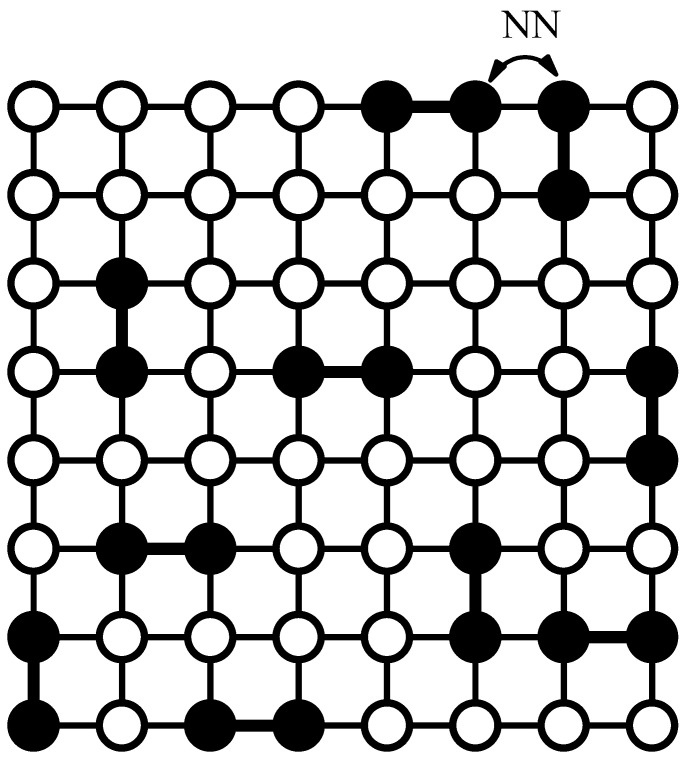
Configuration of N=10 dimers adsorbed onto a square lattice composed of M=64 sites. Representative nearest-neighbor (NN) interactions are indicated. This image illustrates the original system under investigation.

An example regarding adsorbed dimers follows, in order to make this point clear. Let us consider adsorbed dimers on an homogeneous square lattice with $b_{ij}=1$∀〈i,j〉 and the interaction between NN dimer heads as shown in Figure 59 (original system). For this system, there is no rigorous expression of s(N,M,∞) for N>0 in the thermodynamic limit (N→∞, N/M→ constant).

**Figure 60:**
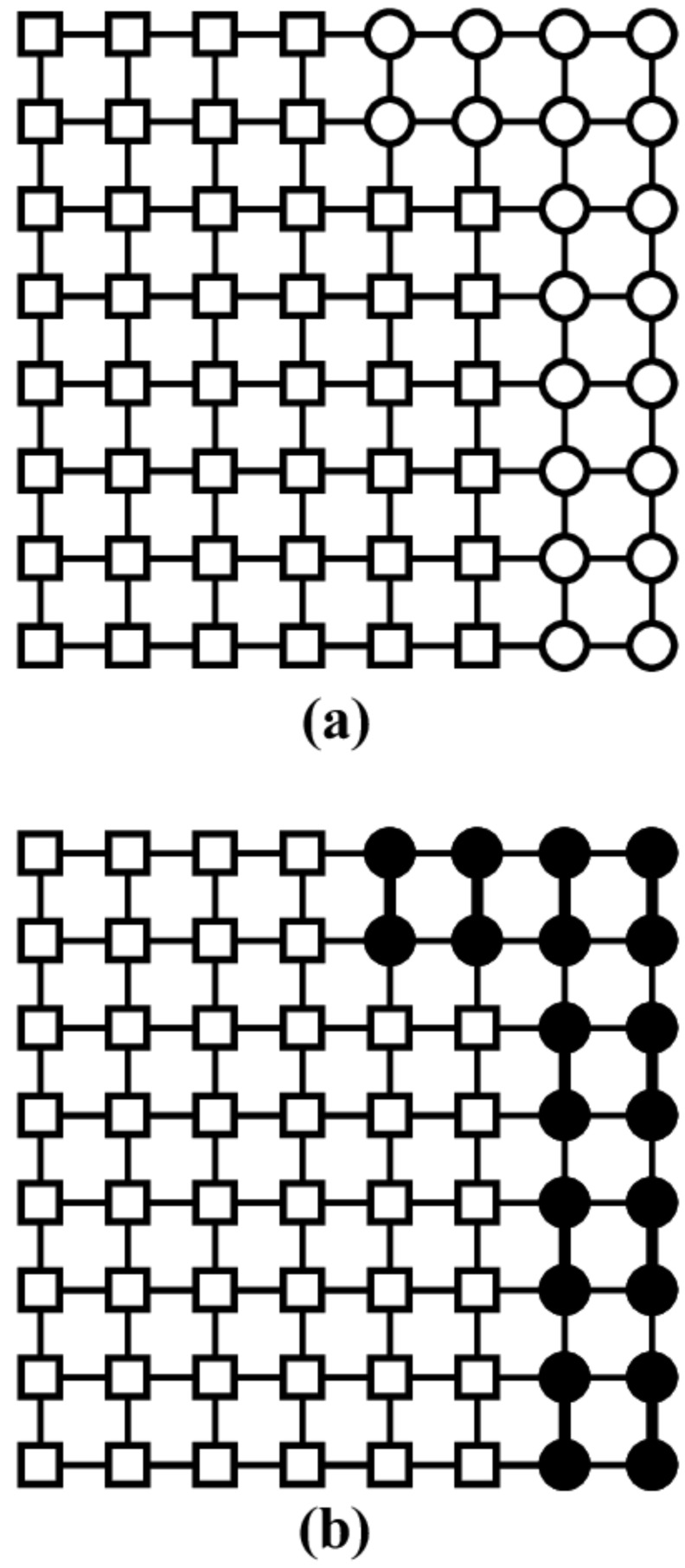
(**a**) Schematic representation of an artificial lattice consisting of M=64 sites. Strong and weak adsorption sites are depicted as circles and squares, respectively. (**b**) Ground- state configuration of N=10 dimers, obtained using the artificial Hamiltonian defined in Equation (316).

To construct an auxiliary system that satisfies Definitions 1 and 2, the following procedure is applied:

(i) The number of particles, lattice dimensions, and geometry are retained exactly as in the original system.

(ii) All nearest-neighbor interaction energies between adsorbed units are set to zero.

(iii) Site-specific adsorption energies are introduced to model the interaction between the adsorbed dimer units and the substrate in the auxiliary system. Two categories of lattice sites are defined: strong and weak, with corresponding adsorption energies $\epsilon_{S}$ and $\epsilon_{W}$, where $\epsilon_{S}<\epsilon_{W}$. For a system containing *N* adsorbed dimers, a total of 2N strong adsorption sites are strategically placed on the lattice. For example, Figure 60a illustrates one such spatial arrangement, with circles representing strong sites ($\epsilon_{S}$) and squares indicating weak sites ($\epsilon_{W}$).

(iv) To energetically favor dimers aligned in a specific direction, an external field is conceptually applied. This is modeled by assigning an interaction energy $w_{n}=-1$ for vertically oriented dimers and $w_{n}=0$ for all others—note that this is a convention chosen for convenience. When applying periodic boundary conditions, care must be taken to ensure the uniqueness of the ground state. Under these assumptions, the Hamiltonian of the artificial system can be expressed as:

(316) $$H_{A}=\sum_{i=1}^{M}\epsilon_{i}c_{i}+\sum_{n=1}^{N}w_{n}$$

where $\epsilon_{i}=\epsilon_{S}=-1$ if the site is strong and $\epsilon_{i}=\epsilon_{W}=0$ if the site is weak.

Consequently, the ground state configuration of the auxiliary system corresponds to the arrangement depicted in Figure 60b. This configuration is unique (non-degenerate), implying that the ground-state entropy is zero; i.e., $s_{A}\left(N,M,0\right)=0$.

The evaluation of s(N,M,T) via Equation (314) is both conceptually straightforward and computationally efficient. This is because the temperature-dependent internal energies $u_{A}\left(T\right)$ and u(T) can be estimated at fixed coverage using standard Monte Carlo simulations within the canonical ensemble framework (refer to Section 8.1.2), employing the well-known Metropolis algorithm [199]. The internal energy values for both the original and auxiliary systems are computed as ensemble averages, then smoothed using spline interpolation and numerically integrated.

**Figure 61:**
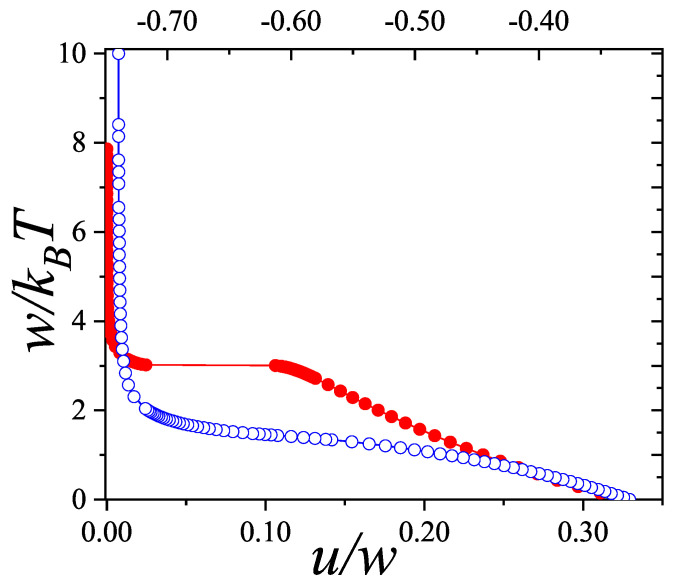
Average total energy per lattice site (expressed in units of the pair interaction energy *w*) for a system of dimers on a square lattice with nearest-neighbor interactions. The coverage is fixed at θ=0.5. Results for attractive interactions are shown as open circles and refer to the top *x*-axis. Results for repulsive interactions are shown as filled circles and refer to the bottom *x*-axis. Simulations were performed in the canonical ensemble. Each data point represents an average over approximately $m=10^{6}$ MC configurations, following an equilibration period of $m^{\prime }=10^{5}$ to $10^{6}$ MC steps.

Specifically, $u_{A}\left(T\right)$ is derived using the Hamiltonian defined in Equation (316), while u(T) corresponds to calculations based on the original system’s Hamiltonian. Figure 61 displays representative plots of $1/k_{B}T$ as a function of internal energy *u* for the cases of both attractive and repulsive dimer interactions on a square lattice.

The methodology outlined above is versatile and can be generalized to a broad range of lattice–gas models.

## 9. Conclusions and Future Perspectives

This review systematically explored the thermodynamic behaviors and statistical mechanics of rigid rods (or *k*-mers) adsorbed onto regular lattices, focusing on equilibrium properties as a function of density, temperature, and particle size. Through a sequence of progressively refined approaches—ranging from exact results in one dimension to numerical simulations and mean-field approximations in higher dimensions, and culminating in the analytical development of Multiple Exclusion (ME) statistics—a broad and coherent framework for understanding collective adsorption phenomena governed by excluded volume effects was developed.

In Section 2, the review focused on multilayer adsorption of *k*-mers on one-dimensional (1D) lattices, which offer the unique opportunity of exact solvability. The treatment of *k*-mers in the multilayer regime bears an elucidating relation with analogous experimental realizations such as the adsorption of linear molecules (alkanes, alkenes) on quasi-regular surfaces of carbon nanotubes, demonstrate that the size/shape entropic contributions of molecules to free energy cannot be oversimplified when interpreting adsorption isotherms to determine the specific surface of the adsorbent.

The 1D treatment set the basis for extending the understanding to higher dimensions through the introduction of coarse-grained approaches that effectively capture key thermodynamic quantities. Here, the notion of excluded volume per particle is generalized, leading to approximate expressions for entropy and pressure that remain remarkably accurate across a broad range of densities. A number of approximations were reviewed, ranging from the configurational dimensional ansatz to extend the 1D analytical forms to higher dimensions through the EA approximation, the conceptualization of the challenging problem of the structured particles lattice gas as being isomorphic to fractional exclusion statistics in quantum systems with independent states through FSTA, up to the introduction of the most elaborate theoretical approach—named Multiple Exclusion Statistics—embodying the complexity emerging from spatial correlations between particle states into a set of simple self-exclusion and cross-exclusion correlation parameters that can be consistently determined theoretically from limiting configurational conditions of the system.

The article then progressed to address more intricate systems in higher dimensions, for which exact solutions are generally not accessible. Here, mean-field approximations and phenomenological entropy-based models were employed to describe the emergence of phase transitions; particularly the isotropic-to-nematic transition observed for elongated particles. These transitions are governed not only by steric constraints but also by emergent orientational ordering, which is absent in the 1D case. The entropy per site as a function of coverage revealed non-trivial behaviors, including entropy-driven phase separations and coexistence regions, which can be tracked even in approximate analytical formulations.

Section 4 presented a series of heuristic and mean-field-like methods aimed at incorporating orientational degrees of freedom and spatial correlations in 2D and 3D lattices. The simple Bragg–Williams-type approximations provide qualitative insights into isotropic–nematic transitions and the roles of *k*-mer length. Improved approaches, such as those based on lattice free volume estimates or effective excluded area models—particularly the Quasi-Chemical approach—offer better agreement with simulation data. However, these approximations often neglect inter-particle correlations and fail to accurately describe critical behaviors.

The review took a major conceptual leap in Section 5 and Section 6, where the statistical description of the system was reframed through the lens of generalized exclusion statistics; particularly in the form of Multiple Exclusion (ME) statistics. This approach introduces the idea of state-counting based on effective exclusion rules that go beyond simple geometric packing. Systematically encoding how a particle’s presence affects the availability of nearby states, the ME formalism captures both entropic interactions and emergent correlations. This represents a profound shift from traditional lattice gas models toward a more abstract and generalizable statistical mechanics framework.

One of the most significant achievements of this formalism is the derivation of spectral exclusion functions, which encode the exclusion parameters between particle states. These functions allow for the construction of thermodynamic quantities such as the chemical potential and entropy from the statistical exclusion rules, circumventing the need for explicit partition functions. Furthermore, the ability to reproduce not only exact 1D results but also to approximate higher-dimensional behavior with improved accuracy underscores the power of the ME approach.

A key theoretical development discussed is the formalization of ME statistics to include multicomponent and geometrically complex particle mixtures. The introduction of state self-exclusion and cross-exclusion functions $e_{ij}\left(\theta \right)$ and $G_{ij}\left(\theta \right)$, as generalizations of chemical potential derivatives with respect to partial densities, allows for a thermodynamic characterization of complex exclusion scenarios. These spectral functions encode how the presence of particles of one species affects the availability of configurations for others, thus extending the exclusion principle into a spatially resolved thermodynamic descriptor.

The application of these ideas to realistic systems was also showcased (Section 7), such as adsorption onto heterogeneous surfaces, including the adsorption of alkanes onto carbon nanotubes as a prototype experimental realization to demonstrate the simplest multilayer adsorption model, thus generalizing the pioneer work of BET to determine adsorption energy and specific area of adsorbent when probe molecules are not ideally spherical. Furthermore, an application to crystal growth from aqueous solutions in the presence of structured impurities was exhibited, showing that the size and shape of impurities must be properly accounted for in the thermodynamic potentials, in order to understand how the crystal growth rate depends on the impurity concentration.

The application examples concluded with an in-depth analysis of the long-standing *k*-mer problem on the square lattice, approached through the advanced framework of Multiple Exclusion Statistics theory. This problem—which still presents several open questions, such as the lack of a unified theoretical explanation for the emergence of a nematic transition at the critical length k=7, and the nature of the high-density transition back to an isotropic phase near saturation—was revisited from the alternative and more comprehensive perspective provided by the ME formalism. Modeling the system as a mixture of two species of particles with distinct orientations, the free energy and entropy surfaces were analytically approximated across the full range of relative occupations along lattice directions. This approach enabled a detailed characterization of density branches, critical points, transition orders, and exclusion spectrum functions, offering new insights into the entropy–density relationship. The analysis revealed that k=7 is the minimal rod length required to undergo a nematic transition, while k=6—although close—does not satisfy the necessary conditions and remains in an isotropic phase even at high coverage.

Furthermore, the review explored the novel outcomes of ME statistics, such as the potential for extracting exclusion spectral functions from experimental data, inverting thermodynamic observables to infer structural information. This opens the possibility of using adsorption isotherms and fluctuation spectra not just as phenomenological descriptors, but as quantitative probes of configurational entropy and microstate topology. Remarkably, the theoretical framework of ME statistics provides a bridge between observable thermodynamic quantities (e.g., chemical potential versus density curves) and latent spatial correlations or ordering tendencies that are otherwise hidden. This fact opens new experimental possibilities: exclusion spectra may eventually be inferred from measurements of adsorption isotherms or compressibility, providing insights into microscopic ordering without relying on direct imaging.

Ultimately, the ME formalism proves to be a unifying language that is capable of handling complex interactions and geometry-induced constraints. It not only serves as a powerful lens for interpreting equilibrium properties of complex particle systems, but also sets the stage for future theoretical and experimental explorations into the geometry and thermodynamics of constrained configuration spaces.

Several open questions and research avenues arise from this unified perspective. First, the extension of ME statistics to non-lattice systems—including continuous 2D and 3D domains with quenched disorder or curved geometries—remains largely unexplored.

Its generality and compatibility with empirical data make it not only a theoretical construct, but also a bridge toward experimentally accessible quantities. From a computational standpoint, there is room to develop inverse statistical mechanical methods that reconstruct exclusion spectral functions directly from experimental data or Monte Carlo simulations. This would establish a concrete protocol for extracting statistical fingerprints from real systems, with potential applications in surface science, porous media, and biological adsorption.

Secondly, dynamical aspects such as adsorption/desorption kinetics, diffusion on fluctuating energy landscapes, or driven systems under external fields are fertile grounds for applying and testing the ME framework beyond equilibrium.

Looking ahead, the ME framework is poised to play a central role in the statistical mechanics of systems with constrained configurations, such as crowding in biological environments, adsorption in porous media, or active matter with limited motility space.

Finally, there is potential to link the ME statistics approach with field-theoretic and renormalization group methods, particularly in systems where critical behaviors or universality classes may be modified by spatial exclusion. The interplay between exclusion-driven entropy and geometric frustration also presents an intriguing challenge, especially in contexts where topology and boundary effects play a dominant role.

A whole Section 8 was devoted to reviewing the Monte Carlo techniques from the early Metropolis state sampling to the new, highly efficient non-local configuration update to overcome the slowdown in reaching equilibrium in lattice gases with state exclusion, particularly at high density. Furthermore, the artificial Hamiltonian technique was revisited as a powerful tool to calculate entropy at arbitrary density from elementary thermodynamic relations, taking advantage of efficient numerical sampling of equilibrium configurations.

Monte Carlo (MC) simulations serve as an essential tool to validate approximate analytical methods for single components and mixtures, as well as exploring the influence of the lattice geometry on the thermodynamic potentials. MC studies have uncovered rich phase behaviors, including continuous and discontinuous isotropic–nematic transitions, as well as layering and jamming effects near full coverage. These results have helped to clarify the limitations of earlier mean-field approximations and motivated the development of more refined theories. Importantly, the numerical data have also inspired empirical functional forms for entropy and chemical potential, which have been instrumental in bridging the gap between simulations and analytical descriptions. The multiple exclusion problem opens new questions concerning potential simulation techniques in systems with strong state correlations and geometrical constraints, exploiting the topological properties of the configuration space and graph theory.

In summary, this article accomplishes several goals: it consolidates a wide range of exact, approximate, and numerical results across spatial dimensions and *k*-mer lengths; it clarifies the domain of validity and limitations of various theoretical approaches; it reviews the recently presented Multiple Exclusion Statistics (ME) as a promising analytical tool that reconciles geometric exclusion under topological constrains and correlations with thermodynamic consistency; and it sets new perspectives for the application and testing of this novel analytical tool to a broader range of systems where spatial correlations between particle states dominate.

The synthesis of exact models, approximate theories, numerical validation, and generalized statistics presented in this review thus offers a comprehensive and forward-looking perspective on *k*-mer adsorption and excluded volume systems, with broader implications across condensed matter, materials science, and statistical physics.

## Figures and Tables

**Table 1:** Parameters used in the fitting of Figure 25.

	${\mathrm{CH}}_{4}$/$C_{2}H_{6}$	$C_{2}H_{6}$/$C_{3}H_{6}$
$g_{max}\times 10^{-2}$ (mol./g)	0.41	0.41
$K_{k}\times 10^{9}$ (${\mathrm{mmHg}}^{-1}$)	3.5	0.48
$K_{l}\times 10^{5}$ (${\mathrm{mmHg}}^{-1}$)	1.6	0.6

**Table 3:** Details of single -walled nanotube samples and gases used in the isotherm measurements.

Sample	Type	Weight (g)	Gas	Area/mol on Graphite (${Å}^{2}$)	Isotherm Temp (K)
SWNTs	HiPco	0.1727	methane	15.4 [170]	77
SWNTs	HiPco	0.325	ethane	21 [171]	165
SWNTs	HiPco	0.325	propane	28.8 [172]	190
SWNTs	HiPco	0.325	butane	32.7 [171]	220

**Table 4:** The parameters used to obtain the theoretical curves shown in Figure 48.

	Kubota and Mullin [193]		This Work	
Impurity	α	K ${\mathrm{(Mole Fraction)}}^{-1}$	k	$K_{k}$ ${\mathrm{(Mole Fraction)}}^{-1}$
CHCOOH	0.502	12.8	1 [Equation (283)]	$K_{1}=3.33$
CH_3COOH	0.532	39.6	2 [Equation (284)]	$K_{2}=2.20$
C_2H_5COOH	0.697	35.0	3 [Equation (285)]	$K_{3}=3.39$
C_3H_7COOH	0.850	35.6	4 [Equation (286)]	$K_{4}=5.38$

## Data Availability

Dataset available on request from the authors.

## Appendix A

The seven tables presented in this appendix provide a summary of the models and computational methodologies of relevance to this review.

**Table A1:** Information for the main models in the case of single adsorption of non-interacting *k*-mers.

Model and Reference	Mathematical Form of the Adsorption Isotherm
1D lattice, non-interacting *k*-mers, exact solution [20]	$\exp\left[\beta \left(\mu -k\epsilon_{0}\right)\right]=\frac{\theta {\left[1-\frac{\left(k-1\right)}{k}\theta \right]}^{k-1}}{{k(1-\theta )}^{k}}$
Flory–Huggins approximation, γ-connectivity lattice, non-interacting flexible *k*-mers [6,7]	$\exp\left[\beta (\mu -k\epsilon_{0})\right]=\frac{\theta }{k{\left(\gamma -1\right)}^{k-1}{\left(1-\theta \right)}^{k}}$
Flory–Huggins approximation, γ-connectivity lattice, non-interacting linear *k*-mers [6,7,16]	$\exp\left[\beta (\mu -k\epsilon_{0})\right]=\frac{2\theta }{k\gamma {\left(1-\theta \right)}^{k}}\;\;\;\;\left(k\geq 2\right)$
Guggenheim–DiMarzio approximation, γ-connectivity lattice, non-interacting linear *k*-mers [9,10]	$\exp\left[\beta \left(\mu -k\epsilon_{0}\right)\right]=\frac{2\theta {\left[1-\frac{\left(k-1\right)}{k}\frac{2\theta }{\gamma }\right]}^{k-1}}{{k\gamma \left(1-\theta \right)}^{k}}\;\;\;\;\left(k\geq 2\right)$
EA approximation, γ-connectivity lattice, non-interacting linear *k*-mers [16,20]	$\exp\left[\beta \left(\mu -k\epsilon_{0}\right)\right]=\frac{2\theta {\left[1-\frac{\left(k-1\right)}{k}\theta \right]}^{k-1}}{{k\gamma \left(1-\theta \right)}^{k}}\;\;\;\;\left(k\geq 2\right)$
FSTA approximation, γ-connectivity lattice, non-interacting linear *k*-mers [12,13]	$\exp\left[\beta \left(\mu -k\epsilon_{0}\right)\right]=\frac{\frac{2\theta }{k\gamma }{\left[1-\theta \frac{\left(k\gamma -2\right)}{k\gamma }\right]}^{\left(\frac{k\gamma }{2}-1\right)}}{{\left[1-\theta \right]}^{\frac{k\gamma }{2}}}\;\;\;\;\left(k\geq 2\right)$
SE approximation, γ-connectivity lattice, non-interacting linear *k*-mers [11,16]	$\exp\left[\beta \left(\mu -k\epsilon_{0}\right)\right]=\frac{2\theta {\left[1-\frac{\left(k-1\right)}{k}\frac{2\theta }{\gamma }\right]}^{\left(1-\theta )(k-1\right)}{\left[1-\frac{\left(k-1\right)}{k}\theta \right]}^{\theta (k-1)}}{{k\gamma \left(1-\theta \right)}^{k}}\;\;\;\;\left(k\geq 2\right)$

**Table A2:** Same as Table A1, but for non-interacting (*k*-mer–*l*-mer) binary mixtures.

Model and Reference	Mathematical Form of the Partial Adsorption Isotherms
1D lattice, exact solution [100]	$$\begin{array}{ccc} \beta (\mu_{i,ads}-\epsilon_{i}) & = & \left(i-1\right)\ln\left[1-\left(\frac{l-1}{l}\right)\theta_{l}-\left(\frac{k-1}{k}\right)\theta_{k}\right]\\ & & +\ln\frac{\theta_{i}}{i}-i\ln\left(1-\theta_{l}-\theta_{k}\right),\;\;\;\;i=\left\{k,l\right\} \end{array}$$
EA approximation, γ-connectivity lattice [97,98]	$$\begin{array}{ccc} \beta \left(\mu_{i,ads}-\epsilon_{i}\right) & = & \ln K_{i}\left(\gamma ,i\right)+\left(i-1\right)\ln\left[1-\left(\frac{k-1}{k}\right)\theta_{k}-\left(\frac{l-1}{l}\right)\theta_{l}\right]\\ & & +\ln\frac{\theta_{i}}{i}-i\ln\left(1-\theta_{k}-\theta_{l}\right),\;\;\;\;i=\left\{k,l\right\} \end{array}$$
GD approximation, γ-connectivity lattice [97,98]	$$\begin{array}{ccc} \beta \left(\mu_{i,ads}-\epsilon_{i}\right) & = & \left(i-1\right)\ln\left[\frac{\gamma }{2}-\frac{\left(k-1\right)}{k}\theta_{k}-\frac{\left(l-1\right)}{l}\theta_{l}\right]+\ln\left(\frac{\theta_{i}}{i}\right)\\ & & -i\ln\left(1-\theta_{k}-\theta_{l}\right)-i\ln\left(\frac{\gamma }{2}\right),\;\;\;\;i=\left\{k,l\right\} \end{array}$$
SE approximation, γ-connectivity lattice [97,98]	$$\begin{array}{ccc} \beta \left(\mu_{i,ads}-\epsilon_{i}\right) & = & \ln\left(\frac{\theta_{i}}{i}\right)-i\ln\left(1-\theta \right)-\ln\left(\frac{\gamma }{2}\right)\\ & & -\left(1-\theta \right)\left(i-1\right)\ln\left[1-\frac{\left(k-1\right)}{k}\frac{2\theta_{k}}{\gamma }-\frac{\left(l-1\right)}{l}\frac{2\theta_{l}}{\gamma }\right]\\ & & -\theta \left(i-1\right)\ln\left[1-\frac{\left(k-1\right)}{k}\theta_{k}-\frac{\left(l-1\right)}{l}\theta_{l}\right],\;\;\;\;i=\left\{k,l\right\} \end{array}$$

**Table A3:** Same as Table A1, but for non-interacting *k*-mers in the multilayer adsorption regime.

Model and Reference	Mathematical Form of the Adsorption Isotherm
1D lattice, dimers (k=2), exact solution [101,102]	$\theta =\frac{1}{\left(1-P/P_{0}\right)}\left\{1-{\left[\frac{\left(1-P/P_{0}\right)}{\left(4c-1\right)P/P_{0}+1}\right]}^{1/2}\right\}$
Non-interacting *k*-mers, γ-connectivity lattice [101,102]	(1) Use $\theta_{mon}$ as an input parameter (ranging from 0 to 1); determine the corresponding reduced pressure $P/P_{0}$ using $P/P_{0}=1/(1+c\lambda_{mon}^{-1})$ and an analytical expression for the monolayer adsorption isotherm (Table A1). (2) Substitute the values of $\theta_{mon}$ and $P/P_{0}$ into the total coverage $\theta =\theta_{mon}/\left(1-P/P_{0}\right)$ to compute θ.

**Table A4:** Same as Table A1, but for single adsorption of interacting *k*-mers.

Model and Reference	Mathematical Form of the Adsorption Isotherm
1D lattice, exact solution [30,31]	$$\begin{array}{ccc} & & k^{\left(1-k\right)}\exp\left[\beta (\mu -w-k\epsilon_{0})\right]=\\ & & \frac{\left[k(b-1+\theta )+\theta \right]}{\left[k(b+1-\theta )-\theta \right]}{\left[1-\frac{\left(k-1\right)}{k}\theta +b\right]}^{\left(k-1\right)} \end{array}$$
Mean-field approximation, γ-connectivity lattice [30,31]	$kK\left(\gamma ,k\right)\exp\left[\beta (\mu -k\epsilon_{0})\right]=\frac{\theta {\left[1-\frac{\left(k-1\right)}{k}\theta \right]}^{\left(k-1\right)}}{{\left(1-\theta \right)}^{k}}e^{\beta \lambda w\theta }$
Quasi-chemical approximation, γ-connectivity lattice [30,31]	$$\begin{array}{ccc} & & K\left(\gamma ,k\right){\left(\frac{2}{\gamma }\right)}^{2(k-1)}\exp\left[\beta \left(\mu -k\epsilon_{0}-w\lambda /2\right)\right]=\\ & & \frac{\theta }{k}\frac{{\left(1-\theta \right)}^{k(\gamma -1)}{\left[k-(k-1)\theta \right]}^{k-1}{\left[\frac{\lambda \theta }{2k}-\alpha \right]}^{\lambda /2}}{{\left[\frac{\gamma k}{2}-\left(k-1\right)\theta )\right]}^{k-1}{\left[\frac{\gamma }{2}\left(1-\theta \right)-\alpha \right]}^{k\gamma /2}{\left(\frac{\lambda \theta }{\gamma k}\right)}^{\lambda }} \end{array}$$

**Table A5:** Same as Table A1, but for interacting (*k*-mers-*l*-mers) binary mixtures.

Model and Reference	Mathematical Form of the Partial Adsorption Isotherms
1D lattice, exact solution [113]	See Section 4.3, deriving the final equations of the partial adsorption isotherms requires the use of a standard mathematical software program
Quasi-chemical approximation, γ-connectivity lattice [113]	See Section 4.3, deriving the final equations of the partial adsorption isotherms requires the use of a standard mathematical software program

**Table A6:** Same as Table A1, but for hard-core structured particles in the Multiple Exclusion (**ME**) theoretical framework.

Model and Reference	Mathematical Form of the Adsorption Isotherm
ME Statistics. General form [17,18,19]	$$\begin{array}{ccc} & & \beta (\mu -k\epsilon_{0})=\\ & & \ln\frac{\theta }{g}-\left[g_{c}e^{-\frac{g_{c}\theta }{g}}+ge^{-\frac{g_{c}}{g}}-\tilde{d_{s}}g\right]\ln\left[\tilde{d_{s}}\theta +e^{-\frac{g_{c}\theta }{g}}-\theta e^{-\frac{g_{c}}{g}}\right]\\ & & +\left[g_{c}e^{-\frac{g_{c}\theta }{g}}+ge^{-\frac{g_{c}}{g}}-\tilde{d_{s}}g-1\right]\ln\left[\theta \left(\tilde{d_{s}}+\frac{1}{g}\right)+e^{-\frac{g_{c}\theta }{g}}-\theta e^{-\frac{g_{c}}{g}}\right] \end{array}$$
ME Statistics. Simplified form assuming vanishing entropy at full coverage ($d_{s}=0$) [18]	$$\begin{array}{ccc} & & \beta (\mu -k\epsilon_{0})=\\ & & \ln\frac{\theta }{g}-\left[g_{c}e^{-\frac{g_{c}\theta }{g}}+ge^{-\frac{g_{c}}{g}}\right]\ln\left[e^{-\frac{g_{c}\theta }{g}}-\theta e^{-\frac{g_{c}}{g}}\right]\\ & & +\left[g_{c}e^{-\frac{g_{c}\theta }{g}}+ge^{-\frac{g_{c}}{g}}-1\right]\ln\left[\frac{\theta }{g}+e^{-\frac{g_{c}\theta }{g}}-\theta e^{-\frac{g_{c}}{g}}\right] \end{array}$$
ME Statistics. Mean-field approximation	Replace $\beta \left(\mu -k\epsilon_{0}\right)\to \beta \left(\mu -k\epsilon_{0}-\lambda w\theta \right)$ in above equations; λw is interaction energy per particle at full coverage

**Table A7:** Main algorithms and computational methods developed for the study of adsorption problems with multisite occupancy employed in this review.

Method/Algorithm and Reference	Main Features
Metropolis MC algorithm, grand canonical ensemble [20,142,198]	The state of the system is modified in one of two ways: by adsorbing a particle onto the substrate or desorbing it from the substrate
Metropolis MC algorithm, canonical ensemble [94,198]	Thermodynamic equilibrium is achieved using Kawasaki dynamics, extended to accommodate polyatomic species. One *k*-mer and a linear sequence of *k* unoccupied lattice sites are randomly selected. Once their positions are determined, an exchange of their occupancy states is attempted
Parallel tempering MC algorithm for single adsorption [200,201]	Starting from a compound system of *R* non-interacting replicas of the system under study, each of them associated to a different gas pressure $P_{i}$. There are two simulation steps: replica-update and replica-exchange. The first basically involves selecting one of the *R* replicas and applying the Metropolis algorithm to it. The second involves the random selection of two replicas and the intent of exchanging their states with a given probability
Parallel tempering MC algorithm for multicomponent adsorption [97,200,201]	The above algorithm for single adsorption can be applied to the case of mixtures of two or more species. In order to simulate the adsorption of a binary mixture (i.e., *k*-mers and *l*-mers), the application of the Metropolis algorithm must be preceded by the random selection with equal probability of one of the possible species
Update algorithm through the use of lists of *k*-tuples [102]	The updating process (replica-update), through application of the Metropolis rule, involves the random selection of a linear *k*-tuple. The use of lists of full and empty *k*-tuples allows this selection to be carried out in a rejection-free manner, which significantly improves the performance of the process
Non-local update Kundu’s algorithm [17,18,19,63,129,137]	The number of particles on the lattice is allowed to fluctuate through non-local changes; i.e, insertion and deletion of many *k*-mers at a time (in contrast to the standard Metropolis rule used in previous algorithms)
Thermodynamic integration method [94,95,202,203,204,205]	Free energy and entropy cannot be directly calculated using MC simulations. However, the thermodynamic integration method enables the estimation of entropy through the numerical integration of adsorption isotherms or other thermodynamic quantities, provided that the entropy of a reference state is known
Artificial Hamiltonian (AH) method [94,95]	Through the AH method, starting from the original system of interest, an artificial system that enables calculation of the entropy of the reference state can be constructed
